# The Mycobacterial cyt-*bc*_1_ *:aac_3_* Oxidase as a Drug Target: Activities of Arylvinylpiperazine Amides and Aminoquinazolines against *Mycobacterium ulcerans*

**DOI:** 10.1021/acs.jmedchem.6c01076

**Published:** 2026-07-14

**Authors:** Louisa Warryn, Matthias Witschel, Tobias Schehl, Daniela Prochazkova, Maryline Kienle, Francine Horn, Karl-Heinz Altmann, Daniel H. Paris, Gerd Pluschke

**Affiliations:** † 30247 Swiss Tropical and Public Health Institute, Allschwil 4123, Switzerland; ‡ University of Basel, Basel 4001, Switzerland; § 5184 BASF SE, Ludwigshafen 67056, Germany; ∥ Department of Chemistry and Applied Biosciences, Institute of Pharmaceutical Sciences, 27219 Swiss Federal Institute of Technology Zürich, Zürich CH-8093, Switzerland

## Abstract

Control of *Mycobacterium ulcerans* disease, or Buruli ulcer (BU), currently depends on identifying and treating every case. The recommended treatment regimen is the daily administration of rifampicin and clarithromycin for 8 weeks, and efforts are being made to develop new treatment regimens that are faster acting, easier to administer, and have fewer side effects. Repurposing new antitubercular drugs is an attractive strategy to reduce development costs of new BU treatments, and drugs targeting the mycobacterial ATP generation pathway are of special interest, given the success of bedaquiline and telacebec (Q203). In this SAR study, we analyzed derivatives of the arylvinylpiperazine amide (APA) and aminoquinazoline (AQ) scaffolds, which, like telacebec, target the mycobacterial respiratory cytochrome *bc*_1_ *:aa*_3_ supercomplex, leading to ATP depletion. Several APA derivatives had low micromolar activity against *M. ulcerans*, while many AQ derivatives showed nanomolar activity with excellent selectivity indices. Thus, we propose the most promising derivatives for further development.

## Graphical abstract

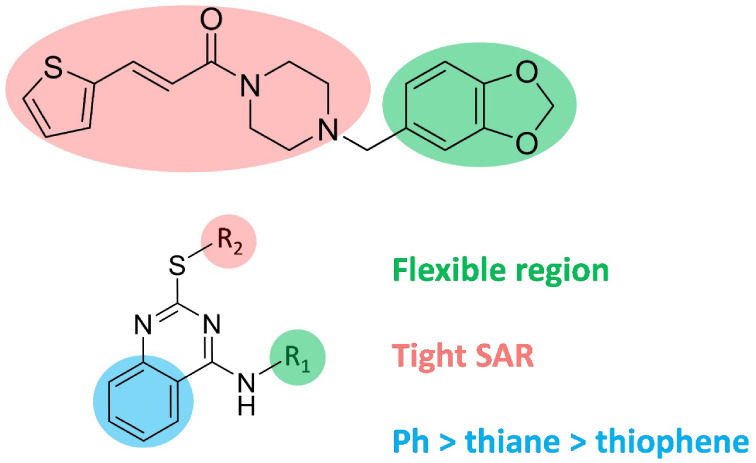

## Introduction

*Mycobacterium ulcerans* (*Mul*) is the etiological agent of Buruli ulcer (BU), a neglected tropical disease characterized by chronic and often devastating ulcers in skin and soft tissue. BU is highly endemic in parts of Central and West Africa and southeastern Australia. The current WHO-recommended treatment of BU involves a daily regimen of rifampicin and clarithromycin for 8 weeks.^1^ However, BU treatment is still less than optimal for several reasons, including the lack of effective replacements for rifampicin in the event of resistance development. Thus, the development of new drug regimens is still a key aspect of effective BU control. However, *de novo* drug discovery and development for BU are prohibitively expensive. Consequently, repurposing of drugs and drug candidates for other diseases, particularly tuberculosis (TB) caused by the related bacterium *Mycobacterium tuberculosis* (*Mtb*), is the most cost-effective way to develop new drugs for BU treatment. Such repurposing is an attractive approach, as the use of pharmacological, formulation, and safety data generated by TB research and development efforts allows the rapid advance of new candidate therapies for BU to clinical efficacy testing.^2^

Unfortunately, compounds active against *Mtb* are often inactive or only weakly active against *Mul*.^3^ Several reasons particular to *Mul* may contribute to this reduced sensitivity, including the loss of drug targets during its reductive evolution,^4^ its extremely slow growth rate,^5^ and the expression of a highly hydrophobic extracellular matrix (ECM).^6^ Nevertheless, a series of imidazopyridine amides, including the anti-TB clinical development candidate telacebec (Q203),^7^ have shown outstanding activity against *Mul*.^8^ Telacebec targets energy production in mycobacteria via interference with oxidative phosphorylation by inhibiting QcrB, the *b* subunit of the mycobacterial respiratory cytochrome *bc*_1_ *:aa*_3_ (cyt-*bc*_1_ *:aa*_3_) supercomplex (Figure 1). Oxidative phosphorylation as a drug target in mycobacteria had been previously validated by the long-established anti-TB drugs clofazimine and pyrazinamide and, more recently, the diarylquinoline bedaquiline as the first licensed anti-TB drug in over four decades.^9–11^ The latter inhibits ATP synthase and leads to direct ATP depletion (Figure 1).

**Figure 1:**
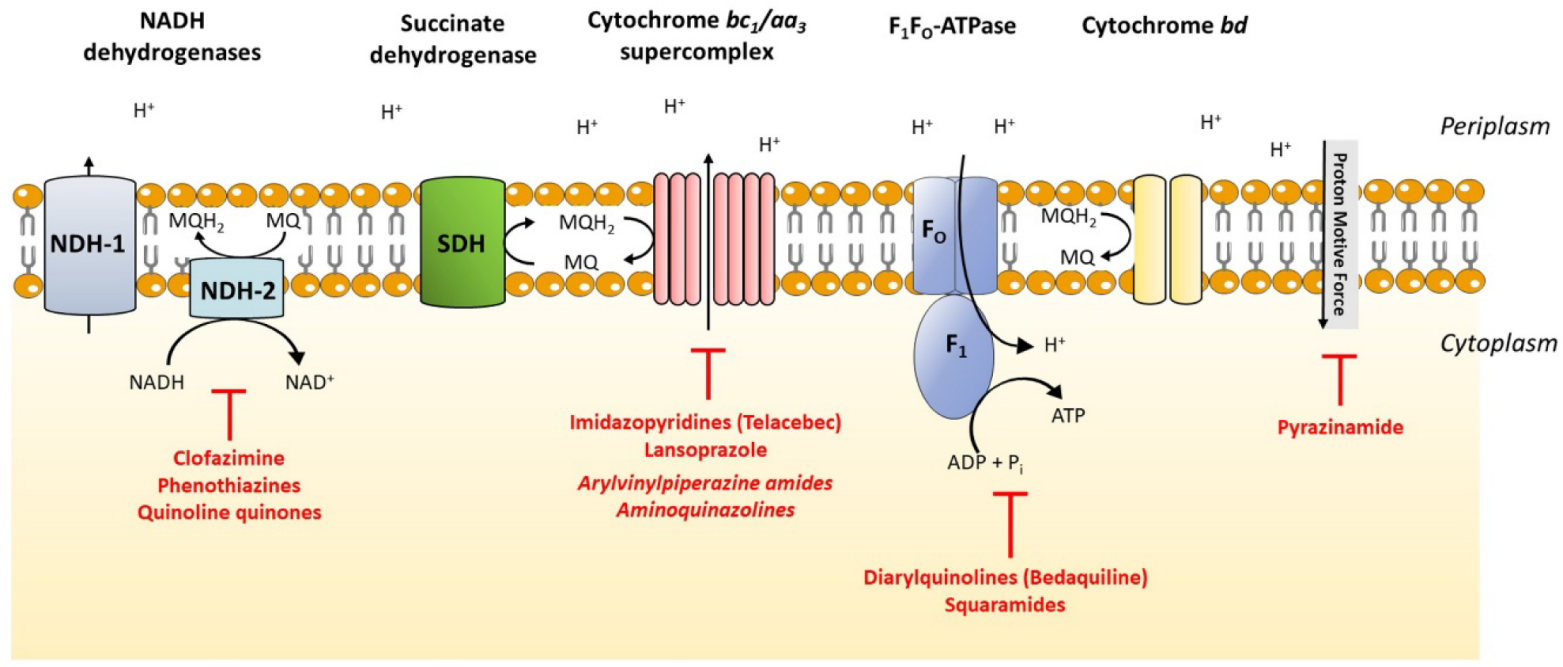
Schematic showing the mycobacterial electron transport chain with known and hypothesized inhibitors. Compound classes tested in this study are shown in italics. Abbreviations: Type I NADH dehydrogenase (NDH-1), Type II NADH dehydrogenase (NDH-2), Menaquinone (MQ). (Graphic generated using icons from SMART Servier Medical Art, www.servier.com).

Importantly, in contrast to *Mtb*, cyt-*bc*_1_ *:aa*_3_ is the sole terminal oxidase in *Mul* strains belonging to the classical lineage (i.e. strains found in African and Australian BU-endemic regions). As a consequence, telacebec and related cyt-*bc*_1_ *:aa*_3_ inhibitors exhibit very potent bactericidal activity against these *Mul* strains, whereas their bactericidal and sterilizing potency against *Mtb* and ancestral lineage *Mul* strains is limited by the presence of an alternate *bd*-type terminal oxidase.^8^ Thus, inhibition of QcrB represents a highly attractive strategy for the development of urgently needed new drugs for the treatment of BU. Additionally, given that a single dose of telacebec was curative in a mouse model of BU,^12^ telacebec and possibly other QcrB inhibitors could potentially simplify the currently recommended BU chemotherapy.

In addition to telacebec, other compounds have been recently identified as inhibitors of *Mtb* QcrB, including the proton pump inhibitor lansoprazole (used to treat peptic ulcer), the arylvinylpiperazine amides (APAs) AX-35 (GW861072X, **APA-1**) and AX-36 (**APA-2**), and the aminoquinazolines (AQs) **AQ-1** (GSK353069A) and **AQ-2/3/4** (Figure 2). **APA-1/2** ^13^ and **AQ-1** to **AQ-4** ^14,15^ are all potent inhibitors of *Mtb in vitro*; **APA-1** also demonstrated *in vivo* activity in an acute TB model in mice.^13^

**Figure 2:**
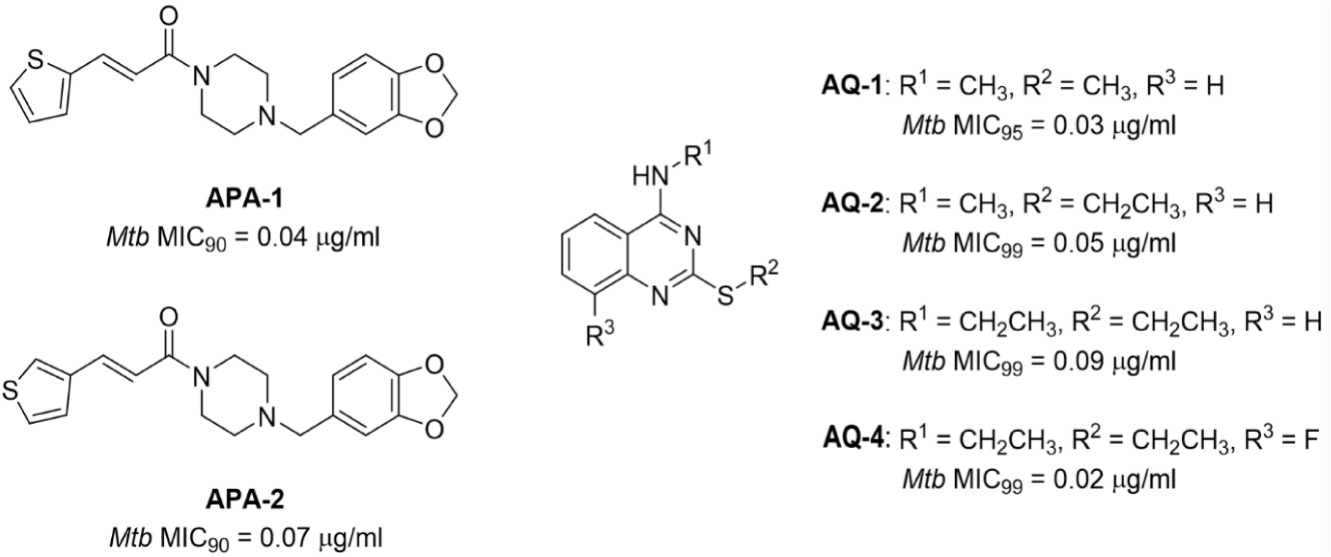
Arylvinylpiperazine amides (APAs) and aminoquinazolines (AQs) with reported activity against *M. tuberculosis* QcrB.^13–15^

As for telacebec, **APA-1** and its 3-thienyl isomer **APA-2** (Figure 2) have also been shown to inhibit the growth of *Mul* with MIC_90_s of 1.6 μg/mL for both compounds,^13^ while no data for *Mul* inhibition by the **AQ-1–4** have been reported. Given their simple modular structures, both the APAs and the AQs represent interesting leads for drug discovery against *Mul*, where low costs of goods are—as for any neglected tropical disease—of crucial importance for drug development. In order to gain a more detailed understanding of the structure–activity relationships of APAs and AQs with respect to inhibition of *Mul*, in this study, we have prepared a series of new analogs of **APA-1/2** and of **AQ-1** to **AQ-4**, and we have assessed their inhibitory activity against *Mul*. This has included the investigation of the differential activity of selected compounds against *Mul* strains belonging to the classical and ancestral lineages, as the latter have a cellular respiratory system more similar to that of *Mtb* with a functional *bd*-type terminal oxidase.^8^

## Results and Discussion

### Arylvinylpiperazine Amides

The lead arylvinylpiperazine amide AX-35 (GW861072X) (**APA-1**; Figure 2) was originally identified in a phenotypic high-throughput screen of a diverse library of low molecular weight compounds against *M. bovis* BCG and was also shown to be active against *Mtb* H37Rv.^14^ Subsequent work by Foo et al.^13^ identified **APA-1** as an inhibitor of *Mtb* QcrB but with a binding mode that appears to be different from that of telacebec. While telacebec-resistant mutants were also resistant to **APA-1**, the reverse was not always the case, with some **APA-1**-resistant mutants being susceptible to telacebec. Additionally, ATP levels were not affected in **APA-1**-resistant mutants in the presence of **APA-1** but were depleted in the presence of telacebec, indicating that both compounds differed in their interaction with QcrB.^13^

Consistent with the report by Foo et al.,^13^ **APA-1** was active against the classical lineage *Mul* strain S1013, with an MBC of 2–4 μg/mL, as determined by CFU enumeration, and of 0.91 μg/mL, as determined in the resazurin assay, following 4 weeks of exposure (Figure 3).

**Figure 3:**
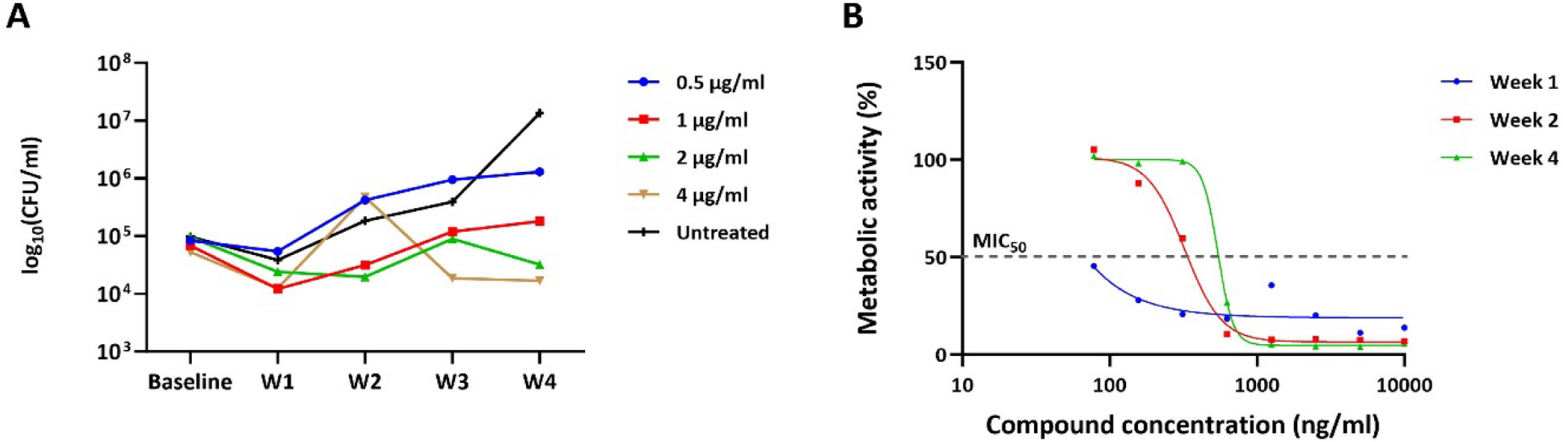
Time-kill assays for **APA-1**. Activity against the classical lineage *M. ulcerans* strain S1013 was determined by CFU enumeration (A) and by a resazurin microtiter assay in which the bacteria were incubated for 1, 2, or 4 weeks prior to 72 h of incubation with resazurin (B).

In order to determine the importance of the thiophene moiety in **APA-1/2** for anti-*Mul* activity, we first investigated the effects of replacing this moiety with other 5-membered heterocycles on the inhibitory activity against the classical *M. ulcerans* strain S1013. As can be seen from the data summarized in Table 1, replacement of the thiophene ring in **APA-1/2** with a corresponding thiazole moiety resulted in a clear loss in activity.

**Table 1:**
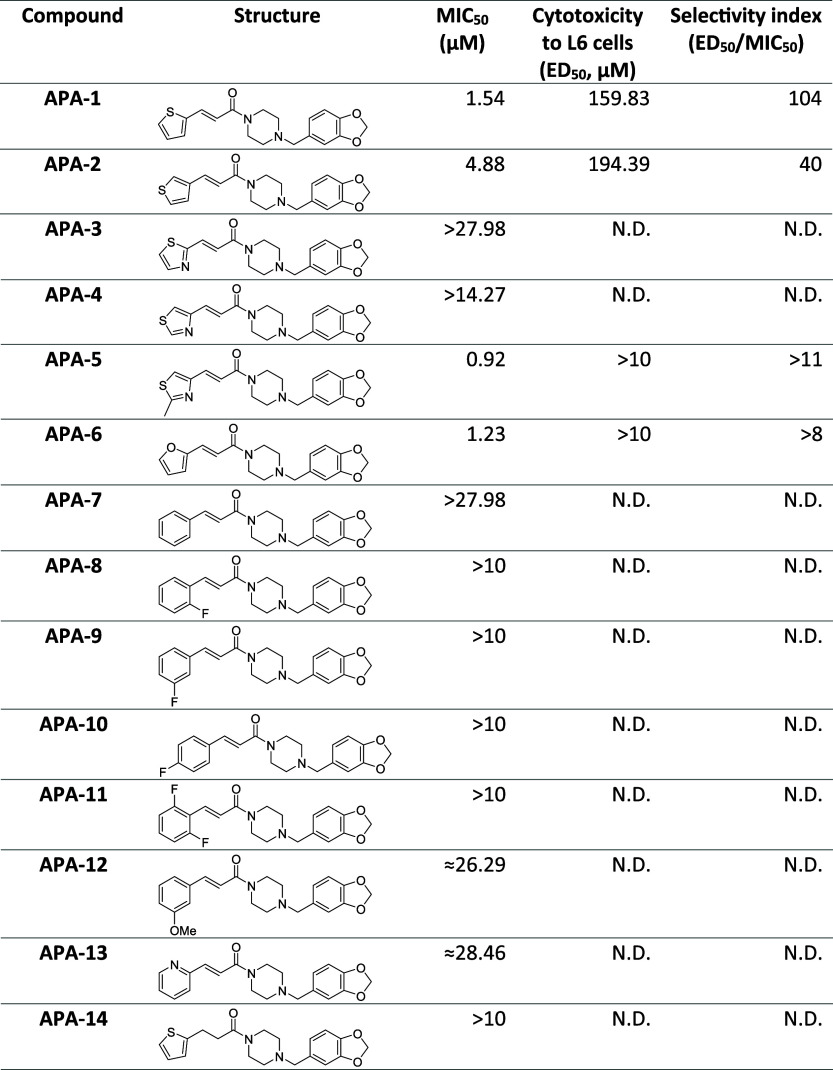
Activity of **APA-1/2** Analogs with Modifications/Replacements of the Thiophene Ring^a^,^b^

a MIC_50_ against the classical *M. ulcerans* strain S1013 was determined in a resazurin microtiter assay at 1 week postexposure.

b N.D. = Not determined.

Interestingly, the activity could be recuperated in methylthiazole-containing analog **APA-5**, which was equally active as **APA-1**. Likewise, anti-*Mul* activity was fully retained upon replacement of the thiophene moiety in **APA-1** by a furan ring (**APA-6**). We speculate that the loss in potency observed for thiazole derivatives **APA-3** and **APA-4** may be ascribed to their enhanced polarity and an associated decrease in cellular permeability. The latter may then be recovered by making the thiazole moiety in **APA-4** more lipophilic through methyl substitution. Interestingly, the activity difference between **APA-1** and **APA-3** against *Mtb* H37Rv is only 6-fold (MIC values of 0.05 μg/mL and 0.3 μg/mL, respectively, while **APA-2** and **APA-4** are equipotent).^13^ These differences once again highlight the distinctly different responsiveness of *Mtb* and *Mul* to the same inhibitors.

Rather surprisingly, the replacement of the thiophene moiety in **APA-1/2** with a bioisosteric phenyl ring (**APA-7**) resulted in a profound loss in inhibitory potency against *Mul*. Introducing fluorine substituents on the phenyl ring (**APA-8** to **APA-11**) or incorporating a N atom in the ring in the pyridine-containing analog **APA-13** did not recover activity. Like **APA-3** and **APA-4**, **APA-7** showed considerable activity against *Mtb* H37Rv, where it was found to be only 4-fold less active than **APA-1**. The reasons for this differential behavior are not obvious at this point, as **APA-7** is slightly more lipophilic than **APA-1** (the clogP values for **APA-1** and **APA-7** as calculated by ACD are 2.47 and 3.08, respectively). Finally, formal reduction of the olefinic double bond in **APA-1** gave an analog (**APA-14**) that had lost all activity.

In the next step, we explored the effects of modifications of the eastern benzodioxole moiety in **APA-1** on inhibition of *M. ulcerans* S1013. As illustrated by the data summarized in Table 2, modifications in this sector in general were well tolerated.

**Table 2:**
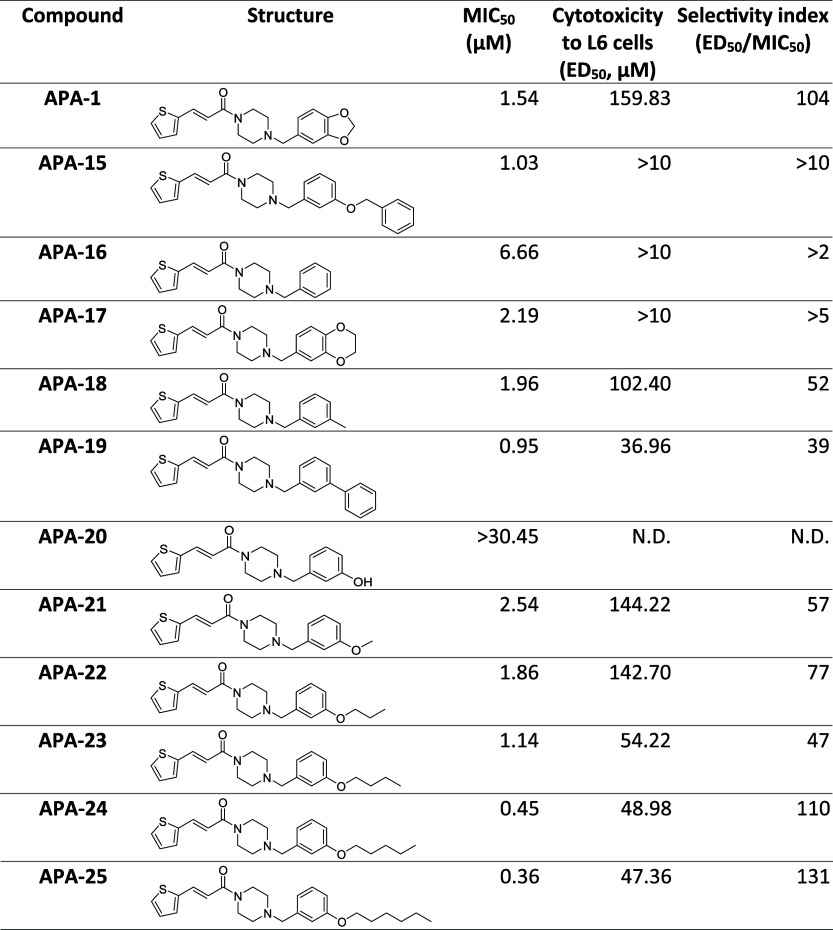
Activity of **APA-1/2** Analogs with Benzodioxole Modifications or Replacements^a^,^b^

a MIC_50_ against the classical *M. ulcerans* strain S1013 was determined by resazurin microtiter assay at 1 week postexposure.

b N.D. = Not determined.

In particular, the presence of a bicyclic structure is not essential. Analogs with monosubstituted benzyl groups retain or even surpass the activity of **APA-1**, depending on the specific substituent on the phenyl ring. As a notable exception, a free hydroxy group is not tolerated, with the corresponding analog **APA-20** being completely inactive. For the alkoxybenzyl derivatives **APA-21**–**APA-25**, there was a general trend toward increased activity against *Mul* with increasing length of the alkyl group, which might be due to an increase in overall hydrophobicity and better penetration of the waxy *Mul* cell wall and highly hydrophobic ECM. The most potent analogs investigated here were pentyloxy- and hexyloxybenzyl analogs **APA-24** and **APA-25**, respectively, which both showed lower MIC_50_ values than **APA-1**. However, the improvement in activity was paralleled by enhanced cytotoxicity to cultured rat L6 cells. Nevertheless, the selectivity indices of **APA-24** and **APA-25** were slightly superior to that of the lead compound **APA-1**, owing to their lower MIC_50_. Overall, the data in Table 2 suggest that the removal of the dioxole moiety from **APA-1** is tolerated if it is compensated for by the presence of a sufficiently hydrophobic substituent on the remaining benzyl group. At the same time, the increase in hydrophobicity appears to be associated with increased cytotoxicity, and further studies will be required to fine-tune the degree of hydrophobicity that will enable an optimal balance between potency and cytotoxicity.

Due to the added structural complexity, which would be undesirable for BU drug development, we have not extensively explored modifications of the piperazine core of **APA-1/2**. However, the lack of activity of methylpiperazine-derived analogs **APA-26** and **APA-27** suggests that substitution of the piperazine ring may not be tolerated (Table 3).

**Table 3:**
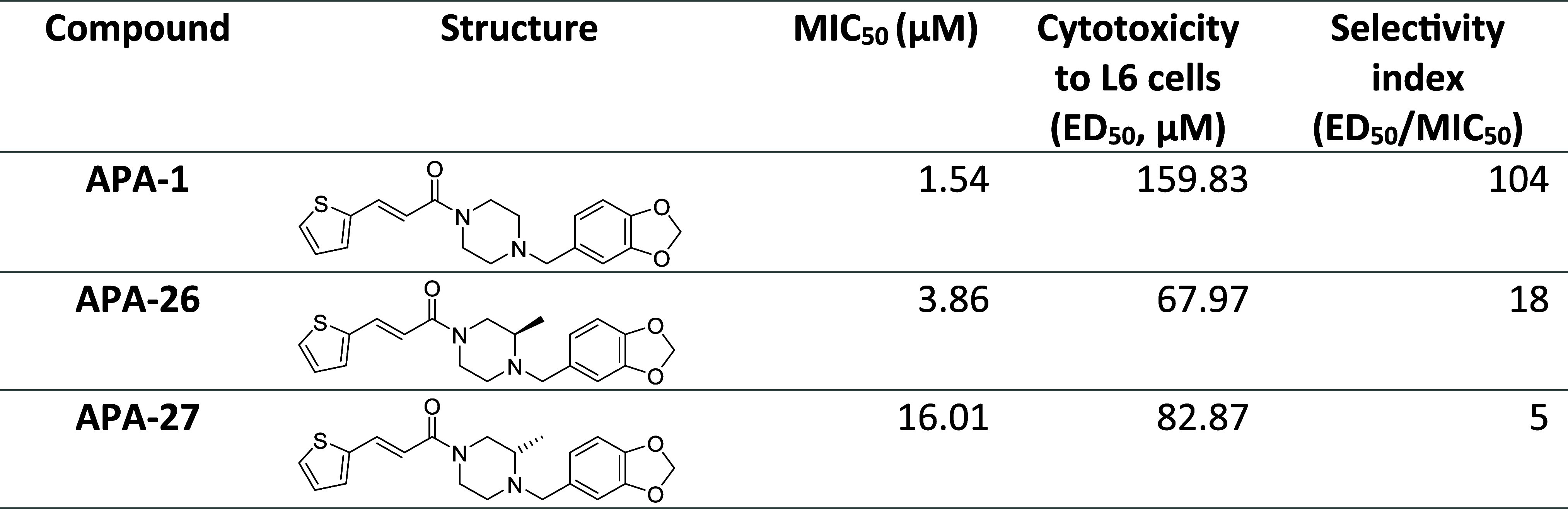
Activity of APA Compounds with Changes to the Central Structures of **APA-1** ^a^

a MIC_50_ against the classical *M. ulcerans* strain S1013 was determined by resazurin microtiter assay at 1 week postexposure.

Activity of arylvinylpiperazine amides against classical (S1013) and ancestral (S1324) *M. ulcerans* strains was assessed using a resazurin-based time-kill assay (Table 4).

**Table 4:** Activity of Selected APAs against Classical and Ancestral Lineage *Mul* Strains

	MBC^a^ (μM)	
Compound	Classical	Ancestral
APA-1	2.55	>28.06
APA-2	22.25	>28.06
APA-5	3.80	>26.92
APA-6	>10	>10
APA-8	>27.14	>27.14
APA-15	3.15	>23.89
APA-16	>10	>10
APA-17	5.59	>26.99
APA-18	4.84	>30.63
APA-19	6.36	>25.74
APA-21	25.96	>29.20
APA-22	13.01	>26.99
APA-23	2.81	>26.01
APA-24	3.21	≈25.09
APA-25	2.62	≈24.24
APA-26	16.82	>26.99
APA-27	>26.99	>26.99

a MBC (MIC_99_) against both *M. ulcerans* strains was determined at 4 weeks postexposure.

While those APAs with the lowest MIC_50_ values (Table 1) were also found to exhibit the lowest MBC values against the classical S1013 strain, there is no linear correlation. Most notably, **APA-6**, whose MIC_50_ value is essentially identical to that of **APA-1**, exhibits a 4-fold higher MBC value than the latter. The reason for this discrepancy is unclear at this point. Importantly, all APAs tested were completely inactive against the ancestral lineage strain, with only **APA-24** and **APA-25** retaining activity at the highest test concentration.

In summary, we analyzed the importance of the thiophene and benzodioxole portions of the APAs and showed that while the latter could be considerably modified without adversely impacting activity against *Mul*, the thiophene displayed a much more rigid SAR, with only a few modifications here still retaining activity against *Mul*. Only methylthiazole and furan replacements of the thiophene retained anti-*Mul* activity, although this activity for the furan derivative was lost upon longer incubation with the bacteria (evidenced by the MBC results shown in Table 4). Changes to the benzodioxole, which improved lipophilicity, as seen for the alkoxybenzyl derivatives, appeared to also improve anti-*Mul* activity (possibly by improving penetration of the thick waxy cell wall and highly hydrophobic ECM); however, further work needs to be done to select lipophilic modifications to the benzodioxole that optimally balance anti-*Mul* activity and cytotoxicity.

### Aminoquinazolines

The lead compound in this class, **AQ-1** (GSK353069A), was identified in the same phenotypic high-throughput screen against *M. bovis* BCG as **APA-1** and, similarly, was also shown to be active against *Mtb* H37Rv with a MIC_95_ of 27 ng/mL.^14^ **AQ-1** was additionally shown to be selective against a panel of Gram-positive and Gram-negative bacteria. It showed low cytotoxicity to HepG2 cells (IC_50_ > 50 μM) but had low stability in mouse (*t*_1/2_ < 3 min) and human liver microsomes (*t*_1/2_ < 6.5 min).^14^ Lupien et al. subsequently characterized the 4-aminoquinazoline derivatives **AQ-2, AQ-3,** and **AQ-4**, which all inhibit *Mtb* QcrB,^15^ but no data was reported for their activity against *Mul*.

In the present study, we have characterized the activity of **AQ-1** against a classical lineage *Mul* strain and determined an MBC of 100 ng/mL by CFU enumeration and of 89 ng/mL in the resazurin assay following 4 weeks of exposure (Figure 4).

**Figure 4:**
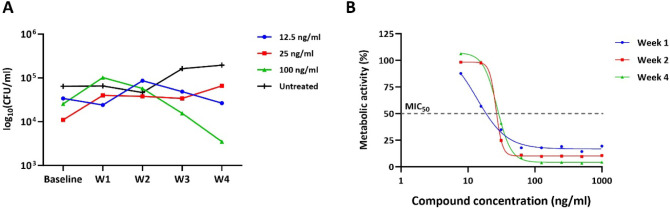
Time-kill assays for **AQ-1**. Activity against the classical lineage *M. ulcerans* strain S1013 was determined by CFU enumeration (A) and by resazurin reduction assay, in which the bacteria were incubated for 1, 2, or 4 weeks prior to 72 h of incubation with resazurin (B).

High-resolution cryo-EM structures of the *Mtb* cyt-*bc*_1_ *:aa*_3_ complexed with telacebec^16,17^ have shown that telacebec binds to the menaquinol binding site of the QcrB subunit of the *Mtb* cyt-*bc*_1_ *:aa*_3_ supercomplex. As the QcrB subunit of the mycobacterial cyt-*bc*_1_ *:aa*_3_ is highly conserved, these structures can also be used for the prediction of *Mul* QcrB binding. *In silico* docking analysis showed interactions between **AQ-1** and the QcrB complex similar to those of telacebec, with one nitrogen of **AQ-1** binding to the Glu314-His317 motif, the phenyl ring binding in the deep lipophilic pocket, and the S-methyl substituent overlaying with the ethyl group of telacebec (Figure 5). On the basis of these structural data and the previous data from Lupien et al.,^15^ we reevaluated the quinazolines as potential drug candidates against *Mul*, using a target-based optimization approach.

**Figure 5:**
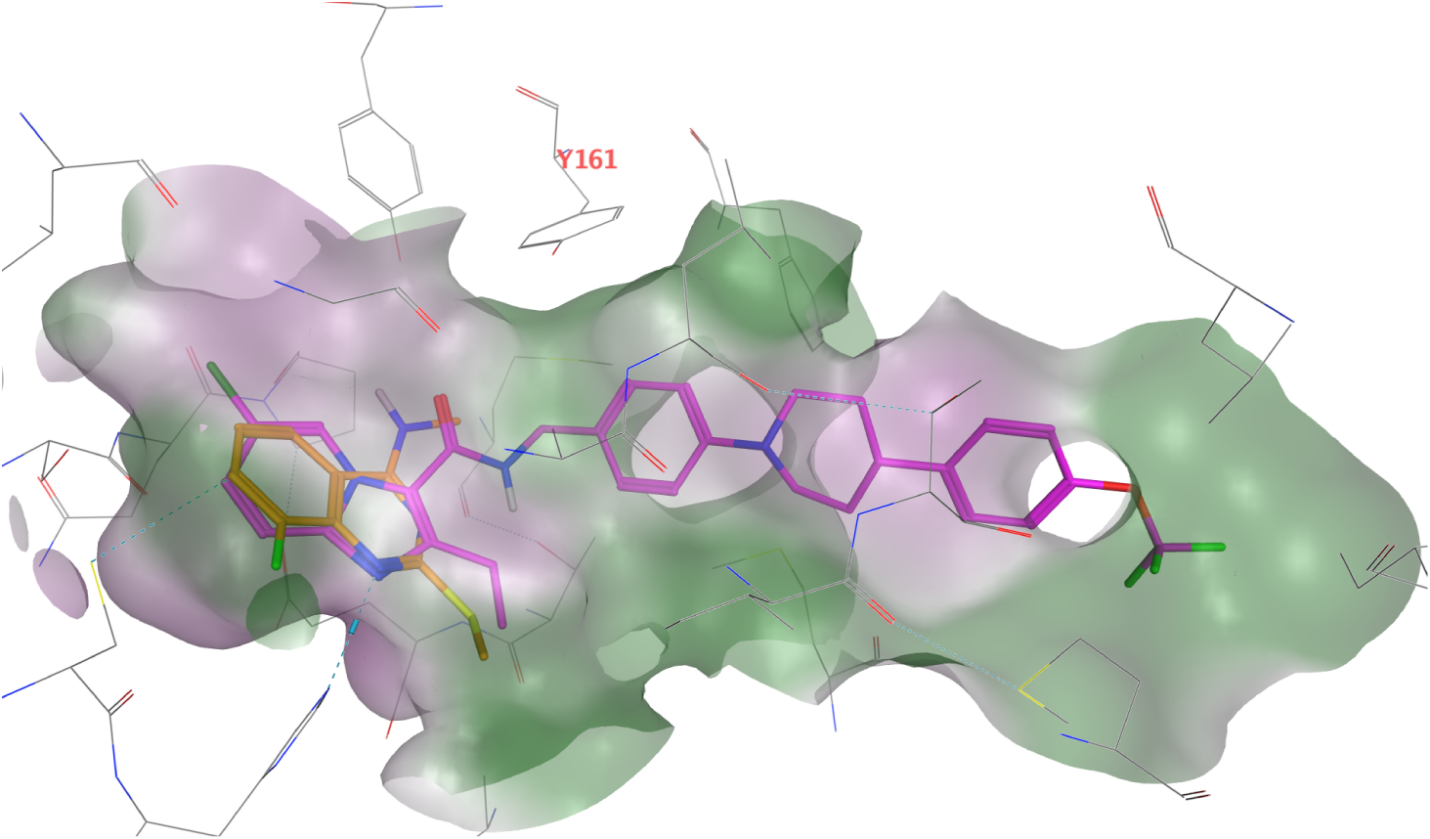
Superposition of **AQ-1** (orange) on telacebec (pink) in *Mtb* QcrB (docked in pdb: 7E1W), showing the CH-π interaction of **AQ-1** to Y161 (*Mtb*-numbering).

**AQ-1** showed similar activity against *Mul* (MIC_50_ 0.08 μM, or 0.02 μg/mL) as that previously reported against *Mtb* (MIC_95_ 0.13 μM or 0.03 μg/mL),^14^ and it had an excellent selectivity index of 3489 (Table 5).

**Table 5:**
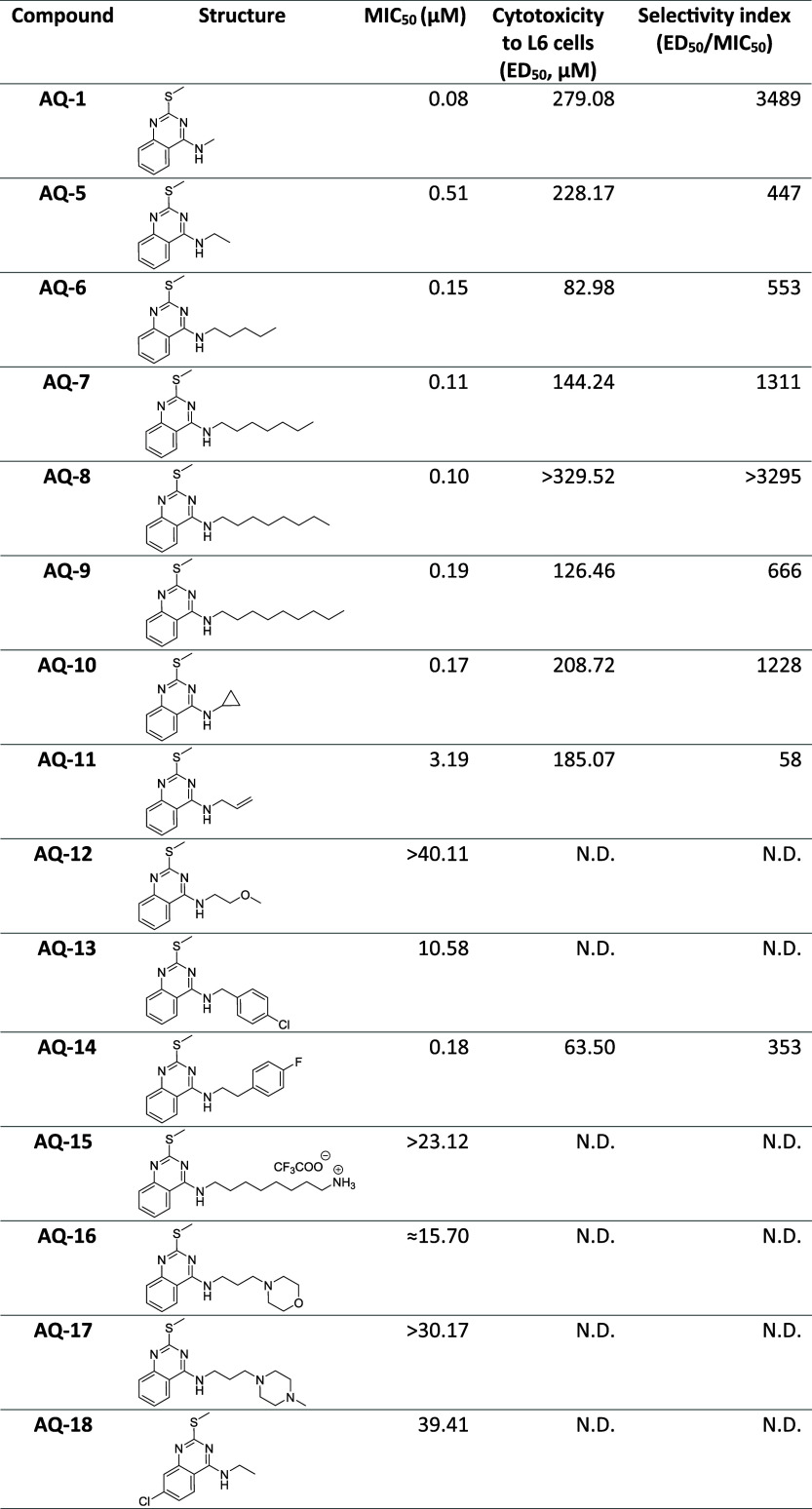
Activity of **AQ-1** Analogs with Varying N-Substituents^a^,^b^

a MIC_50_ against the classical *M. ulcerans* strain S1013 was determined at 1 week postexposure.

b N.D. = Not determined.

The main difference between the (predicted) binding of **AQ-1** and telacebec is the large lipophilic chain of the latter, filling the pocket that typically binds the highly lipophilic terpene chain of menaquinol. Even though the N-ethyl group of **AQ-1** exploits the same part of the binding site as the substituted benzylamide moiety of telacebec, the chains are connected to the bicyclic core in a different orientation, resulting in a different structure–activity relationship (SAR). Therefore, we systematically fine-tuned this chain for the aminoquinazolines.

The part of the menaquinol binding site that accommodates the terpene side chain exclusively comprises lipophilic amino acid residues. Therefore, in a first series of analogs of **AQ-1**, we evaluated the effect of varying the length of the aliphatic chain attached to the exocyclic amino group (Table 5). Additionally, as *Mul* produces an abundant, highly hydrophobic ECM, which combined with the waxy mycobacterial cell wall constitutes a formidable barrier to hydrophilic substances, we hypothesized that such lipophilic substituents would improve penetration of the derivatives, potentially leading to improved activity. Surprisingly, *N*-alkyl modifications of **AQ-1** gave no strong differentiation in MIC_50_ values (**AQ-1**: 0.078 μM; **AQ-5**: 0.511 μM; **AQ-6**: 0.153 μM; **AQ-7**: 0.107 μM; **AQ-8**: 0.099 μM; **AQ-9**: 0.188 μM). **AQ-10** with an N-*c*Pr modification still showed good activity (0.173 μM), although the N-allyl-modified **AQ-11** was 40-fold less active than **AQ-1**; the *N*-methoxyethyl (**AQ-12**) was inactive; and the N-4-Cl-benzyl (**AQ-13**) was >100-fold less active than **AQ-1**. The N-4-F-phenylethyl derivative **AQ-14** again showed strong activity (0.179 μM), whereas introducing basic amines in the side chain (**AQ-15, AQ-16, AQ-17**) resulted in loss of activity.

Although Lupien et al.^15^ have reported potent anti-*Mtb* activity of **AQ-2** and related N-substituted 2-ethylsulfanyl-4-aminoquinazolines, we found that S-ethyl AQ derivatives were comparatively less active than their S-methyl counterparts against *Mul* (Table 6). Indeed, MIC_50_ values of the S-ethyl AQs **AQ-2** (0.173 μM) and **AQ-3** (1.380 μM) were up to 3-fold higher than those of their corresponding S-methyl congeners **AQ-1** (0.078 μM) and **AQ-5** (0.511 μM), respectively. The S-ethyl derivatives also had lower selectivity indices than their S-methyl counterparts. Increasing the size of the S-substituent beyond an ethyl group was detrimental to activity; consequently, **AQ-19** to **AQ-24** were all inactive against *Mul* (Table 6).

**Table 6:**
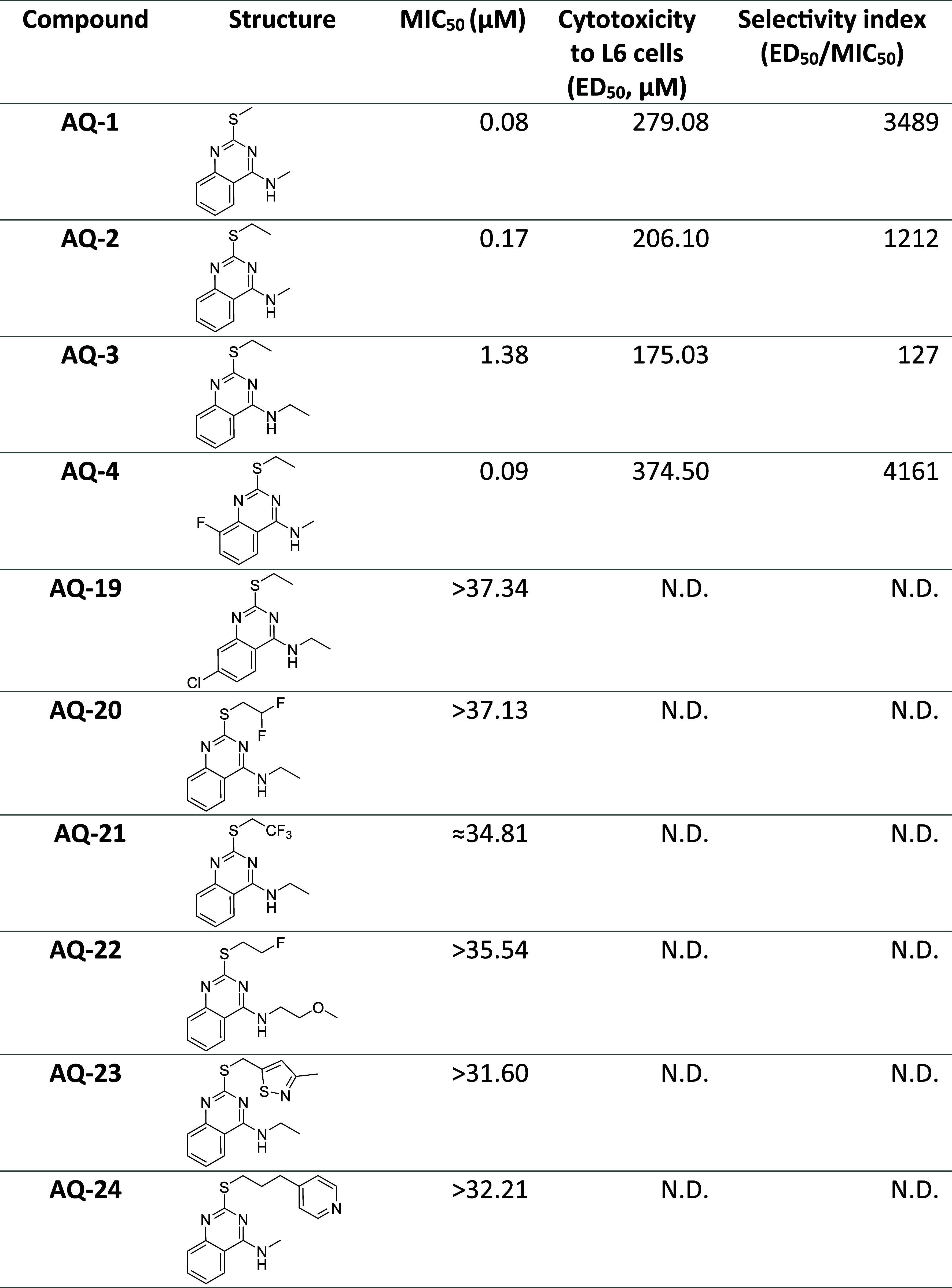
Activity of **AQ-1** Analogs Incorporating a 2-Ethylsulfanyl Group^a^,^b^

a MIC_50_ against the classical *M. ulcerans* strain S1013 was determined at 1 week postexposure.

b N.D. = Not determined.

Lupien et al.^15^ also reported that fluorination of the aminoquinazoline core, in particular the incorporation of fluorine at the 8-position, improved stability in human liver microsomes without negatively affecting activity against *Mtb*. Fluorination of **AQ-1** to give **AQ-25** did not significantly impact potency against *Mul* or selectivity (Table 7), while fluorinating **AQ-2** to give **AQ-4** improved the selectivity (Table 6), similar to what has been reported for *Mtb*.^15^

**Table 7:**
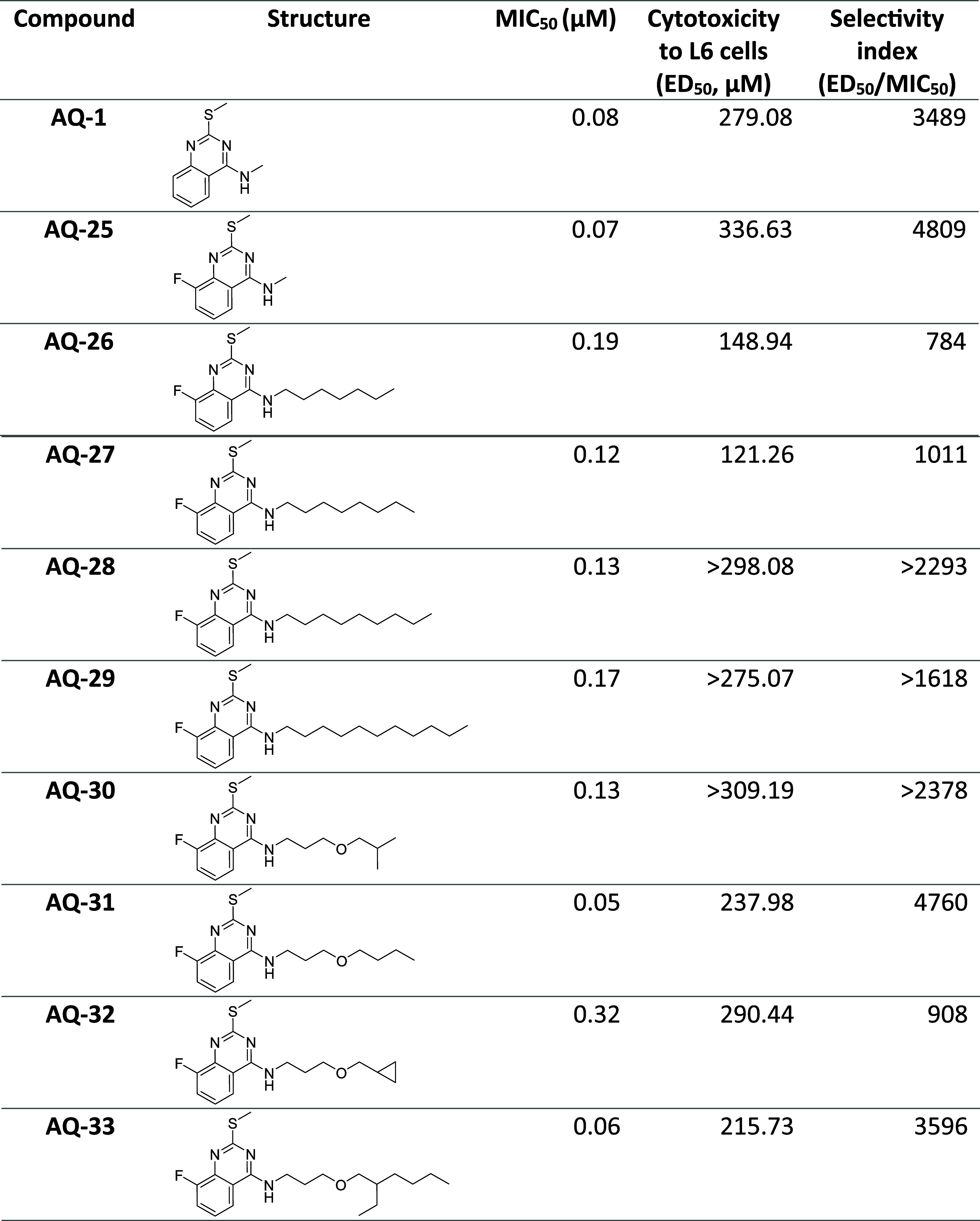
Activity of 8-Fluorinated Analogs of **AQ-1** Compounds with 4-Amino Modifications^a^

a MIC_50_ against the classical *M. ulcerans* strain S1013 was determined at 1 week postexposure.

As had been observed for **AQ-1**, increasing the length of the *N*-alkyl substituent in **AQ-25** likewise gave no strong differentiation in MIC_50_ values (**AQ-25**: 0.072 μM; **AQ-26**: 0.192 μM; **AQ-27**: 0.115 μM; **AQ-28**: 0.131 μM; **AQ-29**: 0.168 μM). Independent of the length of the *N*-alkyl chain, no differences in MIC_50_ values were observed between 8-fluorinated derivatives and the corresponding nonfluorinated parent compounds. In an attempt to polarize the CH-π interactions between Tyr161 of QcrB and the C3 and C5 methylene groups of the *N*-alkyl chain, we also investigated 8-fluorinated analogs with N-alkoxyalkyl substituents incorporating oxygen in place of C4 (**AQ-30** to **AQ-33**). This change appeared favorable as N-alkoxyalkyl derivatives largely had low MIC_50_ values and superb selectivity. For example, **AQ-31** had similar MIC_50_ and SI as **AQ-1** and **AQ-25** (Table 7).

Replacements of the quinazoline core were also explored (Table 8). Replacing the phenyl ring of **AQ-1** with a thiophene moiety (**AQ-34**) resulted in a significant drop of activity (MIC_50_ 1.396 μM), with the S-allyl derivative (**AQ-35**) being completely inactive (MIC_50_ > 42 μM). There was activity when the phenyl ring was replaced with a tetrahydrothiopyran residue (**AQ-36** to **AQ-38**). However, tetrahydrobenzothiophene­[2,3-*d*]­pyrimidine replacements (**AQ-39** to **AQ-41**) as well as complete removal of the phenyl ring with various *N*-alkyl substitutions (pyrimidines **AQ-42** to **AQ-53**) all led to complete loss of activity against *Mul* (Table 8).

**Table 8:**
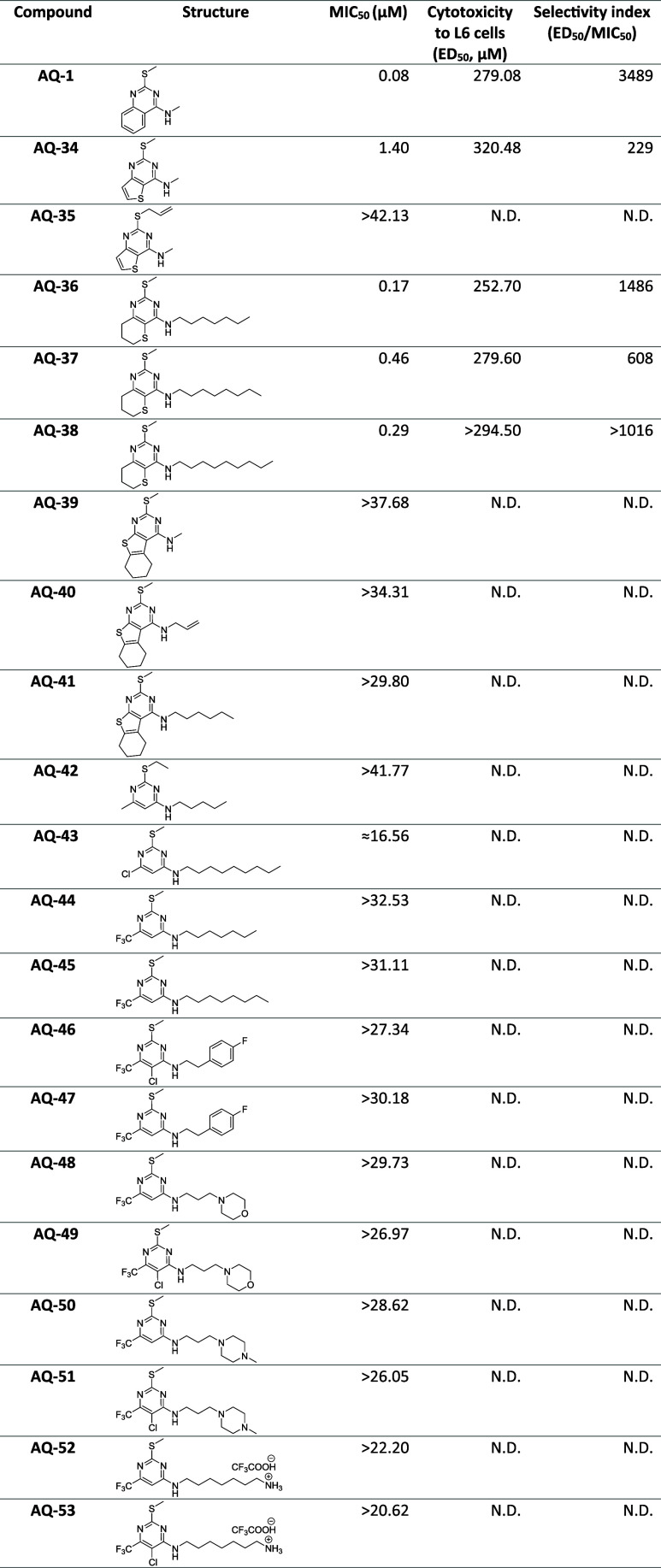
Activity of **AQ-1** Analogs with Phenyl Replacements^a^,^b^

a MIC_50_ against the classical *M. ulcerans* strain S1013 was determined at 1 week postexposure.

b N.D. = Not determined.

As for the APA compounds, we also assessed the MBC of selected AQ compounds against classical (S1013) and ancestral (S1324) lineage *M. ulcerans* strains using the resazurin-based time-kill assay. None of the quinazoline amines were active against the ancestral lineage strain S1324 at any of the time points tested, while several were active against the classical lineage strain with MBCs below 1 μM. Among these, the 8-fluorinated derivative **AQ-25** had a slightly lower MBC than the lead compound **AQ-1** (Table 9).

**Table 9:** Activity of Selected AQ Compounds against Classical and Ancestral Lineage *Mul* Strains

	MBC^a^ (μM)	
Compound	Classical	Ancestral
AQ-1	0.44	>4.87
AQ-2	1.05	>4.56
AQ-3	>4.29	>4.29
AQ-4	0.76	>4.21
AQ-5	>4.56	>4.56
AQ-6	0.61	>3.83
AQ-7	2.90	>3.45
AQ-8	2.93	>3.30
AQ-9	0.54	>3.15
AQ-10	0.86	>4.32
AQ-11	>4.32	>4.32
AQ-14	0.51	>3.19
AQ-25	0.36	>4.48
AQ-26	>3.25	>3.25
AQ-27	1.65	>3.11
AQ-28	1.31	>2.98
AQ-29	0.47	>2.75
AQ-30	0.62	>3.09
AQ-31	0.93	>3.09
AQ-32	>3.11	>3.11
AQ-33	0.82	>2.63
AQ-34	>4.73	>4.73
AQ-36	0.51	>3.21
AQ-37	>3.07	>3.07
AQ-38	1.97	>2.94

a MBC (MIC_99_) against both *M. ulcerans* strains was determined at 4 weeks postexposure.

Again, this is consistent with the reported targets of aminoquinazolines in mycobacteria. There were no general SAR trends in the MBCs, and there was no obvious correlation between MIC_50_s and MBCs, with some AQs with low MIC_50_s exhibiting significantly higher MBCs. This may, however, be due to some metabolic effects, as the MBC was evaluated after 4 weeks of exposure, whereas the MIC_50_ was measured after 1 week.

We employed a target-based optimization strategy for the aminoquinazolines, using the crystal structure published for telacebec in the menaquinol binding pocket of the mycobacterial QcrB (pdb: 7E1W). Overall, we observed that, unlike as was previously reported for *Mtb*,^15^ S-methyl modifications had a largely negative impact on the activity of AQs against *Mul*. N-modifications were, however, generally well-tolerated. Although we had hypothesized that long-chain *N*-alkyl residues would improve the anti-*Mul* activity of the compounds by increasing lipophilicity (and potentially improving penetration into the bacteria), we observed no strong differentiation of activity with increasing length of this side chain, both with the nonfluorinated and 8-fluorinated AQ derivatives. However, we observed that the introduction of oxygen in the alkyl chain and thereby polarization of CH_2_ groups potentially interacting with the Y161 residue of *Mul*-QcrB resulted in improved activity in the range of AQ1. We found the quinazoline core to be important for activity, as replacement of the annulated phenyl ring of this core with a bioisosteric thiophene caused a drop in activity, and complete removal of this phenyl led to loss of activity, although a thiane replacement still retained some activity.

### Chemistry

The synthesis of **APA-1–4** and **APA-7** has been described in ref 13. In analogy to these previous syntheses, **APA-5**, **APA-6**, and **APA-8**–**13** were prepared from phosphonate **I-1** ^13^ by HWE reaction with the appropriate aldehydes in yields between 65% and 99% (Scheme 1). Reduced analog **APA-14** was obtained via EDC/HOBt-mediated coupling of 3-(2-thienyl)­propanoic acid (**I-2**) and 1-piperonylpiperazine (**I-3**) in 92% yield (Scheme 1).

**Scheme 1:**
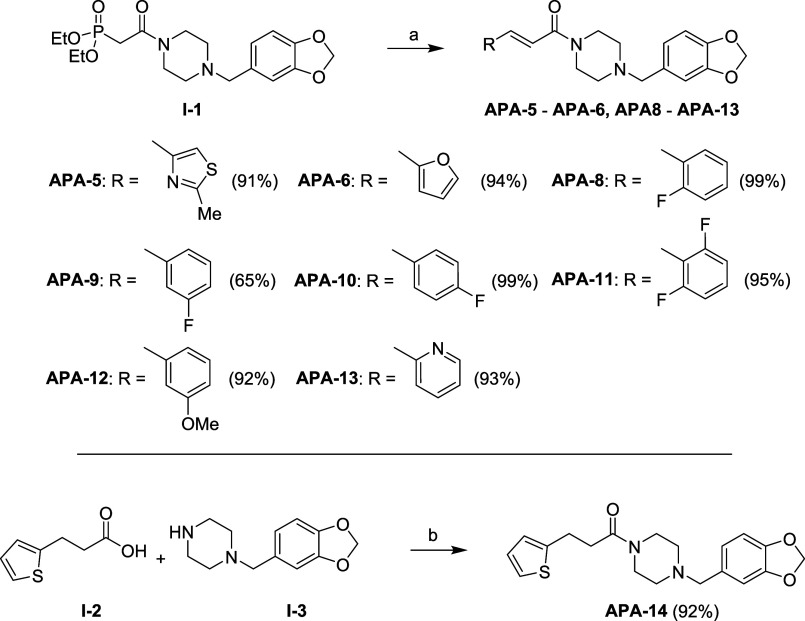
Synthesis of **APA-5**–**APA-6** and **APA-8**–**APA-13** ^a^

a Reagents and conditions: (a) NaH/R-CHO, THF, rt; (b) EDC, HOBt, Et_3_N, CH_2_Cl_2_, 80 °C, MW, 3 h.

Arylvinylpiperazinyl amide **APA-15** was prepared from commercial *N*-benzyl piperazine (**I-4**) via EDC/HOBt-mediated acylation with diethylphosphonoacetic acid followed by HWE reaction of the resulting phosphonoamide with thiophene-2-carbaldehyde (**I-5**) and NaH in 90% overall yield (Scheme 2). The synthesis of **APA-16** and **APA-18**–**21** proceeded through amine **I-9** as a common intermediate, which was converted into the desired arylvinylpiperazinyl amides either by alkylation with the corresponding benzyl bromides and NaH or by reductive alkylation with the appropriate aldehyde and NaBH­(OAc)_3_ in yields between 65% and 99% (Scheme 2). The common intermediate **I-9** was obtained from commercial *N*-Boc-piperazine (**I-6**) via EDC/HOBt-mediated acylation with diethylphosphonoacetic acid, followed by HWE reaction with thiophene-2-carbaldehyde (**I-5**) and NaH and Boc-cleavage with HCl/dioxane in 88% overall yield. To access **APA-22**–**25**, **APA-20** was further processed by alkylation with a series of alkyl bromides; the yields of these reactions were between 73% and 84% (Scheme 2).

**Scheme 2:**
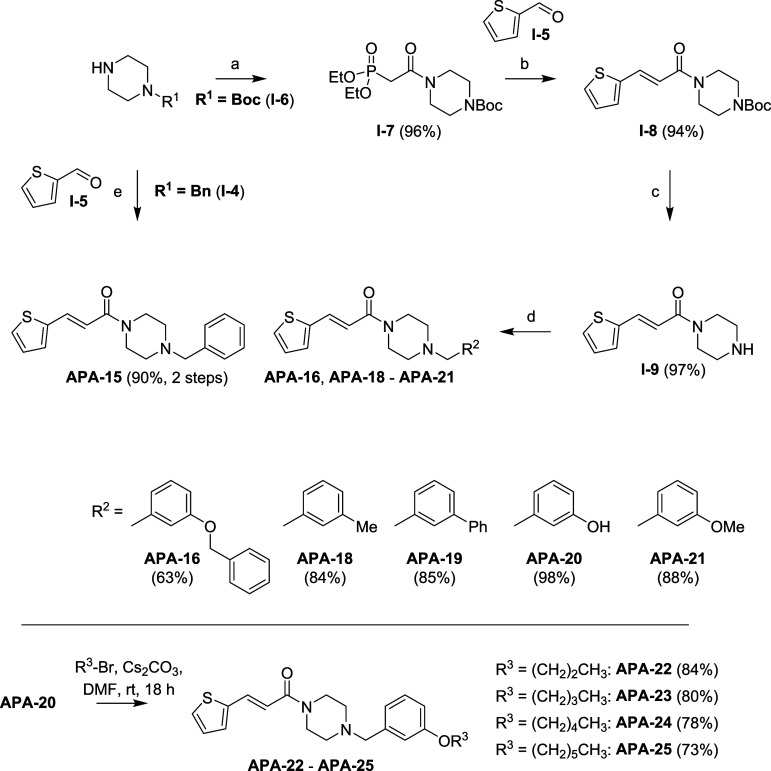
Synthesis of **APA-15**–**APA-16** and **APA-18**–**APA-25** ^a^

a Reagents and conditions: (a) (EtO)_2_P­(O)­CH_2_COOH, EDC, HOBt, Et_3_N, CH_2_Cl_2_, 80 °C, MW, 3 h; (b) **I-5**, NaH, THF, rt, 18 h; (c) 4 M HCl/dioxane, rt, 20 min; (d) R^2^CH_2_Br, NaH, THF (**APA-16**, **APA-18**, **APA-19**) or R^2^CHO, NaBH­(OAc)_3_, CH_2_Cl_2_, 0 °C to rt, 18 h (**APA-20**, **APA-21**); (e) 1. (EtO)_2_P­(O)­CH_2_COOH, EDC, HOBt, Et_3_N, CH_2_Cl_2_, 80 °C, MW, 3; 2. **I-5**, NaH, THF, rt, 18 h.

As depicted in Scheme 3, the synthesis of **APA-17** commenced with the reductive alkylation of *N*-Boc-piperazine (**I-6**) with 2,3-dihydrobenzo­[*b*]­[1,4]­dioxine-6-carbaldehyde (**I-10**) and NaBH­(OAc)_3_ to provide **I-11** in 99% yield. The latter was converted into phosphonate **I-13** via Boc-removal with HCl/dioxane and subsequent acylation of the resulting free amine **I-12** with diethylphosphonoacetic acid in the presence of EDC/HOBt in 56% overall yield. Finally, the HWE reaction of **I-13** with thiophene-2-carbaldehyde and NaH gave **APA-17** in quantitative yield.

**Scheme 3:**
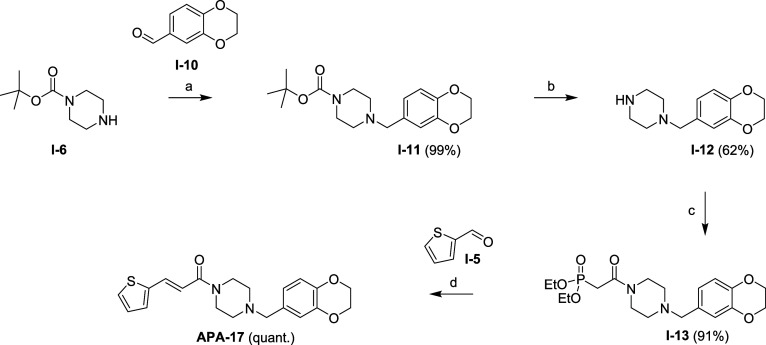
Synthesis of **APA-17** ^a^

a Reagents and conditions: (a) **I-10**, NaBH­(OAc)_3_, CH_2_Cl_2_, 0 °C to rt, 18 h; (b) 4 M HCl/dioxane, rt, 60 min; (c) (EtO)_2_P­(O)­CH_2_COOH, EDC, HOBt, Et_3_N, CH_2_Cl_2_, 80 °C, MW, 3 h; (d) **I-5**, NaH, THF, rt, 18 h.

The synthesis of the two enantiomeric arylvinylpiperazinyl amides, **APA-26** and **APA-27**, is exemplified in Scheme 4 for the (*R*)-isomer **APA-26**. Acylation of commercial *tert*-butyl (*R*)-2-methylpiperazine-1-carboxylate (**I-14**) with diethylphosphonoacetic acid and EDC/HOBt gave phosphonate **I-15** in quantitative yield. The HWE reaction of the latter with thiophene-2-carbaldehyde (**I-5**) and NaH, followed by Boc-cleavage with HCl/dioxane, furnished amine **I-17** in 92% overall yield. Finally, reductive amination of **I-17** with piperonal (**I-3**) and NaBH­(OAc)_3_ gave **APA-26**. The (*S*)-enantiomer **APA-27** was obtained via the same route, starting from commercial *tert*-butyl (*S*)-2-methylpiperazine-1-carboxylate (**I-18**) in 45% overall yield.

**Scheme 4:**
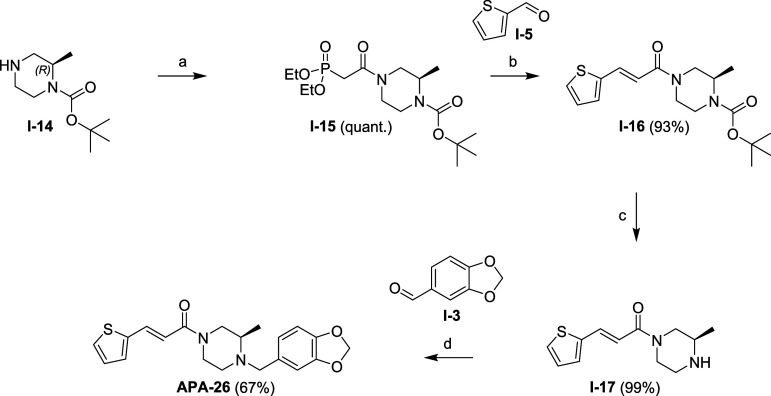
Synthesis of **APA-26** ^a^

a Reagents and conditions: (a) (EtO)_2_P­(O)­CH_2_COOH, EDC, HOBt, Et_3_N, CH_2_Cl_2_, 80 °C, MW, 3 h; (b) **I-5**, NaH, THF, rt, 18 h; (c) 4 M HCl/dioxane, rt, 20 min; (d) **I-3**, NaBH­(OAc)_3_, CH_2_Cl_2_, 0 °C to rt, 18 h.

The synthesis of the tested aminoquinazolines was done by reacting the commercial or predescribed 4-chloro-2-methylsulfanyl-quinazolines^15^ with the corresponding alkylamine (Scheme 5).

**Scheme 5:**
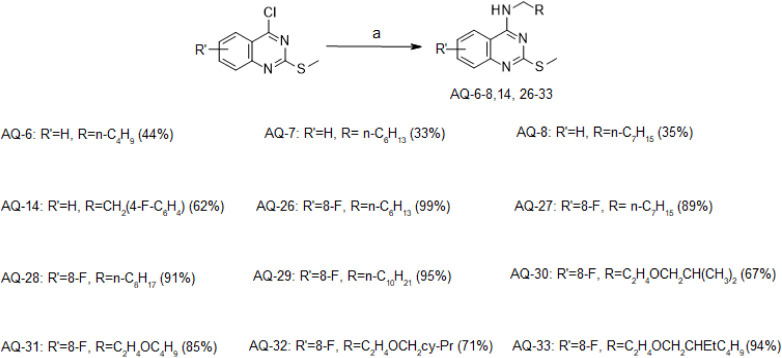
Synthesis of **AQ-6**–**AQ-8**, **AQ-14**, and **AQ-26**–**AQ-33** ^a^

a Reagents and conditions: (a) RCH_2_NH_2_, K_2_CO_3_, THF, 40 °C.

The yields of these reactions were between 33% and 99%. **AQ-1, -2, -3, -5, -10, -11, -12, -13, -34**, and **-35** were commercially available and used after purity confirmation. **AQ-4** was prepared by the described procedure.^15^

## Conclusion

In the current study, we have profiled the arylvinylpiperazine amide **APA-1** and the aminoquinazoline **AQ-1** against *M. ulcerans*, and we have investigated the anti-*Mul* activity of a series of analogs of both lead compounds. Both **APA-1** and **AQ-1** were previously shown to inhibit the QcrB subunit of the mycobacterial cyt-*bc*_1_ *:aa*_3_. For both classes of compounds, modifications to the lead structure could be identified that led to improved activity against classical lineage *Mul* strains without compromising selectivity over mammalian cells. Consistent with their mode of action, even compounds with low MIC_50_ values against classical strains proved to be inactive (AQs) or substantially less active (APAs) against the ancestral lineage *Mul* strains. As the latter strains harbor a functional secondary cyt-*bd* that enables them to bypass the effect of cyt-*bc*_1_ *:aa*_3_ blockade, they were practically insensitive to most of the APA and all of the AQ compounds tested.

More specifically, APAs were less tolerant to changes in the thiophene moiety compared to the 1,3-benzodioxole system, with most of the modifications of the thiophene ring resulting in reduction or abolition of activity against *Mul*. The most potent compounds were **APA-23**, **APA-24**, and **APA-25**, which incorporate an alkoxyphenyl ring in place of the 1,3-benzodioxole moiety and whose anti-*Mul* activity exceeds that of **APA-1**. Activity within this group increased with increasing length of the alkoxy substituent (pentoxy > butoxy > propoxy). There was, however, a concomitant increase in cytotoxicity.

Within the AQ series, N-modifications were much better tolerated than S-modifications, and we also showed that the quinazoline core was required for activity against *Mul*, as replacing the phenyl of the quinazoline core or completely removing it largely resulted in loss of activity. Overall, **AQ-25** proved to be the most interesting derivative, showing the best MIC_50_ (0.072 μM) and MIC_99_ (0.358 μM) against *Mul*, with low cytotoxicity (ED_50_ 336.63 μM) and an excellent selectivity index (SI = 4809). Considering the previously described^15^ favorable effect of 8-F substitution of **AQ-1** on pharmacokinetic properties, **AQ-25** is an interesting candidate for further evaluation against *Mul*.

## Experimental Section

### Bacteria and Media

Low-passage *M. ulcerans* strains isolated from BU lesions of patients in Africa and Japan were used for the analyses. The Cameroonian strain S1013, isolated in 2010 from an ulcerative BU lesion,^18^ represented the classical *M. ulcerans* lineage, while the Japanese strain S1324 (corresponding to the ATCC E10013 strain) represented the ancestral lineage. Both bacterial strains were cultivated at 30 °C in Middlebrook 7H9 medium, supplemented with 1% glycerol, 0.1% casein, and 10% OADC (oleic acid, albumin, dextrose, catalase).

For compound screening, Middlebrook 7H9 medium supplemented with 0.2% glycerol and 10% OADC was used. This was agar solidified for time-kill assays.

Cytotoxicity to cultured mammalian cells was assessed using RPMI medium supplemented with 10% heat-inactivated fetal bovine serum and 1.7 μM L-glutamine.

### MIC_50_ Determination

MIC_50_ determination was done using a resazurin microtiter assay as previously described.^19^ Briefly, *M. ulcerans* cultures at an initial optical density at 600 nm (OD_600_) of 0.04 were incubated with a 2-fold dilution series of test compounds at 30 °C for 8 days. Thereafter, 10% v/v of a 0.125 mg/mL resazurin solution was added, and cultures were incubated for an additional 3 days, after which the fluorescence was measured (Ex_540 nm_/Em_588 nm_). The metabolic activity was determined relative to the included *M. ulcerans* drug-free control, and the MIC_50_ was defined as the concentration of test compound that reduced metabolic activity by 50%. This was done in duplicates, and the average MIC_50_ value was reported.

### Time-Kill Assays

The lead compound of each scaffold was assessed for microbicidal activity against *M. ulcerans*. Cultures were exposed to varying concentrations of test compounds for varying lengths of time (Baseline, Week 1, Week 2, Week 3, Week 4). At each time point, 10-fold serial dilutions were plated out on Middlebrook 7H9 agar medium supplemented with 0.2% glycerol and 10% OADC, and incubated at 30 °C for up to six months. CFUs were counted monthly, and the final count was done at the end of the experiment. The minimum bactericidal concentration (MBC) was defined as the concentration that led to a 3 log_10_ decrease in CFUs relative to the included drug-free control.

To improve the throughput, we analyzed all active compounds (MIC_50_ ≤ 5 μg/mL) using a resazurin-based kinetic assay, which we had previously shown to give comparable results to CFU enumeration.^20^ Here, cultures at an initial optical density at 600 nm (OD_600_) of 0.04 were incubated at 30 °C with a dose range of test compounds for 1 week, 2 weeks, or 4 weeks. At each time point, 10% v/v of a 0.125 mg/mL resazurin solution was added, and then cultures were incubated for an additional 3 days, after which the fluorescence was measured (Ex_540 nm_/Em_588 nm_). The metabolic activity was determined relative to the included *M. ulcerans* drug-free control. The MBC, defined as the concentration that led to ≥99% reduction in metabolic activity, was extrapolated from the dose–response curves using GraphPad Prism (version 8.2.1).

### Cytotoxicity Assays

Wells of 96-well plates were seeded with rat L6 skeletal myoblast cells at a density of 2 × 10^4^ cells/well and incubated overnight at 37 °C in 5% CO_2_. On the following day, 3-fold dilution series of test compounds (highest concentration 100 μg/mL or 100 μM, depending on the test compound stock concentration) were added, and the plates were incubated for a further 69 h. Subsequently, 10% of a 0.125 mg/mL resazurin assay was added and incubated for 3 h, after which fluorescence was measured. Metabolic activity relative to unexposed cells was calculated, and the ED_50_ was defined as the concentration of test compound that reduced metabolic activity by 50%.

### Chemistry

#### Synthesis of Arylvinylpiperazine Amides

##### General Information

All nonaqueous reactions were performed under an argon atmosphere using flame-dried glassware and standard syringe/septa techniques. CH_2_Cl_2_ and THF used for reactions were distilled under argon prior to use (CH_2_Cl_2_ from CaH_2_, THF from Na/benzophenone). All other solvents used for reactions were purchased as anhydrous grade from ACROS (puriss.; dried over molecular sieves; H_2_O < 0.005%) and used without purification. Solvents for extractions, flash column chromatography, and thin-layer chromatography were purchased as commercial grade and distilled prior to use. All other commercially available reagents were used without further purification. In general, reactions were magnetically stirred and monitored by thin-layer chromatography (**TLC**) performed on MERCK **TLC** aluminum sheets (silica gel 60 F254). Spots were visualized with UV light (λ = 254 nm) or through staining with Ce_2_(SO_4_)_3_/phosphomolybdic acid/H_2_SO_4_ (CPS) or KMnO_4_/K_2_CO_3_. Purification of products by flash column chromatography was performed using Fluka silica gel 60 (particle size 40–63 μm).

**Melting points** were obtained in open capillary tubes using a BÜCHI melting point apparatus B-540 and are uncorrected.

^**1**^ **H and** ^13^C NMR **spectra** were recorded in CDCl_3_ or CD_3_OD on a BRUKER AV-400 400 MHz spectrometer at room temperature. Chemical shifts (δ) are reported in ppm and are referenced to chloroform (δ 7.26 ppm for 1H, δ 77.16 ppm for 13C) and methanol (δ 3.31 ppm for 1H, δ 49.00 ppm for 13C), respectively. All ^13^C NMR spectra were measured with complete proton decoupling. Data for NMR spectra are reported as follows: s = singlet, d = doublet, t = triplet, q = quartet, quint. = quintet, sext. = sextet, hept. = heptet, m = multiplet, bs = broad singlet, *J =* coupling constant in Hz. The multiplicity of signals is reported based on appearance (i.e., doublet of doublets that are apparent triplets are described as triplets).

**Infrared spectra (IR)** were recorded on a JASCO FT/IR-6200 instrument. Resonance frequencies are given as wavenumbers in cm^–1^.

**Optical rotations** were measured on a JASCO P-1020 polarimeter or an ANTON-PAAR MCP 300 with a 10 or 100 mm path length cell and are reported as follows: [α]­D20 (concentration (g/100 mL), solvent).

**High-resolution mass spectra (HR-MS)** were recorded on a BRUKER maXis (ESI) or on a WATERS AutoSpec Ultima spectrometer (EI), respectively, by the ETH Zürich MS service.

(*E*)-1-(4-(benzo­[d]­[1,3]­dioxol-5-ylmethyl)­piperazin-1-yl)-3-(2-methylthiazol-4-yl)­prop-2-en-1-one (**APA-5**). To a solution of diethyl (2-(4-(benzo­[d]­[1,3]­dioxol-5-ylmethyl)­piperazin-1-yl)-2-oxoethyl)­phosphonate (**I-1**)^13^ (25.0 mg, 62.8 μmol, 1.00 equiv) in THF (0.8 mL) were added NaH (1.81 mg, 75.3 μmol, 1.20 equiv) and 2-methylthiazole-4-carbaldehyde (9.58 mg, 75.3 μmol, 1.20 equiv). The reaction mixture was stirred at rt for 48 h, filtered through Celite, and concentrated *in vacuo*. Preparative **TLC** (CH_2_Cl_2_/MeOH 95:5) provided **APA-5** (21.9 mg, 59.0 μmol, 94%) as a white solid. **TLC** (SiO_2_; CH_2_Cl_2_/MeOH 9:1, UV): R_f_ = 0.63. **Mp**: 114.1–115.0 °C. ^**1**^ **H NMR** (400 MHz, CDCl_3_): δ (ppm) = 7.55 (d, *J =* 14.8 Hz, 1H), 7.24–7.18 (m, 2H), 6.86 (s, 1H), 6.77–6.71 (m, 2H), 5.95 (s, 2H), 3.77–3.64 (m, 4H), 3.44 (s, 2H), 2.72 (s, 3H), 2.50–2.39 (m, 4H). ^13^C NMR (101 MHz, CDCl_3_): δ (ppm) = 166.9, 165.5, 152.3, 147.9, 146.9, 134.8, 131.6 (HMBC), 122.4, 120.8, 119.1, 109.6, 108.1, 101.1, 62.7, 53.4, 52.7, 45.9, 42.3, 19.5. **IR** (thin film): ν = 3460, 3076, 2898, 2810, 2773, 1646, 1605, 1501, 1488, 1439, 1368, 1332, 1299, 1250, 1239, 1214, 1187, 1144, 1129, 1115, 1096, 1038, 1000, 974, 932, 863, 810, 791, 753, 734, 701, 660, 603, 582, 526 cm^–1^. **HR-MS** (ESI): Calcd for C_19_H_22_N_3_O_3_S [M + H]^+^, 372.1376 *m*/*z*; Found, 372.1372 *m*/*z*.

(*E*)-1-(4-(Benzo­[d]­[1,3]­dioxol-5-ylmethyl)­piperazin-1-yl)-3-(furan-2-yl)­prop-2-en-1-one (**APA-6**). To a solution of diethyl (2-(4-(benzo­[d]­[1,3]­dioxol-5-ylmethyl)­piperazin-1-yl)-2-oxoethyl)­phosphonate (**I-1**) (43.9 mg, 110 μmol, 1.00 equiv) in THF (2.0 mL) were added NaH (3.17 mg, 132 μmol, 1.20 equiv) and furan-2-carbaldehyde (10.6 μL, 132 μmol, 1.20 equiv). The reaction mixture was stirred at rt for 18 h, filtered through Celite, and concentrated *in vacuo*. Preparative **TLC** (CH_2_Cl_2_/MeOH 98:2) provided **APA-6** (34.2 mg, 101 μmol, 91%) as an orange oil. **TLC** (SiO_2_; CH_2_Cl_2_/MeOH 98:2, UV): R_f_ = 0.21. ^**1**^ **H NMR** (400 MHz, CDCl_3_): δ (ppm) = 7.49–7.39 (m, 2H), 6.85 (s, 1H), 6.80–6.71 (m, 3H), 6.53 (d, *J* = 3.3 Hz, 1H), 6.44 (dd, *J* = 3.4, 1.8 Hz, 1H), 5.94 (s, 2H), 3.75–3.60 (m, 4H), 3.43 (s, 2H), 2.50–2.37 (m, 4H). ^13^C NMR (101 MHz, CDCl_3_): δ (ppm) = 165.2, 151.8, 147.8, 146.9, 143.9, 131.7, 129.7, 122.3, 114.7, 113.8, 112.3, 109.5, 108.0, 101.1, 62.7, 53.3, 52.7, 45.9, 45.9, 42.3. **IR** (thin film): ν = 3116, 2989, 2919, 2810, 2772, 1649, 1604, 1557, 1501, 1487, 1439, 1367, 1335, 1299, 1273, 1236, 1188, 1146, 1115, 1096, 1037, 1016, 1000, 968, 928, 883, 866, 847, 810, 791, 738, 727, 648, 594, 531 cm^–1^. **HR-MS** (ESI): Calcd for C_19_H_21_N_2_O_4_ [M + H]^+^, 341.1496 *m*/*z*; Found, 341.1492 *m*/*z*.

(*E*)-1-(4-(Benzo­[d]­[1,3]­dioxol-5-ylmethyl)­piperazin-1-yl)-3-(2-fluorophenyl)­prop-2-en-1-one (**APA-8**). To a solution of diethyl (2-(4-(benzo­[d]­[1,3]­dioxol-5-ylmethyl)­piperazin-1-yl)-2-oxoethyl)­phosphonate (**I-1**) (59.0 mg, 148 μmol, 1.00 equiv) in THF (1.6 mL) were added NaH (4.26 mg, 178 μmol, 1.20 equiv) and 2-fluorobenzaldehyde (18.7 μL, 178 μmol, 1.20 equiv). The reaction mixture was stirred at rt for 18 h, filtered through Celite, and concentrated *in vacuo*. Flash chromatography (CH_2_Cl_2_/MeOH 99:1 to 98:2) provided **APA-8** (54.0 mg, 147 μmol, 99%) as a yellow oil. **TLC** (SiO_2_; CH_2_Cl_2_/MeOH 95:5, UV): R_f_ = 0.32. ^**1**^ **H NMR** (400 MHz, CDCl_3_): δ (ppm) = 7.69 (d, *J* = 15.7 Hz, 1H), 7.51–7.44 (m, 1H), 7.33–7.27 (m, 1H), 7.16–7.09 (m, 1H), 7.06 (ddd, *J* = 11.0, 8.2, 1.2 Hz, 1H), 6.99 (d, *J* = 15.7 Hz, 1H), 6.85 (s, 1H), 6.77–6.69 (m, 2H), 5.93 (s, 2H), 3.80–3.54 (m, 4H), 3.43 (s, 2H), 2.51–2.37 (m, 4H). ^**13**^ **C NMR** (101 MHz, CDCl_3_): δ (ppm) = 165.4, 161.3 (d, ^1^ *J*C-F = 252.8 Hz), 147.8, 146.9, 135.8, 131.5, 130.9 (d, ^3^ *J*C-F = 9.9 Hz), 129.8 (d, ^3^ *J*C-F = 3.0 Hz), 124.4 (d, ^4^ *J*C-F = 3.3 Hz), 123.4 (d, ^3^ *J*C-F = 12.7 Hz), 122.3, 120.3 (d, ^4^ *J*C-F = 9.5 Hz), 116.2 (d, ^3^ *J*C-F = 22.9 Hz), 109.5, 108.0, 101.0, 62.6, 53.2, 52.6, 45.9, 42.3. ^**19**^ **F-NMR** (376 MHz, CDCl_3_): δ (ppm) = −114.1 (m). **IR** (thin film): ν = 2922, 2811, 2773, 1650, 1610, 1578, 1502, 1489, 1440, 1367, 1335, 1312, 1239, 1146, 1114, 1095, 1038, 1001, 980, 933, 866, 829, 809, 759, 579, 503, 468, 445, 428, 418 cm^–1^. **HR-MS** (ESI): Calcd for C_21_H_22_FN_2_O_3_ [M + H]^+^, 369.1609 *m*/*z*; Found, 369.1609 *m*/*z*.

(*E*)-1-(4-(Benzo­[d]­[1,3]­dioxol-5-ylmethyl)­piperazin-1-yl)-3-(3-fluorophenyl)­prop-2-en-1-one (**APA-9**). To a solution of diethyl (2-(4-(benzo­[d]­[1,3]­dioxol-5-ylmethyl)­piperazin-1-yl)-2-oxoethyl)­phosphonate (**I-1**) (29.3 mg, 73.5 μmol, 1.00 equiv) in THF (1.0 mL) were added NaH (2.12 mg, 88.3 μmol, 1.20 equiv) and 3-fluorobenzaldehyde (9.36 μL, 88.3 μmol, 1.20 equiv). The reaction mixture was stirred at rt for 18 h, filtered through Celite, and concentrated *in vacuo*. Preparative **TLC** (CH_2_Cl_2_/MeOH 98:2) provided **APA-9** (17.5 mg, 47.5 μmol, 65%) as a yellow oil. **TLC** (SiO_2_; CH_2_Cl_2_/MeOH 98:2, UV): R_f_ = 0.22. ^**1**^ **H NMR** (400 MHz, CDCl_3_): δ (ppm) = 7.61 (d, *J* = 15.4 Hz, 1H), 7.36–7.26 (m, 2H), 7.23–7.18 (m, 1H), 7.04 (tdd, *J* = 8.2, 2.6, 1.1 Hz, 1H), 6.86 (s, 1H), 6.85 (d, *J* = 15.4 Hz, 1H), 6.77–6.72 (m, 2H), 5.95 (s, 2H), 3.77–3.61 (m, 4H), 3.44 (s, 2H), 2.50–2.44 (m, 4H). ^**13**^ **C NMR** (101 MHz, CDCl_3_): δ (ppm) = 165.1, 163.1 (d, ^1^ *J*C-F = 246.4 Hz), 147.9, 146.9, 141.5, 137.7 (d, ^3^ *J*C-F = 8.7 Hz), 131.6, 130.5 (d, ^3^ *J*C-F = 8.9 Hz), 124.0, 122.4, 118.7, 116.5 (d, ^3^ *J*C-F = 21.0 Hz), 114.0 (d, ^3^ *J*C-F = 22.3 Hz), 109.5, 108.1, 101.1, 62.7, 53.3, 52.7, 46.0, 42.4. ^**19**^ **F-NMR** (376 MHz, CDCl_3_): δ (ppm) = −112.9 (m). **IR** (thin film): ν = 3064, 2921, 2854, 2811, 2773, 1650, 1606, 1583, 1502, 1488, 1441, 1367, 1335, 1303, 1238, 1146, 1115, 1096, 1038, 1000, 978, 933, 866, 848, 809, 786, 742, 716, 673, 566, 520, 448, 435, 417 cm^–1^. **HR-MS** (ESI): Calcd for C_21_H_22_FN_2_O_3_ [M + H]^+^, 369.1609 *m*/*z*; Found, 369.1602 *m*/*z*.

(*E*)-1-(4-(Benzo­[d]­[1,3]­dioxol-5-ylmethyl)­piperazin-1-yl)-3-(4-fluorophenyl)­prop-2-en-1-one (**APA-10**). To a solution of diethyl (2-(4-(benzo­[d]­[1,3]­dioxol-5-ylmethyl)­piperazin-1-yl)-2-oxoethyl)­phosphonate (**I-1**) (26.0 mg, 65.3 μmol, 1.00 equiv) in THF (0.8 mL) were added NaH (1.88 mg, 78.3 μmol, 1.20 equiv) and 4-fluorobenzaldehyde (8.27 μL, 78.3 μmol, 1.20 equiv). The reaction mixture was stirred at rt for 18 h, filtered through Celite, and concentrated *in vacuo*. Preparative **TLC** (CH_2_Cl_2_/MeOH 98:2) provided **APA-10** (23.9 mg, 64.9 μmol, 99%) as a white solid. **TLC** (SiO_2_; CH_2_Cl_2_/MeOH 95:5, UV): R_f_ = 0.20. Mp: 133.1–134.9 °C. ^**1**^ **H NMR** (400 MHz, CDCl_3_): δ (ppm) = 7.61 (d, *J* = 15.3 Hz, 1H), 7.50–7.44 (m, 2H), 7.07–7.00 (m, 2H), 6.84 (s, 1H), 6.78 (d, *J* = 15.4 Hz, 1H), 6.75–6.70 (m, 2H), 5.93 (s, 2H), 3.77–3.58 (m, 4H), 3.42 (s, 2H), 2.49–2.40 (m, 4H). ^**13**^ **C NMR** (101 MHz, CDCl_3_): δ (ppm) = 165.3, 163.5 (d, ^1^ *J*C-F = 250.5 Hz), 147.8, 146.9, 141.5, 131.6, 131.5, 129.6 (d, ^3^ *J*C-F = 8.9 Hz), 122.3, 117.0, 115.9 (d, ^3^ *J*C-F = 22.4 Hz), 109.5, 108.0, 101.0, 62.6, 53.2, 52.6, 45.9, 42.3. ^**19**^ **F-NMR** (376 MHz, CDCl_3_): δ (ppm) = −110.9 (m). **IR** (thin film): ν = 3001, 2898, 2811, 2773, 1649, 1600, 1508, 1489, 1439, 1367, 1335, 1299, 1275, 1222, 1159, 1146, 1115, 1096, 1038, 1001, 979, 933, 881, 865, 826, 792, 776, 751, 736, 639, 580, 532, 511 cm^–1^. **HR-MS** (ESI): Calcd for C_21_H_22_FN_2_O_3_ [M + H]^+^, 369.1609 *m*/*z*; Found, 369.1598 *m*/*z*.

(*E*)-1-(4-(Benzo­[d]­[1,3]­dioxol-5-ylmethyl)­piperazin-1-yl)-3-(2,6-difluorophenyl)­prop-2-en-1-one (**APA-11**). To a solution of diethyl (2-(4-(benzo­[d]­[1,3]­dioxol-5-ylmethyl)­piperazin-1-yl)-2-oxoethyl)­phosphonate (**I-1**) (55.2 mg, 151 μmol, 1.00 equiv) in THF (1.6 mL) were added NaH (4.34 mg, 181 μmol, 1.20 equiv) and 2,6-difluorobenzaldehyde (19.6 μL, 181 μmol, 1.20 equiv). The reaction mixture was stirred at rt for 18 h, filtered through Celite, and concentrated *in vacuo*. Flash chromatography (CH_2_Cl_2_/MeOH 99:1 to 98:2) provided **APA-11** (55.2 mg, 143 μmol, 95%) as a yellow/orange oil. **TLC** (SiO_2_; CH_2_Cl_2_/MeOH 95:5, UV): R_f_ = 0.23. ^**1**^ **H NMR** (400 MHz, CDCl_3_): δ (ppm) = 7.70 (d, *J* = 15.9 Hz, 1H), 7.28–7.20 (m, 1H), 7.16 (d, *J* = 15.9 Hz, 1H), 6.94–6.86 (m, 2H), 6.84 (s, 1H), 6.75–6.70 (m, 2H), 5.93 (s, 2H), 3.79–3.56 (m, 4H), 3.43 (s, 2H), 2.50–2.38 (m, 4H). ^**13**^ **C NMR** (101 MHz, CDCl_3_): δ (ppm) = 165.4, 161.6 (dd, ^1^ *J*C-F = 254.3 Hz, ^3^ *J*C-F = 8.2 Hz), 147.8, 146.9, 131.5, 130.3 (t, ^3^ *J*C-F = 11.2 Hz), 128.7, 123.5 (t, ^4^ *J*C-F = 8.8 Hz), 122.3, 113.2 (t, ^3^ *J*C-F = 15.0 Hz), 111.8 (d, ^3^ *J*C-F = 26.6 Hz), 109.5, 108.0, 101.0, 62.6, 53.2, 52.6, 45.9, 42.3. ^**19**^ **F-NMR** (376 MHz, CDCl_3_): δ (ppm) = −110.2 (m). **IR** (thin film): ν = 3465, 2899, 2812, 2774, 1649, 1620, 1503, 1489, 1469, 1439, 1368, 1336, 1310, 1284, 1268, 1238, 1200, 1146, 1115, 1096, 1038, 1003, 978, 933, 858, 786, 756, 738, 684, 664, 587, 569, 513, 485, 425 cm^–1^. **HR-MS** (ESI): Calcd for C_21_H_21_F_2_N_2_O_3_ [M + H]^+^, 387.1515 *m*/*z*; Found, 387.1524 *m*/*z*.

(*E*)-1-(4-(Benzo­[d]­[1,3]­dioxol-5-ylmethyl)­piperazin-1-yl)-3-(3-methoxyphenyl)­prop-2-en-1-one (**APA-12**). To a solution of diethyl (2-(4-(benzo­[d]­[1,3]­dioxol-5-ylmethyl)­piperazin-1-yl)-2-oxoethyl)­phosphonate (**I-1**) (28.0 mg, 70.3 μmol, 1.00 equiv) in THF (0.8 mL) were added NaH (2.02 mg, 84.3 μmol, 1.20 equiv) and 3-methoxybenzaldehyde (10.3 μL, 84.3 μmol, 1.20 equiv). The reaction mixture was stirred at rt for 18 h, filtered through Celite, and concentrated *in vacuo*. Preparative **TLC** (CH_2_Cl_2_/MeOH 95:5) provided **APA-12** (24.6 mg, 64.7 μmol, 92%) as a yellow oil. **TLC** (SiO_2_; CH_2_Cl_2_/MeOH 98:2, UV): R_f_ = 0.16. ^**1**^ **H NMR** (400 MHz, CDCl_3_): δ (ppm) = 7.62 (d, *J* = 15.4 Hz, 1H), 7.30–7.25 (m, 1H), 7.11 (d, *J* = 7.6 Hz, 1H), 7.02 (dd, *J* = 2.6, 1.6 Hz, 1H), 6.89 (ddd, *J* = 8.2, 2.6, 0.9 Hz, 1H), 6.87–6.81 (m, 2H), 6.77–6.71 (m, 2H), 5.94 (s, 2H), 3.82 (s, 3H), 3.77–3.60 (m, 4H), 3.44 (s, 2H), 2.50–2.42 (m, 4H). ^**13**^ **C NMR** (101 MHz, CDCl_3_): δ (ppm) = 165.4, 160.0, 147.9, 146.9, 142.7, 136.9, 131.6, 129.9, 122.3, 120.4, 117.6, 115.2, 113.2, 109.5, 108.0, 101.1, 62.7, 55.4, 53.3, 52.7, 46.0, 42.3. IR (thin film): ν = 3053, 2999, 2935, 2895, 2811, 2772, 1647, 1602, 1501, 1488, 1439, 1367, 1333, 1241, 1155, 1115, 1095, 1038, 1000, 979, 933, 869, 843, 810, 785, 779, 736, 701, 681, 651, 569 cm^–1^. **HR-MS** (ESI): Calcd for C_22_H_25_N_2_O_4_ [M + H]^+^, 381.1809 *m*/*z*; Found, 381.1812 *m*/*z*.

(*E*)-1-(4-(Benzo­[d]­[1,3]­dioxol-5-ylmethyl)­piperazin-1-yl)-3-(pyridin-2-yl)­prop-2-en-1-one (**APA-13**). To a solution of diethyl (2-(4-(benzo­[d]­[1,3]­dioxol-5-ylmethyl)­piperazin-1-yl)-2-oxoethyl)­phosphonate (**I-1**) (23.8 mg, 59.7 μmol, 1.00 equiv) in THF (0.8 mL) were added NaH (1.72 mg, 71.7 μmol, 1.20 equiv) and pyridine-2-carbaldehyde (6.85 μL, 71.7 μmol, 1.20 equiv). The reaction mixture was stirred at rt for 18 h, filtered through Celite, and concentrated *in vacuo*. Preparative **TLC** (CH_2_Cl_2_/MeOH 95:5) provided **APA-13** (19.5 mg, 55.5 μmol, 93%) as a yellow oil. **TLC** (SiO_2_; CH_2_Cl_2_/MeOH 95:5, UV): R_f_ = 0.31. ^**1**^ **H NMR** (400 MHz, CDCl_3_): δ (ppm) = 8.61 (ddd, *J* = 4.8, 1.8, 0.8 Hz, 1H), 7.69 (td, *J* = 7.7, 1.8 Hz, 1H), 7.63 (d, *J* = 15.0 Hz, 1H), 7.51 (d, *J* = 15.0 Hz, 1H), 7.34 (dt, *J* = 7.8, 1.1 Hz, 1H), 7.23 (ddd, *J* = 7.6, 4.8, 1.2 Hz, 1H), 6.85 (bs, 1H), 6.79–6.69 (m, 2H), 5.95 (s, 2H), 3.78–3.67 (m, 4H), 3.43 (s, 2H), 2.52–2.39 (m, 4H). ^**13**^ **C NMR** (101 MHz, CDCl_3_): δ (ppm) = 165.2, 153.6, 150.0, 146.9, 141.2, 137.0, 131.6, 125.0, 124.0, 122.4, 121.4, 109.5, 108.1, 101.1, 62.7, 53.4, 52.7, 46.1, 42.3. **IR** (thin film): ν = 3441, 3058, 2811, 1650, 1609, 1566, 1501, 1489, 1439, 1367, 1334, 1310, 1241, 1147, 1115, 1096, 1037, 998, 977, 932, 868, 810, 780, 749 cm^–1^. **HR-MS** (ESI): Calcd for C_20_H_21_N_3_NaO_3_ [M + Na]^+^, 374.1475 *m*/*z*; Found, 374.1479 *m*/*z*.

1-(4-(Benzo­[d]­[1,3]­dioxol-5-ylmethyl)­piperazin-1-yl)-3-(thiophen-2-yl)­propan-1-one (**APA-14**). 3-(2-Thienyl)­propanoic acid (91.0 μL, 516 μmol, 1.20 equiv), HOBt (69.7 mg, 516 μmol, 1.20 equiv), EDC (91.0 μL, 516 μmol, 1.20 equiv), Et_3_N (71.5 μL, 516 μmol, 1.20 equiv), and 1-piperonylpiperazine (94.7 mg, 430 μmol, 1.00 equiv) were dissolved in CH_2_Cl_2_ (2.0 mL). The reaction mixture was heated in the microwave at 80 °C for 3 h. The reaction was quenched with sat. aq. NaHCO_3_ (10 mL), and the solution was extracted with CH_2_Cl_2_ (3 × 10 mL). The combined organic layers were dried over MgSO_4_, filtered, and concentrated *in vacuo*. Flash chromatography (hexane/EtOAc 95:5 to 0:1) provided **APA-14** (142 mg, 397 μmol, 92%) as a colorless/pale-yellow oil. **TLC** (SiO_2_; EtOAc, UV): R_f_ = 0.34. ^**1**^ **H NMR** (400 MHz, CDCl_3_): δ (ppm) = 7.11 (dd, *J* = 5.1, 1.2 Hz, 1H), 6.91 (dd, *J* = 5.2, 3.4 Hz, 1H), 6.85–6.80 (m, 2H), 6.76–6.70 (m, 2H), 5.94 (s, 2H), 3.66–3.59 (m, 2H), 3.43–3.39 (m, 2H), 3.40 (s, 2H), 3.21–3.16 (m, 2H), 2.69–2.62 (m, 2H), 2.40–2.31 (m, 4H). ^**13**^ **C NMR** (101 MHz, CDCl_3_): δ (ppm) = 170.1, 147.8, 146.9, 144.0, 131.6, 126.9, 124.8, 123.5, 122.3, 109.5, 108.0, 101.0, 62.7, 53.0, 52.7, 45.6, 41.8, 35.3, 25.7. **IR** (thin film): ν = 2900, 2810, 2772, 1640, 1501, 1488, 1439, 1367, 1339, 1238, 1145, 1115, 1096, 1036, 1000, 930, 850, 810, 791, 698, 567 cm^–1^. **HR-MS** (ESI): Calcd for C_19_H_23_N_2_O_3_S [M + H]^+^, 359.1424 *m*/*z*; Found, 359.1420 *m*/*z*.

Diethyl (2-(4-benzylpiperazin-1-yl)-2-oxoethyl)­phosphonate (**I-2**). Diethylphosphonoacetic acid (276 μL, 1.72 mmol, 1.20 equiv), HOBt (232 mg, 1.72 mmol, 1.20 equiv), EDC (303 μL, 1.72 mmol, 1.20 equiv), Et_3_N (238 μL, 1.72 mmol, 1.20 equiv), and 1-benzylpiperazine (**I-4**) (250 μL, 1.43 mmol, 1.00 equiv) were dissolved in CH_2_Cl_2_ (5.0 mL). The reaction mixture was heated to 80 °C in a microwave reactor for 3 h. The solution was then washed with sat. aq. NaHCO_3_ (3 × 15 mL), dried over MgSO_4_, filtered, and concentrated *in vacuo*. Flash chromatography (CH_2_Cl_2_/MeOH 98:2 to 95:5) provided **I-2** (507 mg, 1.43 mmol, quant.) as a yellow oil. **TLC** (SiO_2_; CH_2_Cl_2_/MeOH 95:5, UV): R_f_ = 0.14. ^**1**^ **H NMR** (400 MHz, CDCl_3_): δ (ppm) = 7.36–7.16 (m, 5H), 4.19–4.04 (m, 4H), 3.64–3.50 (m, 4H), 3.47 (s, 2H), 3.00 (d, *J =* 22.0 Hz, 2H), 2.51–2.31 (m, 4H), 1.29 (t, *J =* 7.1 Hz, 6H). ^**13**^ **C NMR** (101 MHz, CDCl_3_): δ (ppm) = 163.2 (d, ^3^JC*-P* = 6.2 Hz), 137.7, 129.2, 128.4, 127.4, 62.9, 62.7 (d, ^3^JC-P = 7.4 Hz), 53.1, 52.7, 47.1, 42.2, 34.1, 32.8, 16.5 (d, ^3^JC-P = 8.8 Hz). ^**31**^ **P NMR** (162 MHz, CDCl_3_): δ (ppm) = 21.2 (m). **IR** (thin film): ν = 2980, 2925, 2857, 2811, 2768, 1644, 1442, 1393, 1367, 1349, 1254, 1214, 1146, 1097, 1055, 1026, 1005, 965, 868, 811, 787, 765, 741, 700, 676, 599, 576, 550, 508, 495, 480 cm^–1^. **HR-MS** (ESI): Calcd for C_17_H_28_N_2_O_4_P [M + H]^+^, 355.1781 *m*/*z*; Found, 355.1775 *m*/*z*.

(*E*)-1-(4-Benzylpiperazin-1-yl)-3-(thiophen-2-yl)­prop-2-en-1-one (**APA-15**). To a solution of **I-2** (65.5 mg, 185 μmol, 1.00 equiv) in THF (3.0 mL) were added NaH (5.32 mg, 222 μmol, 1.20 equiv) and thiophene-2-carbaldehyde (**I-5**) (289) (20.7 μL, 222 μmol, 1.20 equiv). The reaction mixture was stirred at rt for 18 h, filtered through Celite, and concentrated *in vacuo*. Preparative **TLC** (CH_2_Cl_2_/MeOH 98:2) provided **APA-15** (52.1 mg, 167 μmol, 90%) as a yellow oil. **TLC** (SiO_2_; CH_2_Cl_2_/MeOH 98:2, UV): R_f_ = 0.23. ^**1**^ **H NMR** (400 MHz, CDCl_3_): δ (ppm) = 7.74 (d, *J =* 15.1 Hz, 1H), 7.32–7.18 (m, 6H), 7.14 (d, *J =* 3.6 Hz, 1H), 6.98–6.94 (m, 1H), 6.60 (d, *J =* 15.1 Hz, 1H), 3.73–3.51 (m, 4H), 3.47 (s, 2H), 2.44–2.38 (m, 4H). **^13^C NMR** (101 MHz, CDCl_3_): δ (ppm) = 165.0, 140.6, 137.7, 135.6, 130.2, 129.2, 128.4, 128.1, 127.4, 127.2, 115.9, 62.9, 53.4, 52.8, 45.9, 42.3. **IR** (thin film): ν = 3062, 3026, 2918, 2856, 2808, 2766, 1638, 1597, 1517, 1494, 1438, 1416, 1365, 1348, 1297, 1272, 1233, 1205, 1145, 1103, 1075, 1041, 999, 966, 911, 854, 824, 800, 740, 698, 663, 597, 577, 523, 487, 466 cm^–1^. **HR-MS** (ESI): Calcd for C_18_H_21_N_2_OS [M + H]^+^, 313.1369 *m*/*z*; Found, 313.1369 *m*/*z*.

*tert-Butyl* 4-(2-(diethoxyphosphoryl)­acetyl)­piperazine-1-carboxylate (**I-7**). Diethylphosphonoacetic acid (519 μL, 3.23 mmol, 1.20 equiv), HOBt (436 mg, 3.23 mmol, 1.20 equiv), EDC (570 μL, 3.23 mmol, 1.20 equiv), Et_3_N (448 μL, 3.23 mmol, 1.20 equiv), and 1-Boc-piperazine (**I-6**) (501 mg, 2.69 mmol, 1.00 equiv) were dissolved in CH_2_Cl_2_ (5.0 mL). The reaction mixture was heated to 80 °C in a microwave reactor for 3 h. The reaction was quenched with sat. aq. NaHCO_3_ (20 mL) and extracted with CH_2_Cl_2_ (3 × 30 mL). The combined organic layers were dried over MgSO_4_, filtered, and concentrated *in vacuo*. Flash chromatography (CH_2_Cl_2_/MeOH 98:2 to 95:5) provided phosphonate **I-7** (941 mg, 2.58 mmol, 96%) as a white solid. **TLC** (SiO_2_; CH_2_Cl_2_/MeOH 95:5, KMnO4): R_f_ = 0.25. **Mp**: 105.7–108.9 °C. ^**1**^ **H NMR** (400 MHz, CDCl_3_): δ (ppm) = 4.11 (dq, *J =* 8.1, 7.1 Hz, 4H), 3.57–3.34 (m, 8H), 3.02 (d, *J =* 22.1 Hz, 2H), 1.40 (s, 9H), 1.28 (td, *J =* 7.0, 0.5 Hz, 6H). **^13^C NMR** (101 MHz, CDCl_3_): δ (ppm) = 163.4 (d, ^3^JC-P = 5.8 Hz), 154.6, 80.3, 62.7 (d, ^3^JC-P = 7.2 Hz), 46.8, 43.7, 43.1, 41.9, 34.2, 32.9, 28.4, 16.4 (d, ^3^JC-P = 8.0 Hz). ^**31**^ **P NMR** (162 MHz, CDCl_3_): δ (ppm) = 20.8 (m). **IR** (thin film): ν = 2979, 2931, 1697, 1649, 1416, 1365, 1286, 1251, 1169, 1119, 1054, 1026, 997, 968, 864, 832, 771 cm^–1^. **HR-MS** (ESI): Calcd for C_15_H_29_N_2_NaO_6_P [M + Na]^+^, 387.1655 *m*/*z*; Found, 387.1655 *m*/*z*.

*tert*-Butyl (*E*)-4-(3-(thiophen-2-yl)­acryloyl)­piperazine-1-carboxylate (**I-8**). To a solution of **I-7** (1.01 g, 2.77 mmol, 1.00 equiv) in THF (40 mL) were added NaH (79.6 mg, 3.32 mmol, 1.20 equiv) and thiophene-2-carbaldehyde (**I-5**) (289) (310 μL, 3.32 mmol, 1.20 equiv). The reaction mixture was stirred at rt for 18 h, filtered through Celite, and concentrated *in vacuo*. Flash chromatography (hexane/EtOAc 2:1 to 1:1) provided **I-8** (837 mg, 2.59 mmol, 94%) as a white solid. **TLC** (SiO_2_; hexane/EtOAc 1:1, UV): R_f_ = 0.42. **Mp**: 132.2–132.8 °C. ^**1**^ **H NMR** (400 MHz, CDCl_3_): δ (ppm) = 7.79 (d, *J =* 15.1 Hz, 1H), 7.29 (d, *J =* 5.1 Hz, 1H), 7.19 (d, *J =* 3.1 Hz, 1H), 7.00 (dd, *J =* 5.1, 3.6 Hz, 1H), 6.62 (d, *J =* 15.0 Hz, 1H), 3.70–3.53 (m, 4H), 3.48–3.40 (m, 4H), 1.45 (s, 9H). **^13^C NMR** (101 MHz, CDCl_3_): δ (ppm) = 165.3, 154.6, 140.3, 136.1, 130.5, 128.1, 127.5, 115.4, 80.3, 45.6, 43.7 (m), 42.0, 28.4. **IR** (thin film): ν = 2977, 2925, 2860, 1694, 1644, 1603, 1517, 1455, 1415, 1365, 1280, 1236, 1205, 1169, 1124, 1082, 1038, 996, 969, 868, 826, 767, 747, 709, 555, 526, 501 cm^–1^. **HR-MS** (ESI): Calcd for C_16_H_22_N_2_NaO_3_S [M + Na]^+^, 345.1243 *m*/*z*; Found, 345.1244 *m*/*z*.

(*E*)-1-(Piperazin-1-yl)-3-(thiophen-2-yl)­prop-2-en-1-one (**I-9**). Carbamate **I-8** (302 mg, 936 μmol, 1.00 equiv) was dissolved in HCl (4 M in dioxane, 10.5 mL, 42.1 mmol, 45.0 equiv). The reaction mixture was stirred at rt for 20 min, then concentrated *in vacuo*. The residue was dissolved in sat. aq. Na_2_CO_3_ (15 mL) and EtOAc (20 mL), and the aqueous layer was extracted with EtOAc (3 × 20 mL). The combined organic layers were dried over MgSO_4_, filtered, and concentrated *in vacuo*. Flash chromatography (CH_2_Cl_2_/MeOH/Et_3_N 80:20:1) provided amine **I-9** (201 mg, 905 μmol, 97%) as an orange oil. **TLC** (SiO_2_; CH_2_Cl_2_/MeOH/Et_3_N 80:20:1, UV): R_f_ = 0.33. ^**1**^ **H NMR** (400 MHz, CD_3_OD): δ (ppm) = 7.72 (dt, *J =* 15.2, 0.7 Hz, 1H), 7.49 (dt, *J =* 5.1, 1.0 Hz, 1H), 7.34 (dt, *J =* 3.6, 0.7 Hz, 1H), 7.08 (dd, *J =* 5.1, 3.6 Hz, 1H), 6.86 (d, *J =* 15.2 Hz, 1H), 3.67–3.65 (m, 4H), 2.89–2.82 (m, 4H). **^13^C NMR** (101 MHz, CD_3_OD): δ (ppm) = 167.3, 141.4, 137.0, 131.6, 129.2, 116.5, 47.5, 46.8, 46.2, 43.9. **IR** (thin film): ν = 3435, 3302, 3080, 2917, 2853, 1636, 1593, 1517, 1441, 1417, 1362, 1325, 1273, 1257, 1236, 1212, 1140, 1115, 1041, 968, 825, 711, 577, 562 cm^–1^. **HR-MS** (ESI): Calcd for C_11_H_14_N_2_NaOS [M + Na]^+^, 245.0719 *m*/*z*; Found, 245.0718 *m*/*z*.

(*E*)-1-(4-(4-(Benzyloxy)­benzyl)­piperazin-1-yl)-3-(thiophen-2-yl)­prop-2-en-1-one (**APA-16**). To a solution of 0.665 mg 4-benzyloxybenzyl alcohol (3.1 mmol, 1 equiv) in CH_2_Cl_2_ (30 mL) were added 10 drops of pyridine, followed by the addition of PBr_3_ (0.53 mL, 5.6 mmol, 1.81 equiv) at 0 °C over a period of 10 min. The reaction mixture was allowed to warm to r.t. and stirred overnight. Water (5 mL) was then added, the phases were separated, and the aqueous phase was extracted with CH_2_Cl_2_. The combined organic extracts were successively washed with sat. aqu. Na_2_CO_3_ and dried over MgSO_4_, and the solvent was evaporated. The residue thus obtained (803 mg) was directly used in the next step without further purification. To a solution of 64.1 mg of **I-9** (78% purity; 225 μmol, 1.00 equiv) in THF (1.5 mL) were added 9.9 mg NaH (247 μmol, 1.10 equiv), and the mixture was stirred at r.t. for 30 min. A solution of 95.4 mg of the above bromide (344 μmol, 1.53 equiv) in THF (1.5 mL) was then added dropwise at 0 °C. The reaction mixture was allowed to warm to r.t. and stirred overnight. The reaction was then quenched with 5 mL sat. aqu. NaHCO_3_, and the aqueous layer was extracted with AcOEt (3 × 10 mL). The combined organic phases were dried over MgSO_4_ and concentrated under reduced pressure. Purification of the residue by flash chromatography (CH_2_Cl_2_/MeOH 19:1) followed by a second purification step by preparative **TLC** in the same solvent mixture gave **APA-16** (59.8 mg, 63%). ^**1**^ **H NMR** (400 MHz, CDCl_3_): δ (ppm) = 7.73 (dt, *J =* 15.1, 0.7 Hz, 1H), 7.40–7.20 (m, 6H), 7.18–7.12 (m, 2H), 7.02–6.76 (m, 4H), 6.59 (d, *J =* 15.1 Hz, 1H), 5.01 (s, 2H), 3.59 (d, *J =* 45.1 Hz, 4H), 3.44 (s, 2H), 2.39 (t, *J =* 5.1 Hz, 4H). **^13^C NMR** (101 MHz, CDCl_3_): δ (ppm) = 164.98, 158.89, 140.51, 137.03, 135.55, 130.20, 129.35, 128.59, 127.96, 127.50, 127.12, 121.68, 115.84, 115.54, 113.66, 77.35, 77.02, 76.71, 69.96, 62.73, 52.72, 42.22. **HR-MS** (ESI): Calcd for C_25_H_26_N_2_O_2_S [M + H]^+^, 419.1783 *m*/*z*; Found, 419.1788 *m*/*z*.

*tert*-Butyl 4-((2,3-dihydrobenzo­[b]­[1,4]­dioxin-6-yl)­methyl)­piperazine-1-carboxylate (**I-11**). To a solution of 1-Boc-piperazine (**I-6**) (250 mg, 1.34 mmol, 1.00 equiv) and 1,4-benzodioxane-6-carbaldehyde (286 mg, 1.74 mmol, 1.30 equiv) in CH_2_Cl_2_ (10 mL) was added at 0 °C NaBH­(OAc)_3_ (711 mg, 3.36 mmol, 2.50 equiv). The reaction mixture was stirred at rt for 18 h. The reaction was then quenched with sat. aq. NaHCO_3_ (10 mL), and the solution was extracted with CH_2_Cl_2_ (3 × 15 mL). The combined organic layers were dried over MgSO_4_, filtered, and concentrated *in vacuo*. Flash chromatography (hexane/EtOAc 3:1 to 2:1) provided **I-11** (444 mg, 1.33 mmol, 99%) as a yellow oil. **TLC** (SiO_2_; hexane/EtOAc 2:1, UV): R_f_ = 0.17. ^**1**^ **H NMR** (400 MHz, CDCl_3_): δ (ppm) = 6.83 (d, *J =* 1.9 Hz, 1H), 6.81–6.74 (m, 2H), 4.24 (s, 4H), 3.45–3.37 (m, 6H), 2.40–2.31 (m, 4H), 1.44 (s, 9H). **^13^C NMR** (101 MHz, CDCl_3_): δ (ppm) = 154.9, 143.4, 142.8, 131.2, 122.3, 118.0, 117.1, 79.7, 64.5, 62.6, 52.9, 44.3, 43.4, 28.6. **IR** (thin film): ν = 2975, 2931, 2873, 2806, 2766, 1689, 1590, 1506, 1477, 1457, 1419, 1393, 1364, 1347, 1331, 1286, 1259, 1245, 1203, 1169, 1120, 1067, 1005, 918, 887, 870, 833, 819, 793, 769, 741, 656, 628, 617, 580, 530 cm^–1^. **HR-MS** (ESI): Calcd for C_18_H_27_N_2_O_4_ [M + H]^+^, 335.1965 *m*/*z*; Found, 335.1971 *m*/*z*.

1-((2,3-Dihydrobenzo­[b]­[1,4]­dioxin-6-yl)­methyl)­piperazine (**I-12**). Carbamate **I-11** (421 mg, 1.26 mmol, 1.00 equiv) was dissolved in HCl (4 M in dioxane, 14.2 mL, 56.6 mmol, 45.0 equiv). The reaction mixture was stirred at rt for 30 min, then concentrated *in vacuo*. Sat. aq. NaHCO_3_ (20 mL) was added, and the aqueous layer was extracted with CH_2_Cl_2_ (5 × 25 mL). The combined organic layers were dried over MgSO_4_, filtered, and concentrated *in vacuo*. Flash chromatography (EtOAc/MeOH/Et_3_N 80:20:5 to 75:25:10) provided amine **I-12** (184 mg, 784 μmol, 62%) as a yellow oil. **TLC** (SiO_2_; CH_2_Cl_2_/MeOH/Et_3_N 80:20:1, UV, KMnO_4_): R_f_ = 0.29. ^**1**^ **H NMR** (400 MHz, CDCl_3_): δ (ppm) = 6.84–6.81 (m, 1H), 6.80–6.74 (m, 2H), 4.23 (s, 4H), 3.37 (s, 2H), 2.91–2.83 (m, 4H), 2.46–2.33 (m, 4H), 2.12 (s, 1H). **^13^C NMR** (101 MHz, CDCl_3_): δ (ppm) = 143.3, 142.7, 131.4, 122.3, 118.1, 117.0, 64.5, 63.1, 54.3, 46.1. **IR** (thin film): ν = 2975, 2931, 2873, 2806, 2766, 1689, 1619, 1590, 1506, 1477, 1457, 1419, 1393, 1364, 1347, 1331, 1286, 1259, 1245, 1203, 1169, 1120, 1067, 1053, 1005, 918, 887, 870, 848, 833, 819, 793, 769, 741, 646, 628, 617, 580, 560, 530 cm^–1^. **HR-MS** (ESI): Calcd for C_13_H_19_N_2_O_2_ [M + H]^+^, 235.1441 *m*/*z*; Found, 235.1440 *m*/*z*.

Diethyl (2-(4-((2,3-dihydrobenzo­[b]­[1,4]­dioxin-6-yl)­methyl)­piperazin-1-yl)-2-oxoethyl)­phosphonate (**I-13**). Diethylphosphonoacetic acid (122 μL, 758 μmol, 1.20 equiv), HOBt (102 mg, 758 μmol, 1.20 equiv), EDC (134 μL, 758 μmol, 1.20 equiv), Et_3_N (105 μL, 758 μmol, 1.20 equiv), and amine **I-12** (148 mg, 632 μmol, 1.00 equiv) were dissolved in CH_2_Cl_2_ (1.5 mL). The reaction mixture was heated to 80 °C in a microwave reactor for 3 h. The reaction was then quenched with sat. aq. NaHCO_3_ (20 mL) and extracted with CH_2_Cl_2_ (3 × 30 mL). The combined organic layers were dried over MgSO_4_, filtered, and concentrated *in vacuo*. Flash chromatography (CH_2_Cl_2_/MeOH 98:2 to 95:5) provided phosphonate **I-13** (236 mg, 572 μmol, 91%) as an orange oil. **TLC** (SiO_2_; CH_2_Cl_2_/MeOH 95:5, UV): R_f_ = 0.23. ^**1**^ **H NMR** (400 MHz, CDCl_3_): δ (ppm) = 6.81 (d, *J =* 1.9 Hz, 1H), 6.79–6.72 (m, 2H), 4.23 (s, 4H), 4.14 (dq, *J =* 8.1, 7.1 Hz, 4H), 3.58 (dt, *J =* 31.5, 4.8 Hz, 4H), 3.39 (s, 2H), 3.03 (d, *J =* 22.0 Hz, 2H), 2.42 (dt, *J =* 29.9, 5.1 Hz, 4H), 1.31 (dt, *J =* 7.0, 0.5 Hz, 6H). **^13^C NMR** (101 MHz, CDCl_3_): δ (ppm) = 163.2 (d, ^3^JC-P = 6.7 Hz), 143.4, 142.8, 130.9, 122.2, 117.9, 117.1, 64.4, 62.7 (d, ^3^JC-P = 7.9 Hz), 62.2, 53.0, 52.5, 47.1, 42.1, 33.4 (d, 1*J*C-P = 132.6 Hz), 16.5 (d, ^3^JC-P = 7.4 Hz). ^**31**^ **P NMR** (162 MHz, CDCl_3_): δ (ppm) = 21.2 (m). **IR** (thin film): ν = 3471, 2981, 2931, 2808, 2766, 1639, 1590, 1506, 1434, 1393, 1366, 1346, 1287, 1257, 1206, 1147, 1122, 1097, 1064, 1021, 1004, 965, 917, 887, 871, 822, 790, 770, 741, 671, 577, 548 cm^–1^. **HR-MS** (ESI): Calcd for C_19_H_30_N_2_O_6_P [M + H]^+^, 413.1836 *m*/*z*; Found, 413.1841 *m*/*z*.

(*E*)-1-(4-((2,3-Dihydrobenzo­[b]­[1,4]­dioxin-6-yl)­methyl)­piperazin-1-yl)-3-(thiophen-2-yl)­prop-2-en-1-one (**APA-17**). To a solution of **I-13** (61.5 mg, 149 μmol, 1.00 equiv) in THF (2.5 mL) were added NaH (4.29 mg, 179 μmol, 1.20 equiv) and thiophene-2-carbaldehyde (**I-5**) (16.7 μL, 179 μmol, 1.20 equiv). The reaction mixture was stirred at rt for 18 h, filtered through Celite, and concentrated *in vacuo*. Flash chromatography (CH_2_Cl_2_/MeOH 98:2 to 95:5) provided **APA-17** (55.1 mg, 149 μmol, quant.) as a yellow/orange oil. **TLC** (SiO_2_; CH_2_Cl_2_/MeOH 95:5, UV): R_f_ = 0.28. ^**1**^ **H NMR** (400 MHz, CDCl_3_): δ (ppm) = 7.79 (d, *J =* 15.1 Hz, 1H), 7.30 (d, *J =* 5.1 Hz, 1H), 7.20 (d, *J =* 3.5 Hz, 1H), 7.02 (dd, *J =* 5.1, 3.6 Hz, 1H), 6.85 (d, *J =* 1.8 Hz, 1H), 6.83–6.76 (m, 2H), 6.65 (d, *J =* 15.0 Hz, 1H), 4.25 (s, 4H), 3.77–3.60 (m, 4H), 3.46 (s, 2H), 2.52–2.45 (m, 4H). **^13^C NMR** (101 MHz, CDCl_3_): δ (ppm) = 165.1, 143.5, 143.0, 140.6, 135.7, 130.3, 128.1, 127.3, 122.4, 118.1, 117.2, 115.9, 64.5, 62.3, 53.2, 52.6, 45.8, 42.2. **IR** (thin film): ν = 3064, 2924, 2872, 2806, 2766, 1638, 1593, 1506, 1457, 1433, 1417, 1365, 1345, 1286, 1260, 1238, 1205, 1146, 1122, 1103, 1067, 1044, 1001, 967, 917, 886, 823, 794, 769, 739, 704, 651, 595, 574 cm^–1^. **HR-MS** (ESI): Calcd for C_20_H_23_N_2_O_3_S [M + H]^+^, 371.1424 *m*/*z*; Found, 371.1416 *m*/*z*.

(*E*)-1-(4-(3-Methylbenzyl)­piperazin-1-yl)-3-(thiophen-2-yl)­prop-2-en-1-one (**APA-18**). To a solution of amine **I-9** (20.9 mg, 94.0 μmol, 1.00 equiv) in THF (0.8 mL) were added NaH (2.71 mg, 113 μmol, 1.20 equiv) and 1-(bromomethyl)-3-methylbenzene (15.2 μL, 113 μmol, 1.20 equiv). The reaction mixture was stirred at rt for 18 h, filtered through Celite, and concentrated *in vacuo*. Preparative **TLC** (CH_2_Cl_2_/MeOH 95:5) provided **APA-18** (26.9 mg, 82.4 μmol, 84%) as a yellow oil. **TLC** (SiO_2_; CH_2_Cl_2_/MeOH 95:5, UV): R_f_ = 0.25. ^**1**^ **H NMR** (400 MHz, CDCl_3_): δ (ppm) = 7.80 (d, *J =* 15.1 Hz, 1H), 7.30 (d, *J =* 5.1 Hz, 1H), 7.24–7.07 (m, 5H), 7.02 (dd, *J =* 5.1, 3.6 Hz, 1H), 6.66 (d, *J =* 15.0 Hz, 1H), 3.77–3.60 (m, 4H), 3.50 (s, 2H), 2.51–2.45 (m, 4H), 2.35 (s, 3H). **^13^C NMR** (101 MHz, CDCl_3_): δ (ppm) = 165.1, 140.6, 138.1, 137.5, 135.6, 130.3, 130.0, 128.3, 128.2, 128.1, 127.2, 126.4, 115.9, 63.0, 53.4, 52.9, 45.9, 42.3, 21.5. **IR** (thin film): ν = 3659, 3481, 3062, 2988, 2911, 2806, 2765, 1639, 1599, 1518, 1488, 1456, 1440, 1417, 1366, 1345, 1297, 1272, 1233, 1206, 1146, 1105, 1042, 1001, 967, 901, 855, 825, 804, 775, 745, 699, 578 cm^–1^. **HR-MS** (ESI): Calcd for C_19_H_23_N_2_OS [M + H]^+^, 327.1526 *m*/*z*; Found, 327.1526 *m*/*z*.

(*E*)-1-(4-([1,1′-Biphenyl]-3-ylmethyl)­piperazin-1-yl)-3-(thiophen-2-yl)­prop-2-en-1-one (**APA-19**). To a solution of amine **I-9** (20.9 mg, 94.0 μmol, 1.00 equiv) in THF (0.8 mL) were added NaH (2.71 mg, 113 μmol, 1.20 equiv) and 3-phenylbenzyl bromide (27.9 mg, 113 μmol, 1.20 equiv). The reaction mixture was stirred at rt for 18 h, filtered through Celite, and concentrated *in vacuo*. Preparative **TLC** (CH_2_Cl_2_/MeOH 95:5) provided **APA-19** (31.1 mg, 80.0 μmol, 85%) as a yellow oil. **TLC** (SiO_2_; CH_2_Cl_2_/MeOH 95:5, UV): R_f_ = 0.27. ^**1**^ **H NMR** (400 MHz, CDCl_3_): δ (ppm) = 7.82 (d, *J =* 15.1 Hz, 1H), 7.66–7.61 (m, 2H), 7.59 (bs, 1H), 7.54 (dt, *J =* 7.7, 1.6 Hz, 1H), 7.50–7.32 (m, 6H), 7.23 (dt, *J =* 3.7, 0.9 Hz, 1H), 7.05 (dd, *J =* 5.1, 3.6 Hz, 1H), 6.69 (d, *J =* 15.1 Hz, 1H), 3.85–3.60 (m, 6H), 2.62–2.49 (m, 4H). **^13^C NMR** (101 MHz, CDCl_3_): δ (ppm) = 165.1, 141.5, 141.1, 140.6, 138.2, 135.7, 130.3, 128.9, 128.2, 128.1, 128.0, 127.5, 127.3, 127.3, 126.3, 115.9, 63.0, 53.4, 52.9, 45.9, 42.3. **IR** (thin film): ν = 3657, 3479, 3060, 2989, 2936, 2911, 2805, 2766, 1638, 1597, 1518, 1479, 1455, 1439, 1417, 1365, 1345, 1299, 1271, 1234, 1206, 1145, 1105, 1076, 1042, 1001, 966, 904, 862, 824, 808, 790, 755, 729, 700, 665, 644, 616, 601, 579, 520 cm^–1^. **HR-MS** (ESI): Calcd for C_24_H_25_N_2_OS [M + H]^+^, 389.1682 *m*/*z*; Found, 389.1683 *m*/*z*.

(*E*)-1-(4-(3-Hydroxybenzyl)­piperazin-1-yl)-3-(thiophen-2-yl)­prop-2-en-1-one (**APA-20**). To a solution of amine **I-9** (43.3 mg, 195 μmol, 1.00 equiv) and 3-hydroxybenzaldehyde (34.5 mg, 253 μmol, 1.30 equiv) in CH_2_Cl_2_ (1.6 mL) was added at 0 °C NaBH­(OAc)_3_ (103 mg, 487 μmol, 2.50 equiv). The reaction mixture was stirred at rt for 18 h. The reaction was then quenched with sat. aq. NaHCO_3_ (10 mL) and extracted with CH_2_Cl_2_ (3 × 10 mL). The combined organic layers were dried over MgSO_4_, filtered, and concentrated *in vacuo*. Flash chromatography (CH_2_Cl_2_/MeOH 99:1 to 95:5) provided **APA-20** (62.7 mg, 191 μmol, 98%) as a beige oil/amorphous solid. **TLC** (SiO_2_; CH_2_Cl_2_/MeOH 9:1, UV): R_f_ = 0.61. ^**1**^ **H NMR** (400 MHz, CDCl_3_): δ (ppm) = 7.81 (d, *J =* 15.2 Hz, 1H), 7.32–7.29 (m, 1H), 7.19 (d, *J =* 3.6 Hz, 1H), 7.15 (t, *J =* 7.7 Hz, 1H), 7.02 (dd, *J =* 5.1, 3.6 Hz, 1H), 6.86–6.73 (m, 3H), 6.65 (d, *J =* 15.1 Hz, 1H), 3.80–3.56 (m, 4H), 3.46 (s, 2H), 2.54–2.40 (m, 4H). **^13^C NMR** (101 MHz, CDCl_3_): δ (ppm) = 165.5, 156.7, 140.4, 138.8, 136.3, 130.7, 129.7, 128.2, 127.6, 121.1, 116.3, 115.5, 114.9, 62.8, 53.4, 52.7, 45.9, 42.4. **IR** (thin film): ν = 3212, 3104, 3003, 2917, 2815, 2771, 1634, 1583, 1518, 1458, 1445, 1418, 1364, 1343, 1298, 1277, 1240, 1215, 1153, 1105, 1080, 1042, 1001, 965, 930, 864, 825, 801, 778, 757, 704, 580 cm^–1^. **HR-MS** (ESI): Calcd for C_18_H_21_N_2_O_2_S [M + H]^+^, 329.1318 *m*/*z*; Found, 329.1318 *m*/*z*.

(*E*)-1-(4-(3-Methoxybenzyl)­piperazin-1-yl)-3-(thiophen-2-yl)­prop-2-en-1-one (**APA-21**). To a solution of amine **I-9** (16.3 mg, 73.3 μmol, 1.00 equiv) and 3-methoxybenzaldehyde (11.6 μL, 95.3 μmol, 1.30 equiv) in CH_2_Cl_2_ (0.8 mL) was added at 0 °C NaBH­(OAc)_3_ (38.8 mg, 183 μmol, 2.50 equiv). The reaction mixture was stirred at rt for 18 h. The reaction was then quenched with sat. aq. NaHCO_3_ (5 mL), and the solution was extracted with CH_2_Cl_2_ (3 × 5 mL). The combined organic layers were dried over MgSO_4_, filtered, and concentrated *in vacuo*. Preparative **TLC** (CH_2_Cl_2_/MeOH 95:5) provided **APA-21** (22.2 mg, 64.8 μmol, 88%) as a yellow oil. **TLC** (SiO_2_; CH_2_Cl_2_/MeOH 95:5, UV): R_f_ = 0.24. ^**1**^ **H NMR** (400 MHz, CDCl_3_): δ (ppm) = 7.79 (dt, *J =* 15.1, 0.7 Hz, 1H), 7.30 (dt, *J =* 5.1, 1.0 Hz, 1H), 7.23 (d, *J =* 8.0 Hz, 1H), 7.22–7.19 (m, 1H), 7.03 (dd, *J =* 5.1, 3.6 Hz, 1H), 6.93–6.88 (m, 2H), 6.82 (ddd, *J =* 8.2, 2.6, 1.0 Hz, 1H), 6.66 (d, *J =* 15.1 Hz, 1H), 3.81 (s, 3H), 3.77–3.60 (m, 4H), 3.52 (s, 2H), 2.52–2.45 (m, 4H). **^13^C NMR** (101 MHz, CDCl_3_): δ (ppm) = 165.1, 159.8, 140.6, 139.4, 135.7, 130.3, 129.4, 128.1, 127.3, 121.6, 115.9, 114.8, 112.8, 62.9, 55.4, 53.4, 52.9, 45.9, 42.3. **IR** (thin film): ν = 3384, 2997, 2922, 2855, 2834, 2810, 2768, 1637, 1596, 1586, 1518, 1488, 1455, 1438, 1418, 1365, 1346, 1290, 1262, 1239, 1209, 1153, 1105, 1080, 1042, 1001, 968, 862, 826, 778, 743, 695, 578, 521 cm^–1^. **HR-MS** (ESI): Calcd for C_19_H_23_N_2_O_2_S [M + H]^+^, 343.1475 *m*/*z*; Found, 343.1477 *m*/*z.*

(*E*)-1-(4-(3-Propoxybenzyl)­piperazin-1-yl)-3-(thiophen-2-yl)­prop-2-en-1-one (**APA-22**). To a solution of **APA-20** (18.7 mg, 56.9 μmol, 1.00 equiv) in DMF (0.8 mL) were added Cs_2_CO_3_ (22.3 mg, 68.3 μmol, 1.20 equiv) and 1-bromopropane (6.21 μL, 68.3 μmol, 1.20 equiv). The reaction mixture was stirred at rt for 18 h and then filtered through Celite. The filter cake was washed with EtOAc, and the combined filtrates were concentrated *in vacuo* and dried under high vacuum. Preparative **TLC** (CH_2_Cl_2_/MeOH 95:5) provided **APA-22** (17.8 mg, 48.0 μmol, 84%) as a yellow oil. **TLC** (SiO_2_; CH_2_Cl_2_/MeOH 95:5, UV): R_f_ = 0.21. ^**1**^ **H NMR** (400 MHz, CDCl_3_): δ (ppm) = 7.80 (d, *J =* 15.1 Hz, 1H), 7.30 (d, *J =* 5.1 Hz, 1H), 7.25–7.19 (m, 2H), 7.03 (dd, *J =* 5.1, 3.6 Hz, 1H), 6.92–6.87 (m, 2H), 6.83–6.79 (m, 1H), 6.66 (d, *J =* 15.1 Hz, 1H), 3.93 (t, *J =* 6.6 Hz, 2H), 3.78–3.59 (m, 4H), 3.51 (s, 2H), 2.54–2.43 (m, 4H), 1.86–1.76 (m, 2H), 1.04 (t, *J =* 7.4 Hz, 3H). **^13^C NMR** (101 MHz, CDCl_3_): δ (ppm) = 165.1, 159.4, 140.6, 139.3, 135.7, 130.3, 129.4, 128.1, 127.3, 121.4, 116.0, 115.4, 113.4, 69.6, 62.9, 53.4, 52.9, 45.9, 42.3, 22.8, 10.7. **IR** (thin film): ν = 2963, 2935, 2877, 2808, 2766, 1641, 1600, 1518, 1488, 1441, 1417, 1393, 1365, 1346, 1292, 1264, 1236, 1207, 1146, 1106, 1066, 1044, 1001, 968, 907, 863, 825, 802, 775, 744, 697, 580 cm^–1^. **HR-MS** (ESI): Calcd for C_21_H_27_N_2_O_2_S [M + H]^+^, 371.1791 *m*/*z*; Found, 371.1791 *m*/*z*.

(*E*)-1-(4-(3-Butoxybenzyl)­piperazin-1-yl)-3-(thiophen-2-yl)­prop-2-en-1-one (**APA-23**). To a solution of **APA-20** (12.3 mg, 37.5 μmol, 1.00 equiv) in DMF (0.8 mL) were added Cs_2_CO_3_ (14.6 mg, 44.9 μmol, 1.20 equiv) and 1-bromobutane (4.83 μL, 44.9 μmol, 1.20 equiv). The reaction mixture was stirred at rt for 18 h and then filtered through Celite. The filter cake was washed with EtOAc, and the combined filtrates were concentrated *in vacuo* and dried under high vacuum. Preparative **TLC** (CH_2_Cl_2_/MeOH 95:5) provided **APA-23** (11.5 mg, 29.9 μmol, 80%) as a pale-yellow oil. **TLC** (SiO_2_; CH_2_Cl_2_/MeOH 95:5, UV): R_f_ = 0.22. ^**1**^ **H NMR** (400 MHz, CDCl_3_): δ (ppm) = 7.80 (d, *J =* 15.2 Hz, 1H), 7.30 (d, *J =* 5.1 Hz, 1H), 7.25–7.19 (m, 2H), 7.03 (dd, *J =* 5.1, 3.6 Hz, 1H), 6.91–6.86 (m, 2H), 6.83–6.79 (m, 1H), 6.66 (d, *J =* 15.1 Hz, 1H), 3.96 (t, *J =* 6.5 Hz, 2H), 3.79–3.59 (m, 4H), 3.51 (s, 2H), 2.54–2.43 (m, 4H), 1.81–1.73 (m, 2H), 1.55–1.45 (m, 2H), 0.98 (t, *J =* 7.4 Hz, 3H). **^13^C NMR** (101 MHz, CDCl_3_): δ (ppm) = 165.1, 159.4, 140.6, 139.3, 135.7, 130.3, 129.4, 128.1, 127.3, 121.4, 116.0, 115.4, 113.3, 67.8, 62.9, 53.4, 52.9, 45.9, 42.3, 31.5, 19.4, 14.0. **IR** (thin film): ν = 3067, 2956, 2933, 2871, 2808, 2766, 1640, 1600, 1518, 1488, 1440, 1417, 1394, 1365, 1345, 1291, 1263, 1235, 1206, 1156, 1146, 1104, 1068, 1042, 1002, 968, 863, 825, 802, 778, 696, 579 cm^–1^. **HR-MS** (ESI): Calcd for C_22_H_29_N_2_O_2_S [M + H]^+^, 385.1944 *m*/*z*; Found, 385.1946 *m*/*z*.

(*E*)-1-(4-(3-(Pentyloxy)­benzyl)­piperazin-1-yl)-3-(thiophen-2-yl)­prop-2-en-1-one (**APA-24**). To a solution of **APA-20** (12.3 mg, 37.5 μmol, 1.00 equiv) in DMF (0.8 mL) were added Cs_2_CO_3_ (14.6 mg, 44.9 μmol, 1.20 equiv) and 1-bromopentane (5.57 μL, 44.9 μmol, 1.20 equiv). The reaction mixture was stirred at rt for 18 h and then filtered through Celite. The filter cake was washed with EtOAc, and the combined filtrates were concentrated *in vacuo* and dried under high vacuum. Preparative **TLC** (CH_2_Cl_2_/MeOH 95:5) provided **APA-24** (11.6 mg, 29.1 μmol, 78%) as a pale-yellow oil. **TLC** (SiO_2_; CH_2_Cl_2_/MeOH 95:5, UV): R_f_ = 0.28. ^**1**^ **H NMR** (400 MHz, CDCl_3_): δ (ppm) = 7.80 (d, *J =* 15.1 Hz, 1H), 7.30 (d, *J =* 5.1 Hz, 1H), 7.25–7.19 (m, 2H), 7.03 (dd, *J =* 5.1, 3.6 Hz, 1H), 6.91–6.86 (m, 2H), 6.82–6.79 (m, 1H), 6.66 (d, *J =* 15.1 Hz, 1H), 3.96 (t, *J =* 6.6 Hz, 2H), 3.78–3.59 (m, 4H), 3.51 (s, 2H), 2.54–2.44 (m, 4H), 1.83–1.74 (m, 2H), 1.49–1.34 (m, 4H), 0.93 (t, *J =* 7.1 Hz, 3H). **^13^C NMR** (101 MHz, CDCl_3_): δ (ppm) = 165.1, 159.4, 140.6, 139.3, 135.7, 130.3, 129.4, 128.1, 127.3, 121.4, 115.9, 115.4, 113.4, 68.1, 62.9, 53.4, 52.9, 45.9, 42.3, 29.2, 28.4, 22.6, 14.2. **IR** (thin film): ν = 2952, 2932, 2869, 2808, 2766, 1640, 1600, 1518, 1488, 1440, 1417, 1365, 1345, 1291, 1262, 1235, 1206, 1155, 1146, 1105, 1077, 1041, 1002, 966, 862, 825, 802, 776, 696, 579 cm^–1^. **HR-MS** (ESI): Calcd for C_23_H_31_N_2_O_2_S [M + H]^+^, 399.2101 *m*/*z*; Found, 399.2102 *m*/*z*.

(*E*)-1-(4-(3-(Hexyloxy)­benzyl)­piperazin-1-yl)-3-(thiophen-2-yl)­prop-2-en-1-one (**APA-25**). To a solution of **APA-20** (12.3 mg, 37.5 μmol, 1.00 equiv) in DMF (0.8 mL) were added Cs_2_CO_3_ (14.6 mg, 44.9 μmol, 1.20 equiv) and 1-bromohexane (6.34 μL, 44.9 μmol, 1.20 equiv). The reaction mixture was stirred at rt for 18 h and then filtered through Celite. The filter cake was washed with EtOAc, and the combined filtrates were concentrated *in vacuo* and dried under high vacuum. Preparative **TLC** (CH_2_Cl_2_/MeOH 95:5) provided **APA-25** (11.3 mg, 27.4 μmol, 73%) as a pale-yellow oil. **TLC** (SiO_2_; CH_2_Cl_2_/MeOH 95:5, UV): R_f_ = 0.30. ^**1**^ **H NMR** (400 MHz, CDCl_3_): δ (ppm) = 7.80 (d, *J =* 15.1 Hz, 1H), 7.30 (d, *J =* 5.1 Hz, 1H), 7.25–7.19 (m, 2H), 7.03 (dd, *J =* 5.1, 3.6 Hz, 1H), 6.91–6.87 (m, 2H), 6.83–6.79 (m, 1H), 6.66 (d, *J =* 15.1 Hz, 1H), 3.96 (t, *J =* 6.6 Hz, 2H), 3.78–3.59 (m, 4H), 3.51 (s, 2H), 2.54–2.43 (m, 4H), 1.82–1.74 (m, 2H), 1.51–1.42 (m, 2H), 1.38–1.31 (m, 4H), 0.94–0.87 (m, 3H). **^13^C NMR** (101 MHz, CDCl_3_): δ (ppm) = 165.1, 159.4, 140.6, 139.3 (HMBC), 135.7, 130.3, 129.4, 128.1, 127.3, 121.4, 115.9, 115.4, 113.4, 68.1, 62.9, 53.4, 52.9, 45.9, 42.3, 31.8, 29.4, 25.9, 22.8, 14.2. **IR** (thin film): ν = 2930, 2858, 2808, 2765, 1642, 1601, 1518, 1488, 1453, 1441, 1417, 1365, 1345, 1291, 1264, 1235, 1206, 1170, 1155, 1105, 1042, 1002, 967, 861, 824, 777, 696, 579 cm^–1^. **HR-MS** (ESI): Calcd for C_24_H_33_N_2_O_2_S [M + H]^+^, 413.2257 *m*/*z*; Found, 413.2259 *m*/*z*.

*tert*-Butyl (*R*)-4-(2-(diethoxyphosphoryl)­acetyl)-2-methylpiperazine-1-carboxylate (**I-15**). Diethylphosphonoacetic acid (482 μL, 3.00 mmol, 1.20 equiv), HOBt (405 mg, 3.00 mmol, 1.20 equiv), EDC (530 μL, 3.00 mmol, 1.20 equiv), Et_3_N (415 μL, 3.00 mmol, 1.20 equiv), and (*R*)-*N*-Boc-2-methylpiperazine (**I-14**) (500 mg, 2.50 mmol, 1.00 equiv) were dissolved in CH_2_Cl_2_ (5.0 mL). The reaction mixture was heated to 80 °C in a microwave reactor for 3 h. The reaction was then quenched with sat. aq. NaHCO_3_ (10 mL) and extracted with CH_2_Cl_2_ (3 × 15 mL). The combined organic layers were dried over MgSO_4_, filtered, and concentrated *in vacuo*. Flash chromatography (CH_2_Cl_2_/MeOH 98:2 to 95:5) provided **I-15** (940 mg, 2.48 mmol, quant.) as a colorless oil. **TLC** (SiO_2_; CH_2_Cl_2_/MeOH 95:5, UV): R_f_ = 0.25. **[α]**_**D**_^**20**^ = −7.53 (*c* 1.20, CHCl_3_). ^**1**^ **H NMR** (400 MHz, CDCl_3_): δ (ppm) = 4.44–4.21 (m, 2H), 4.20–4.09 (m, 4H), 3.92–3.61 (m, 2H), 3.43–2.71 (m, 5H), 1.43 (d, *J =* 1.7 Hz, 9H), 1.31 (td, *J =* 7.1, 4.8 Hz, 6H), 1.12 (dd, *J =* 18.4, 6.7 Hz, 3H). **^13^C NMR** (101 MHz, CDCl_3_): δ (ppm) = 164.3 (d, ^3^JC-P = 6.8 Hz), 163.98 (d, ^3^JC-P = 6.8 Hz), 154.6, 154.5, 80.3, 80.2, 63.0 (d, ^3^JC-P = 7.1 Hz), 62.8 (d, ^3^JC-P = 7.1 Hz), 62.6 (d, ^3^JC-P = 7.1 Hz), 51.0, 47.2, 46.9, 46.8, 46.0, 42.2, 38.7, 38.3, 33.5 (d, ^1^ *J*C-P = 133.4 Hz), 33.3 (d, ^1^ *J*C-P = 133.4 Hz), 28.5, 16.4 (d, ^3^JC-P = 7.1 Hz), 15.4, 15.2. ^**31**^ **P NMR** (162 MHz, CDCl_3_): δ (ppm) = 20.9 (m). **Remarks**: Presence of rotamers. **IR** (thin film): ν = 3470, 2978, 2934, 1693, 1646, 1449, 1405, 1365, 1342, 1331, 1312, 1252, 1168, 1119, 1084, 1028, 969, 896, 862, 812, 773, 703, 661, 593 cm^–1^. **HR-MS** (ESI): Calcd for C_16_H_31_N_2_NaO_6_P [M + Na]^+^, 401.1812 *m*/*z*; Found, 401.1812 *m*/*z*.

*tert*-Butyl (*R,E*)-2-methyl-4-(3-(thiophen-2-yl)­acryloyl)­piperazine-1-carboxylate (**I-16**). To a solution of **I-15** (697 mg, 1.84 mmol, 1.00 equiv) in THF (30 mL) were added NaH (53.0 mg, 2.21 mmol, 1.20 equiv) and thiophene-2-carbaldehyde (**I-5**) (207 μL, 2.21 mmol, 1.20 equiv). The reaction mixture was stirred at rt for 18 h, filtered through Celite, and concentrated *in vacuo*. Flash chromatography (hexane/EtOAc 2:1) provided **I-16** (576 mg, 1.71 mmol, 93%) as a white/pale-yellow solid. **TLC** (SiO_2_; hexane/EtOAc 1:1, UV): R_f_ = 0.35. **[α]**_**D**_^**20**^ = −25.45 (*c* 1.10, CHCl_3_). **Mp**: 122.2–123.1 °C. ^**1**^ **H NMR** (400 MHz, CDCl_3_): δ (ppm) = 7.83 (d, *J =* 15.1 Hz, 1H), 7.32 (ddd, *J =* 5.1, 3.1, 1.1 Hz, 1H), 7.21 (d, *J =* 3.4 Hz, 1H), 7.06–7.01 (m, 1H), 6.71–6.56 (m, 1H), 4.67–4.50 (m, 0.5H), 4.44–4.23 (m, 1.5H), 4.04–3.71 (m, 2H), 3.46–3.21 (m, 1H), 3.16–2.99 (m, 1.5H), 2.89–2.72 (m, 0.5H), 1.46 (s, 9H), 1.14 (dd, *J =* 6.9, 2.8 Hz, 3H). **^13^C NMR** (101 MHz, CDCl_3_): δ (ppm) = 165.9, 154.6, 140.4, 136.2, 130.6, 128.2, 127.5, 115.3, 80.3, 50.1, 47.6, 47.1, 45.9, 45.6, 42.4, 38.9, 38.6, 28.5, 15.7, 15.2. **Remarks**: Presence of rotamers. **IR** (thin film): ν = 3510, 3075, 2975, 2930, 2871, 1687, 1639, 1599, 1518, 1440, 1403, 1364, 1340, 1313, 1275, 1245, 1224, 1204, 1165, 1125, 1097, 1083, 1068, 1040, 966, 901, 880, 856, 824, 804, 750, 703, 665, 572, 522, 499, 487 cm^–1^. **HR-MS** (ESI): Calcd for C_17_H_25_N_2_O_3_S [M + H]^+^, 337.1580 *m*/*z*; Found, 337.1575 *m*/*z*.

*(R,E*)-1-(3-Methylpiperazin-1-yl)-3-(thiophen-2-yl)­prop-2-en-1-one (**I-17**). Carbamate **I-16** (241 mg, 717 μmol, 1.00 equiv) was dissolved in HCl (4 M in dioxane, 8.07 mL, 32.3 mmol, 45.0 equiv). The reaction mixture was stirred at rt for 20 min and then concentrated *in vacuo*. Flash chromatography (EtOAc/MeOH/Et_3_N 95:5:0 to 80:20:1) provided **I-17** (168 mg, 709 μmol, 99%) as a yellow oil. **TLC** (SiO_2_; CH_2_Cl_2_/MeOH 9:1, UV): R_f_ = 0.33. **[α]**_**D**_^**20**^ = +21.00 (*c* 1.00, CHCl_3_). ^**1**^ **H NMR** (400 MHz, CDCl_3_): δ (ppm) = 7.80 (d, *J =* 15.1 Hz, 1H), 7.31 (dt, *J =* 5.0, 1.0 Hz, 1H), 7.23–7.18 (m, 1H), 7.03 (dd, *J =* 5.1, 3.6 Hz, 1H), 6.66 (d, *J =* 15.1 Hz, 1H), 4.57 (bs, 1H), 4.02–3.83 (m, 1H), 3.36–2.65 (m, 5H), 2.47–2.31 (m, 1H), 1.13 (d, *J* = 5.7 Hz, 3H). **^13^C NMR** (101 MHz, CDCl_3_): δ (ppm) = 165.2, 140.6, 135.8, 130.4, 128.1, 127.3, 116.0, 53.1, 51.4, 50.8, 49.3, 46.4, 45.6, 42.6, 19.5. **Remarks**: Presence of rotamers. **IR** (thin film): ν = 3401, 2747, 1636, 1599, 1441, 1220 cm^–1^. **HR-MS** (ESI): Calcd for C_12_H_17_N_2_OS [M + H]^+^, 237.1056 *m*/*z*; Found, 237.1059 *m*/*z*.

*(R,E*)-1-(4-(Benzo­[d]­[1,3]­dioxol-5-ylmethyl)-3-methylpiperazin-1-yl)-3-(thiophen-2-yl)­prop-2-en-1-one (**APA-26**). To a solution of **I-17** (26.2 mg, 111 μmol, 1.00 equiv) and piperonal (21.6 mg, 144 μmol, 1.30 equiv) in CH_2_Cl_2_ (0.8 mL) was added at 0 °C NaBH­(OAc)_3_ (58.7 mg, 277 μmol, 2.50 equiv). The reaction mixture was stirred at rt for 18 h. The reaction was then quenched with sat. aq. NaHCO_3_ (5 mL) and extracted with CH_2_Cl_2_ (3 × 5 mL). The combined organic layers were dried over MgSO_4_, filtered, and concentrated *in vacuo*. Preparative **TLC** (hexane/EtOAc 1:1) provided **APA-26** (27.7 mg, 74.8 μmol, 67%) as a yellow oil. **TLC** (SiO_2_; CH_2_Cl_2_/MeOH 98:2, UV): R_f_ = 0.20. **[α]**_**D**_^**20**^ = −93.12 (*c* 1.02, CHCl_3_). ^**1**^ **H NMR** (400 MHz, CDCl_3_): δ (ppm) = 7.79 (d, *J =* 15.1 Hz, 1H), 7.30 (d, *J =* 5.1 Hz, 1H), 7.20 (d, *J =* 3.5 Hz, 1H), 7.05–7.00 (m, 1H), 6.86 (s, 1H), 6.77–6.72 (m, 2H), 6.65 (d, *J =* 15.1 Hz, 1H), 5.94 (s, 2H), 4.22 (d, *J =* 13.1 Hz, 0.5H), 4.06 (d, *J =* 13.1 Hz, 0.5H), 3.90 (dd, *J =* 21.2, 13.4 Hz, 1H), 3.73 (d, *J =* 12.8 Hz, 1H), 3.41–3.09 (m, 2.5H), 2.96 (t, *J =* 10.9 Hz, 0.5H), 2.78–2.68 (m, 1H), 2.51 (s, 1H), 2.14 (t, *J =* 10.5 Hz, 1H), 1.16 (d, *J =* 6.2 Hz, 3H). **^13^C NMR** (101 MHz, CDCl_3_): δ (ppm) = 165.0, 147.9, 146.8, 140.6, 135.6, 132.4, 130.3, 128.1, 127.2, 122.1, 115.9, 109.4, 108.0, 101.0, 57.8, 57.7, 55.2, 55.1, 52.4, 50.8, 49.6, 48.7, 46.1, 42.5, 15.7, 15.1. **Remarks**: Presence of rotamers. **IR** (thin film): ν = 3068, 2965, 2895, 2808, 1639, 1599, 1502, 1489, 1441, 1420, 1371, 1332, 1274, 1241, 1206, 1146, 1119, 1079, 1038, 966, 930, 858, 822, 777, 748, 706, 581 cm^–1^. **HR-MS** (ESI): Calcd for C_20_H_23_N_2_O_3_S [M + H]^+^, 371.1424 *m*/*z*; Found, 371.1425 *m*/*z*.

*tert*-Butyl (*S*)-4-(2-(diethoxyphosphoryl)­acetyl)-2-methylpiperazine-1-carboxylate (**I-18**). Diethylphosphonoacetic acid (482 μL, 3.00 mmol, 1.20 equiv), HOBt (405 mg, 3.00 mmol, 1.20 equiv), EDC (530 μL, 3.00 mmol, 1.20 equiv), Et_3_N (415 μL, 3.00 mmol, 1.20 equiv), and (*S*)-*N*-Boc-2-methylpiperazine (**I-18**) (500 mg, 2.50 mmol, 1.00 equiv) were dissolved in CH_2_Cl_2_ (5.0 mL). The reaction mixture was heated to 80 °C in a microwave reactor for 3 h. The reaction was then quenched with sat. aq. NaHCO_3_ (10 mL) and extracted with CH_2_Cl_2_ (3 × 15 mL). The combined organic layers were dried over MgSO_4_, filtered, and concentrated *in vacuo*. Flash chromatography (CH_2_Cl_2_/MeOH 98:2 to 95:5) provided **I-19** (935 mg, 2.47 mmol, 99%) as a colorless oil. **TLC** (SiO_2_; CH_2_Cl_2_/MeOH 95:5, UV): R_f_ = 0.27. **[α]**_**D**_^**20**^ = +8.24 (*c* 0.73, CHCl_3_). ^**1**^ **H NMR** (400 MHz, CDCl_3_): δ (ppm) = 4.46–4.23 (m, 2H), 4.21–4.10 (m, 4H), 3.93–3.64 (m, 2H), 3.43–2.73 (m, 5H), 1.45 (d, *J =* 1.4 Hz, 9H), 1.33 (td, *J =* 7.1, 4.5 Hz, 6H), 1.14 (dd, *J =* 18.0, 6.7 Hz, 3H). **^13^C NMR** (101 MHz, CDCl_3_): δ (ppm) = 164.40 (d, ^3^JC-P = 6.8 Hz), 164.0 (d, ^3^JC-P = 7.1 Hz), 154.6, 154.5, 80.4, 80.2, 63.0 (d, ^3^JC-P = 6.9 Hz), 62.8 (d, ^3^JC-P = 6.9 Hz), 62.7 (d, ^3^JC-P = 6.9 Hz), 51.0, 47.2, 47.0, 46.9, 46.1, 42.2, 38.8, 38.3, 33.5 (d, ^1^ *J*C-P = 132.0 Hz), 33.3 (d, ^1^ *J*C-P = 134.4 Hz), 28.5, 16.5 (d, ^3^JC-P = 7.0 Hz), 15.4, 15.2. ^**31**^ **P NMR** (162 MHz, CDCl_3_): δ (ppm) = 20.9 (m). **Remarks**: Presence of rotamers. **IR** (thin film): ν = 3454, 2979, 2932, 1693, 1647, 1451, 1406, 1365, 1342, 1313, 1253, 1169, 1085, 1029, 969, 772, 657, 589, 537 cm^–1^. **HR-MS** (ESI): Calcd for C_16_H_31_N_2_NaO_6_P [M + Na]^+^, 401.1812 *m*/*z*; Found, 401.1811 *m*/*z*.

*tert*-Butyl (*S,E*)-2-methyl-4-(3-(thiophen-2-yl)­acryloyl)­piperazine-1-carboxylate (**I-19**). To a solution of **I-18** (692 mg, 1.83 mmol, 1.00 equiv) in THF (30 mL) were added NaH (52.7 mg, 2.20 mmol, 1.20 equiv) and thiophene-2-carbaldehyde (**I-5**) (205 μL, 2.20 mmol, 1.20 equiv). The reaction mixture was stirred at rt for 18 h, filtered through Celite, and concentrated *in vacuo*. Flash chromatography (hexane/EtOAc 2:1) provided **I-19** (561 mg, 1.69 mmol, 91%) as a white/pale-yellow solid. **TLC** (SiO_2_; hexane/EtOAc 1:1, UV): R_f_ = 0.35. **[α]_D_** ^**20**^ = +24.57 (*c* 1.18, CHCl_3_). **Mp**: 122.0–123.1 °C. ^**1**^ **H NMR** (400 MHz, CDCl_3_): δ (ppm) = 7.84 (dd, *J =* 15.0, 1.0 Hz, 1H), 7.32 (dd, *J =* 5.2, 1.2 Hz, 1H), 7.22 (d, *J =* 3.5 Hz, 1H), 7.06–7.01 (m, 1H), 6.71–6.56 (m, 1H), 4.69–4.53 (m, 0.5H), 4.43–4.24 (m, 1.5H), 4.01–3.72 (m, 2H), 3.47–3.21 (m, 1H), 3.16–3.00 (m, 1.5H), 2.88–2.71 (m, 0.5H), 1.47 (s, 9H), 1.15 (d, *J =* 6.7 Hz, 3H). **^13^C NMR** (101 MHz, CDCl_3_): δ (ppm) = 165.9, 154.6, 140.4, 136.2, 130.6, 128.2, 127.5, 115.3, 80.3, 50.1, 47.6, 47.1, 45.9, 45.6, 42.3, 38.8, 38.5, 28.5, 15.7, 15.2. **Remarks**: Presence of rotamers. **IR** (thin film): ν = 3482, 3077, 2976, 2930, 2871, 1687, 1639, 1599, 1518, 1441, 1403, 1364, 1340, 1313, 1275, 1244, 1224, 1205, 1165, 1125, 1097, 1083, 1068, 1040, 966, 901, 880, 856, 824, 805, 750, 703, 665, 572, 522, 499, 482 cm^–1^. **HR-MS** (ESI): Calcd for C_17_H_25_N_2_O_3_S [M + H]^+^, 337.1580 *m*/*z*; Found, 337.1574 *m*/*z*.

*(S,E)*-1-(3-Methylpiperazin-1-yl)-3-(thiophen-2-yl)­prop-2-en-1-one (**I-20**). Carbamate **I-19** (244 mg, 726 μmol, 1.00 equiv) was dissolved in HCl (4 M in dioxane, 8.17 mL, 32.7 mmol, 45.0 equiv). The reaction mixture was stirred at rt for 20 min and then concentrated *in vacuo*. Flash chromatography (EtOAc/MeOH/Et_3_N 95:5:0 to 80:20:1) provided **I-20** (125 mg, 529 μmol, 73%) as a yellow oil. **TLC** (SiO_2_; CH_2_Cl_2_/MeOH 9:1, UV): R_f_ = 0.33. **[α]**_**D**_^**20**^ = −19.04 (*c* 0.42, CHCl_3_). ^**1**^ **H NMR** (400 MHz, CDCl_3_): δ (ppm) = 7.79 (d, *J =* 15.0 Hz, 1H), 7.29 (dt, *J =* 5.1, 1.0 Hz, 1H), 7.20 (d, *J =* 3.7 Hz, 1H), 7.01 (dd, *J =* 5.1, 3.6 Hz, 1H), 6.65 (d, *J =* 15.1 Hz, 1H), 4.56 (d, *J =* 10.9 Hz, 1H), 3.88 (dd, *J =* 27.5, 11.2 Hz, 1H), 3.23–3.11 (s, 0.5H), 3.09–3.00 (m, 1H), 2.87–2.69 (m, 3H), 2.43–2.31 (m, 0.5H), 1.10 (d, *J =* 5.7 Hz, 3H). **^13^C NMR** (101 MHz, CDCl_3_): δ (ppm) = 165.1, 140.6, 135.7, 130.3, 128.1, 127.2, 116.0, 53.2, 51.3, 50.8, 49.4, 46.5, 46.4, 45.6, 42.7, 19.6. **Remarks**: Presence of rotamers. **IR** (thin film): ν = 3662, 2987, 2901, 1746, 1638, 1599, 1452, 1407, 1394, 1382, 1251, 1228, 1076, 1066, 1055, 893, 773 cm^–1^. **HR-MS** (ESI): Calcd for C_12_H_17_N_2_OS [M + H]^+^, 237.1056 *m*/*z*; Found, 237.1062 *m*/*z*.

*(S,E)*-1-(4-(Benzo­[d]­[1,3]­dioxol-5-ylmethyl)-3-methylpiperazin-1-yl)-3-(thiophen-2-yl)­prop-2-en-1-one (**APA-27**). To a solution of **I-20** (34.8 mg, 147 μmol, 1.00 equiv) and piperonal (28.7 mg, 191 μmol, 1.30 equiv) in CH_2_Cl_2_ (1.0 mL) was added at 0 °C NaBH­(OAc)_3_ (78.0 mg, 368 μmol, 2.50 equiv). The reaction mixture was stirred at rt for 18 h. The reaction was then quenched with sat. aq. NaHCO_3_ (5 mL) and extracted with CH_2_Cl_2_ (3 × 5 mL). The combined organic layers were dried over MgSO_4_, filtered, and concentrated *in vacuo*. Preparative TLC (hexane/EtOAc 1:1) provided **APA-27** (37.2 mg, 100 μmol, 68%) as a yellow oil. **TLC** (SiO_2_; hexane/EtOAc 1:1, UV): R_f_ = 0.15. **[α]**_**D**_^**20**^ = +97.35 (*c* 0.76, CHCl_3_). ^**1**^ **H NMR** (400 MHz, CDCl_3_): δ (ppm) = 7.79 (d, *J =* 15.1 Hz, 1H), 7.33–7.28 (m, 1H), 7.20 (d, *J =* 3.5 Hz, 1H), 7.05–7.00 (m, 1H), 6.86 (s, 1H), 6.76–6.72 (m, 2H), 6.65 (d, *J =* 15.1 Hz, 1H), 5.94 (s, 2H), 4.22 (d, *J =* 13.1 Hz, 0.5H), 4.06 (d, *J =* 13.1 Hz, 0.5H), 3.90 (dd, *J =* 21.2, 13.5 Hz, 1H), 3.73 (d, *J =* 12.9 Hz, 1H), 3.39–3.09 (m, 2.5H), 2.96 (t, *J =* 10.8 Hz, 0.5H), 2.77–2.69 (m, 1H), 2.51 (bs, 1H), 2.14 (t, *J =* 10.7 Hz, 1H), 1.16 (d, *J =* 6.2 Hz, 3H). **^13^C NMR** (101 MHz, CDCl_3_): δ (ppm) = 165.0, 147.9, 146.8, 140.6, 135.7, 132.3, 130.3, 128.1, 127.2, 122.2, 115.9, 109.4, 108.0, 101.1, 57.8, 57.7, 55.2, 55.1, 52.4, 50.8, 49.6, 48.7, 46.1, 42.5, 15.7, 15.1. **Remarks**: Presence of rotamers. **IR** (thin film): ν = 3073, 2969, 2901, 2806, 1640, 1600, 1502, 1489, 1441, 1419, 1371, 1332, 1274, 1242, 1207, 1145, 1119, 1080, 1038, 966, 930, 863, 823, 812, 774, 749, 709 cm^–1^. **HR-MS** (ESI): Calcd for C_20_H_23_N_2_O_3_S [M + H]^+^, 371.1424 *m*/*z*; Found, 371.1419 *m*/*z*.

#### Synthesis of Aminoquinazolines

##### General Methods

All reagents were purchased from commercial suppliers and used without further purification, unless noted otherwise. All chemical reactions occurring solely in an anhydrous organic solvent were carried out under an inert atmosphere of argon or nitrogen, unless noted otherwise. Analytical **TLC** was performed with Merck silica gel 60 F254 plates. Silica gel column chromatography was conducted with a Teledyne Isco CombiFlash Companion system. ^**1**^ **H NMR** spectra were acquired on Bruker 400 MHz instruments and are listed in parts per million downfield from TMS. **LC–MS** was performed on a Shimadzu Nexera LC-30 LCMS-2020, with a Kinetex XB C18 1.7 μm 50 × 2.1 mm column, using water +0.1% TFA and acetonitrile as mobile phases. All synthesized and purchased compounds were >95% pure by **HPLC** analysis and were characterized by the expected parent ion/s in the MS.

The starting materials 4-chloro-2-methylsulfanyl-quinazoline (CAS 58803-74-0, UORSY BBV-40259836), 4-chloro-8-fluoro-2-methylsulfanyl-quinazoline (CAS 1598310-52-1, UORSY BBV-53144655), and 4-chloro-7,8-dihydro-2-(methylthio)-6H-thiopyrano­[3,2-*d*]­pyrimidine (CAS 111896-67-4, Aurora 210.336.838) are commercially available.

##### General Procedure for the Preparation of **AQ-1** to **AQ-38**

The 4-chloro-2-methylsulfanyl-quinazoline (1 equiv), potassium carbonate (2 equiv), and the amine reagent (2 equiv) were dissolved in tetrahydrofuran (10 mL/mmol quinazoline). The solution was heated to 40 °C and stirred overnight. The reaction was confirmed by LCMS, and the solvent was evaporated. The product was quenched with water, extracted 3-fold with dichloromethane, and the organic layer was dried over sodium sulfate. The solvent was evaporated, and the crude product was purified through normal-phase chromatography (ethyl acetate:cyclohexane, starting at 5% ethyl acetate) to receive the final quinazoline derivative.

*2-methylsulfanyl-N-pentyl-quinazolin-4-amine (**AQ-6**).* Following the general procedure, 0.055 g (44%) was obtained as a colorless solid. ^**1**^ **H NMR** (400 MHz, DMSO-d_6_) δ 8.30 (t, J = 5.3 Hz, 1H), 8.19–8.11 (m, 1H), 7.68 (ddd, J = 8.3, 7.0, 1.3 Hz, 1H), 7.55–7.47 (m, 1H), 7.37 (ddd, J = 8.2, 7.0, 1.2 Hz, 1H), 3.50 (q, J = 6.9 Hz, 2H), 2.50 (s, 5H), 1.69–1.60 (m, 2H), 1.39–1.29 (m, 4H), 0.93–0.84 (m, 1H). **LC/MS**: *m*/*z* (%) 262.4 (100, [M + H]^+^). TR = 0.909 min.

*N-heptyl-2-methylsulfanyl-quinazolin-4-amine (**AQ-7**).* Following the general procedure, 0.045 g (32.8%) was obtained as a colorless solid. **^1^H NMR** (400 MHz, DMSO-d_6_) δ 8.43 (s, 1H), 8.20–8.13 (m, 1H), 7.75–7.65 (m, 1H), 7.55–7.47 (m, 2H), 7.43–7.34 (m, 1H), 3.51 (q, J = 6.4 Hz, 2H), 1.64 (p, J = 7.1 Hz, 3H), 1.36–1.29 (m, 6H), 1.26 (d, J = 3.7 Hz, 4H), 0.90–0.81 (m, 3H). **LC/MS**: *m*/*z* (%) 290.5 (100, [M + H]^+^); TR = 1.021 min.

*2-methylsulfanyl-N-octyl-quinazolin-4-amine (**AQ-8**) (CAS 866137-97-5).* Following the general procedure, 0.051 g (35%) was obtained as a colorless solid. **^1^H NMR** (400 MHz, DMSO-d_6_) δ 8.29 (t, J = 5.4 Hz, 1H), 8.19–8.11 (m, 1H), 7.68 (ddd, J = 8.3, 7.0, 1.3 Hz, 1H), 7.54–7.47 (m, 1H), 7.36 (ddd, J = 8.2, 7.0, 1.2 Hz, 1H), 3.50 (q, J = 6.9 Hz, 2H), 2.49 (s, 5H), 1.68–1.59 (m, 2H), 1.38–1.29 (m, 5H), 1.29–1.22 (m, 6H), 0.89–0.80 (m, 2H). **LC/MS**: *m*/*z* (%) 304.5 (100, [M + H]^+^); TR = 1.073 min.

*N-[2-(4-fluorophenyl)­ethyl]-2-methylsulfanyl-quinazolin-4-amine (**AQ-14**).* Following the general procedure, 0.092 g (62%) was obtained as a colorless solid. **^1^H NMR** (500 MHz, DMSO-d_6_) δ 8.43 (t, J = 5.4 Hz, 1H), 8.12 (d, J = 7.7 Hz, 1H), 7.73–7.65 (m, 1H), 7.52 (d, J = 7.8 Hz, 1H), 7.41–7.33 (m, 1H), 7.29 (dd, J = 8.5, 5.7 Hz, 2H), 7.12 (t, J = 8.9 Hz, 1H), 3.75–3.67 (m, 2H), 2.96 (t, J = 7.4 Hz, 2H), 2.52 (s, 2H). **LC/MS**: *m*/*z* (%) 314.3 (100, [M + H]^+^). TR = 0.900 min.

*N-heptyl-2-methylsulfanyl-8-fluoro-quinazolin-4-amine (**AQ-26**)*. Following the general procedure, 0.200 g (0.651 mmol, 99%) was obtained as a colorless solid. **^1^H NMR** (500 MHz, DMSO-d_6_) δH 8.45 (t, J = 5.4 Hz, 1H), 7.98 (d, J = 8.3 Hz, 1H), 7.54 (ddd, J = 10.8, 7.9, 1.1 Hz, 1H), 7.33 (td, J = 8.1, 5.0 Hz, 1H), 3.56–3.44 (m, 2H), 2.51 (s, 3H), 1.63 (q, J = 7.0 Hz, 2H), 1.36–1.19 (m, 8H), 0.91–0.79 (m, 3H). **LC/MS**: *m*/*z* (%) 308.1 (100, [M + H]^+^); TR = 1.067 min.

*2-methylsulfanyl-N-octyl-8-fluoro-quinazolin-4-amine (**AQ-27**)*. Following the general procedure, 0.188 g (0.211 mmol, 89%) was obtained as a colorless solid. ^**1**^ **H NMR** (400 MHz, DMSO-d_6_) δH 8.45 (t, J = 5.6 Hz, 1H), 7.98 (dd, J = 8.3, 1.2 Hz, 1H), 7.54 (ddd, J = 10.9, 7.9, 1.1 Hz, 1H), 7.33 (td, J = 8.1, 5.0 Hz, 1H), 3.50 (td, J = 7.3, 5.7 Hz, 2H), 1.64 (p, J = 7.0 Hz, 2H), 1.44–1.12 (m, 11H), 1.01–0.59 (m, 3H). **LC/MS**: *m*/*z* (%) 322.1 (100, [M + H]^+^); TR = 1.125 min.

*2-methylsulfanyl-N-nonyl-8-fluoro-quinazolin-4-amine (**AQ-28**).* Following the general procedure, 0.201 g (0.599 mmol, 91%) was obtained as a colorless solid. ^**1**^ **H NMR** (500 MHz, DMSO-d_6_) δH 8.46 (t, J = 5.5 Hz, 1H), 7.98 (d, J = 8.3 Hz, 1H), 7.54 (ddd, J = 10.9, 7.9, 1.1 Hz, 1H), 7.34 (td, J = 8.1, 5.0 Hz, 1H), 3.50 (q, J = 6.6 Hz, 2H), 2.50 (d, J = 2.0 Hz, 3H), 1.63 (p, J = 7.0 Hz, 2H), 1.38–1.17 (m, 12H), 0.90–0.79 (m, 3H). **LC/MS**: *m*/*z* (%) 336.3 (100, [M + H]^+^); TR = 1.180 min.

*2-methylsulfanyl-N-undecyl-8-fluoro-quinazolin-4-amine (**AQ-29**).* Following the general procedure, 0.226 g (0.622 mmol, 95%) was obtained as a colorless solid. ^**1**^ **H NMR** (400 MHz, DMSO-d_6_) δH 8.51–8.37 (m, 1H), 7.98 (d, J = 8.3 Hz, 1H), 7.54 (ddd, J = 10.9, 7.9, 1.2 Hz, 1H), 7.33 (td, J = 8.1, 5.0 Hz, 1H), 3.50 (q, J = 6.6 Hz, 2H), 2.51 (s, 3H), 1.63 (p, J = 7.1 Hz, 2H), 1.40–1.13 (m, 16H), 0.92–0.77 (m, 3H). **LC/MS**: *m*/*z* (%) 364.3 (100, [M + H]^+^); TR = 1.302 min.

*N-(3-isobutoxypropyl)-2-methylsulfanyl-8-fluoro-quinazolin-4-amine (**AQ-30**).* Following the general procedure (0.141 g, 0.436 mmol, 67%) was obtained as a colorless solid. ^**1**^ **H NMR** (400 MHz, DMSO-d_6_) δH 8.45 (t, J = 5.4 Hz, 1H), 7.97 (d, J = 8.3 Hz, 1H), 7.54 (ddd, J = 10.9, 7.8, 1.1 Hz, 1H), 7.34 (td, J = 8.1, 5.0 Hz, 1H), 3.58 (q, J = 6.6 Hz, 2H), 3.46 (t, J = 6.1 Hz, 2H), 3.14 (d, J = 6.6 Hz, 2H), 2.52 (s, 3H), 1.92–1.84 (m, 2H), 1.78 (hept, J = 6.7 Hz, 1H), 0.86 (d, J = 6.8 Hz, 6H). **LC/MS**: *m*/*z* (%) 324.1 (100, [M + H]^+^); TR = 0.960 min.

*N-(3-butoxypropyl)-2-methylsulfanyl-8-fluoro-quinazolin-4-amine (**AQ-31**).* Following the general procedure (0.180 g, 0.557 mmol, 85%) was obtained as a colorless solid. ^**1**^ **H NMR** (400 MHz, DMSO-d_6_) δH 8.44 (d, J = 5.0 Hz, 1H), 7.97 (d, J = 8.3 Hz, 1H), 7.54 (ddd, J = 10.8, 7.9, 1.1 Hz, 1H), 7.34 (td, J = 8.1, 5.0 Hz, 1H), 3.57 (q, J = 6.4 Hz, 2H), 3.45 (t, J = 6.1 Hz, 2H), 3.36 (t, J = 6.5 Hz, 2H), 2.52 (s, 3H), 1.87 (p, J = 6.4 Hz, 2H), 1.47 (dq, J = 8.5, 6.6 Hz, 2H), 1.36–1.20 (m, 2H), 0.86 (t, J = 7.3 Hz, 3H). **LC/MS**: *m*/*z* (%) 324.1 (100, [M + H]^+^); TR = 0.953 min.

*N-[3-(cyclopropylmethoxy)­propyl]-2-methylsulfanyl-8-fluoro-quinazolin-4-amine (**AQ-32**).* Following the general procedure, 0.149 g (0.464 mmol, 70.7%) was obtained as a colorless solid. ^**1**^ **H NMR** (400 MHz, DMSO-d_6_) δH 8.45 (t, J = 5.5 Hz, 1H), 7.97 (d, J = 8.3 Hz, 1H), 7.55 (ddd, J = 10.9, 7.9, 1.1 Hz, 1H), 7.34 (td, J = 8.1, 5.1 Hz, 1H), 3.64–3.53 (m, 2H), 3.47 (t, J = 6.2 Hz, 2H), 3.22 (d, J = 6.8 Hz, 2H), 2.52 (s, 3H), 1.88 (p, J = 6.5 Hz, 2H), 0.99 (dddd, J = 13.3, 6.8, 3.2, 1.9 Hz, 1H), 0.50–0.37 (m, 2H), 0.24–0.09 (m, 2H). **LC/MS**: *m*/*z* (%) 322.1 (100, [M + H]^+^); TR = 0.877 min.

*N-[3-(2-ethylhexoxy)­propyl]-2-methylsulfanyl-8-fluoro-quinazolin-4-amine (**AQ-33**).* Following the general procedure (0.233 g, 0.614 mmol, 94%) was obtained as a colorless solid. ^**1**^ **H NMR** (500 MHz, DMSO-d_6_) δH 8.42 (d, J = 5.0 Hz, 1H), 7.97 (d, J = 8.2 Hz, 1H), 7.62–7.44 (m, 1H), 7.33 (td, J = 8.1, 5.0 Hz, 1H), 3.58 (q, J = 6.4, 5.9 Hz, 2H), 3.45 (t, J = 6.1 Hz, 2H), 3.25 (d, J = 5.9 Hz, 2H), 2.51 (s, 3H), 1.88 (p, J = 6.5 Hz, 2H), 1.41 (h, J = 5.9 Hz, 1H), 1.32–1.14 (m, 8H), 0.83 (dt, J = 12.0, 7.1 Hz, 6H). **LC/MS**: *m*/*z* (%) 380.3 (100, [M + H]^+^); TR = 1.210 min.

*N-heptyl-2-methylsulfanyl-7,8-dihydro-6H-thiopyrano­[3,2-d]­pyrimidin-4-amine (**AQ-36**).* Following the general procedure, 0.069 g (52%) was obtained as a colorless solid. ^**1**^ **H NMR** (500 MHz, DMSO-d_6_) δ 3.35 (q, J = 6.4 Hz, 2H), 3.06–2.99 (m, 2H), 2.63 (t, J = 6.3 Hz, 2H), 2.38 (s, 2H), 1.55–1.48 (m, 2H), 1.28–1.22 (m, 11H), 0.85 (t, J = 6.9 Hz, 3H). **LC/MS**: *m*/*z* (%) 312.3 (100, [M + H]^+^); TR = 1.054 min.

*2-methylsulfanyl-N-octyl-7,8-dihydro-6H-thiopyrano­[3,2-d]­pyrimidin-4-amine (**AQ-37**).* Following the general procedure, 0.096 g (69%) was obtained as a colorless solid. ^**1**^ **H NMR** (400 MHz, DMSO-d_6_) δ 3.06–2.98 (m, 2H), 2.63 (t, J = 6.4 Hz, 2H), 2.38 (s, 2H), 2.09–2.01 (m, 2H), 1.56–1.46 (m, 2H), 1.28–1.22 (m, 16H). **LC/MS**: *m*/*z* (%) 326.6 (100, [M + H]^+^); TR = 1.103 min.

*2-methylsulfanyl-N-nonyl-7,8-dihydro-6H-thiopyrano­[3,2-d]­pyrimidin-4-amine (**AQ-38**).* Following the general procedure (0.095 g, 65%) was obtained as a colorless solid. ^**1**^ **H NMR** (400 MHz, DMSO-d_6_) δ 3.36 (d, J = 6.3 Hz, 1H), 3.06–2.98 (m, 2H), 2.62 (t, J = 6.4 Hz, 2H), 2.48 (s, 0H), 2.38 (s, 3H), 1.24 (s, 15H), 0.90–0.81 (m, 3H). **LC/MS**: *m*/*z* (%) 340.3 (100, [M + H]^+^); TR = 1.163 min.

## Supplementary Material







## Notes

Docking of compound **AQ-1** in 7E1W (PDB)
NMR spectra and HPLC traces of tested compounds (PDF)
Molecular formula strings of all tested compounds (CSV)

## ABBREVIATIONS

APA: arylvinylpiperazine amide
AQ: aminoquinazoline
BU: Buruli ulcer
CFU: colony-forming unit
cyt-*bc*_1_ *:aa*_3_: cytochrome *bc*_1_ *:aa*_3_
ECM: extracellular matrix
MBC: minimum bactericidal concentration
*Mtb*: *Mycobacterium tuberculosis*
*Mul*: *Mycobacterium ulcerans*
SI: selectivity index
