# Supplementary material for: The Mycobacterial cyt-bc 1 :aac3 Oxidase as a Drug Target: Activities of Arylvinylpiperazine Amides and Aminoquinazolines against Mycobacterium ulcerans
Source: J Med Chem. 2026 Jul 14;69(14):17260–81. doi: 10.1021/acs.jmedchem.6c01076 (PMC13403322; doi:10.1021/acs.jmedchem.6c01076)
Supplement: Supplementary file 2 [file jm6c01076_si_003.pdf]

Supporting Information

The mycobacterial cyt-*bc₁:aac₃* oxidase as a drug target: Activities of arylvinylpiperazine amides and aminoquinazolines against *Mycobacterium ulcerans*

Louisa Warryn[1,2*], Matthias Witschel[3], Tobias Schehl[3], Daniela Prochazkova[3], Maryline Kienle[4], Francine Horn[4], Karl-Heinz Altmann[4], Daniel H. Paris[1,2], and Gerd Pluschke[1,2].

1. *Swiss Tropical and Public Health Institute, 4123 Allschwil, Switzerland.*
2. *University of Basel, 4001 Basel, Switzerland.*
3. *BASF SE, Ludwigshafen, 67063, Germany.*
4. *Department of Chemistry and Applied Biosciences, Institute of Pharmaceutical Sciences, Swiss Federal Institute of Technology Zürich, CH-8093 Zürich, Switzerland.*

*Corresponding author: louisa.warryn@swisstph.ch

Contents

^{1}H- and ^{13}C-NMR Spectra for Arylvinylpiperazine Amides and Intermediates……………………..…S3

HPLC Traces of Representative Aminoquinazolines…………………………………..……………………..S39

^{1}H-NMR of **APA-5** (400 MHz, $CDCl_3$)

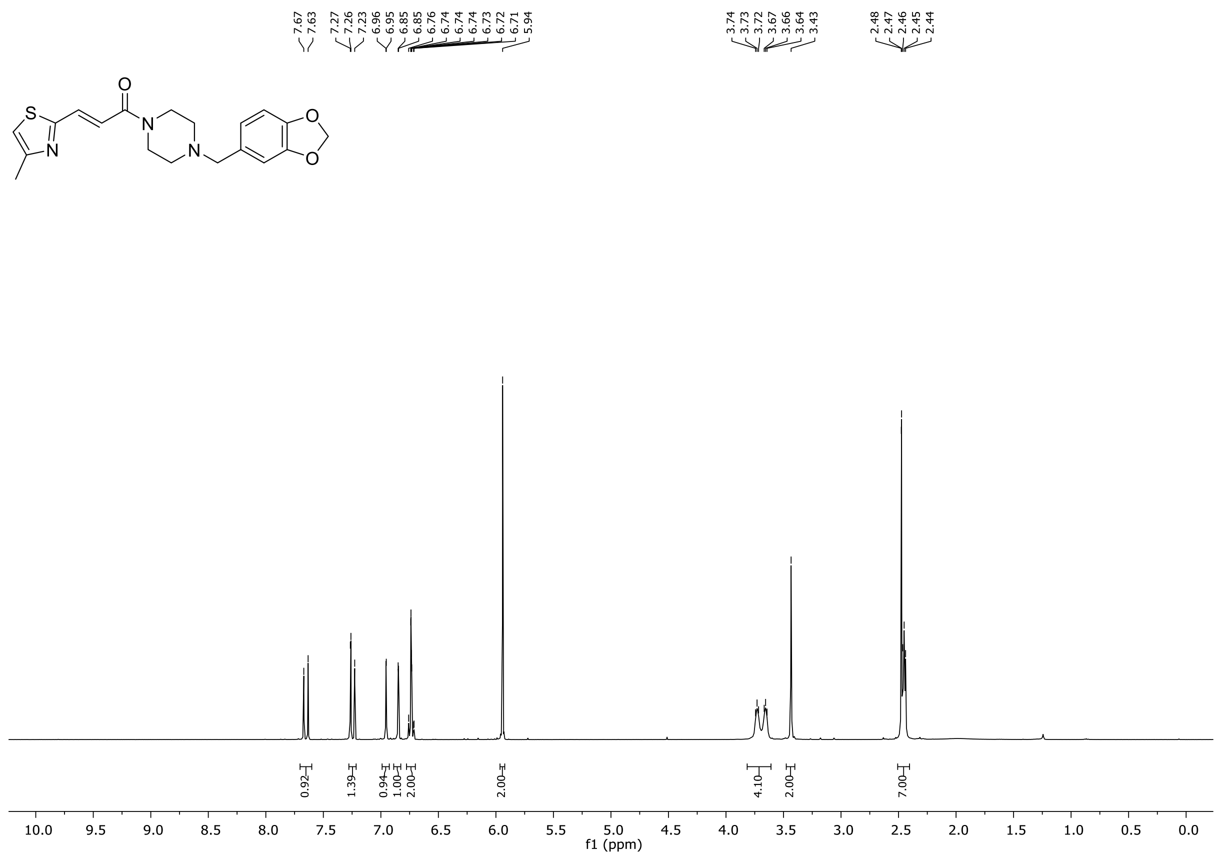

^{13}C-NMR of **APA-5** (101 MHz, $CDCl_3$)

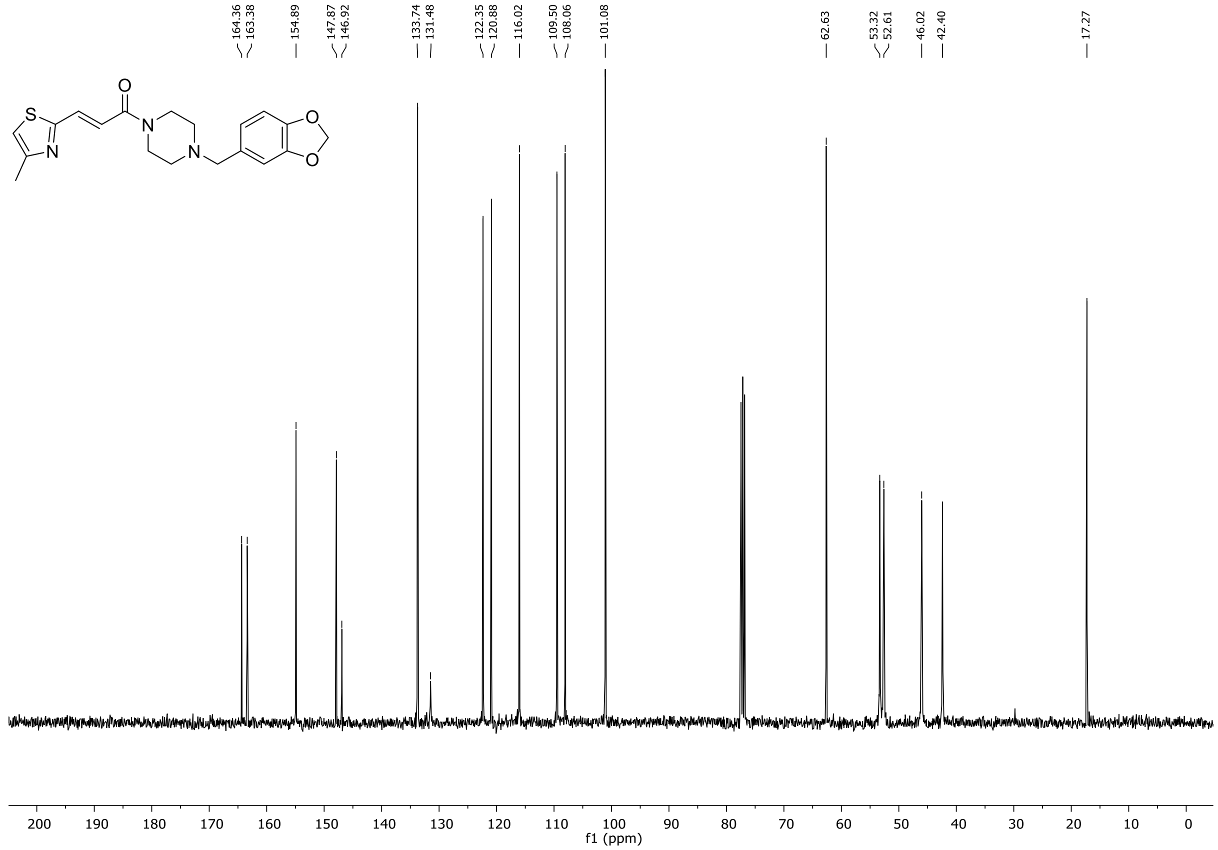

^{1}H-NMR of **APA-6** (400 MHz, $CDCl_3$)

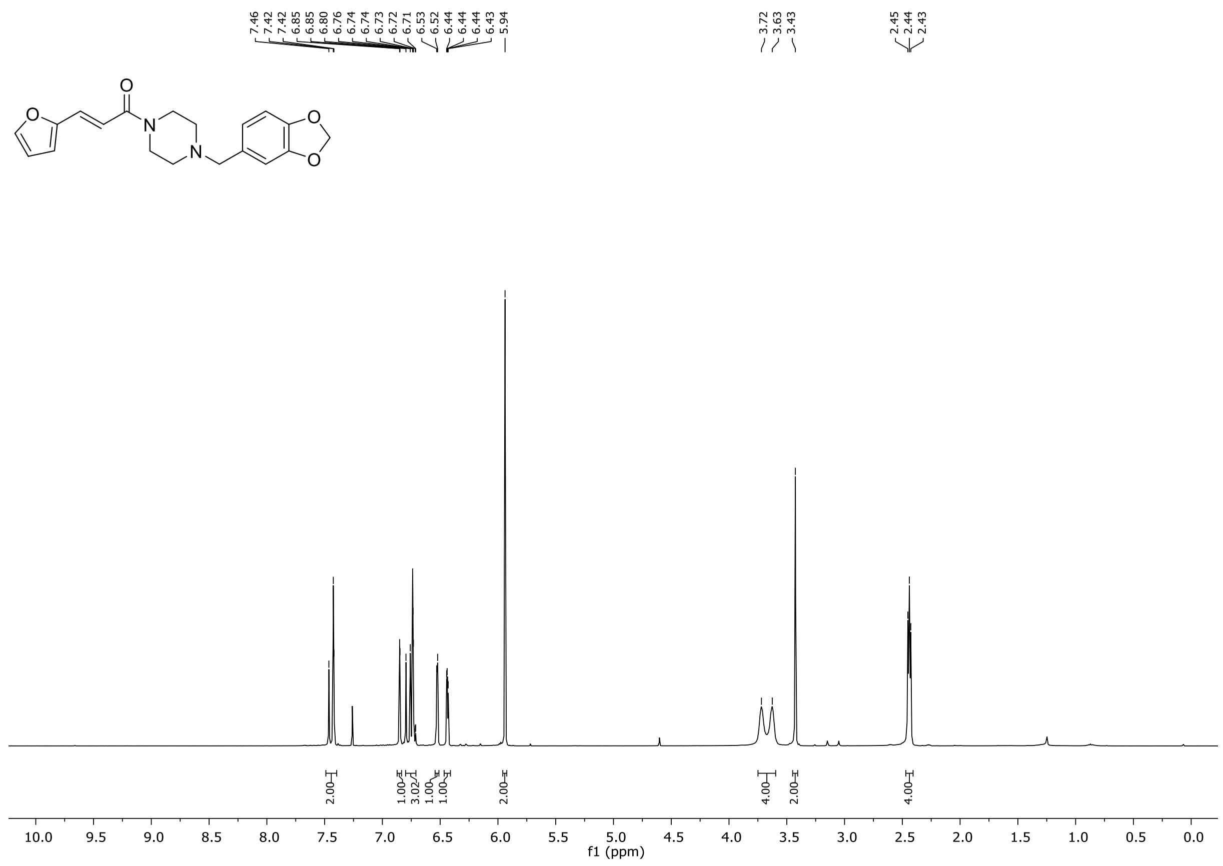

^{13}C-NMR of **APA-6** (101 MHz, $CDCl_3$)

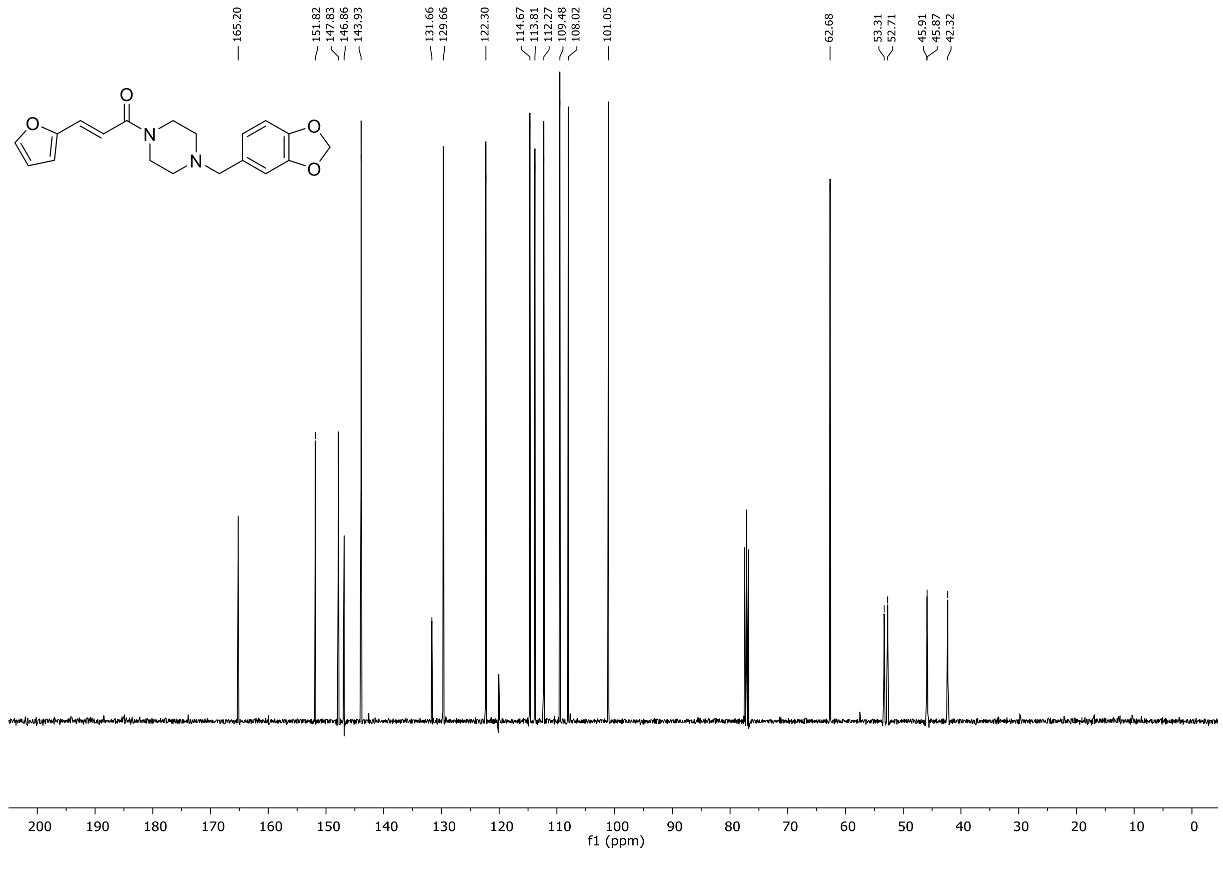

^{1}H-NMR of **APA-8** (400 MHz, $CDCl_3$)

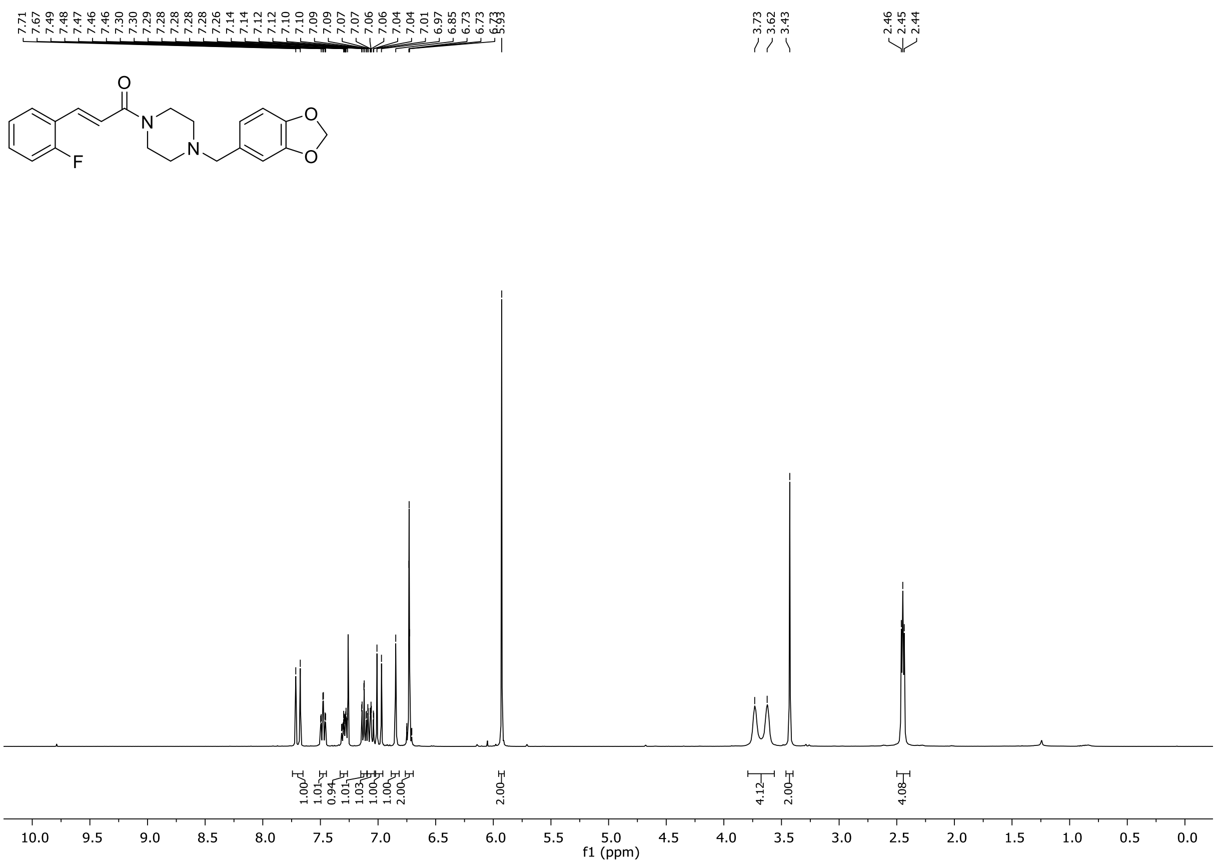

^{13}C-NMR of **APA-8** (101 MHz, $CDCl_3$)

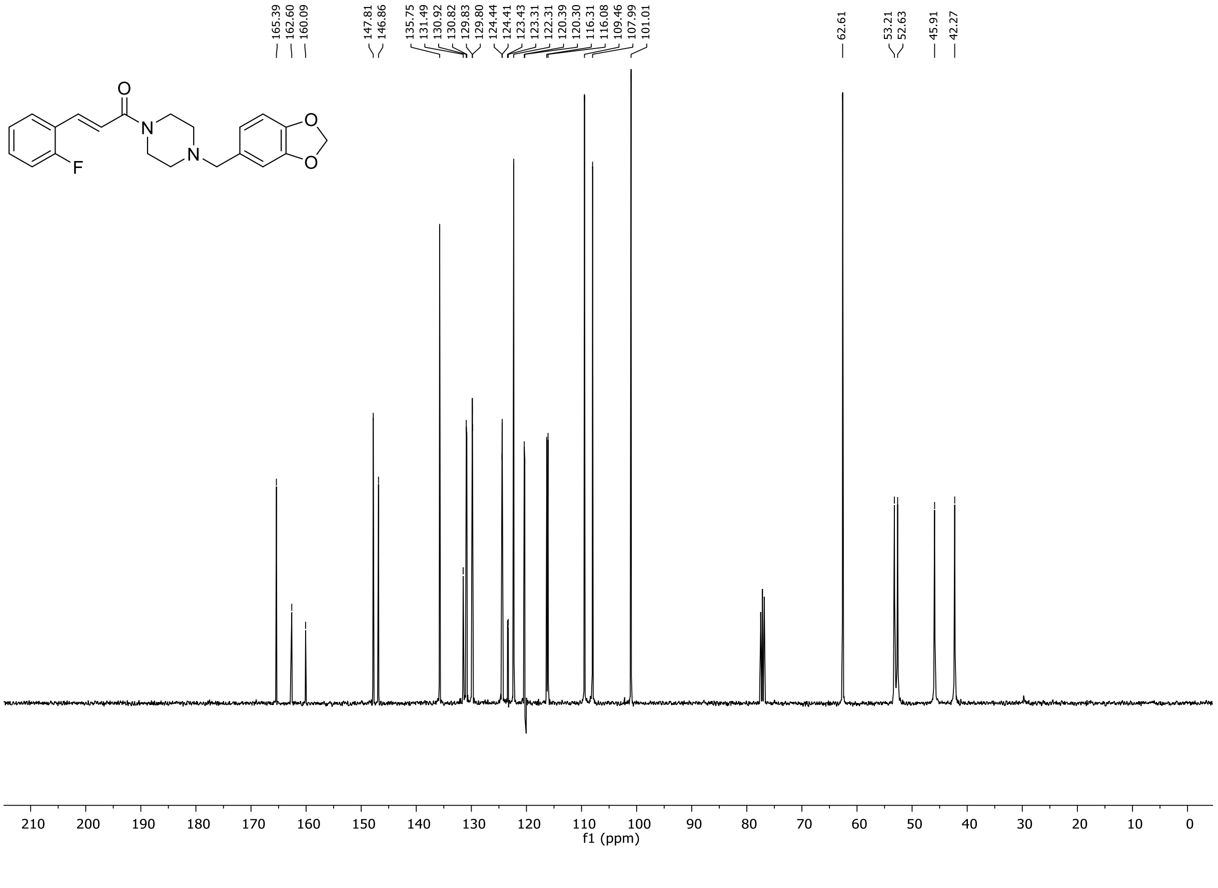

^{1}H-NMR of **APA-9** (400 MHz, $CDCl_3$)

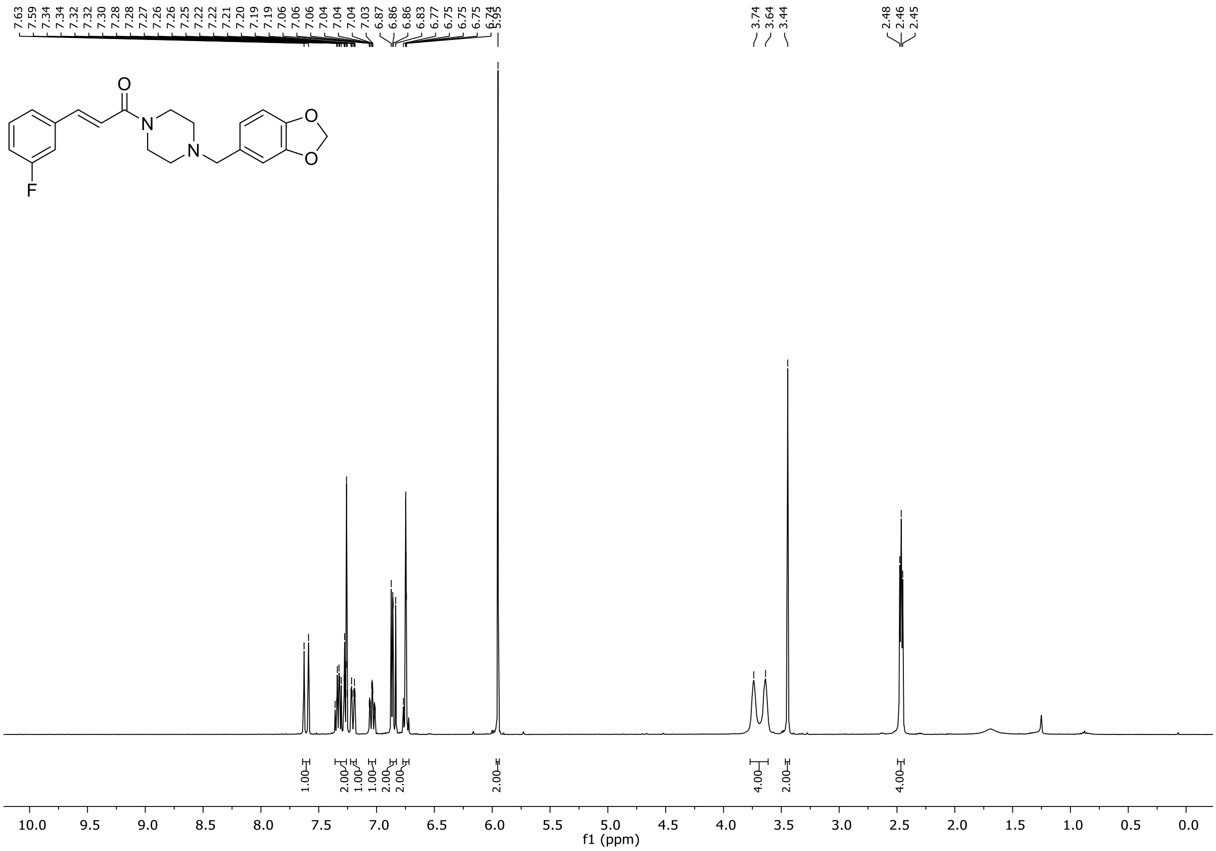

^{13}C-NMR of **APA-9** (101 MHz, $CDCl_3$)

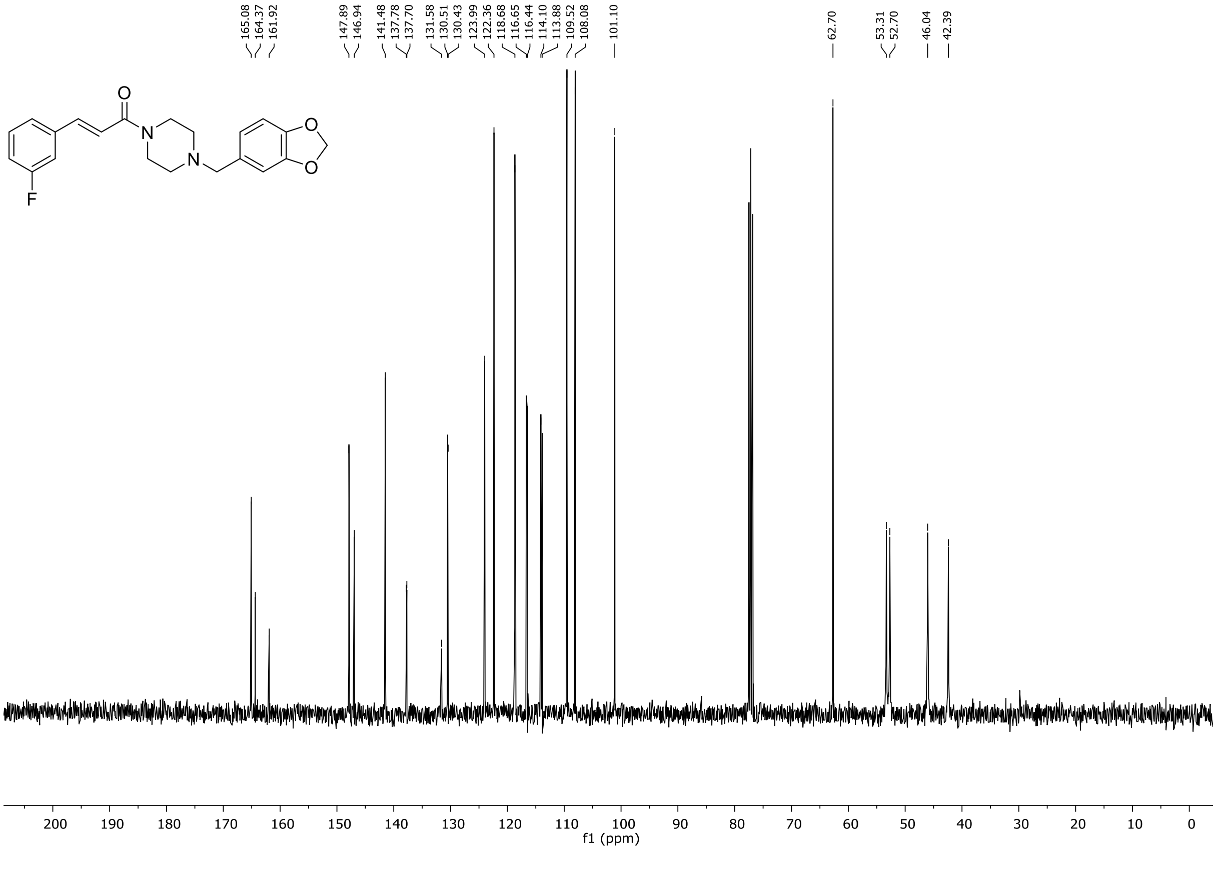

^{1}H-NMR of **APA-10** (400 MHz, $CDCl_3$)

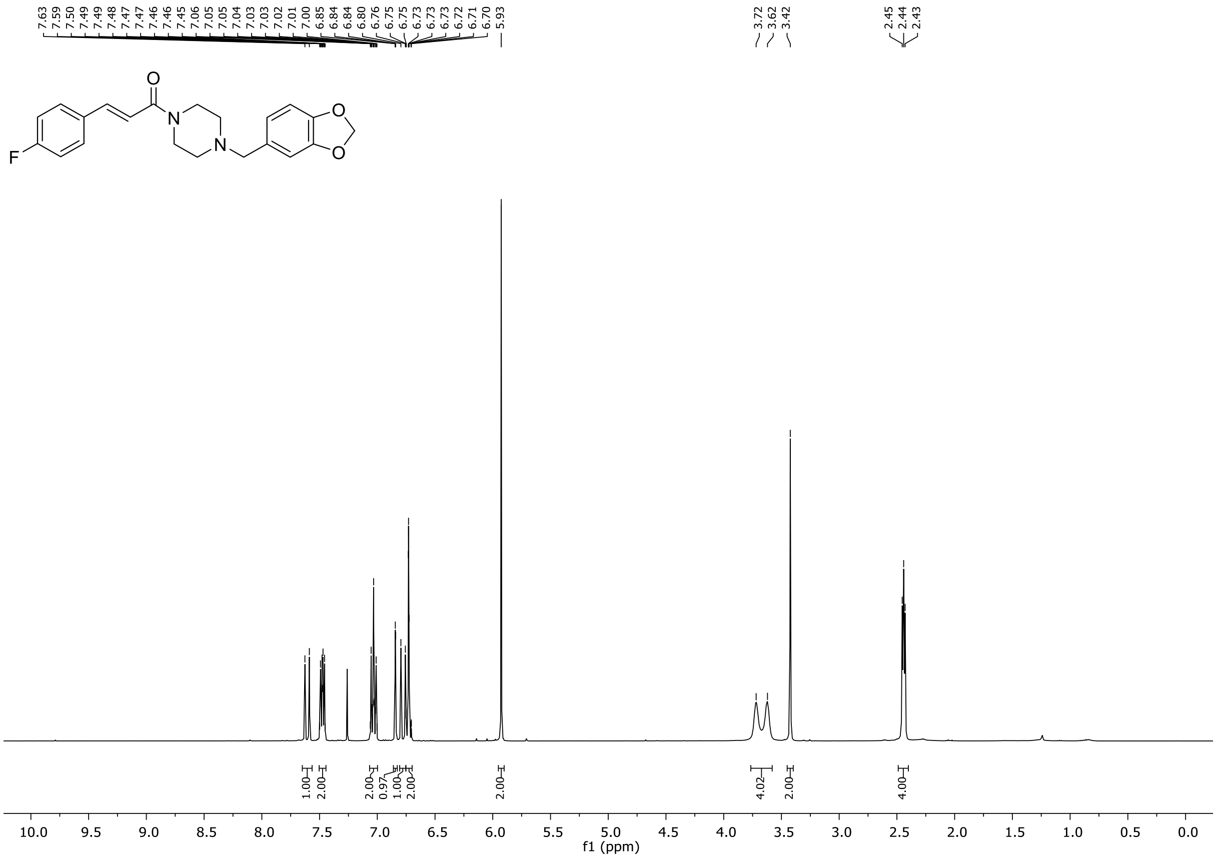

^{13}C-NMR of **APA-10** (101 MHz, $CDCl_3$)

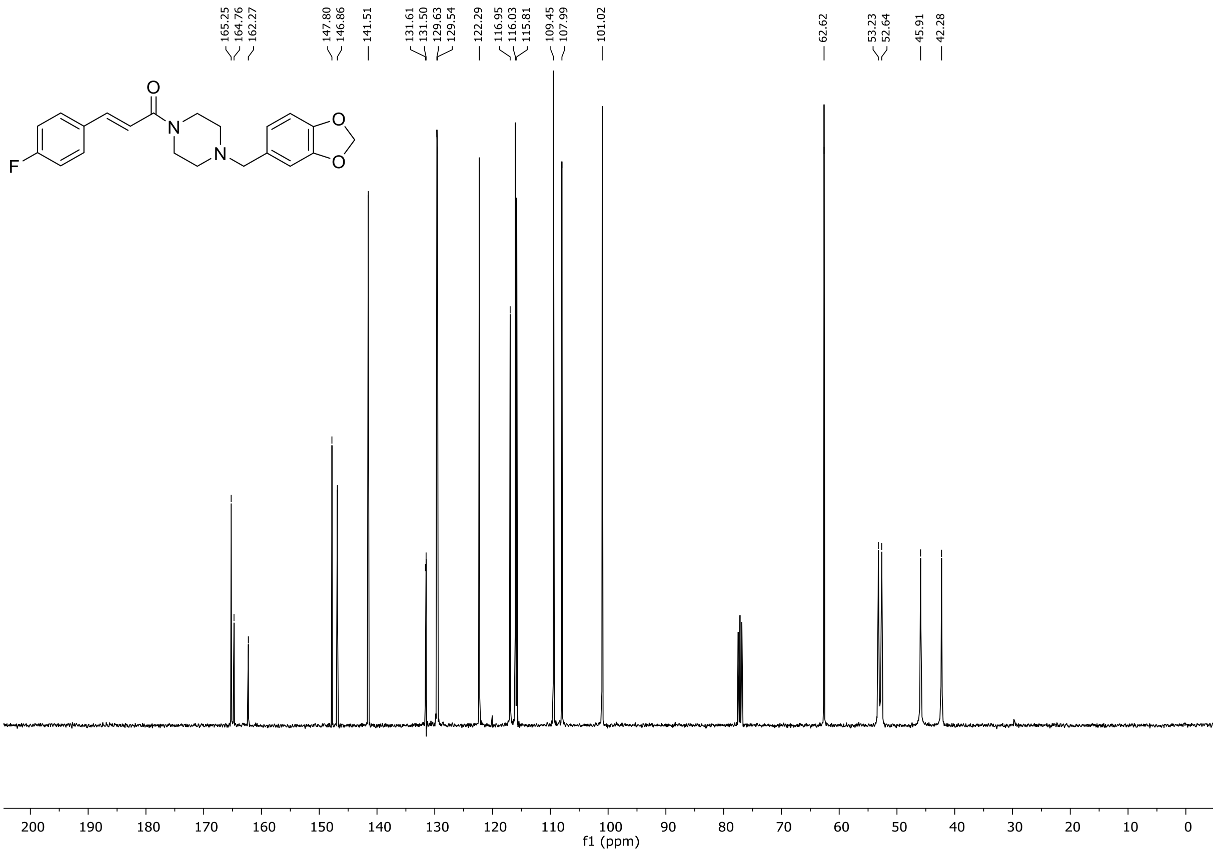

^{1}H-NMR of **APA-11** (400 MHz, $CDCl_3$)

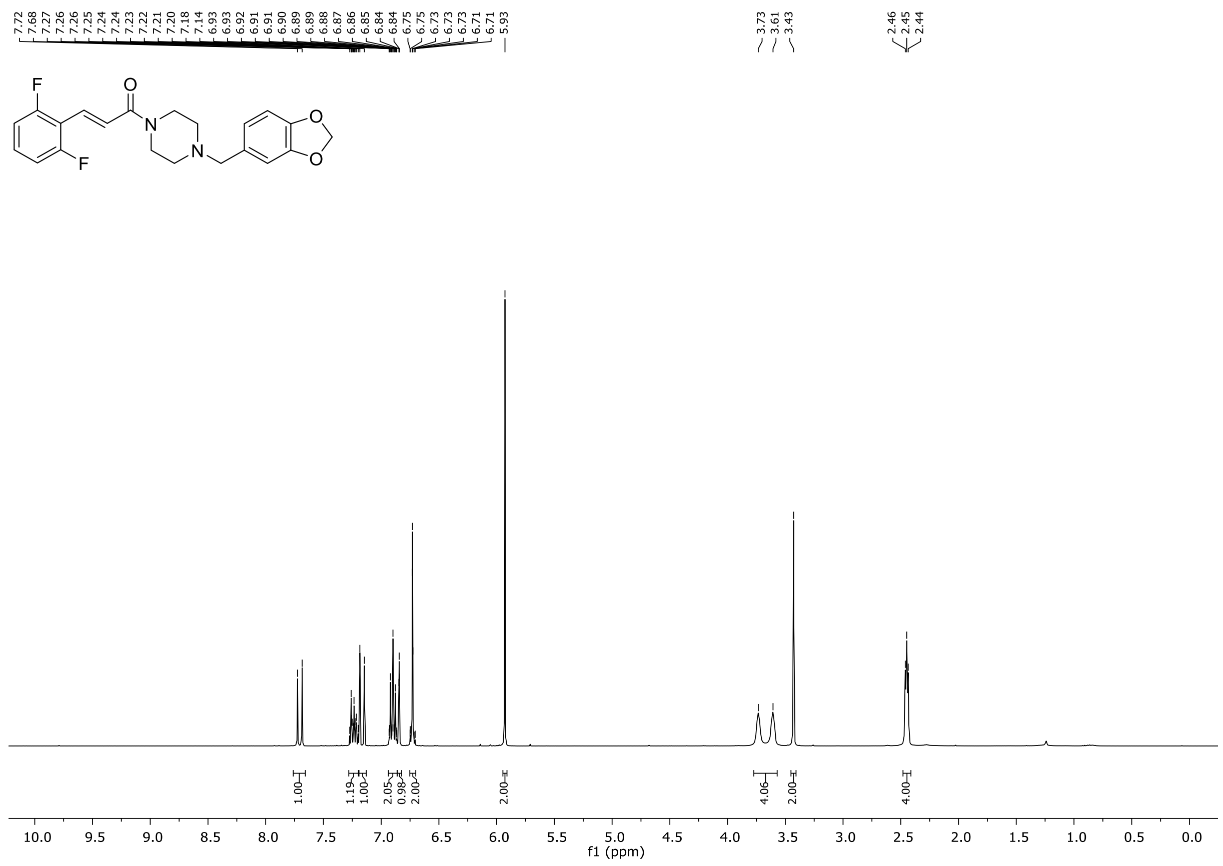

^{13}C-NMR of **APA-11** (101 MHz, $CDCl_3$)

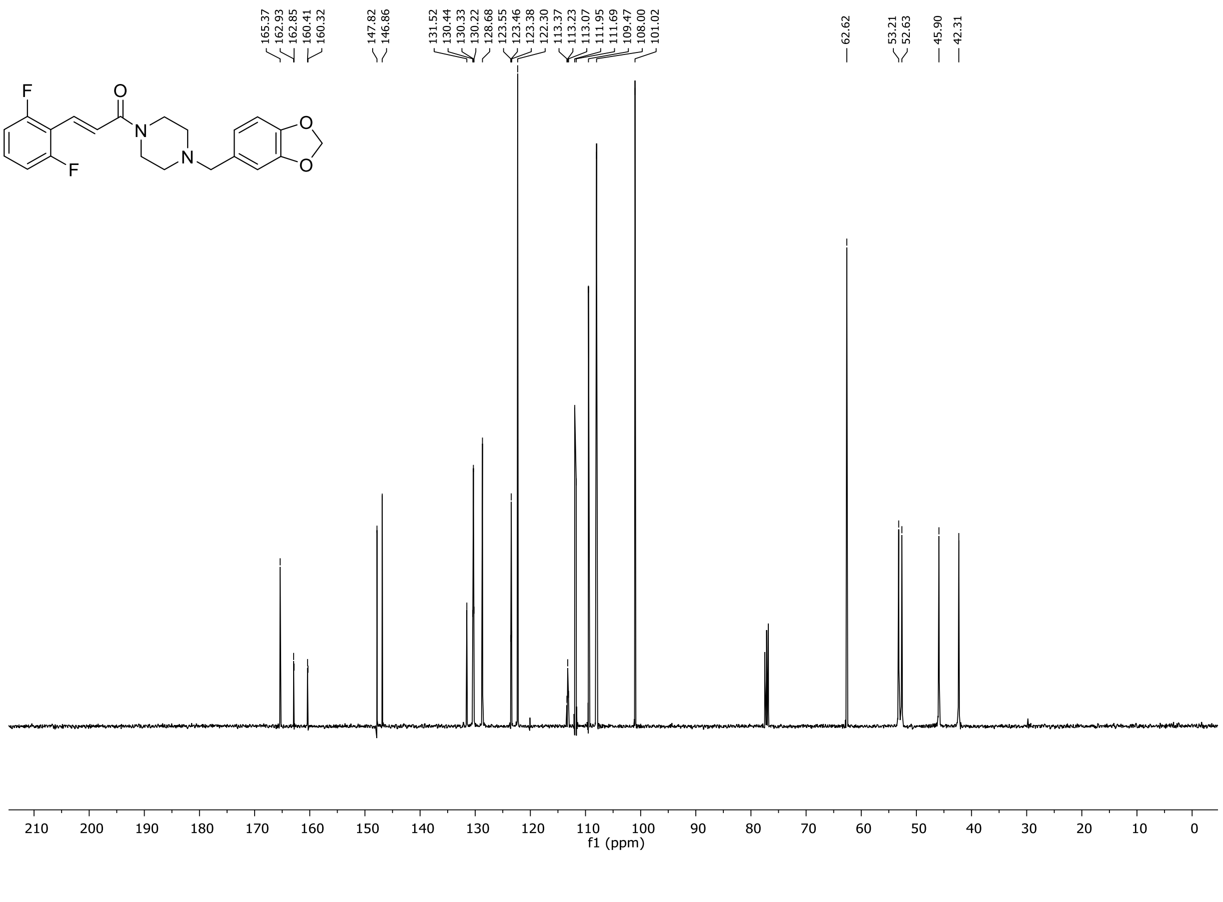

^{1}H-NMR of **APA-12** (400 MHz, $CDCl_3$)

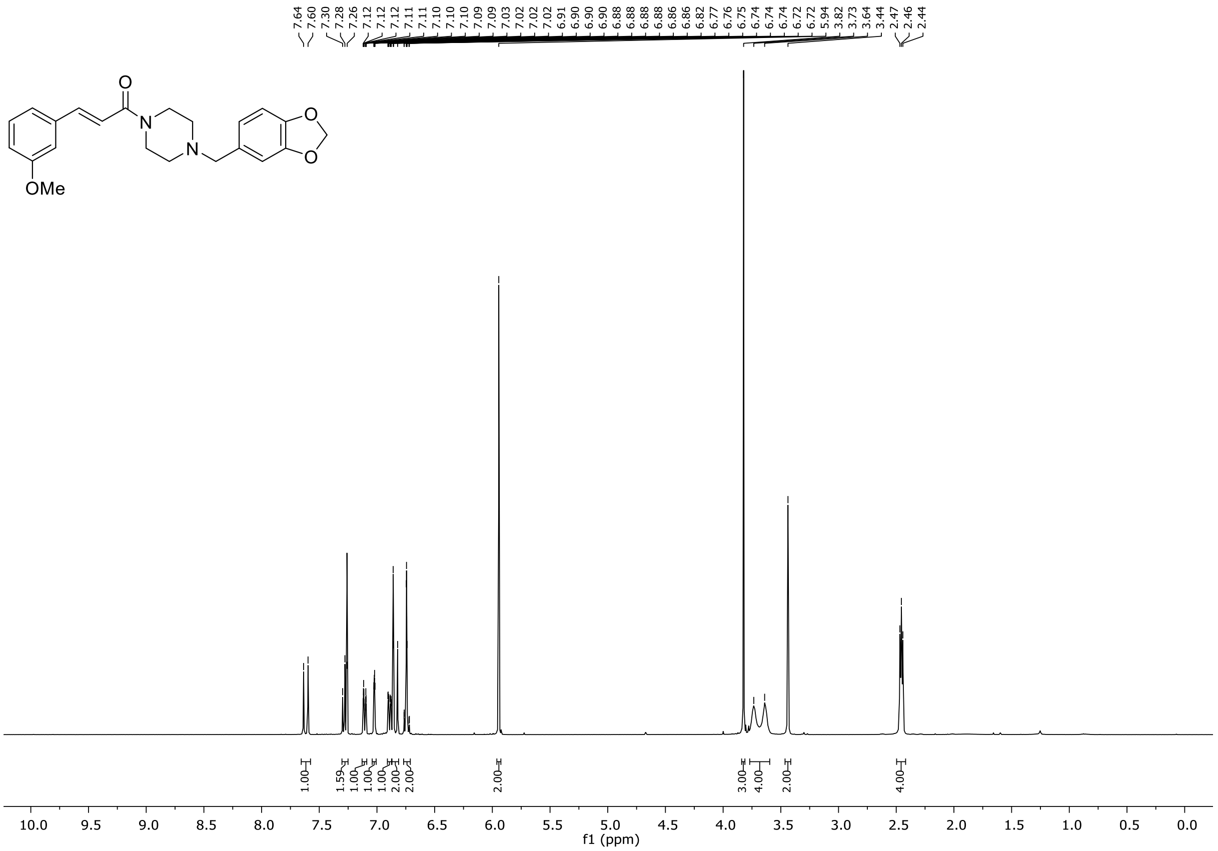

^{13}C-NMR of **APA-12** (101 MHz, $CDCl_3$)

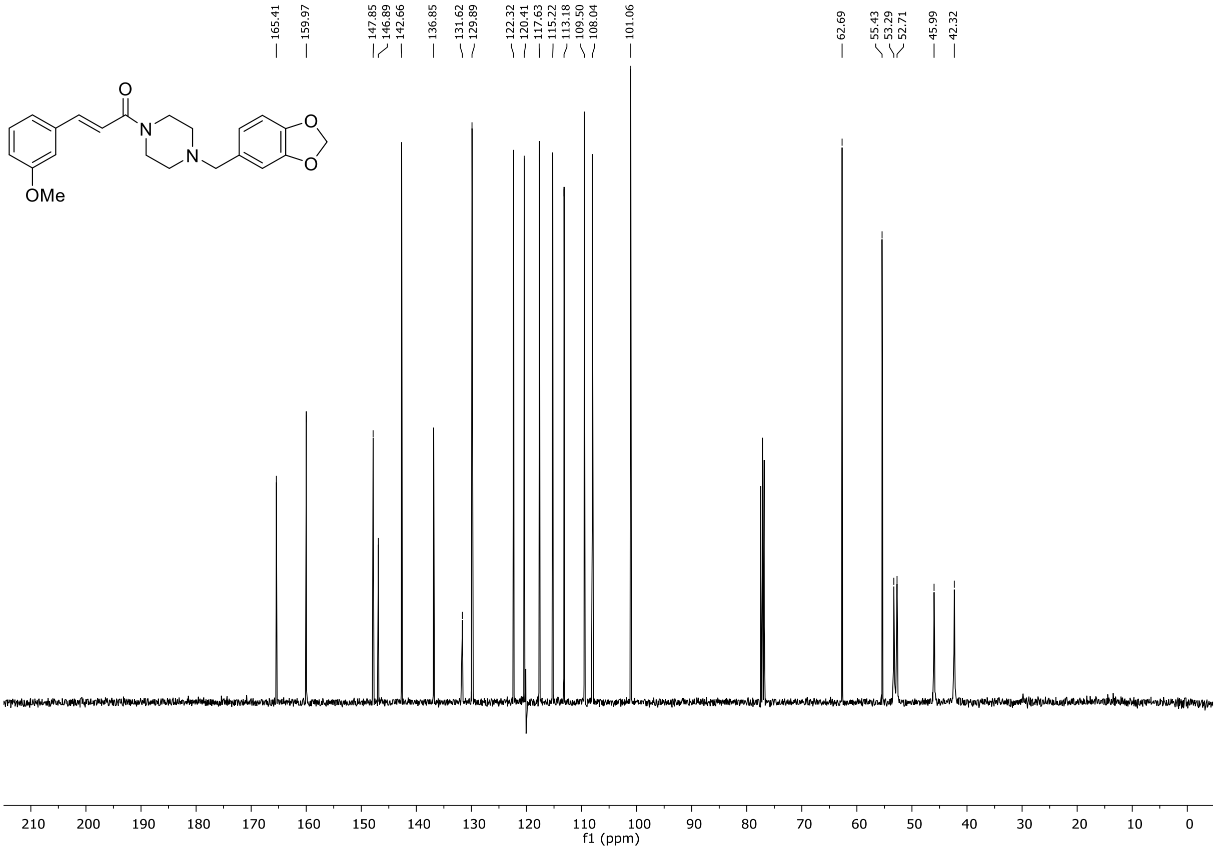

^{1}H-NMR of **APA-13** (400 MHz, $CDCl_3$)

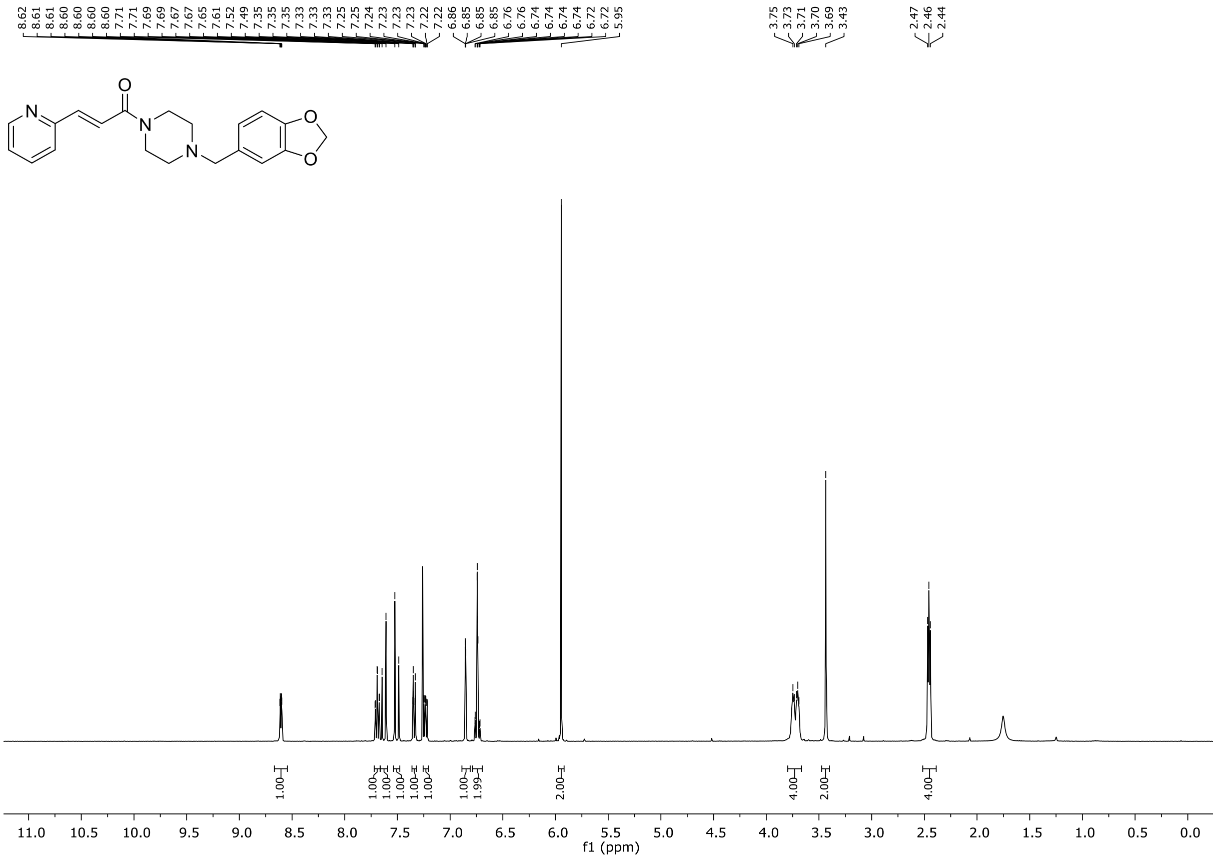

^{13}C-NMR of **APA-13** (101 MHz, $CDCl_3$)

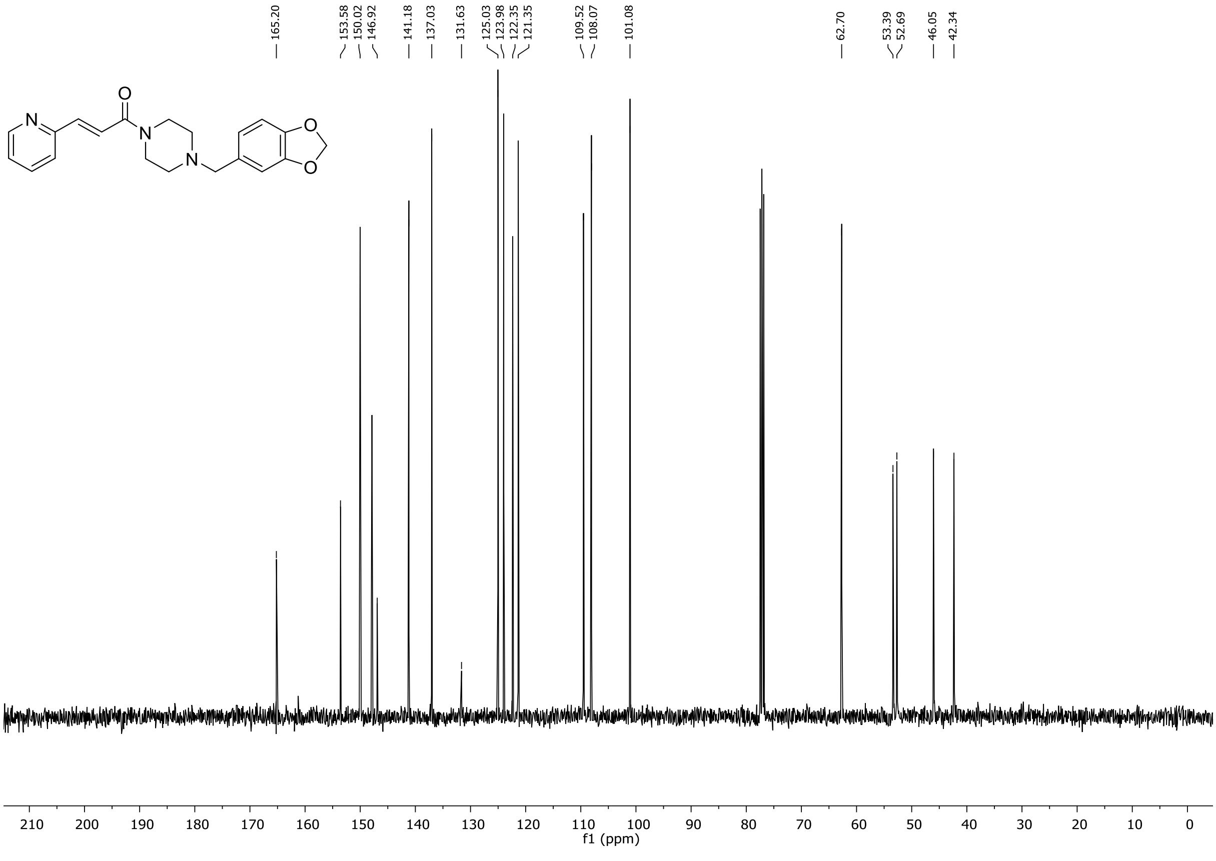

^{1}H-NMR of **APA-14** (400 MHz, $CDCl_3$)

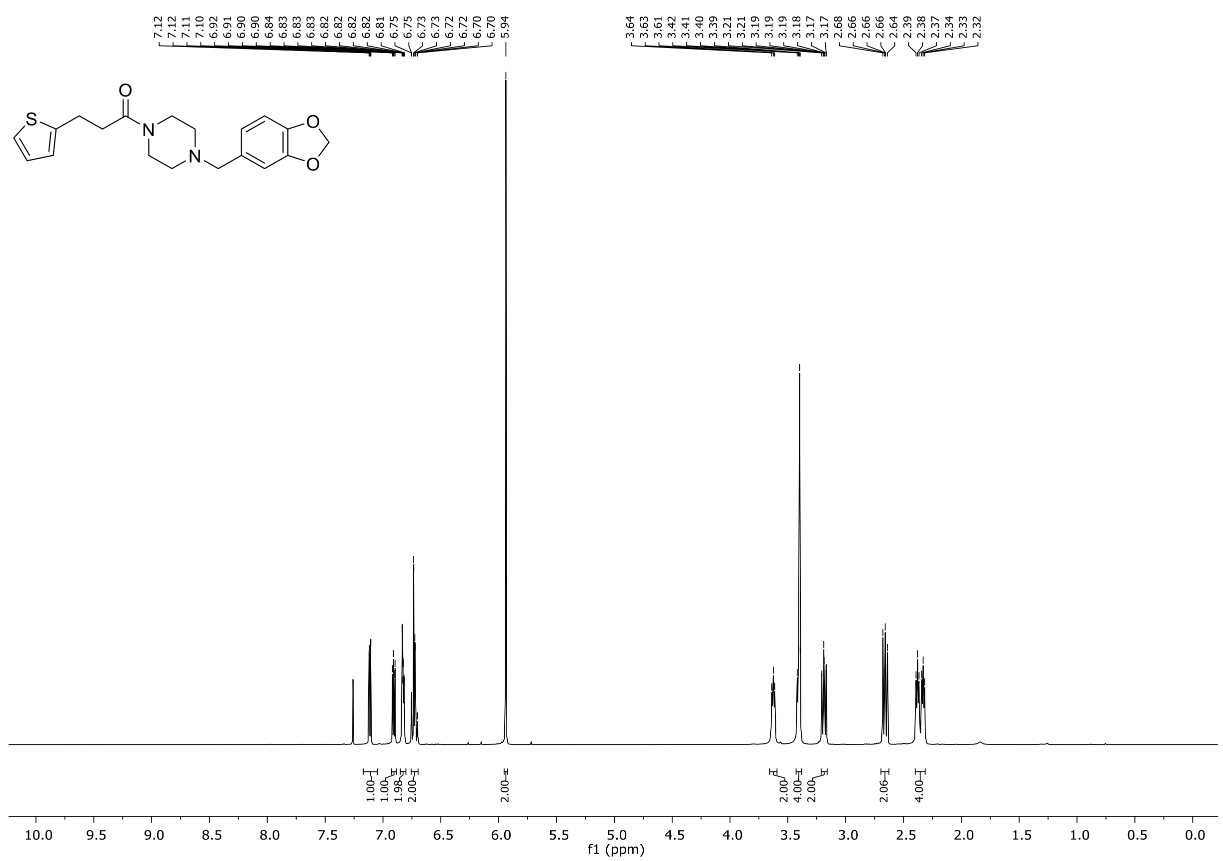

^{13}C-NMR of **APA-14** (101 MHz, $CDCl_3$)

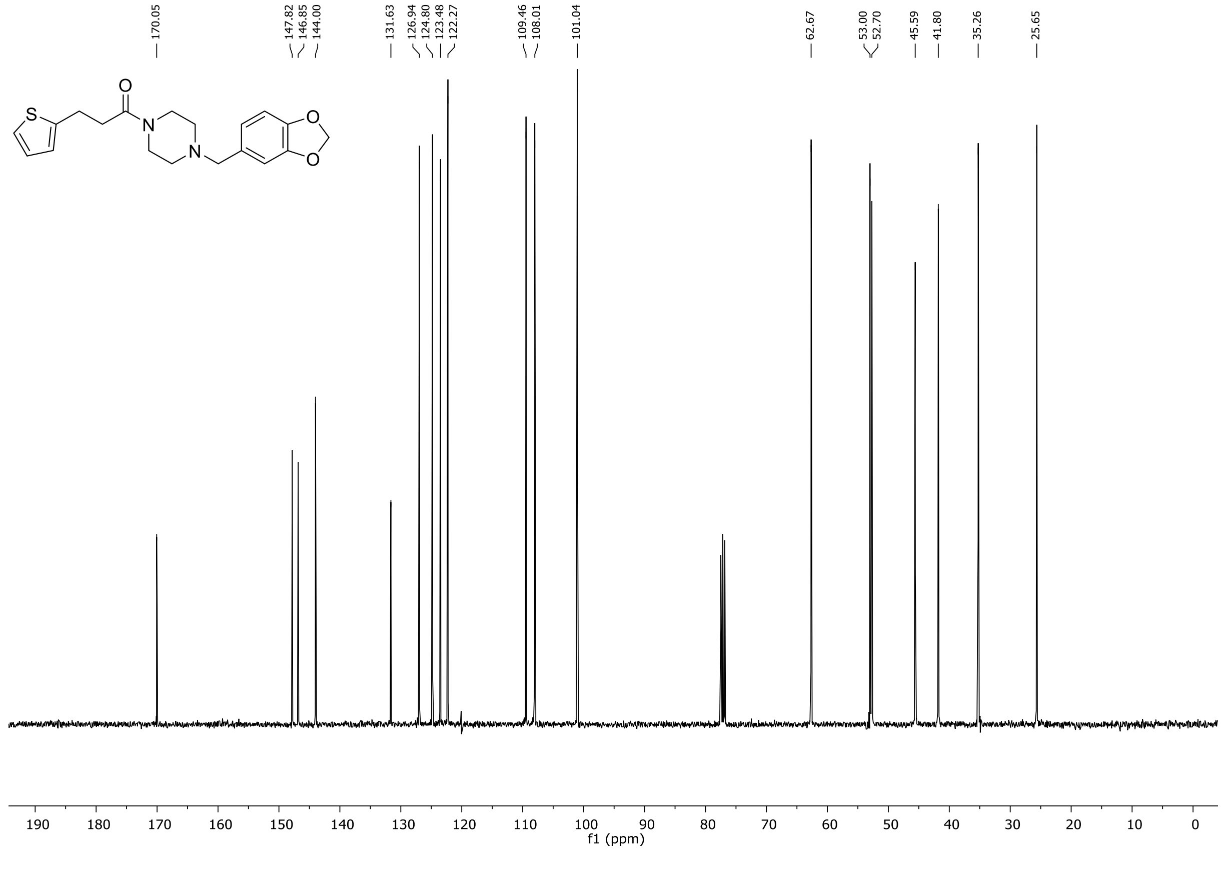

^{1}H-NMR of **I-4** (400 MHz, $CDCl_3$)

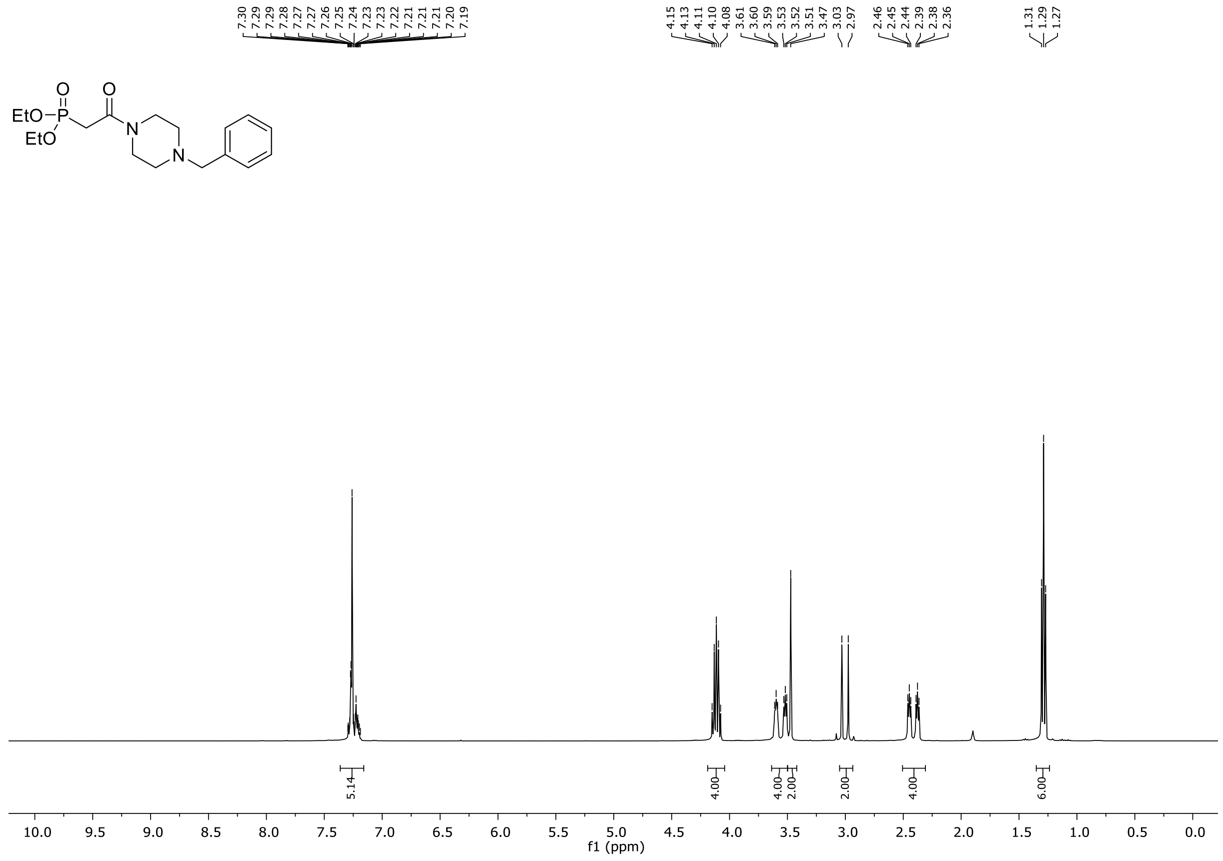

^{13}C-NMR of **I-4** (101 MHz, $CDCl_3$)

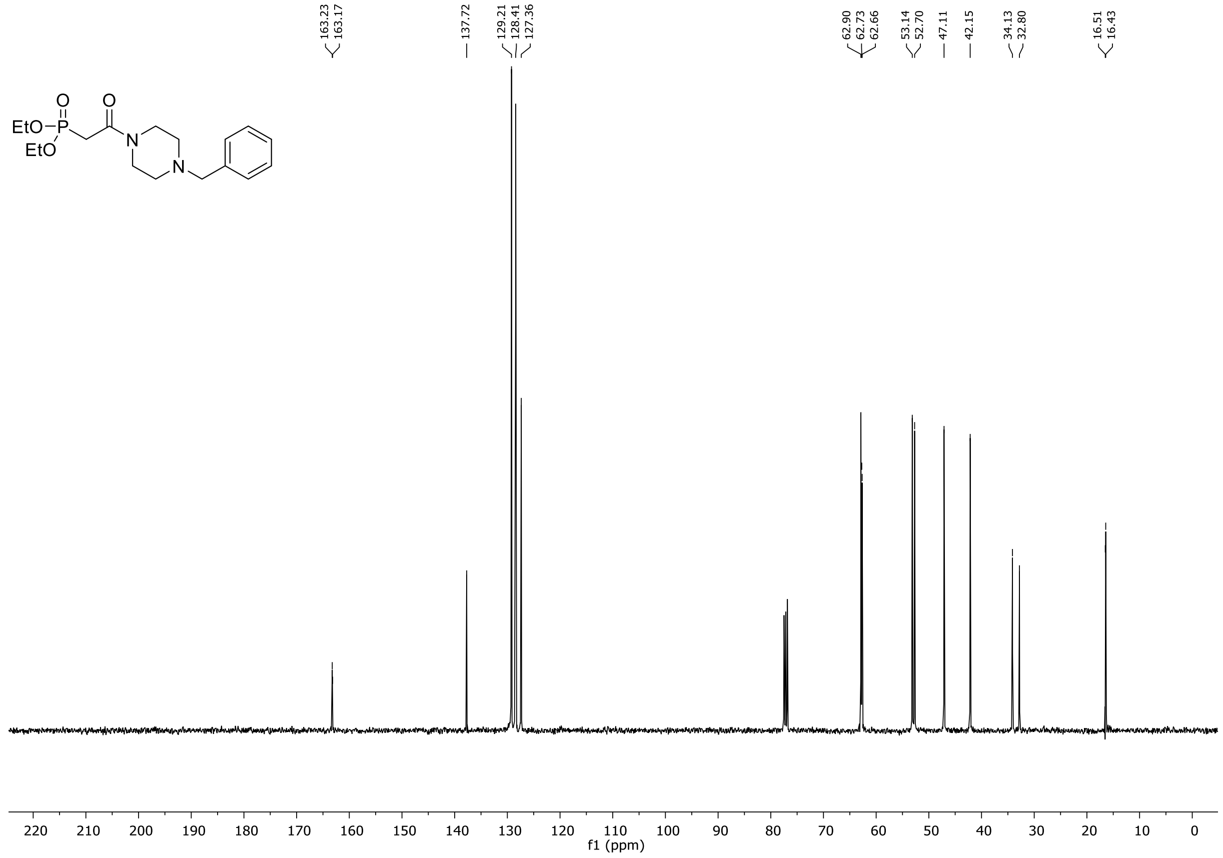

^{1}H-NMR of **APA-14** (400 MHz, $CDCl_3$)

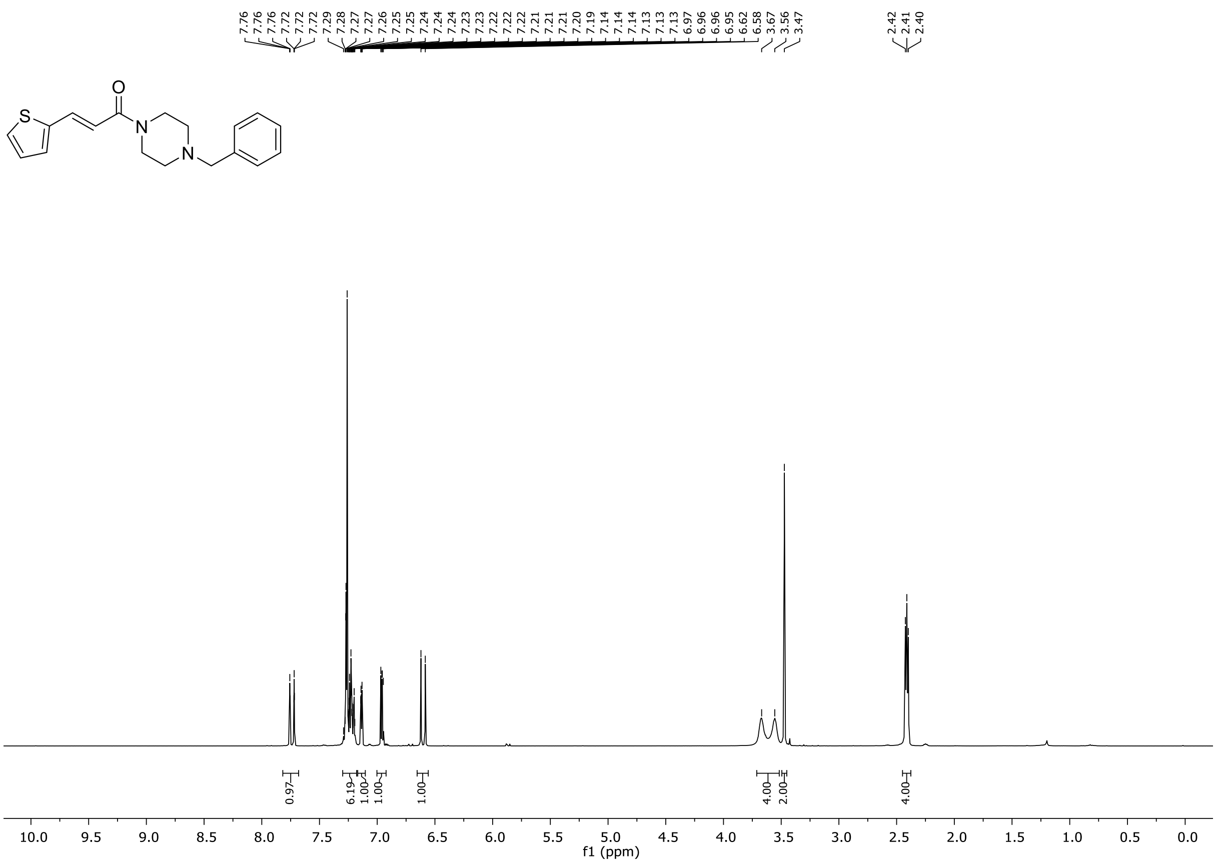

^{13}C-NMR of **APA-14** (101 MHz, $CDCl_3$)

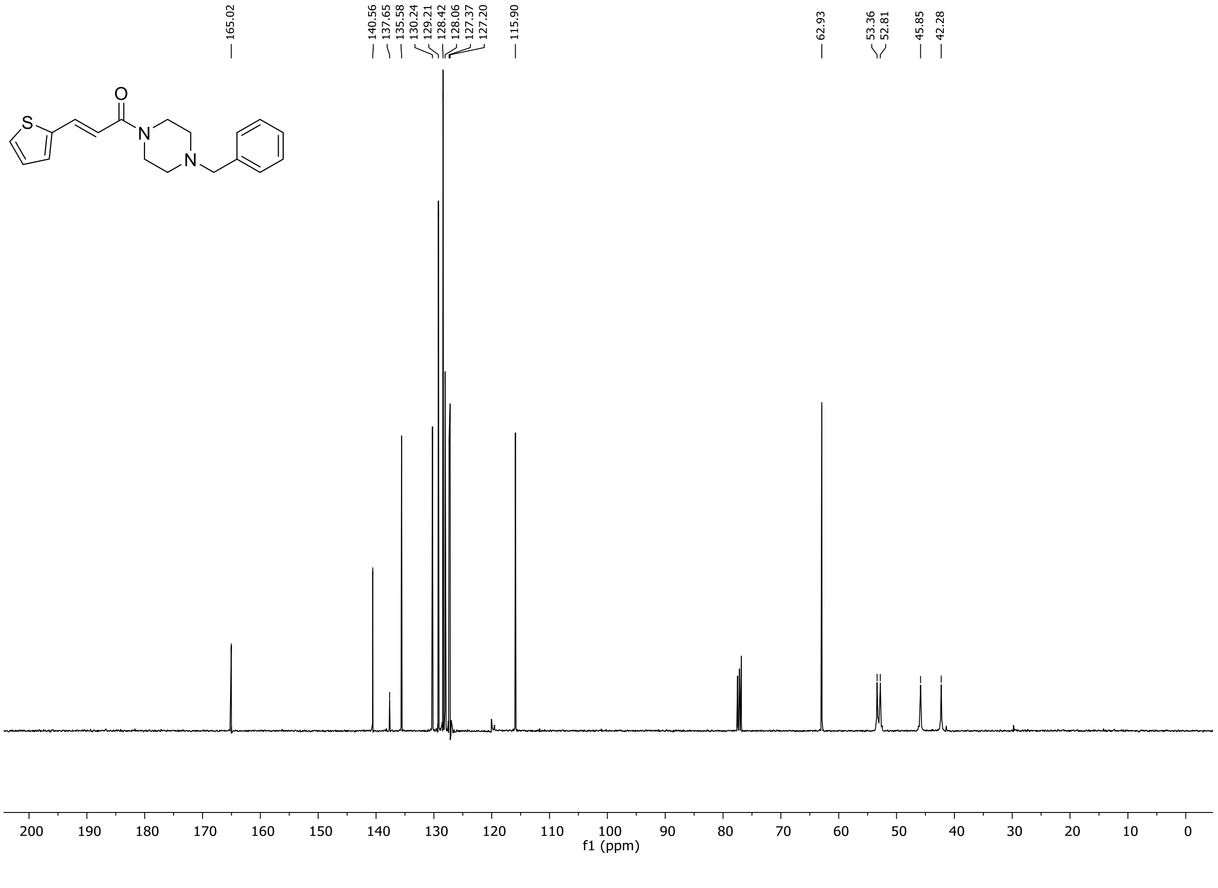

^{1}H-NMR of **I-7** (400 MHz, $CDCl_3$)

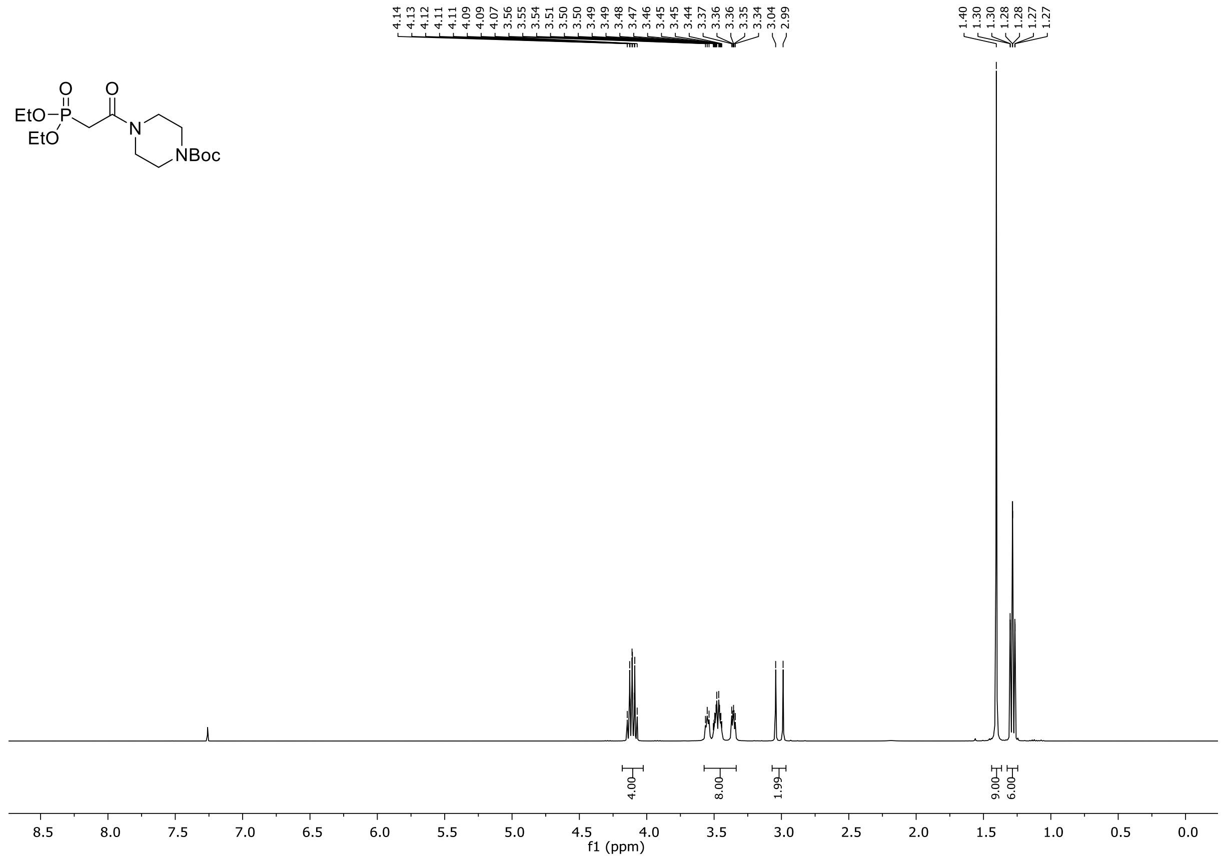

^{13}C-NMR of **I-7** (101 MHz, $CDCl_3$)

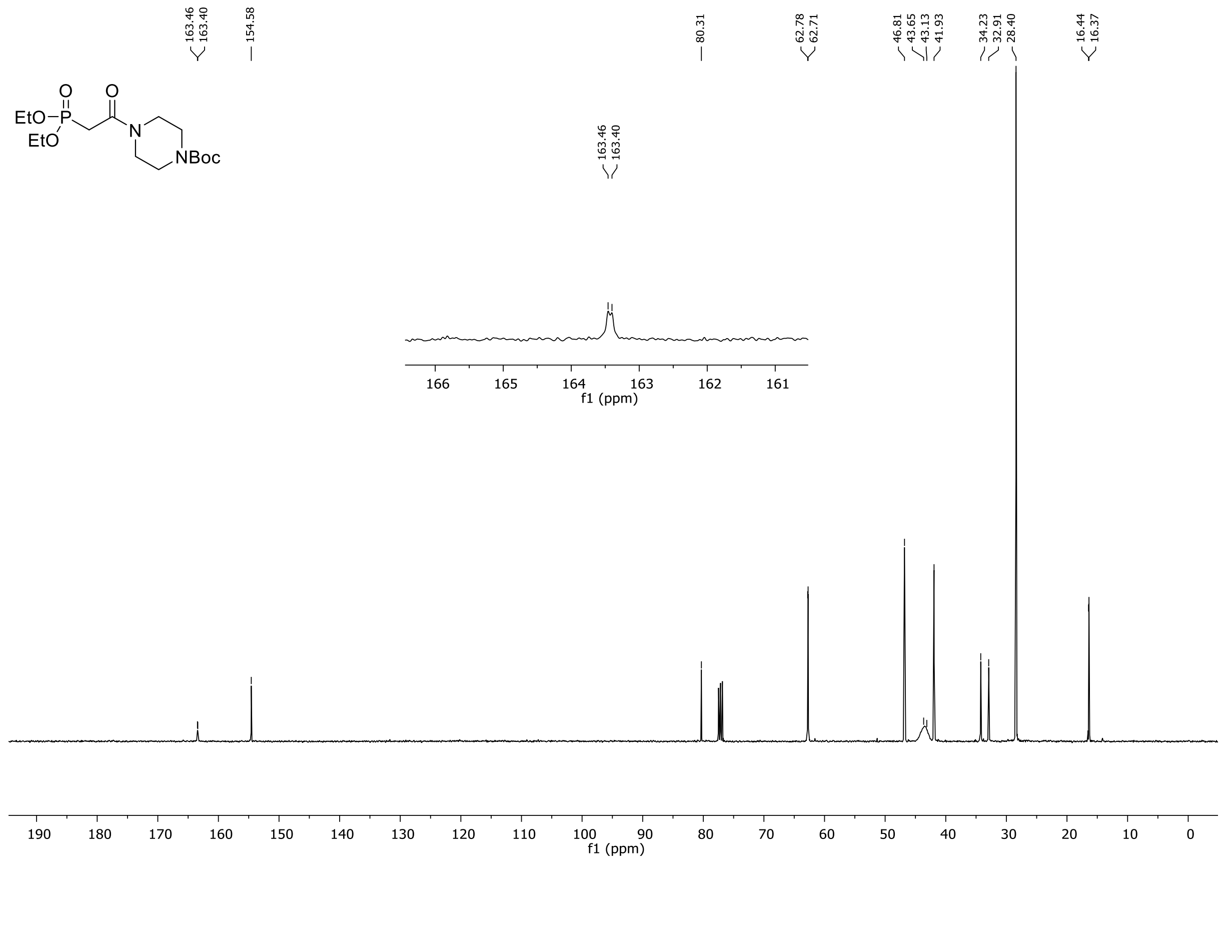

^{1}H-NMR of **I-8** (400 MHz, $CDCl_3$)

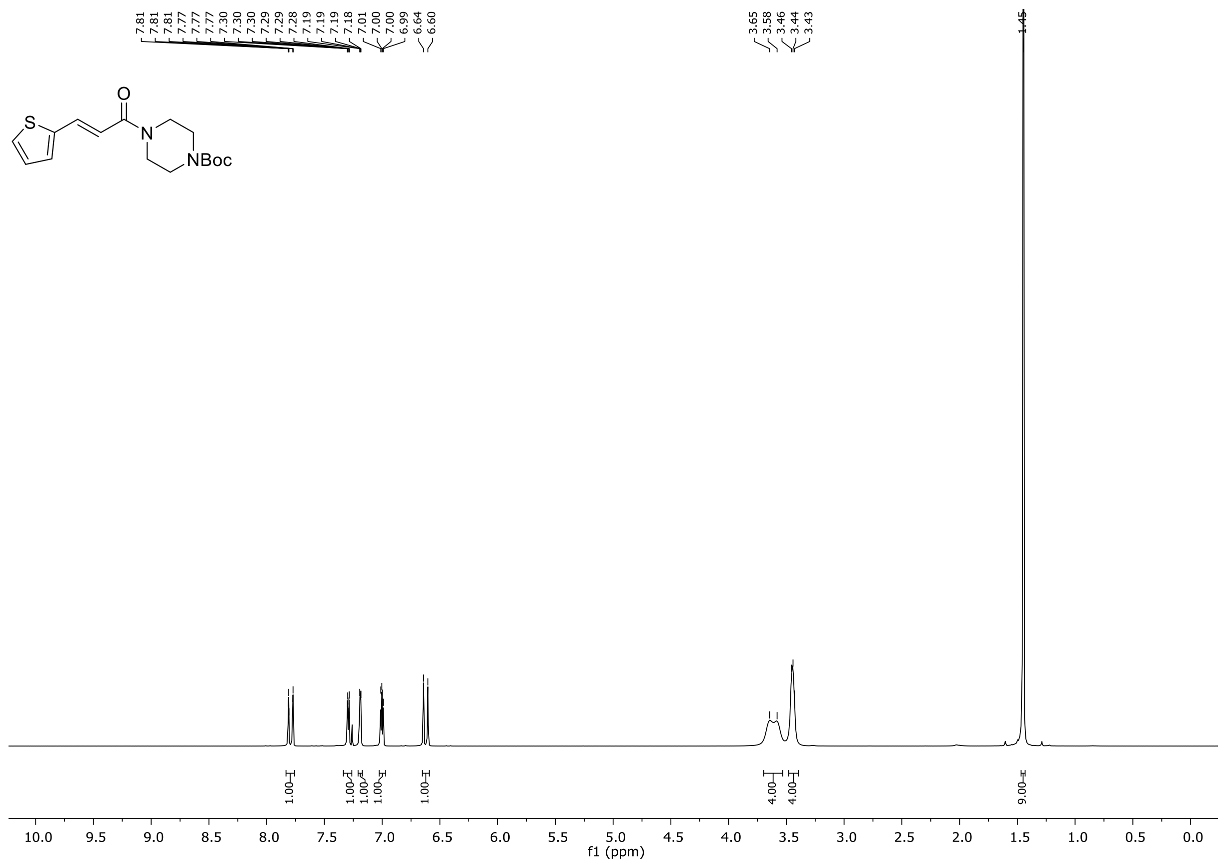

^{13}C-NMR of **I-8** (101 MHz, $CDCl_3$)

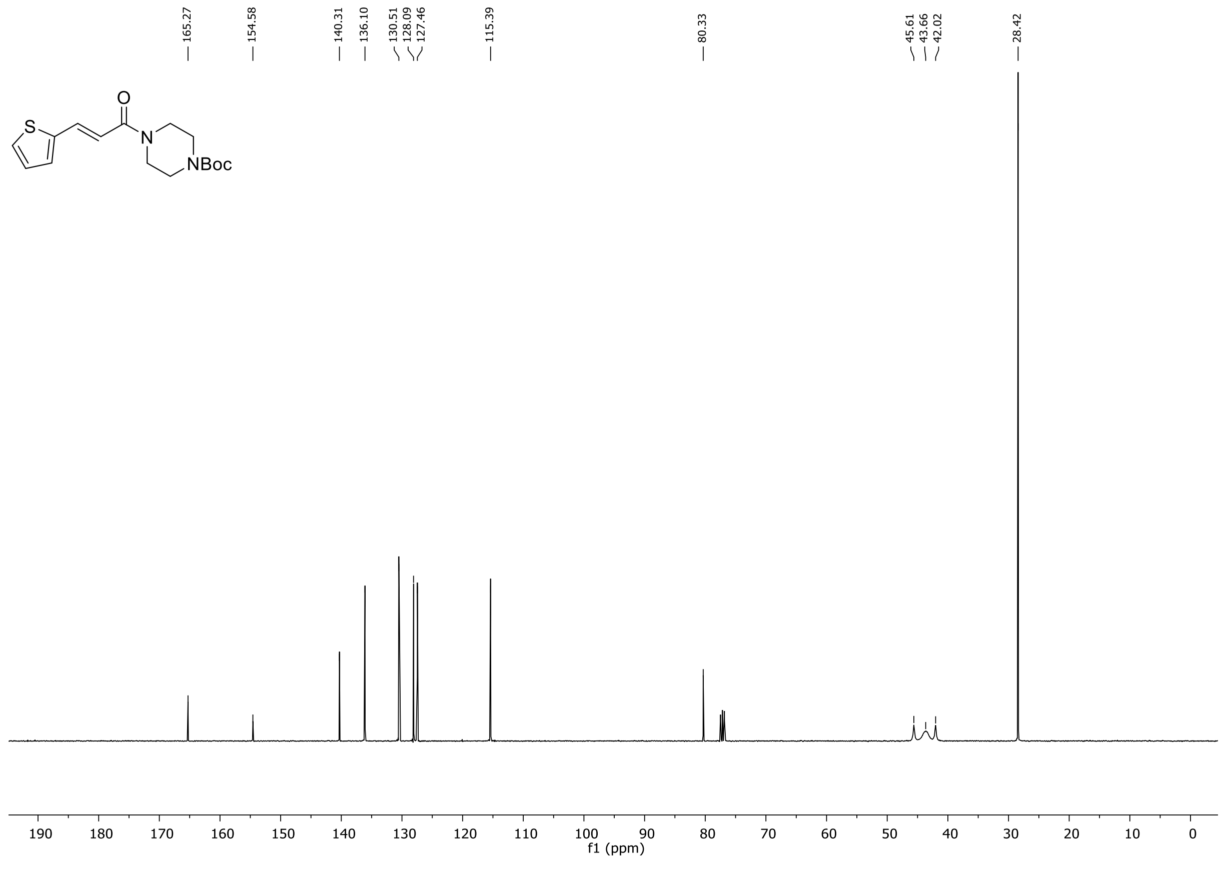

^{1}H-NMR of **I-9** (400 MHz, CD_3OD)

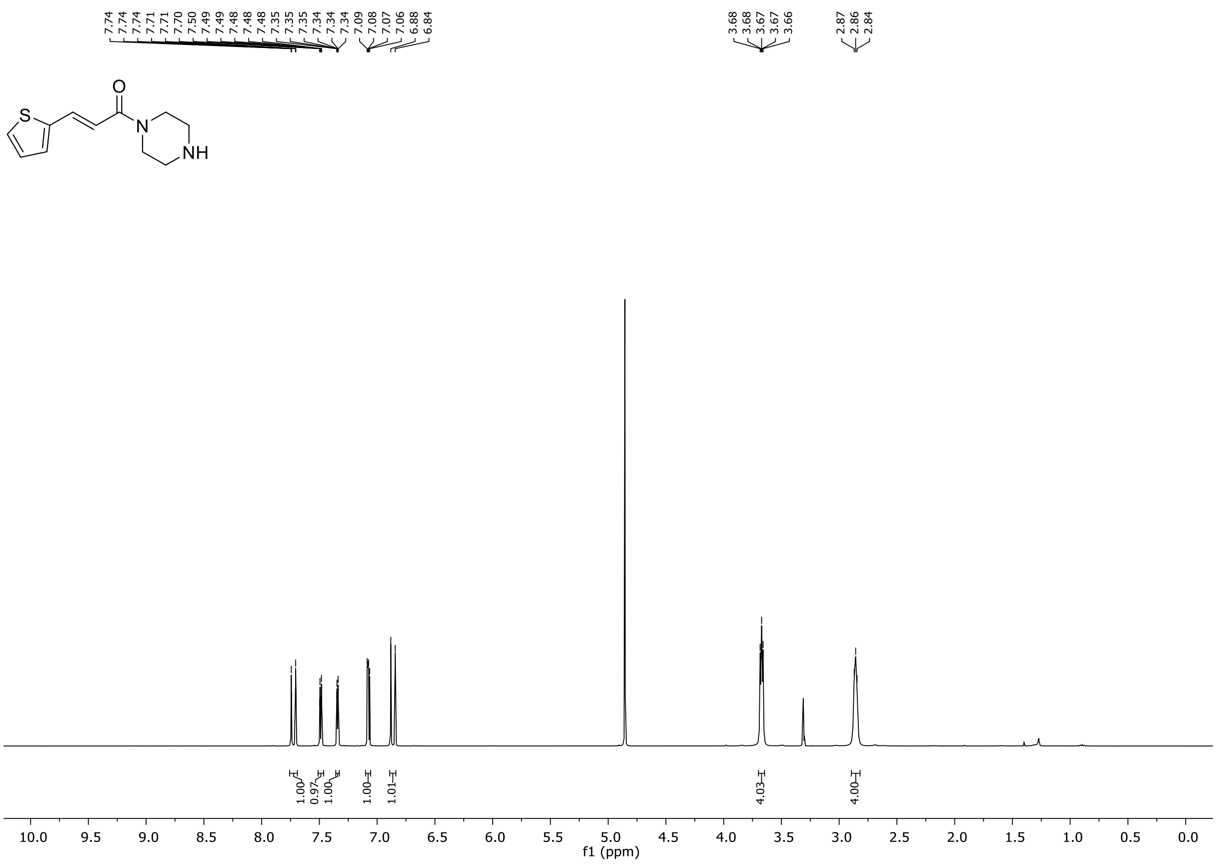

^{13}C-NMR of **I-9** (101 MHz, CD_3OD)

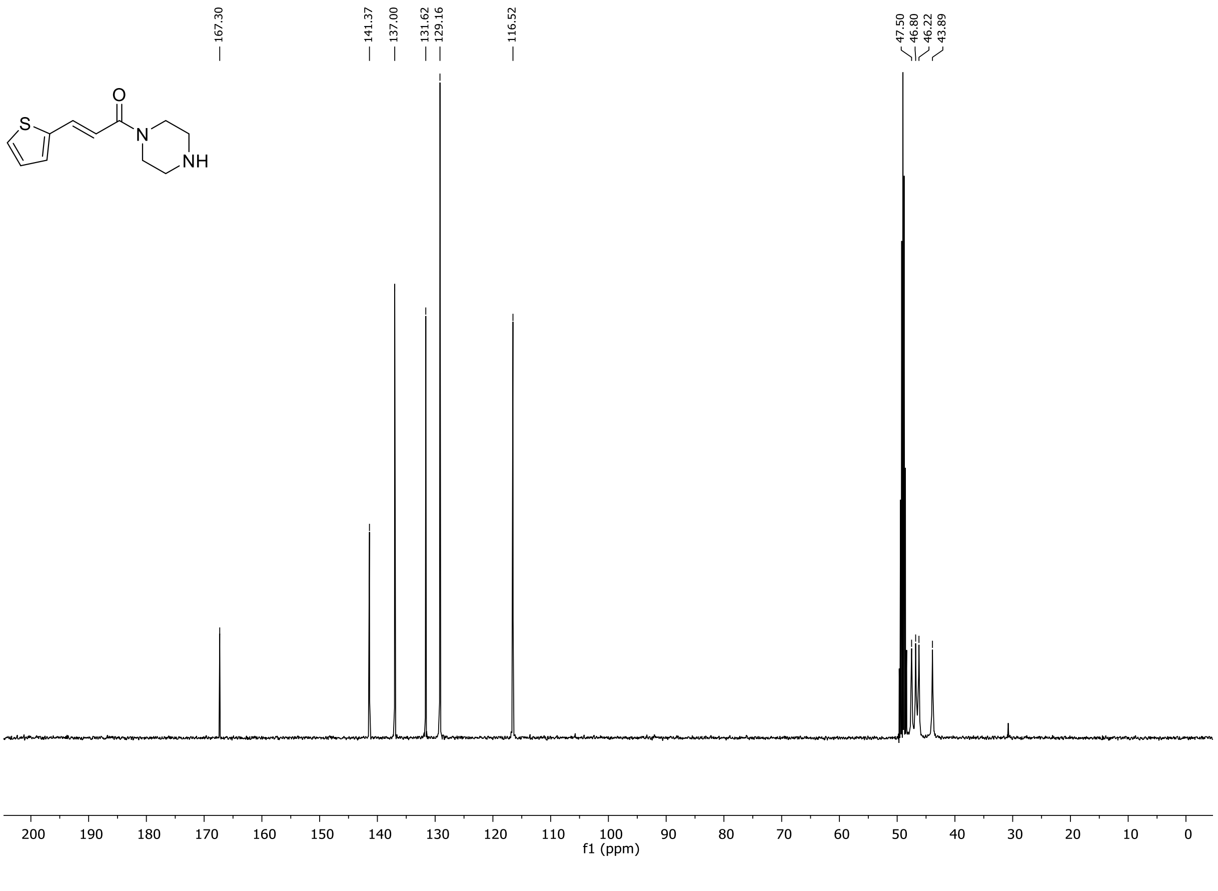

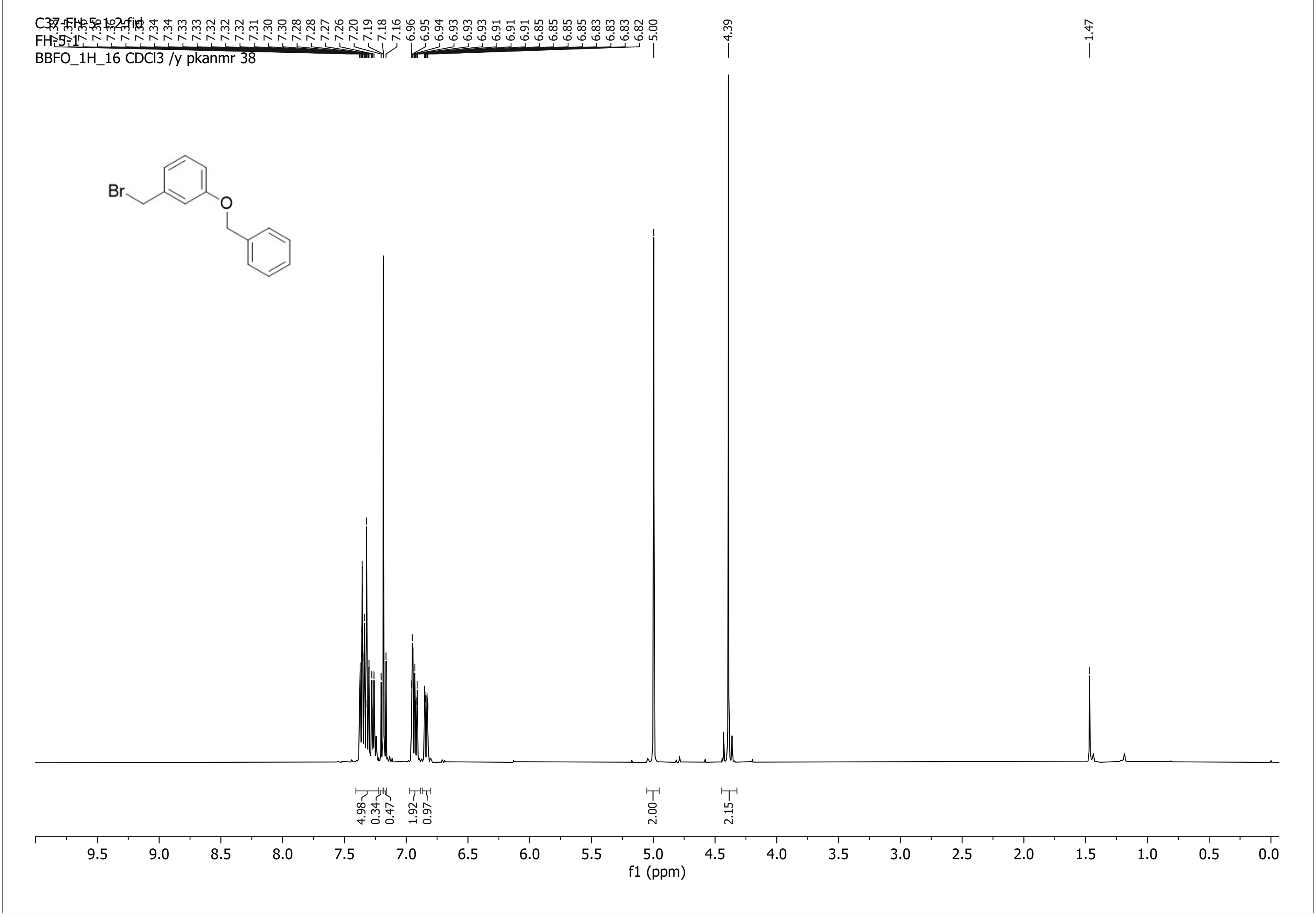
C37-FH-5-1-2.fid
FH-5-1
BBFO_1H_16 CDCl3 /y pkanmr 38
7.34
7.34
7.33
7.33
7.32
7.32
7.32
7.31
7.30
7.30
7.28
7.28
7.27
7.26
7.20
7.19
7.18
7.16
6.96
6.95
6.94
6.93
6.93
6.93
6.91
6.91
6.91
6.85
6.85
6.85
6.85
6.83
6.83
6.83
6.83
6.82
5.00
4.39
1.47
Br
O
4.98
0.34
0.47
1.92
0.97
2.00
2.15
9.5
9.0
8.5
8.0
7.5
7.0
6.5
6.0
5.5
5.0
4.5
4.0
3.5
3.0
2.5
2.0
1.5
1.0
0.5
0.0
f1 (ppm)

^{1}H-NMR of **APA-15** (400 MHz, CDCl3)

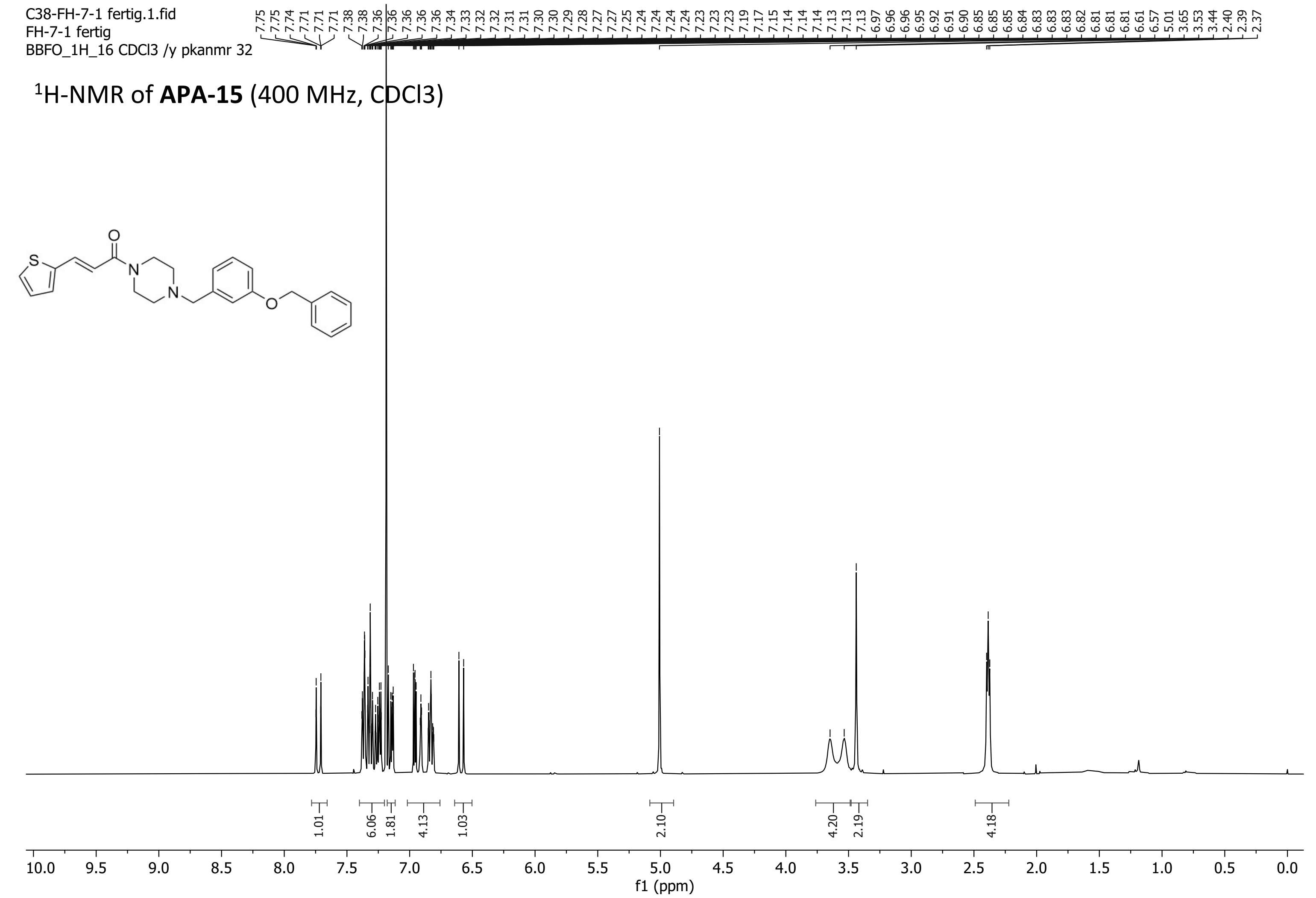

^{13}C-NMR of **APA-15** (101 MHz, $CDCl_3$)

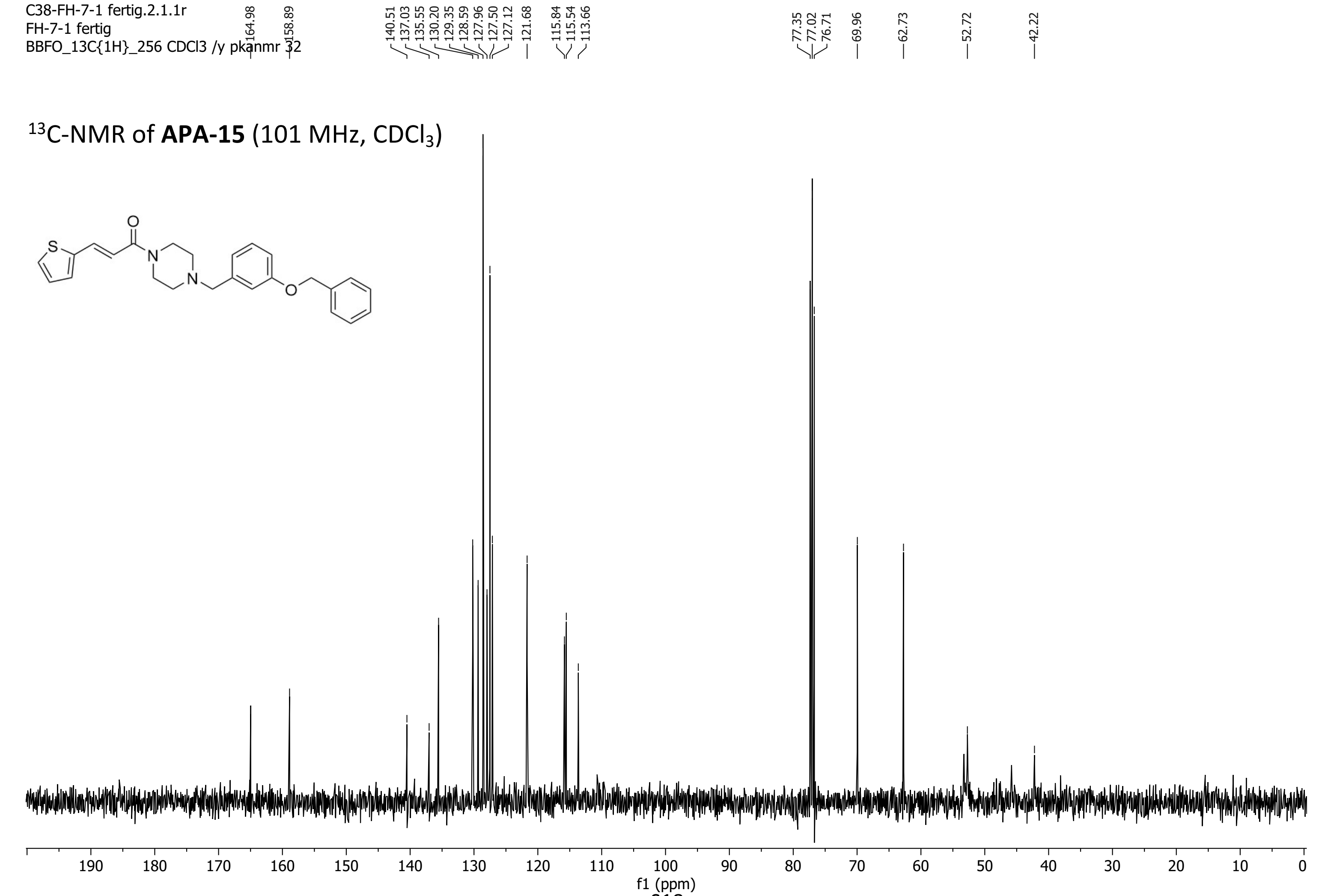

^{1}H-NMR of **I-11** (400 MHz, $CDCl_3$)

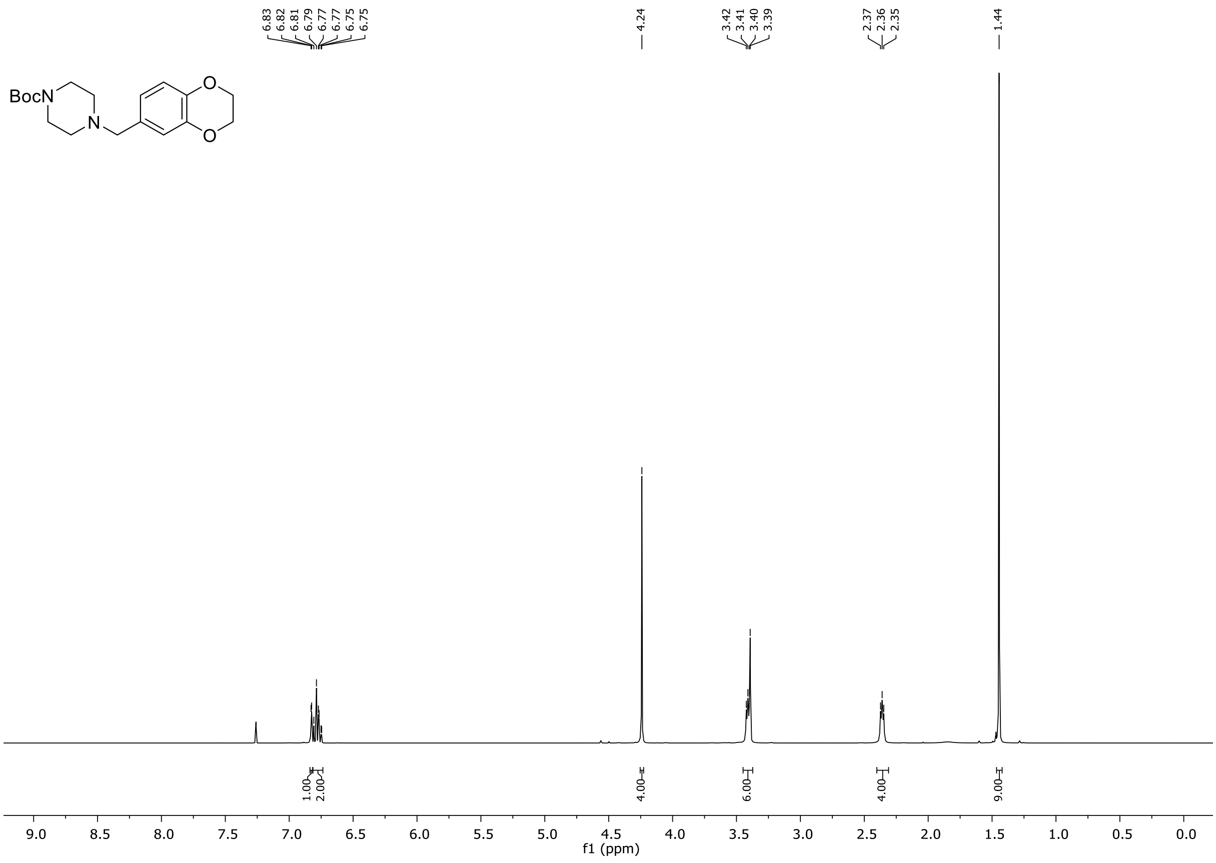

^{13}C-NMR of **I-11** (101 MHz, $CDCl_3$)

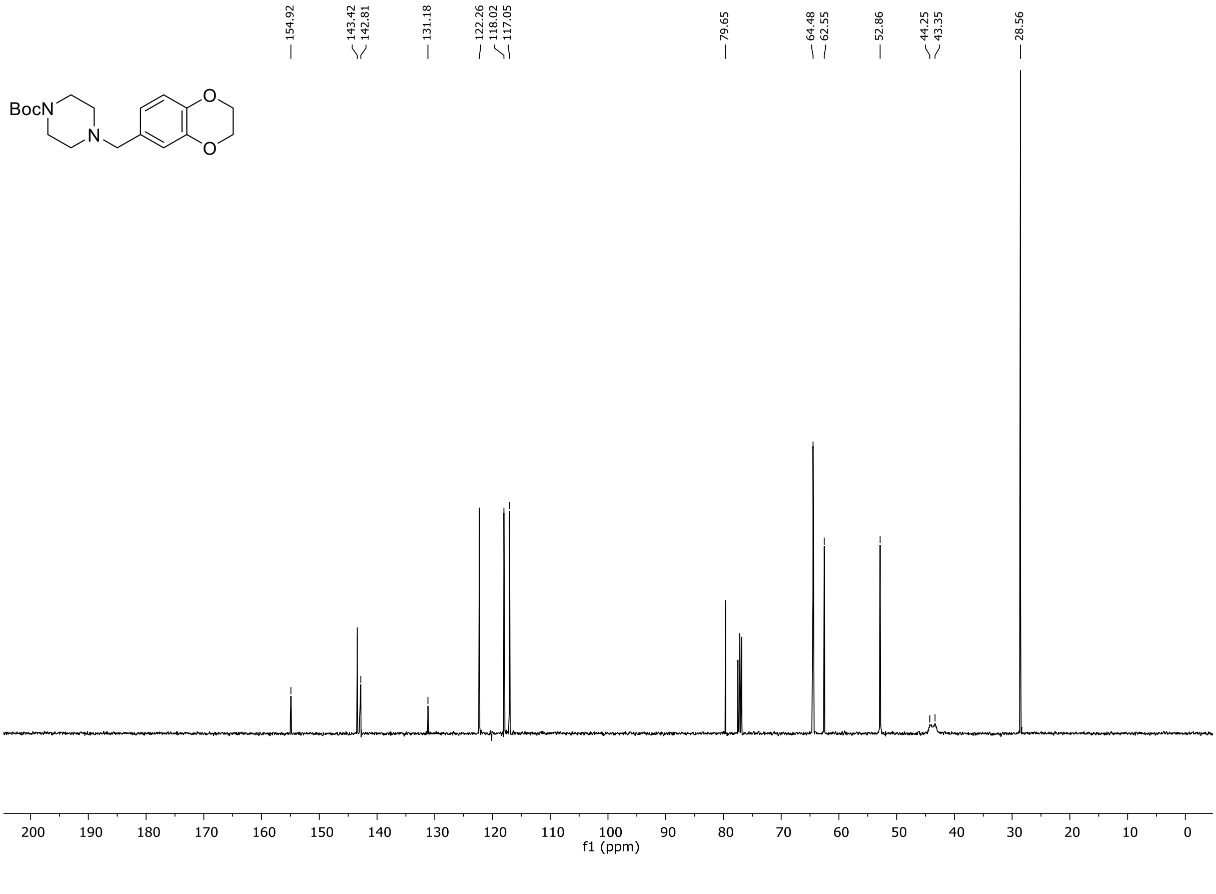

^{1}H-NMR of **I-12** (400 MHz, $CDCl_3$)

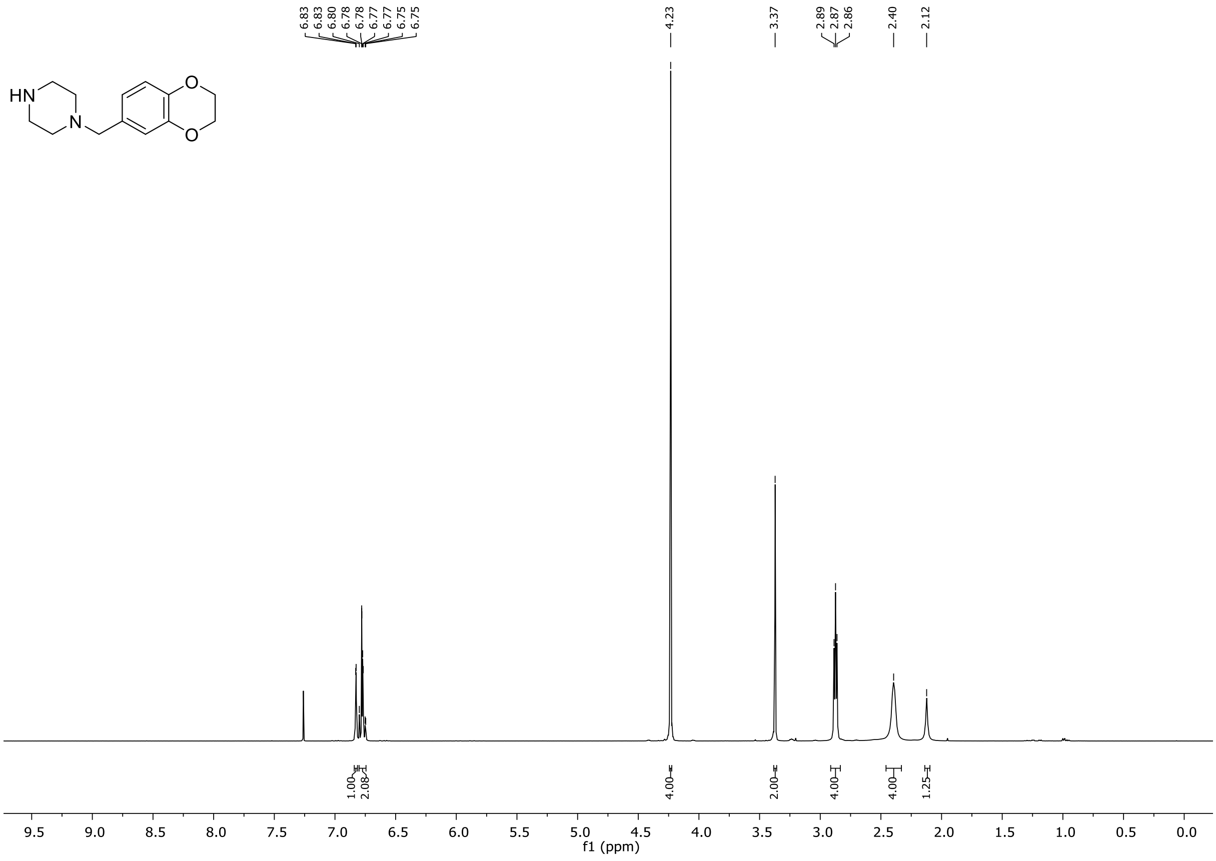

^{13}C-NMR of **I-12** (101 MHz, $CDCl_3$)

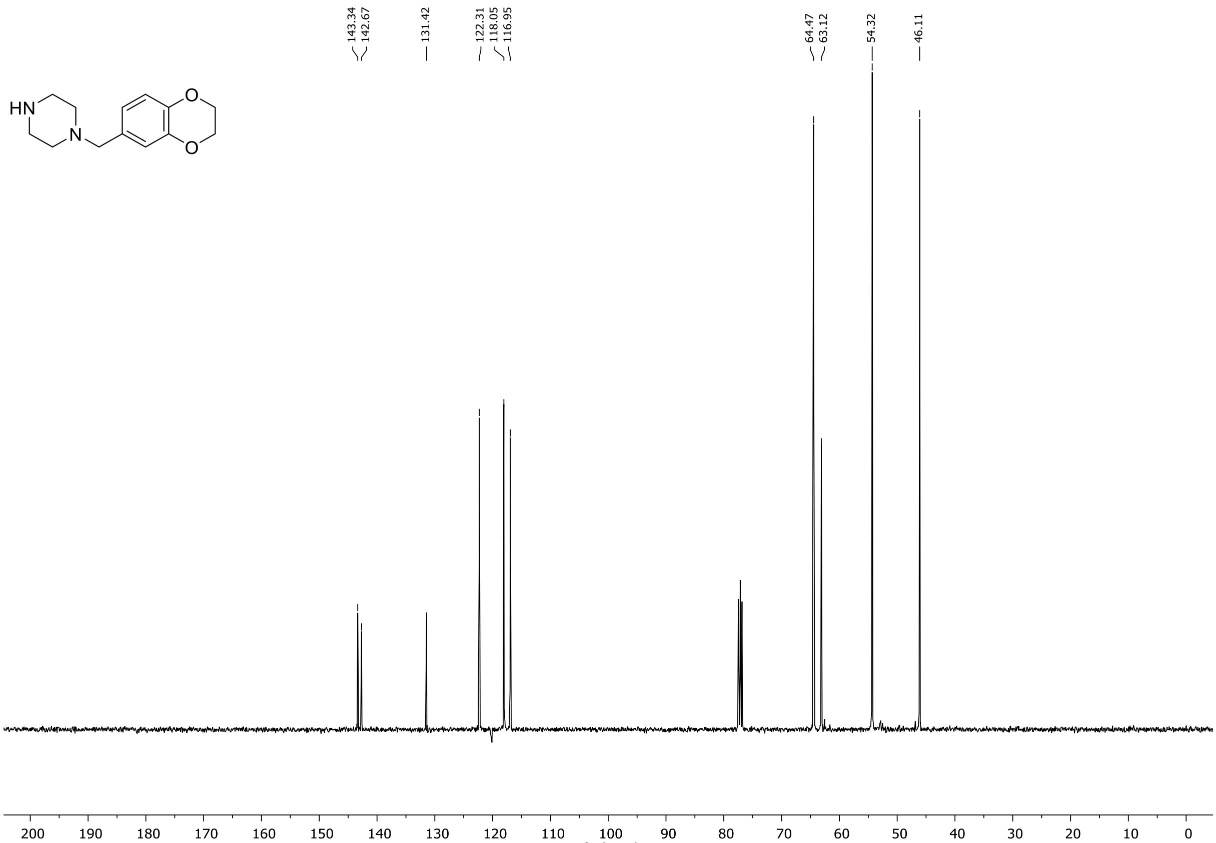

^{1}H-NMR of **I-13** (400 MHz, $CDCl_3$)

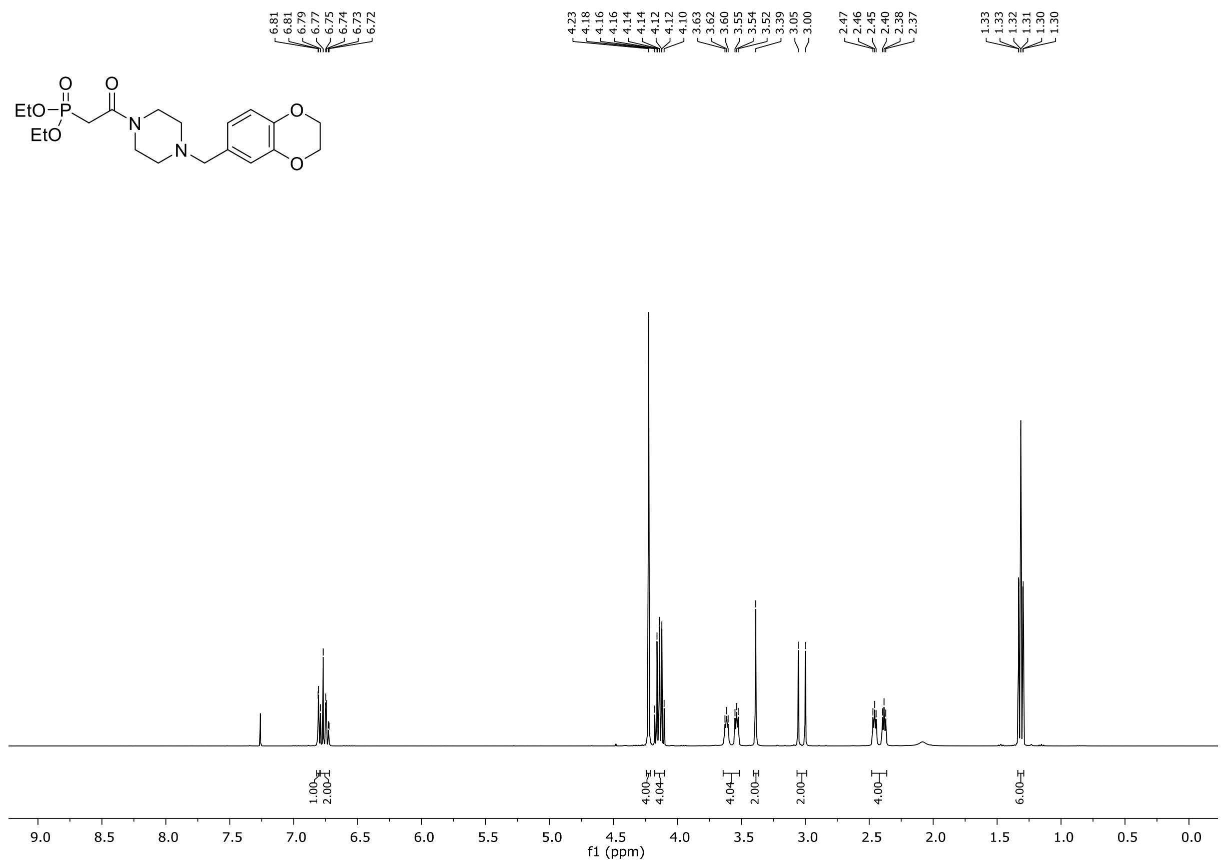

^{13}C-NMR of **I-13** (101 MHz, $CDCl_3$)

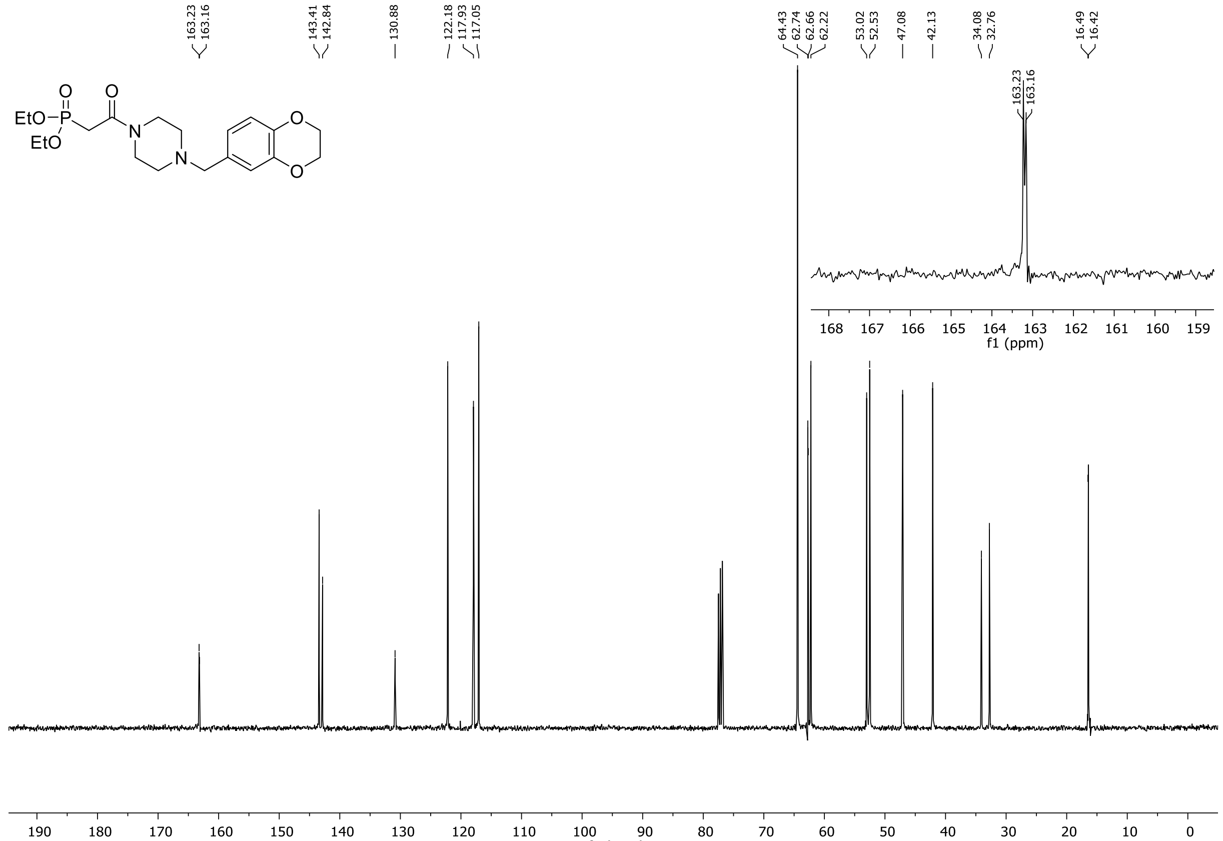

^{1}H-NMR of **APA-17** (400 MHz, $CDCl_3$

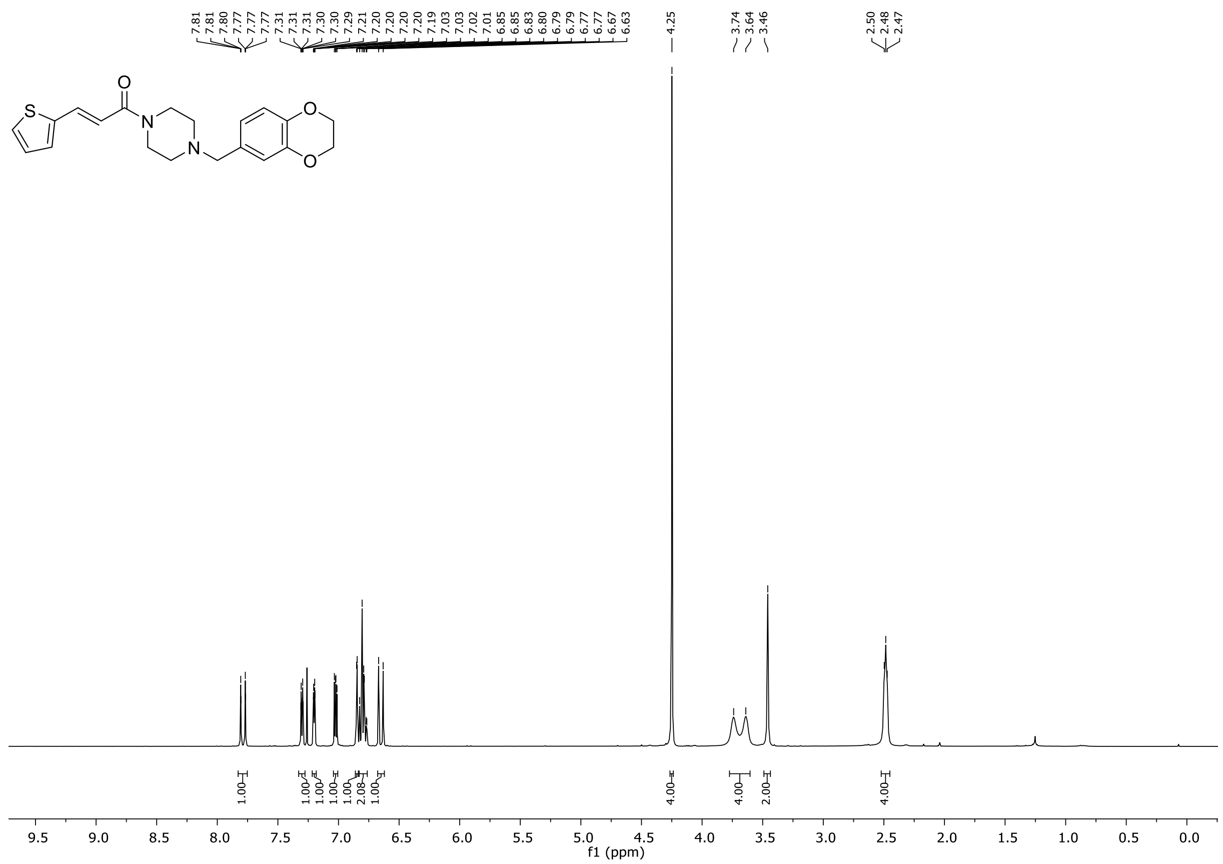

^{13}C-NMR of **APA-17** (400 MHz, $CDCl_3$

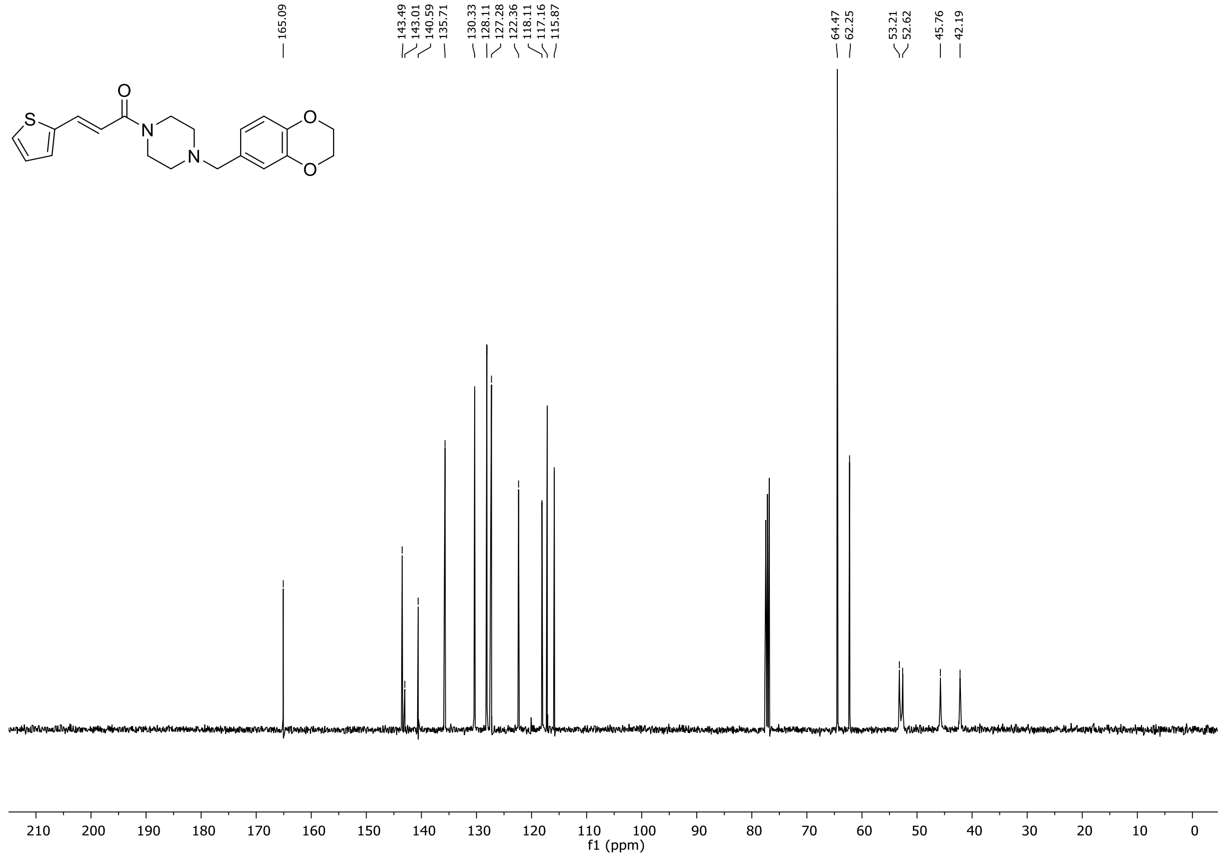

^{1}H-NMR of **APA-18** (400 MHz, $CDCl_3$)

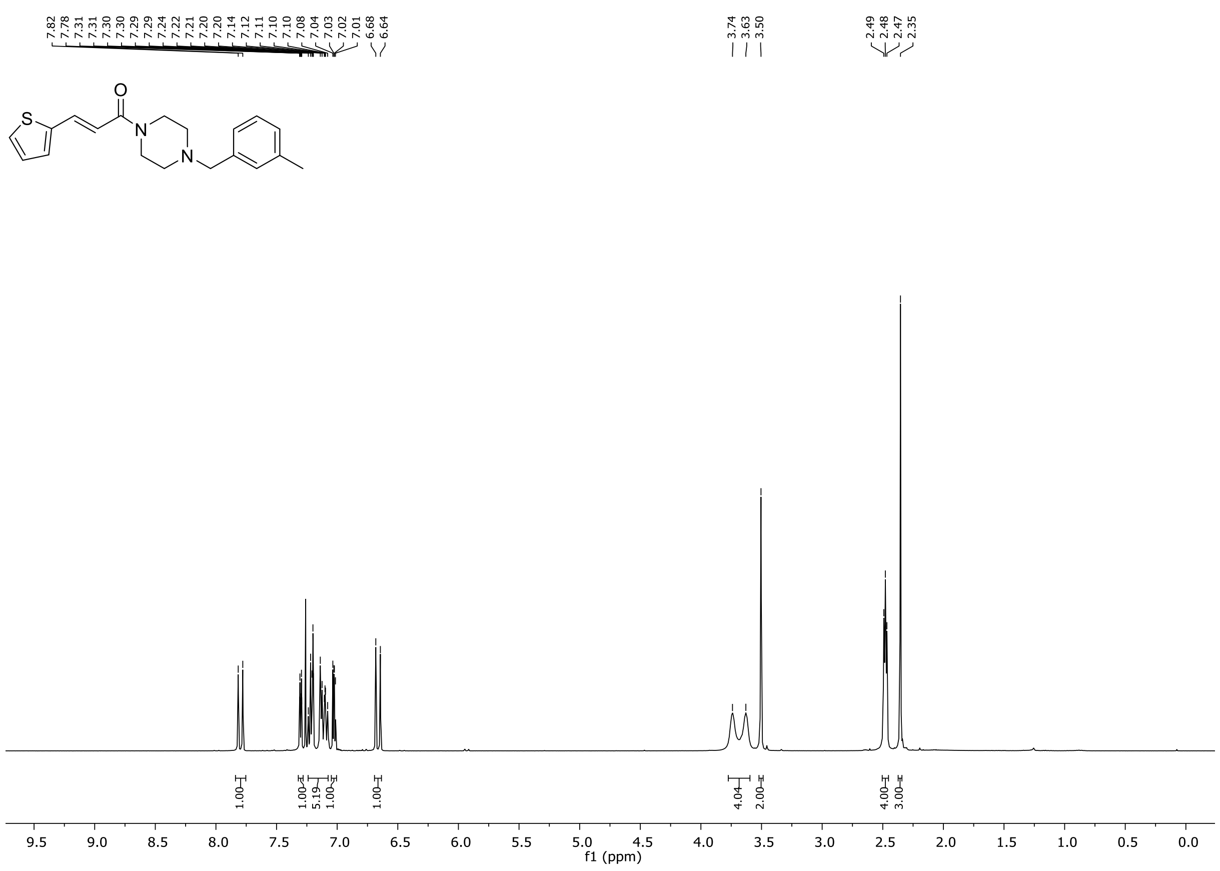

^{13}C-NMR of **APA-18** (101 MHz, $CDCl_3$)

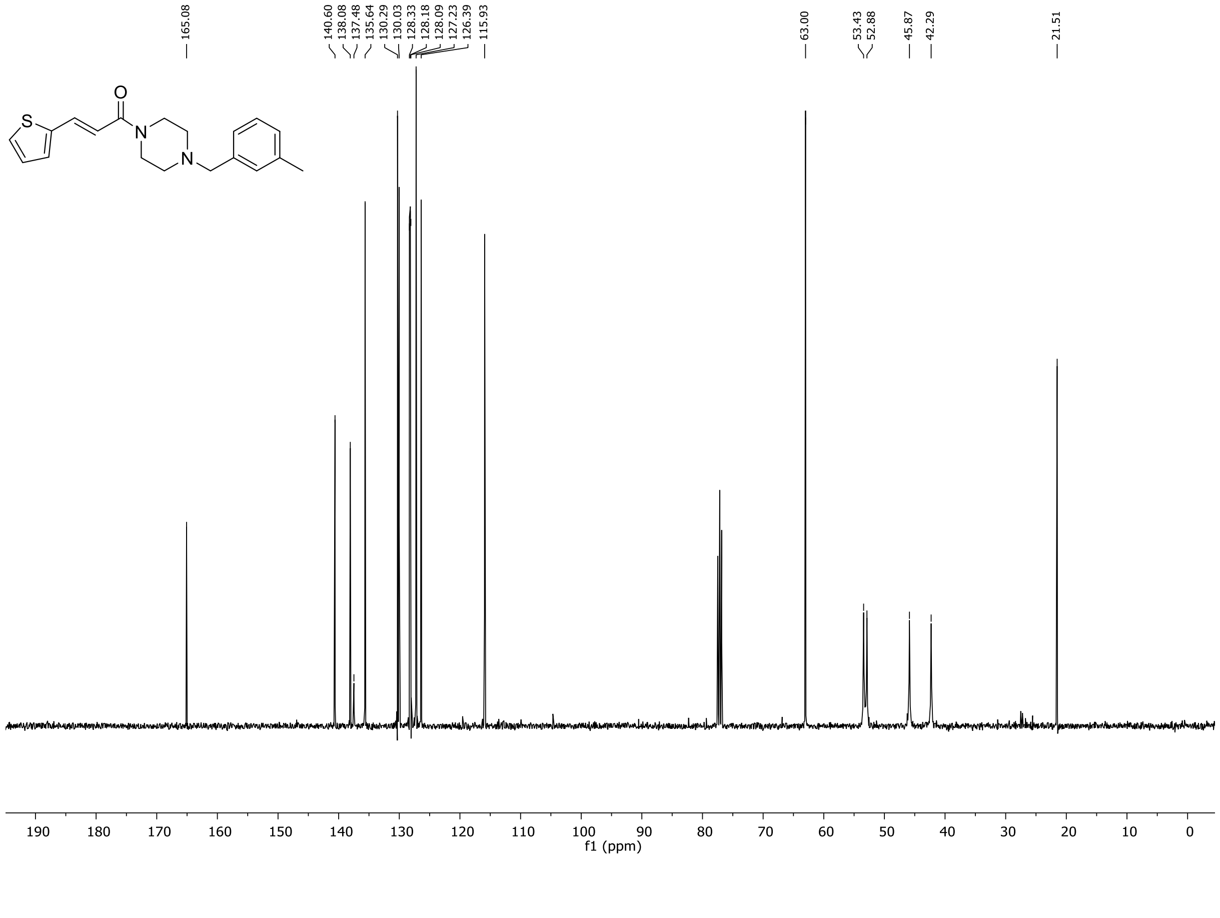

^{1}H-NMR of **APA-19** (400 MHz, $CDCl_3$)

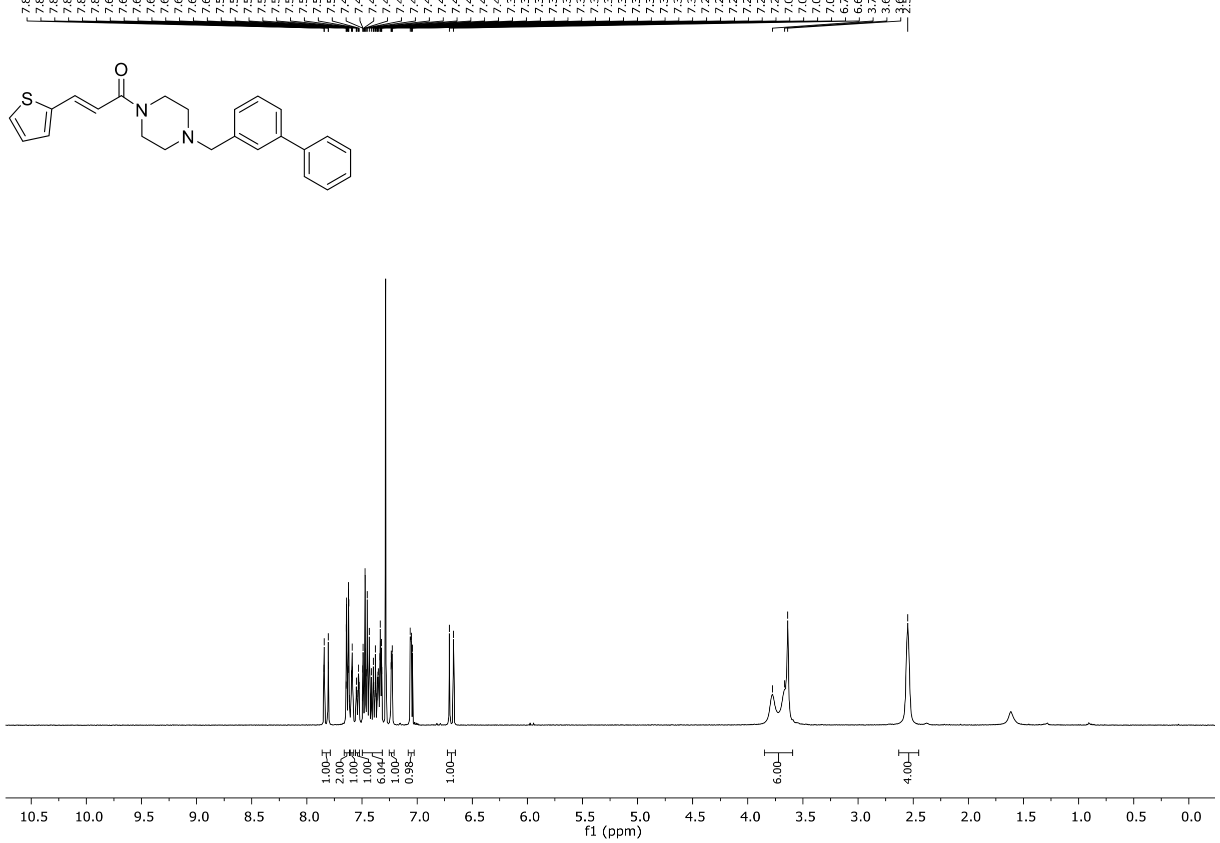

^{13}C-NMR of **APA-19** (101 MHz, $CDCl_3$)

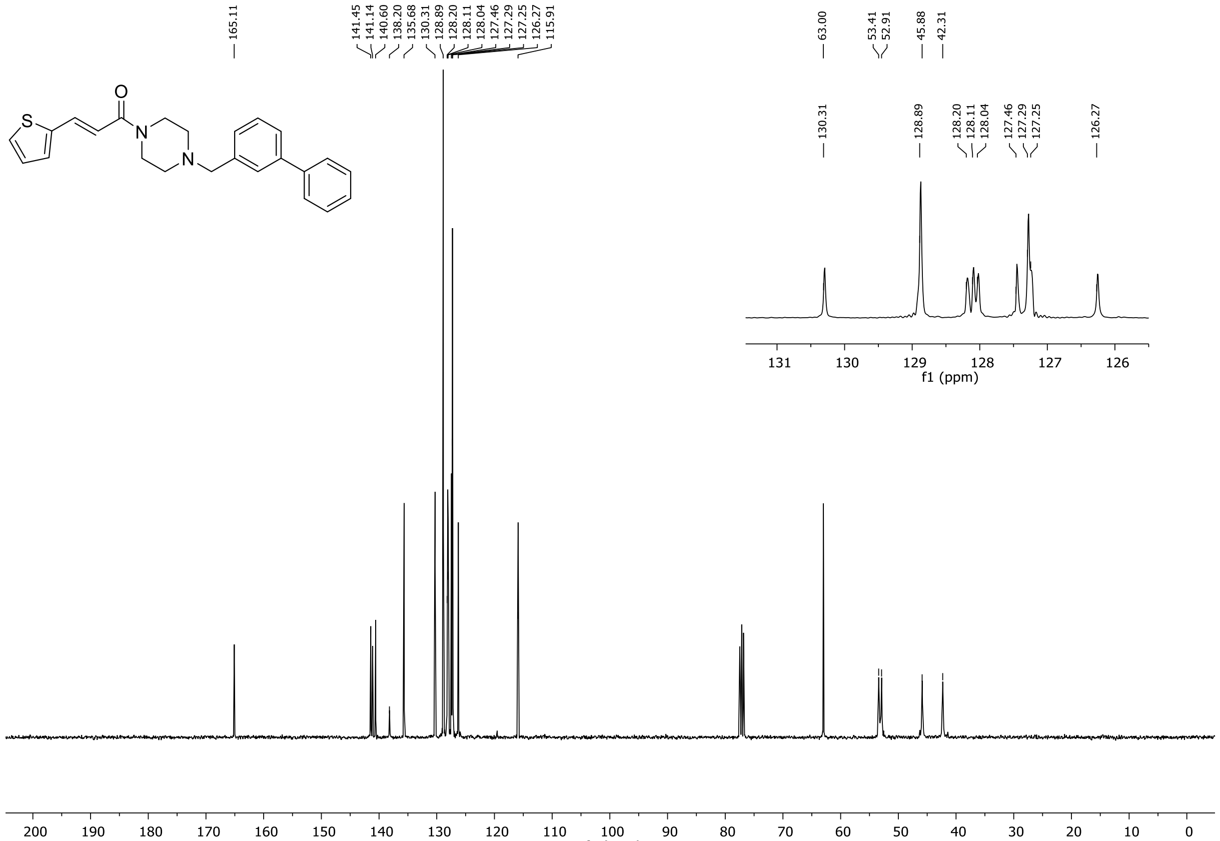

^{1}H-NMR of **APA-20** (400 MHz, $CDCl_3$)

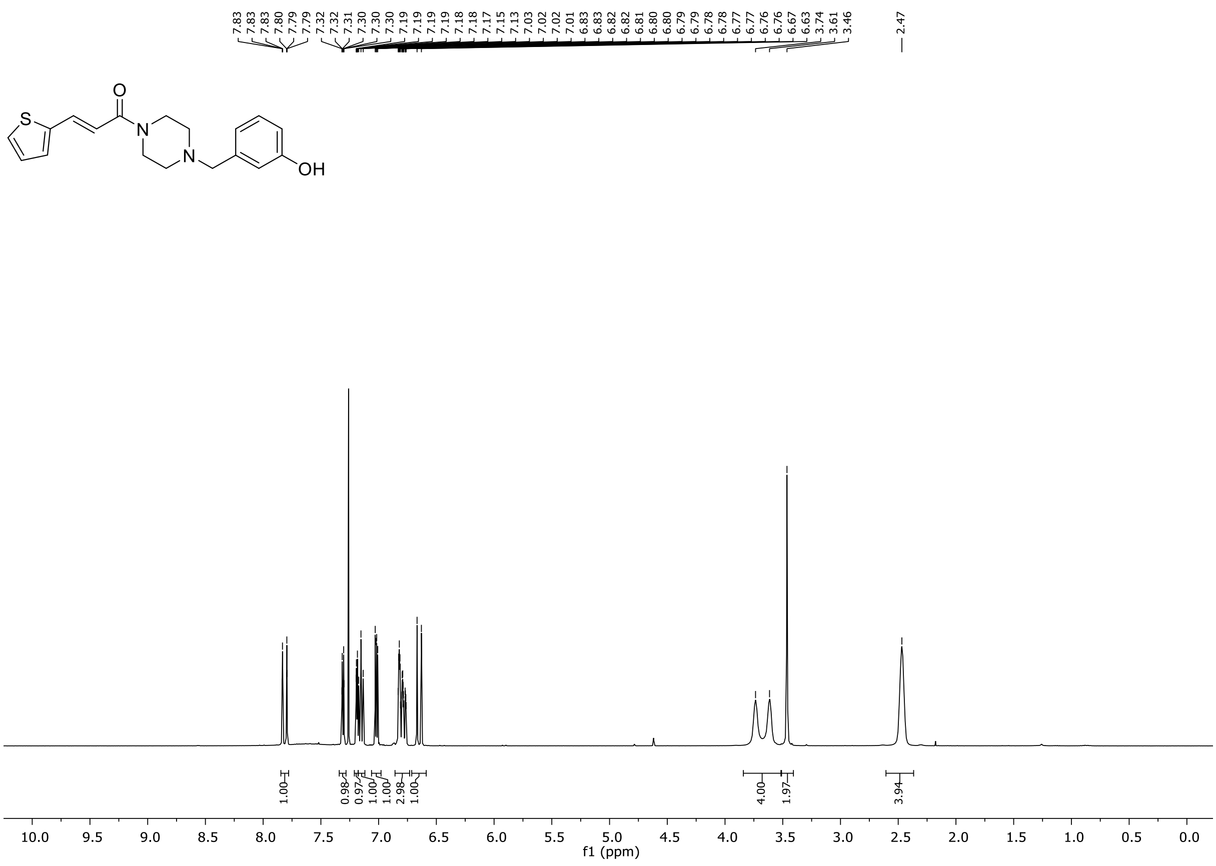

^{13}C-NMR of **APA-20** (101 MHz, $CDCl_3$)

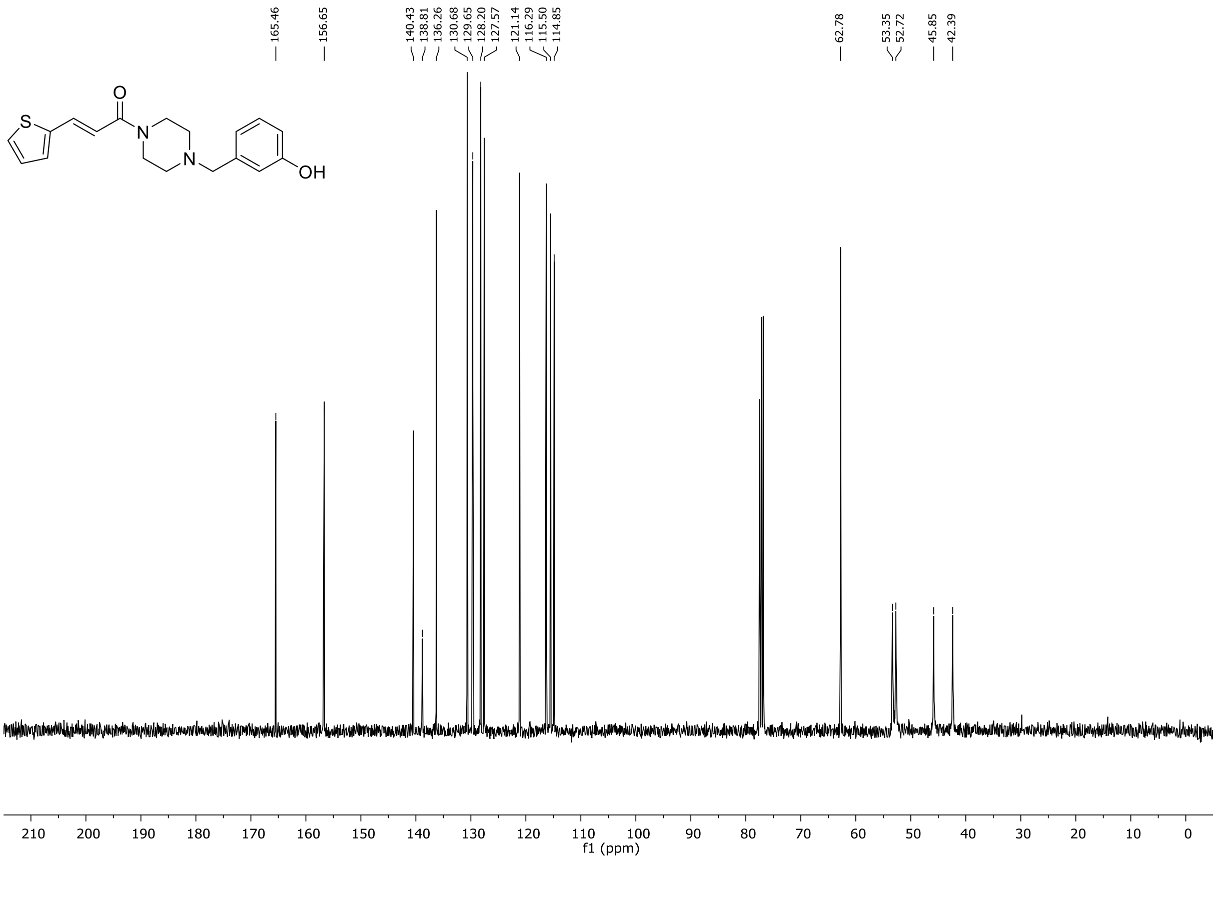

^{1}H-NMR of **APA-21** (400 MHz, $CDCl_3$)

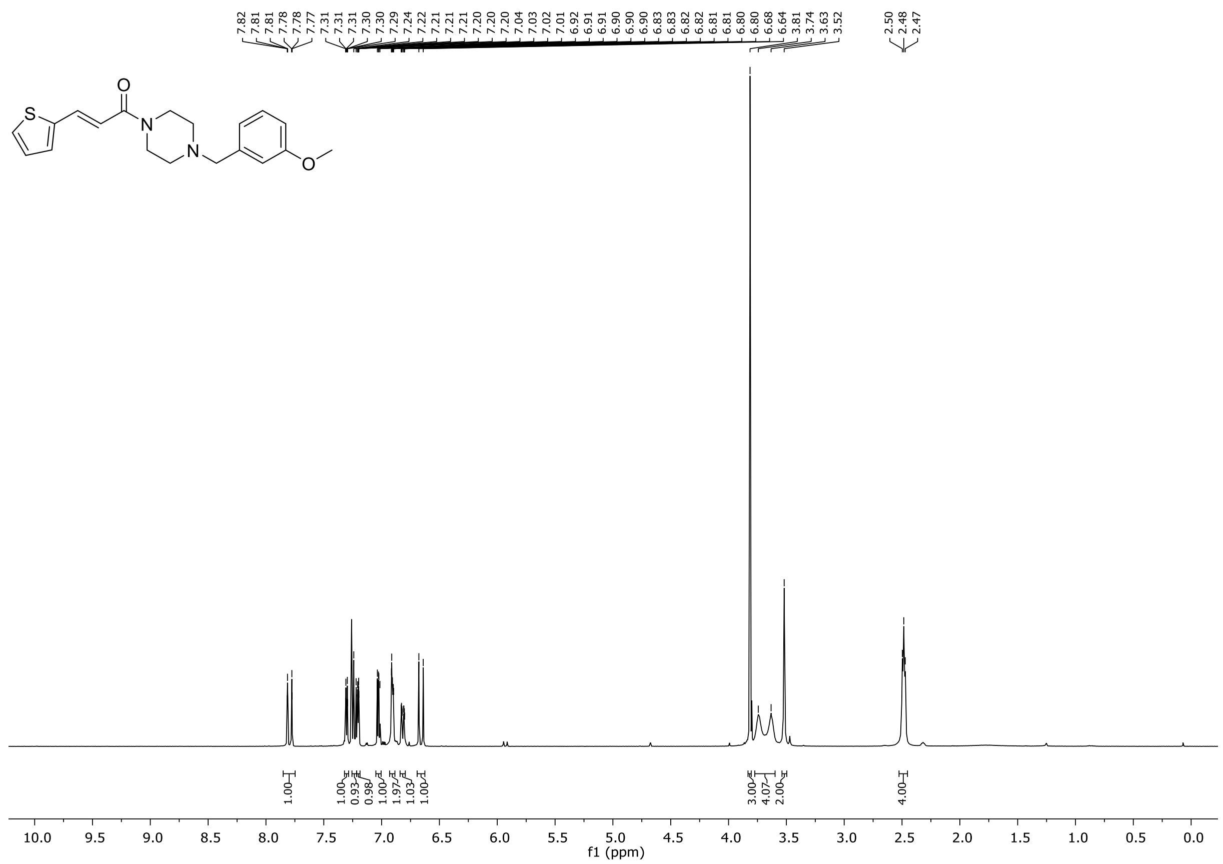

^{13}C-NMR of **APA-21** (101 MHz, $CDCl_3$)

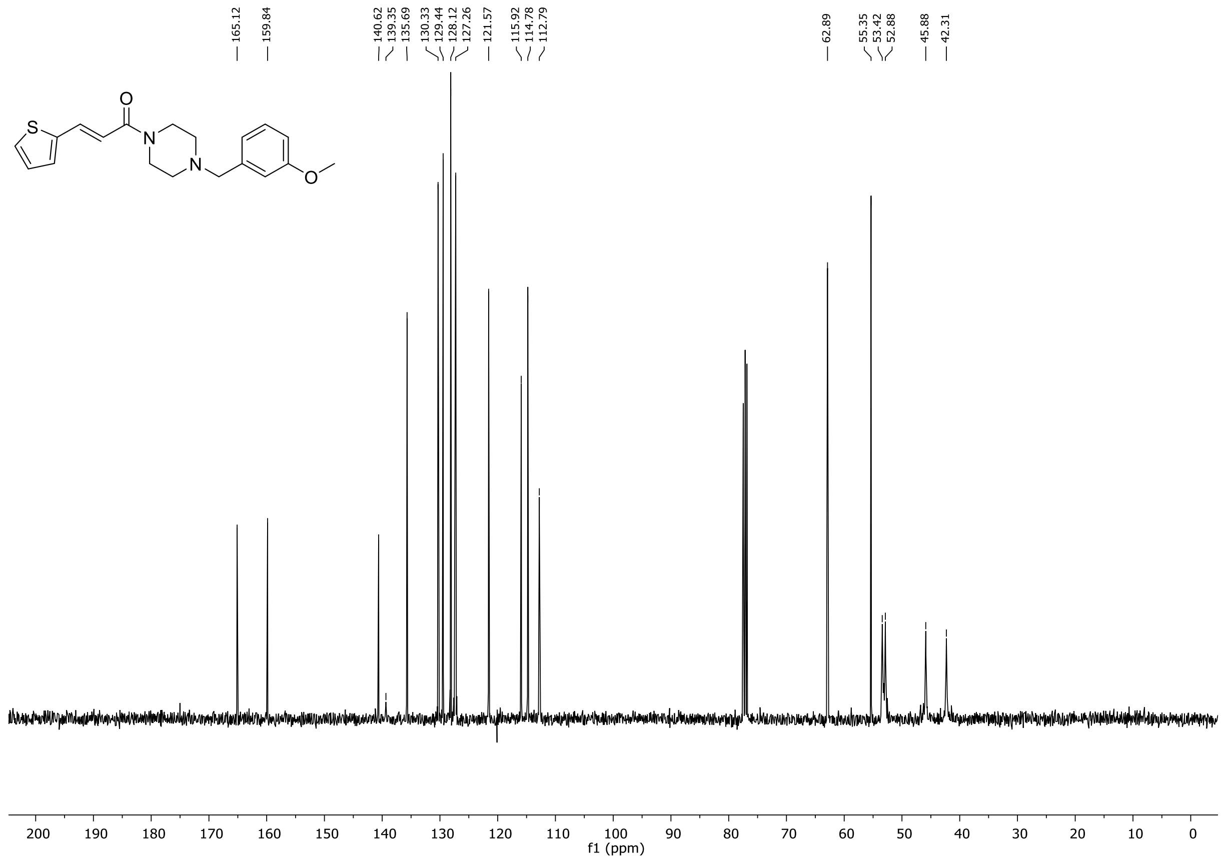

^{1}H-NMR of **APA-22** (400 MHz, $CDCl_3$)

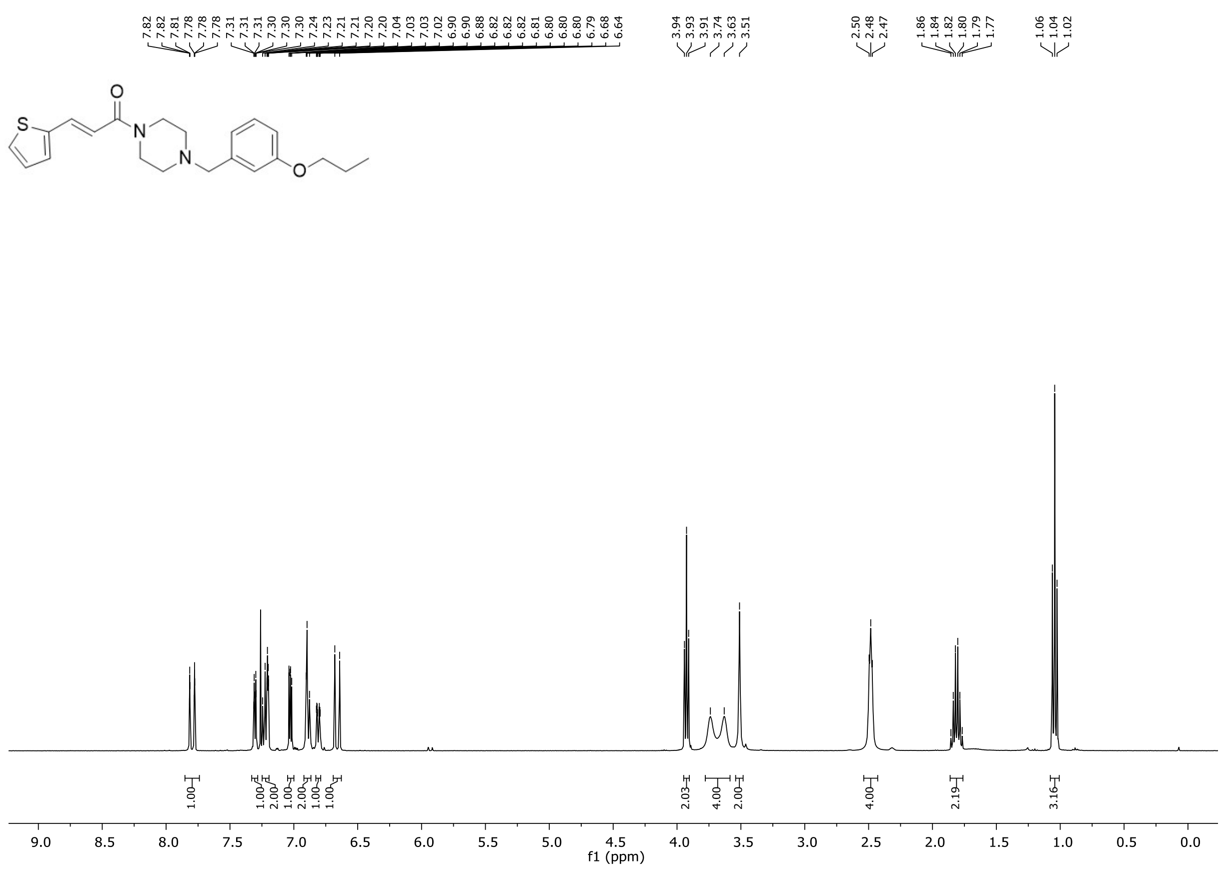

^{13}C-NMR of **APA-22** (101 MHz, $CDCl_3$)

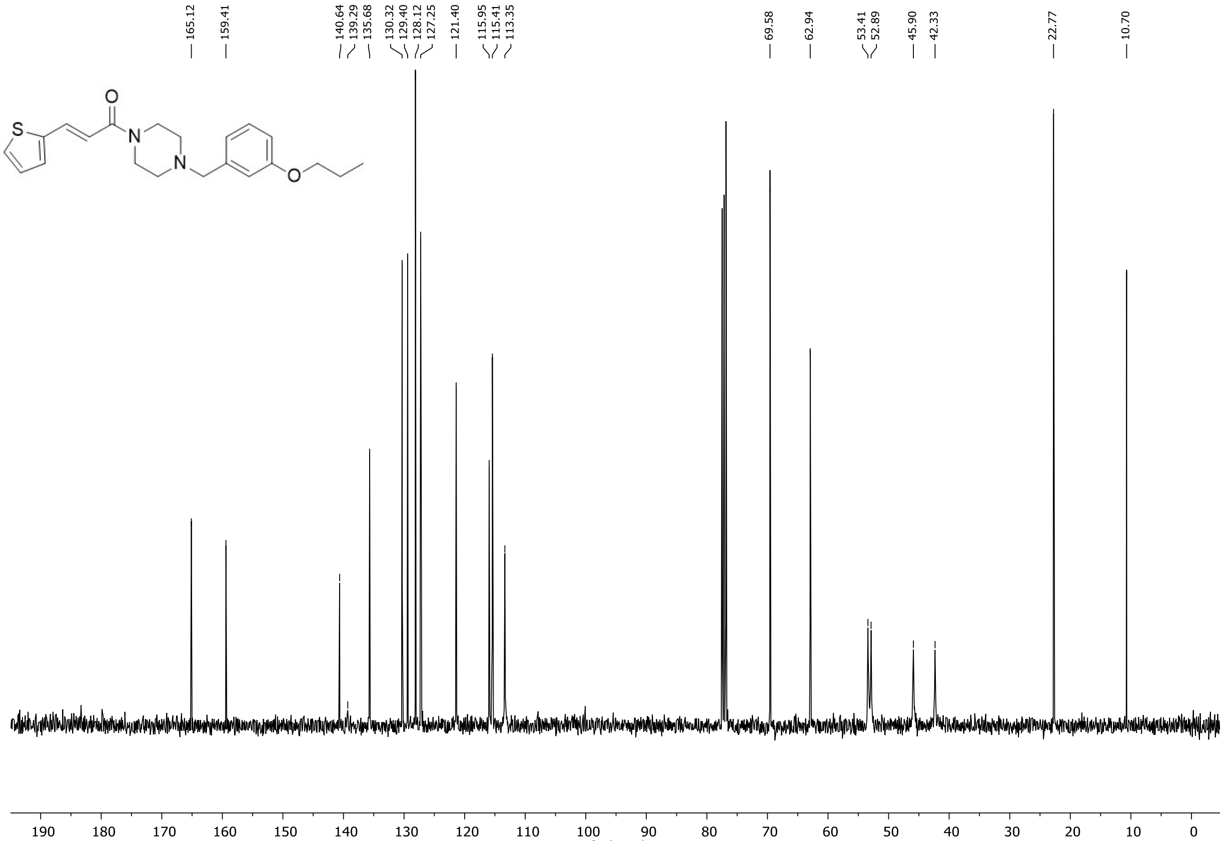

^{1}H-NMR of **APA-23** (400 MHz, $CDCl_3$)

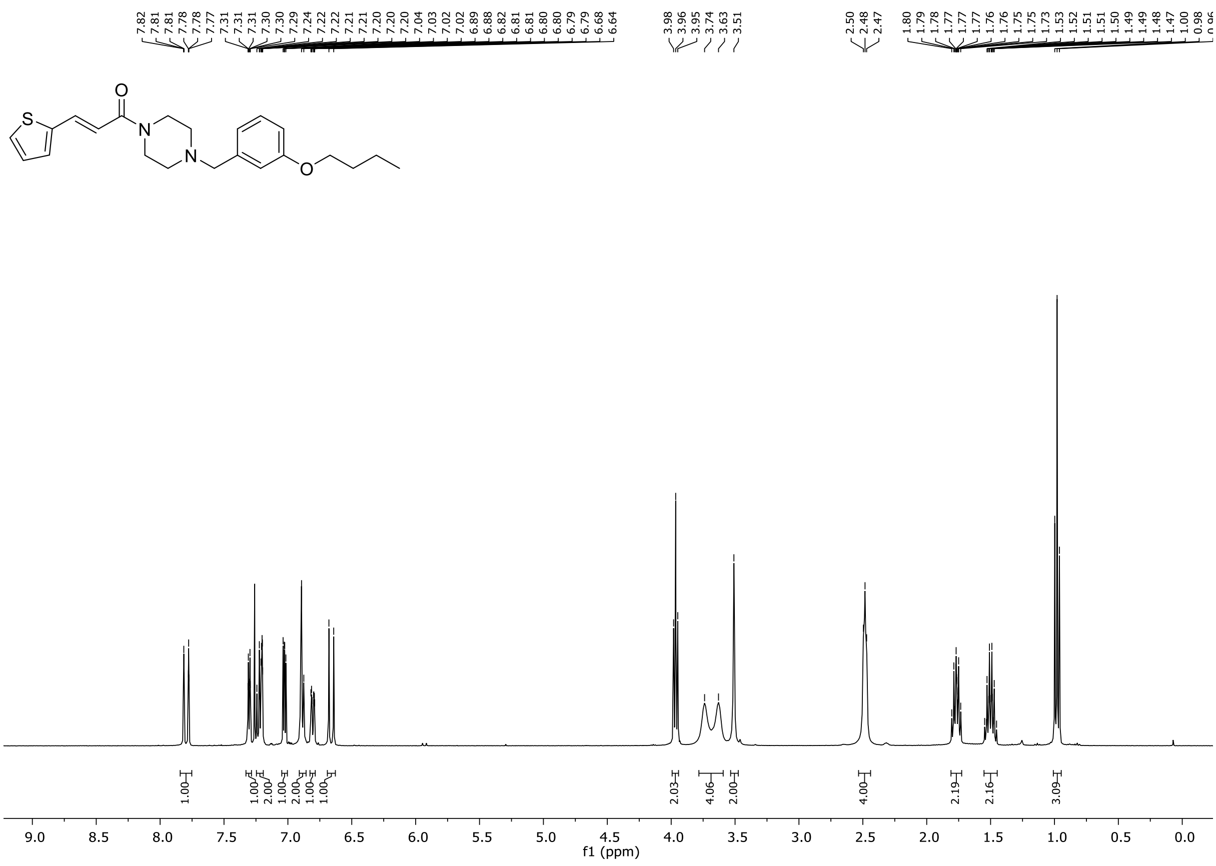

^{13}C-NMR of **APA-23** (101 MHz, $CDCl_3$)

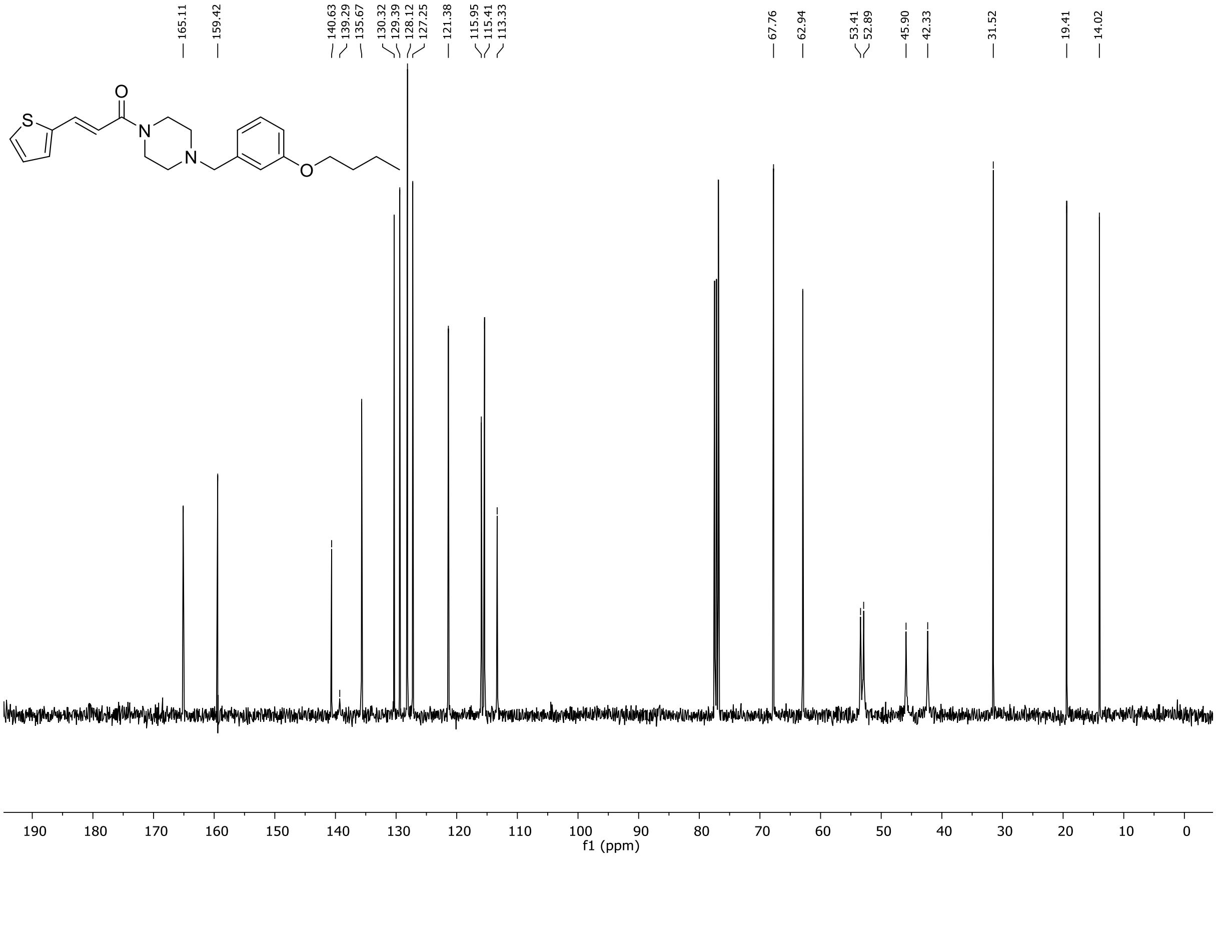

^{1}H-NMR of **APA-24** (400 MHz, CDCl$_3$)

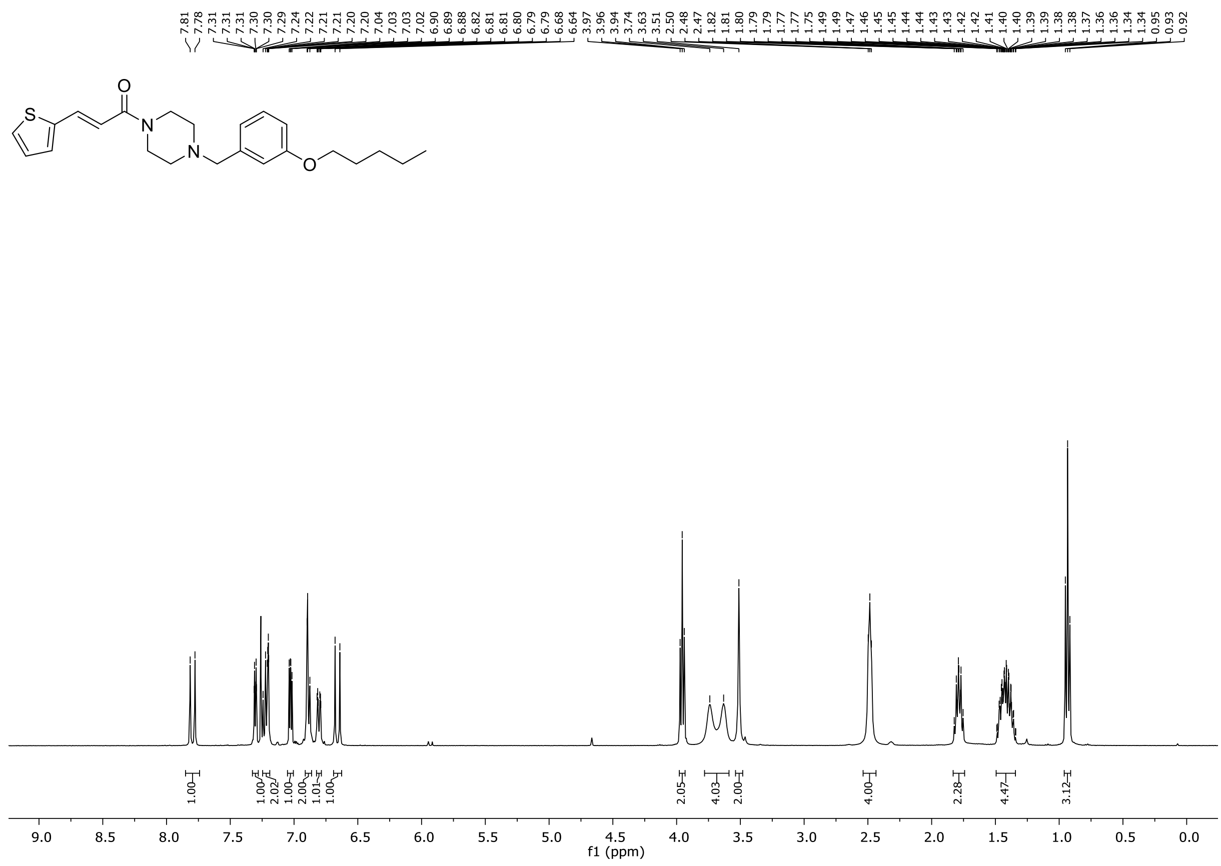

^{413}C-NMR of **APA-24** (101 MHz, CDCl$_3$)

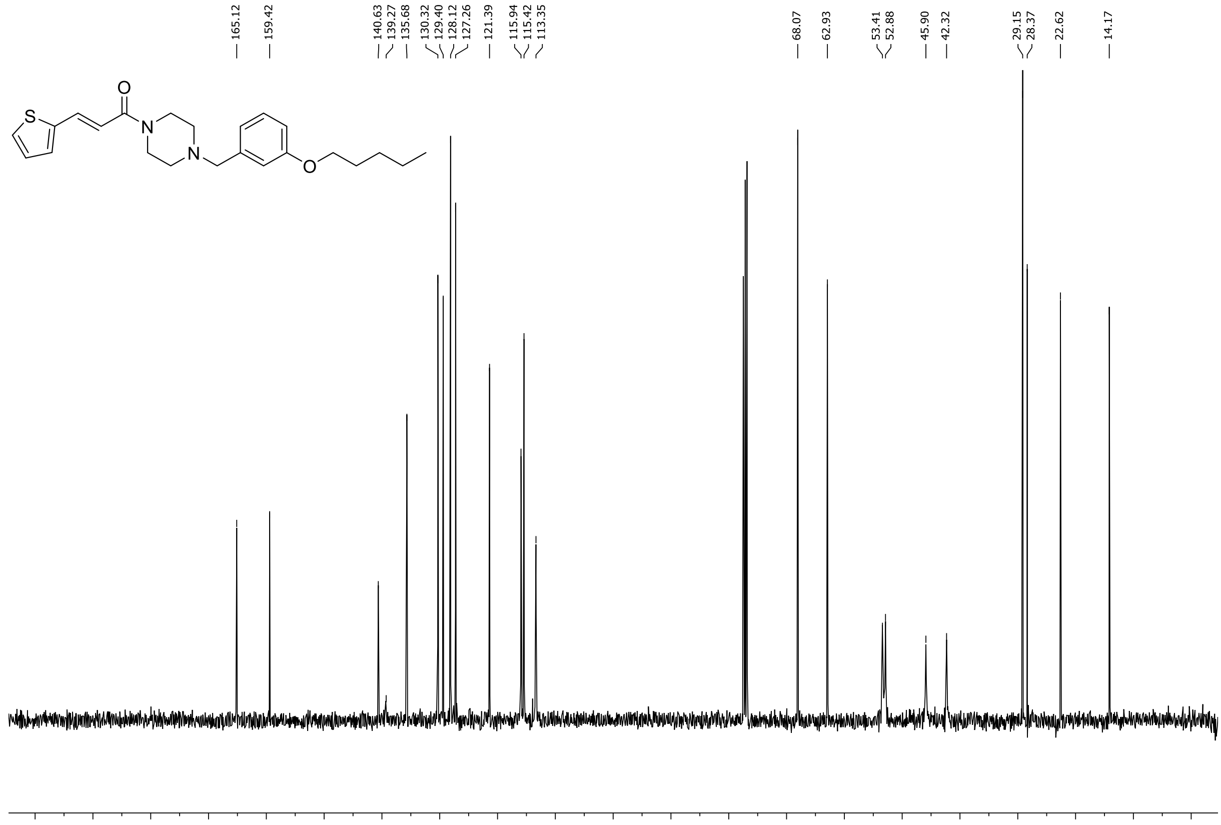

^{1}H-NMR of **APA-25** (400 MHz, $CDCl_3$)

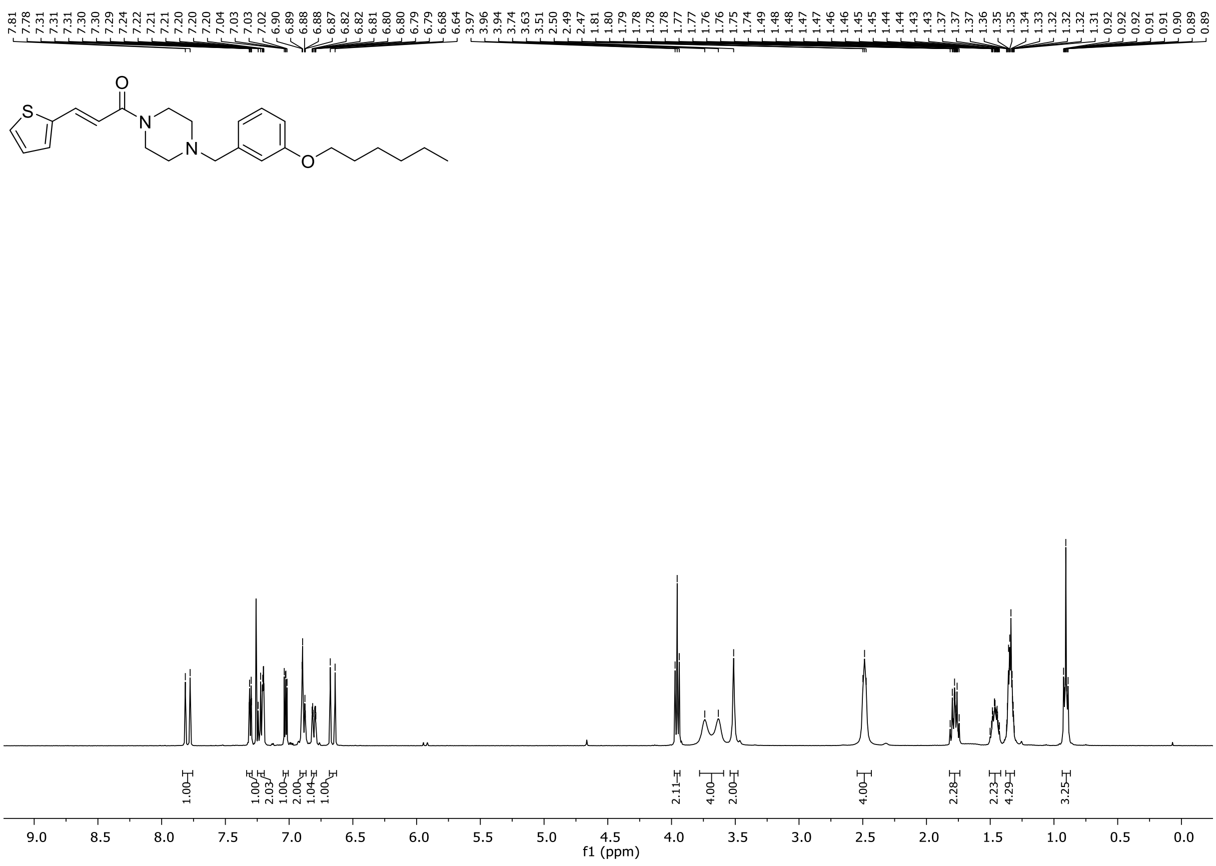

^{13}C-NMR of **APA-25** (101 MHz, $CDCl_3$)

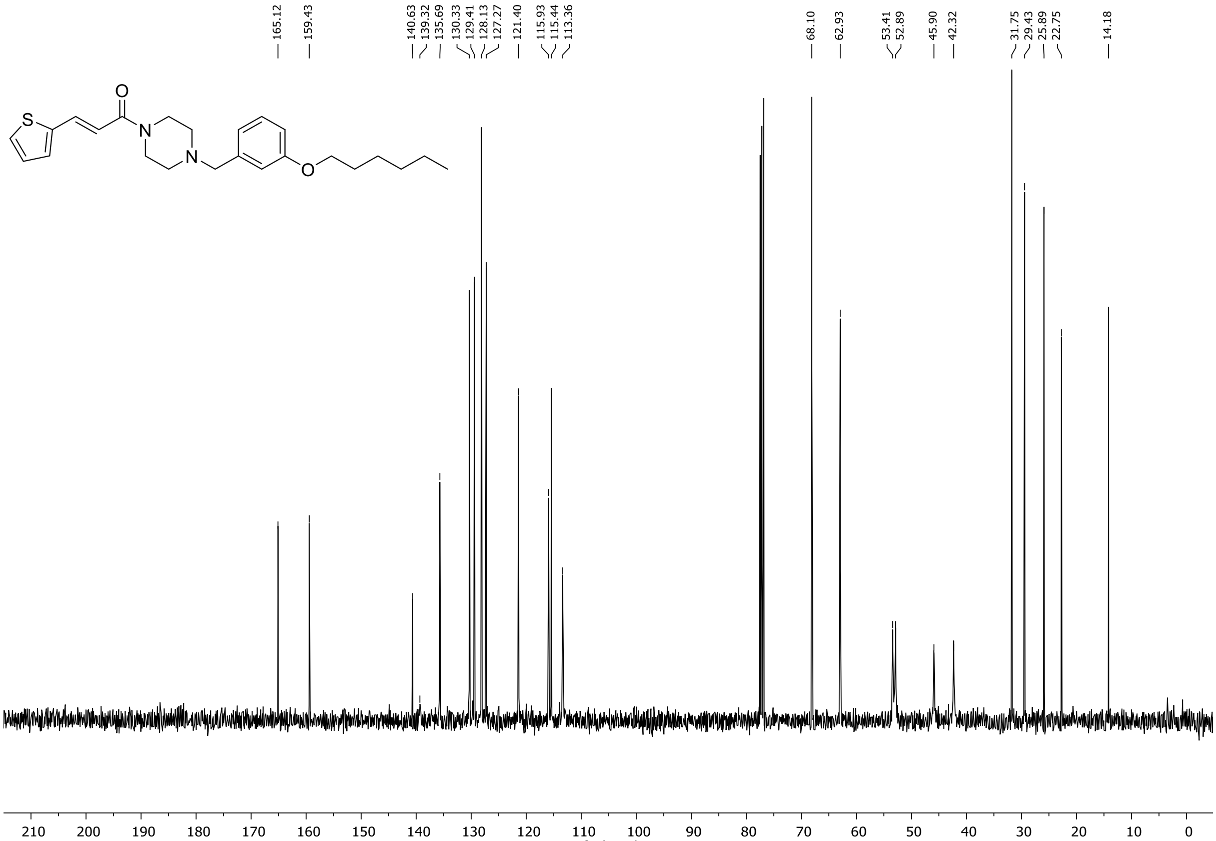

^{1}H-NMR of **I-15** (400 MHz, $CDCl_3$)

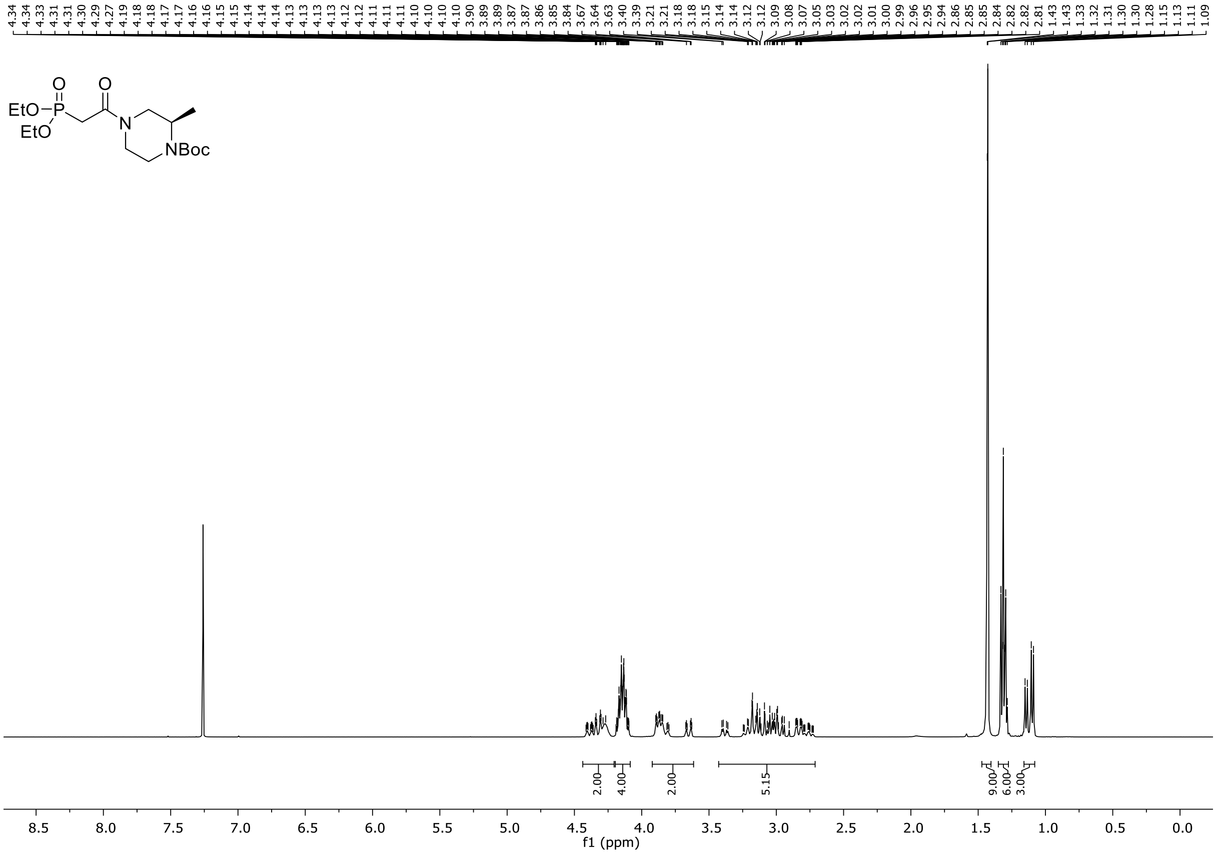

^{13}C-NMR of **I-15** (101 MHz, $CDCl_3$)

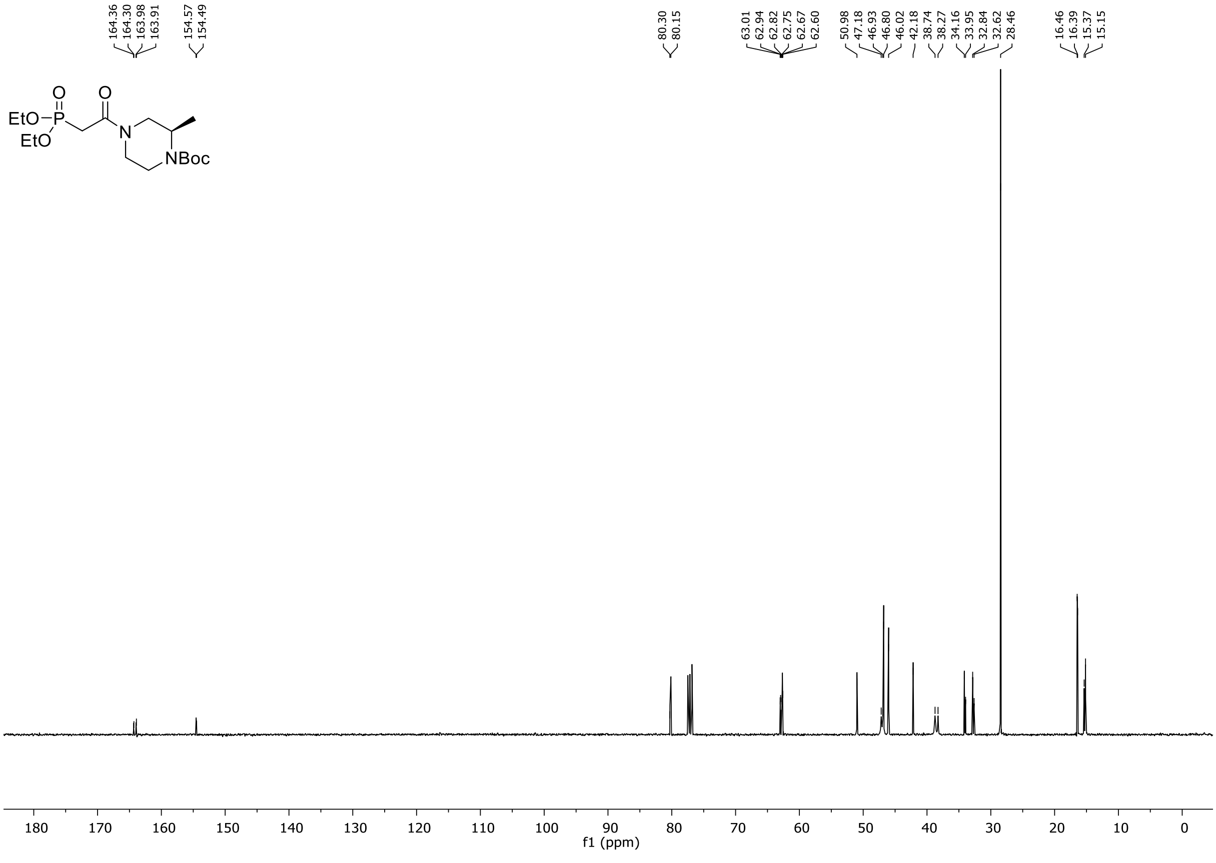

^{1}H-NMR of **I-16** (400 MHz, $CDCl_3$)

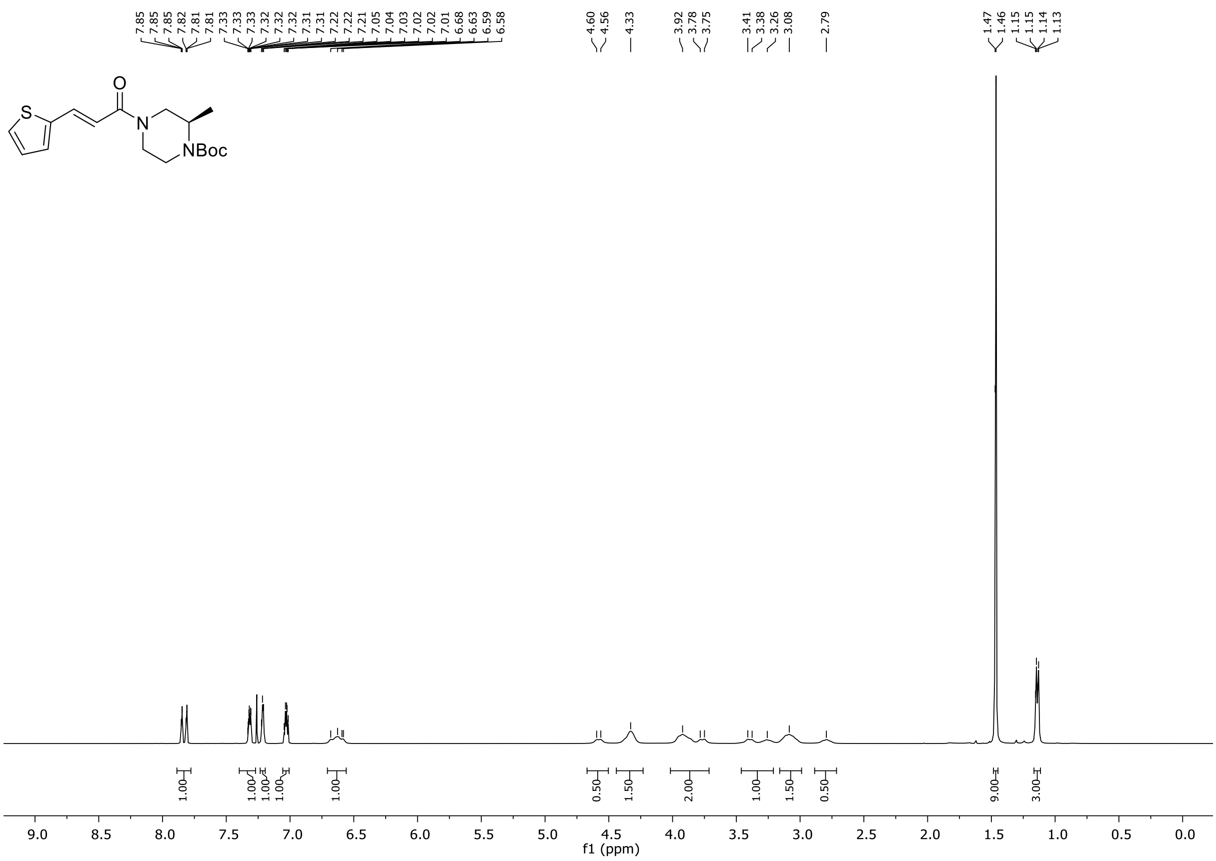

^{13}C-NMR of **I-16** (101 MHz, $CDCl_3$)

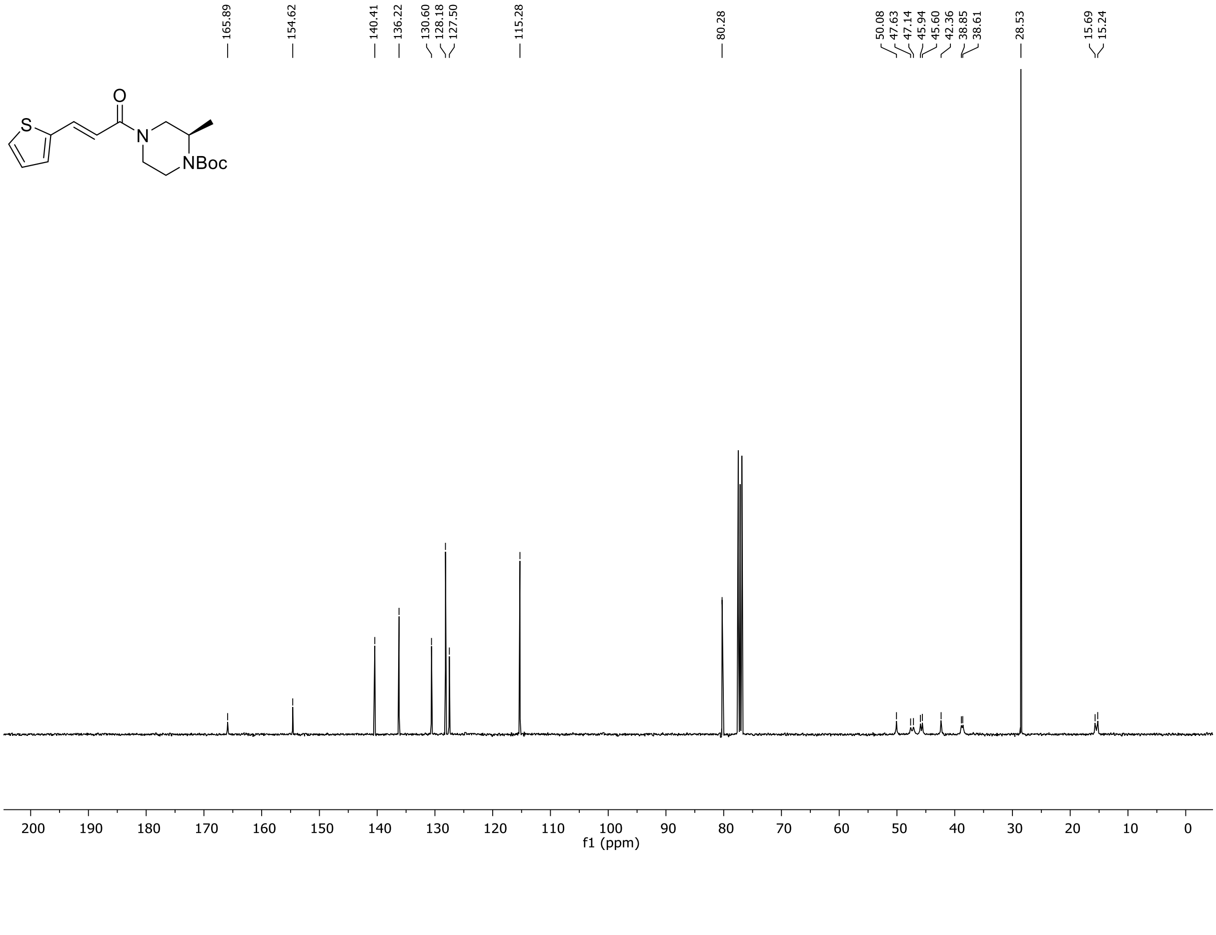

^{1}H-NMR of **I-17** (400 MHz, $CDCl_3$)

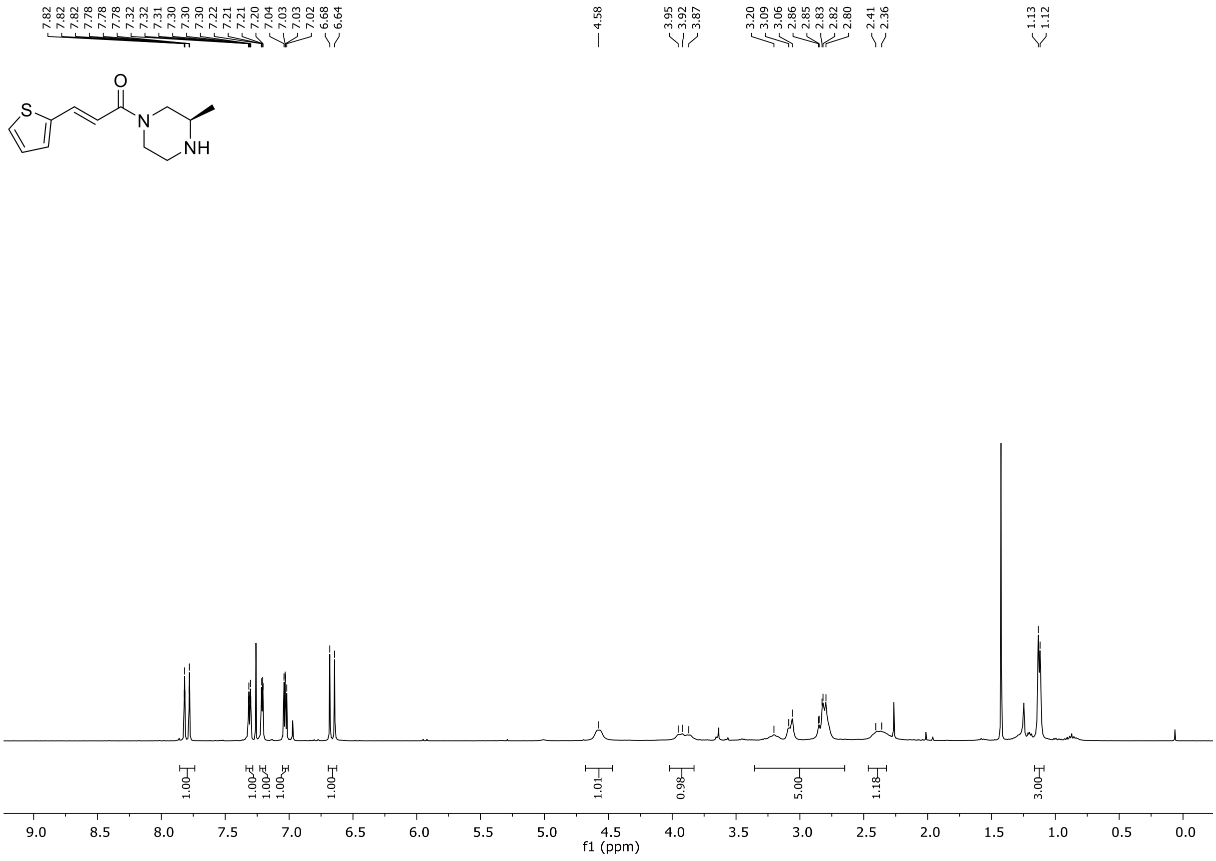

^{13}C-NMR of **I-17** (101 MHz, $CDCl_3$)

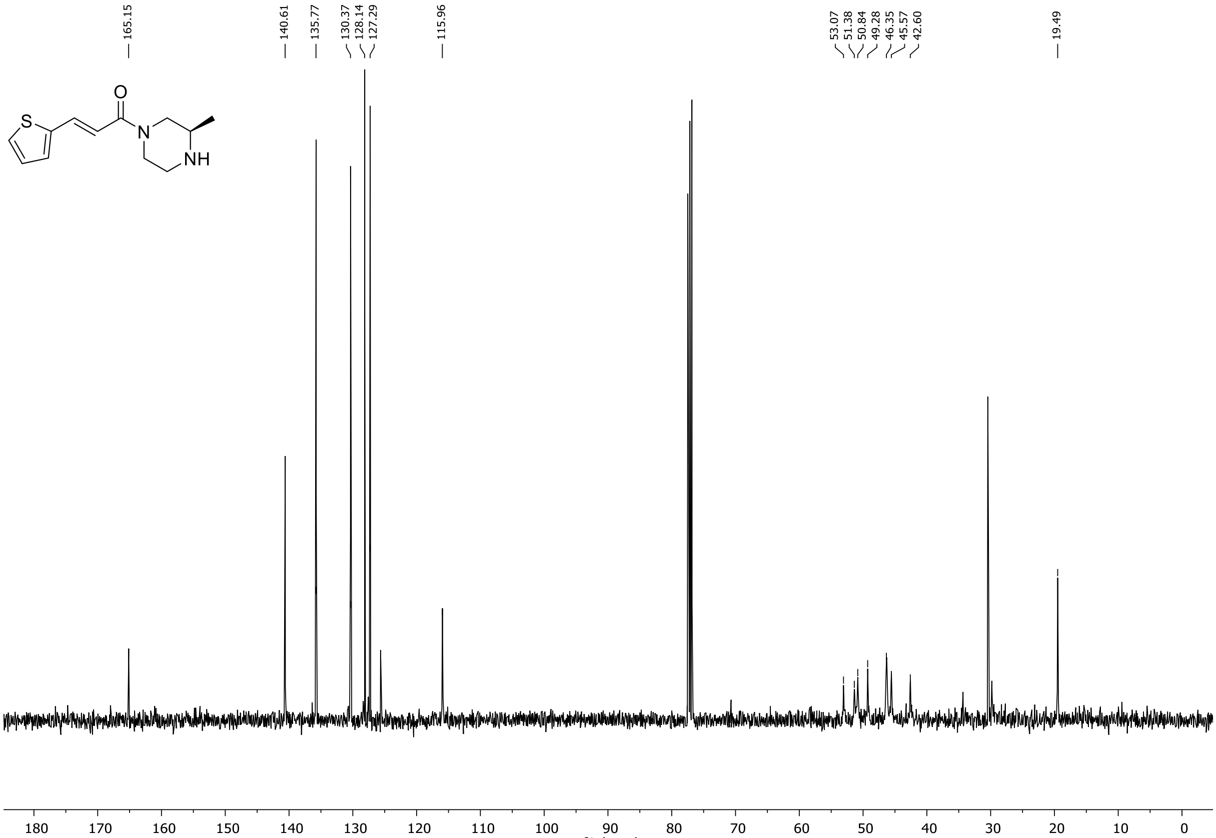

^{1}H-NMR of **APA-26** (400 MHz, $CDCl_3$)

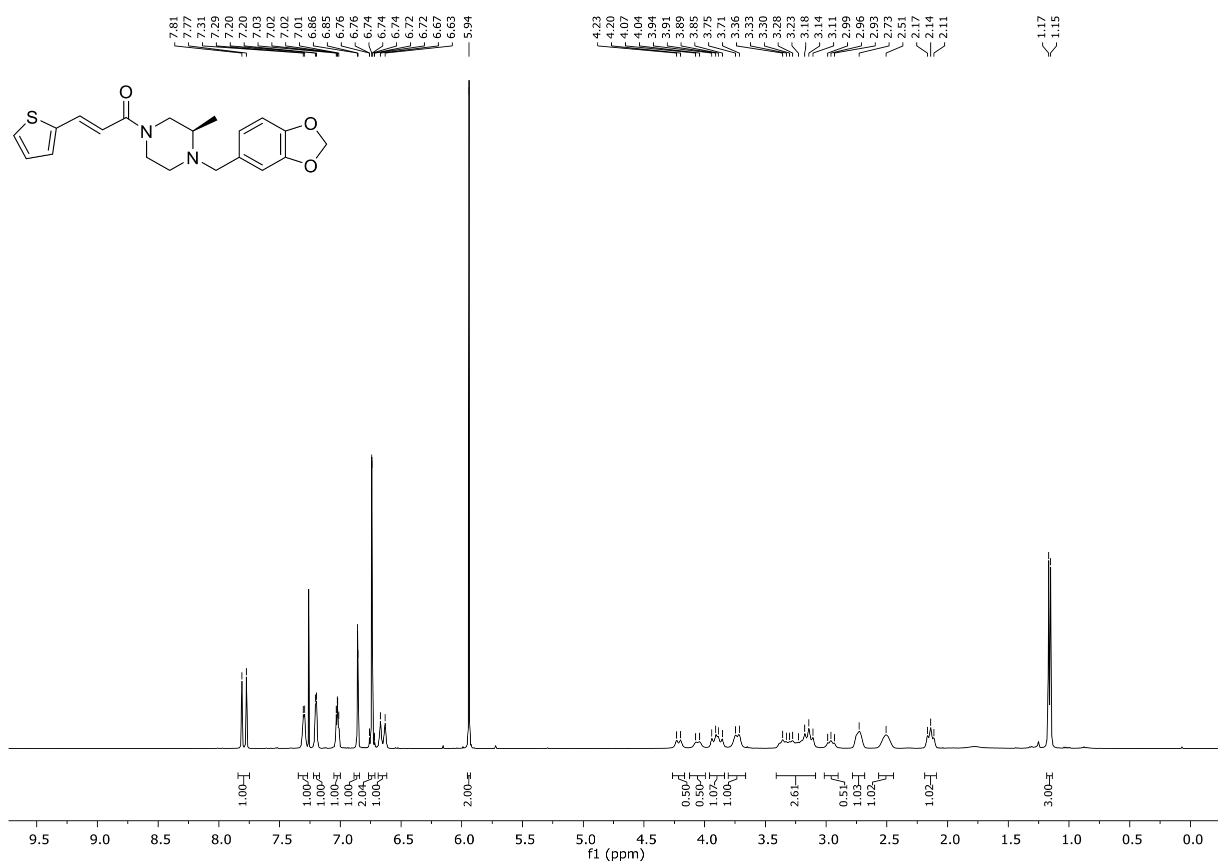

^{13}C-NMR of **APA-26** (101 MHz, $CDCl_3$)

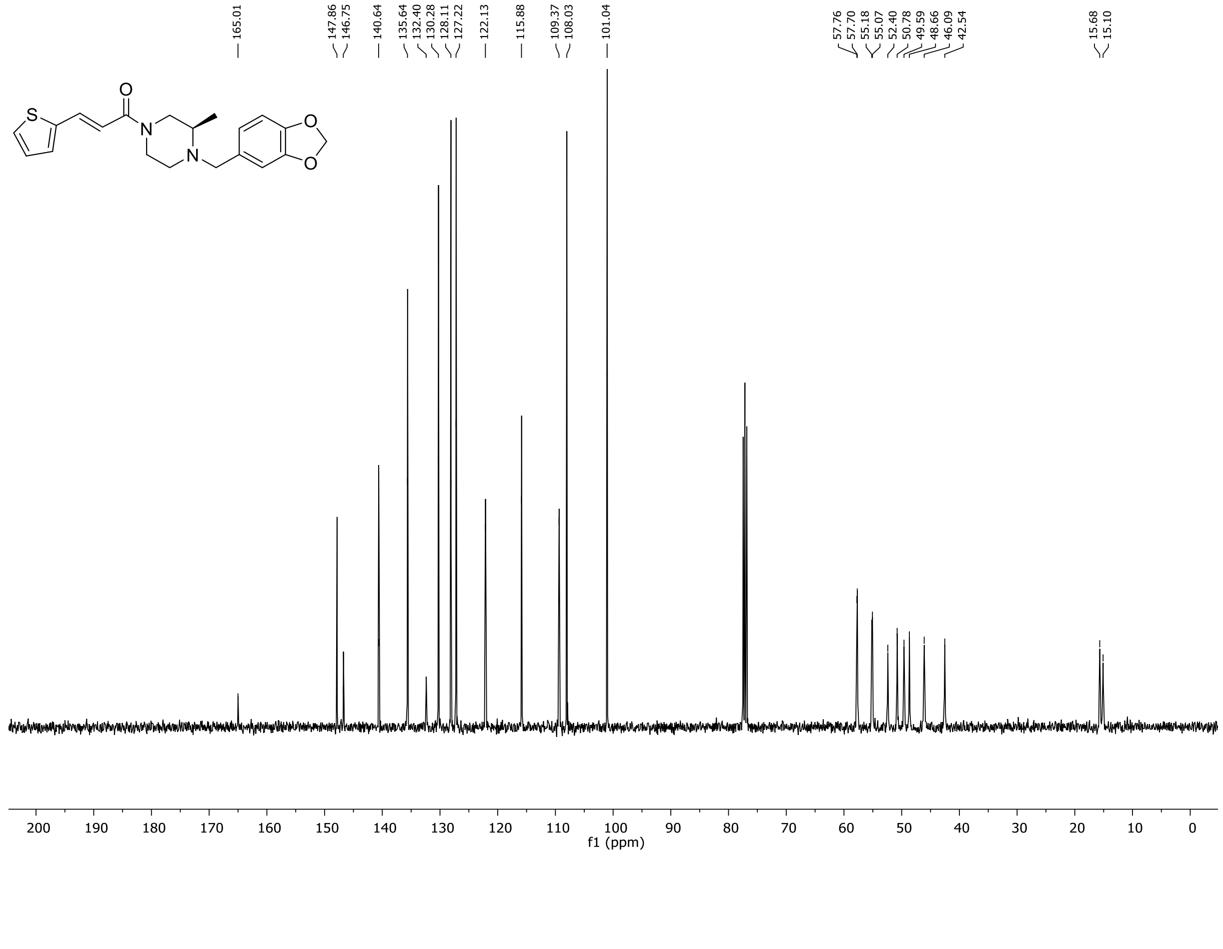

^{1}H-NMR of **I-18** (400 MHz, $CDCl_3$)

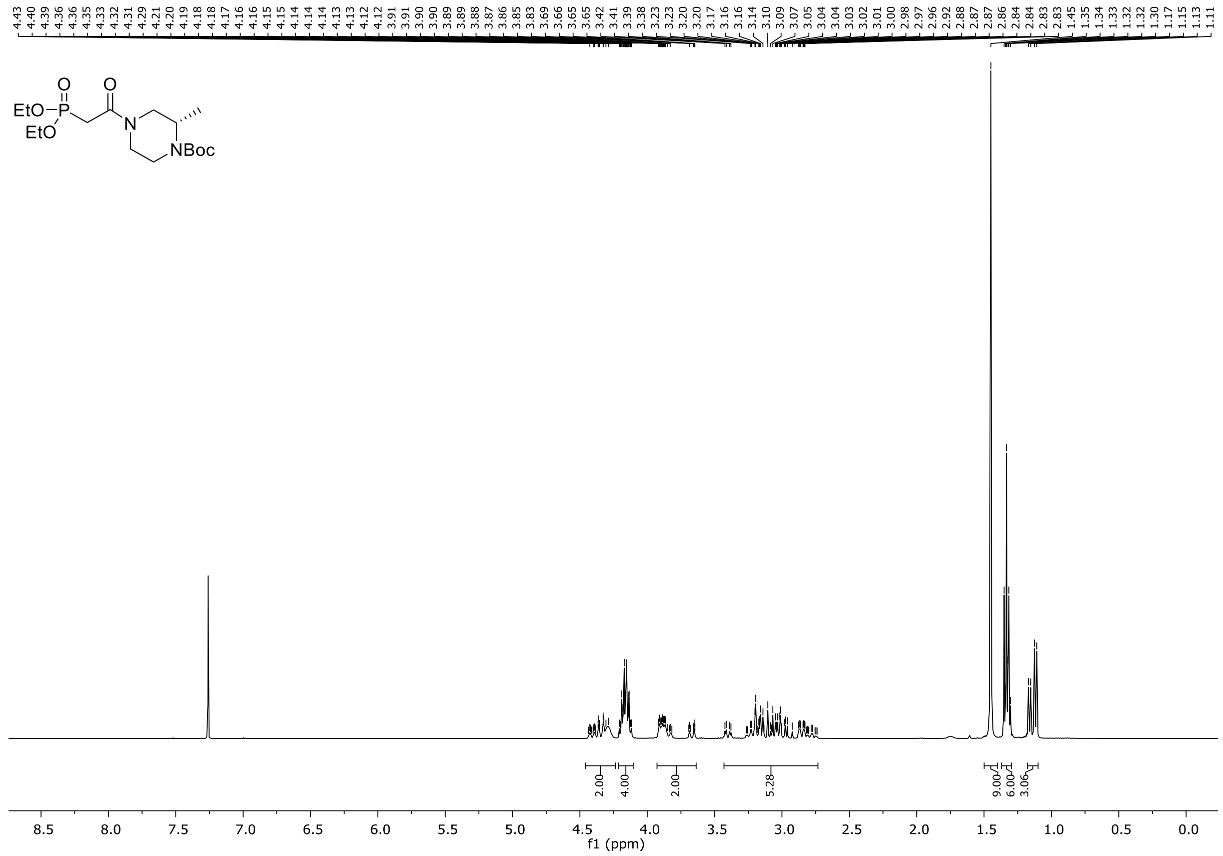

^{13}C-NMR of **I-18** (101 MHz, $CDCl_3$)

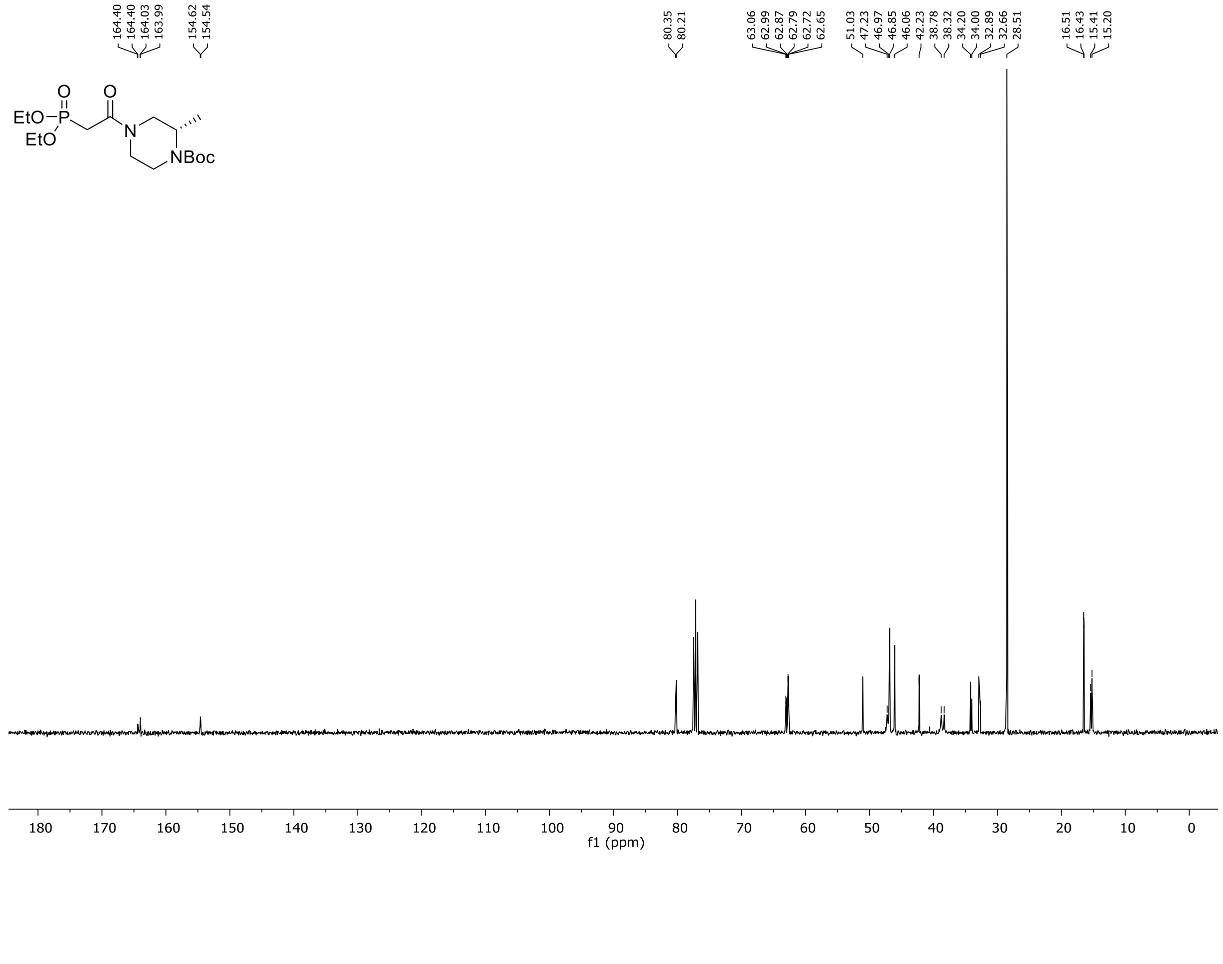

^{1}H-NMR of **I-19** (400 MHz, $CDCl_3$)

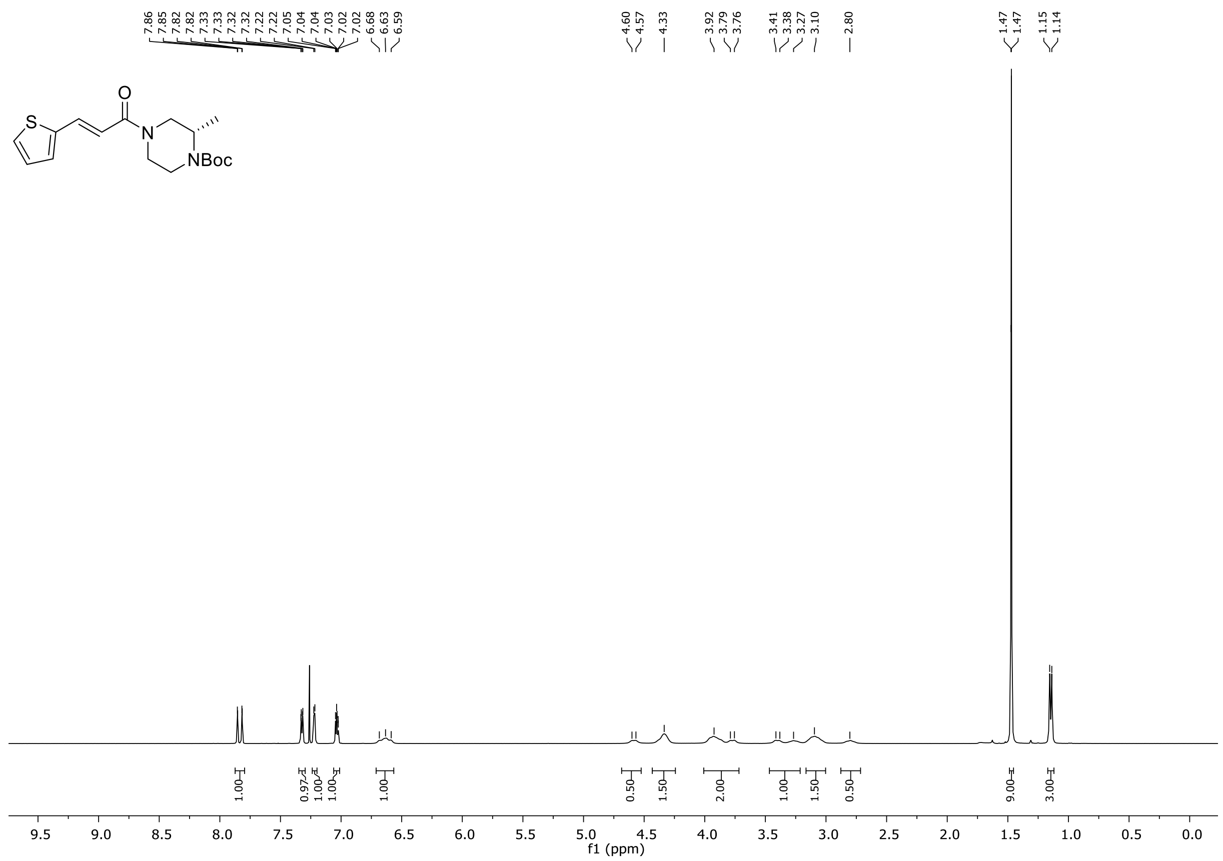

^{13}C-NMR of **I-19** (101 MHz, $CDCl_3$)

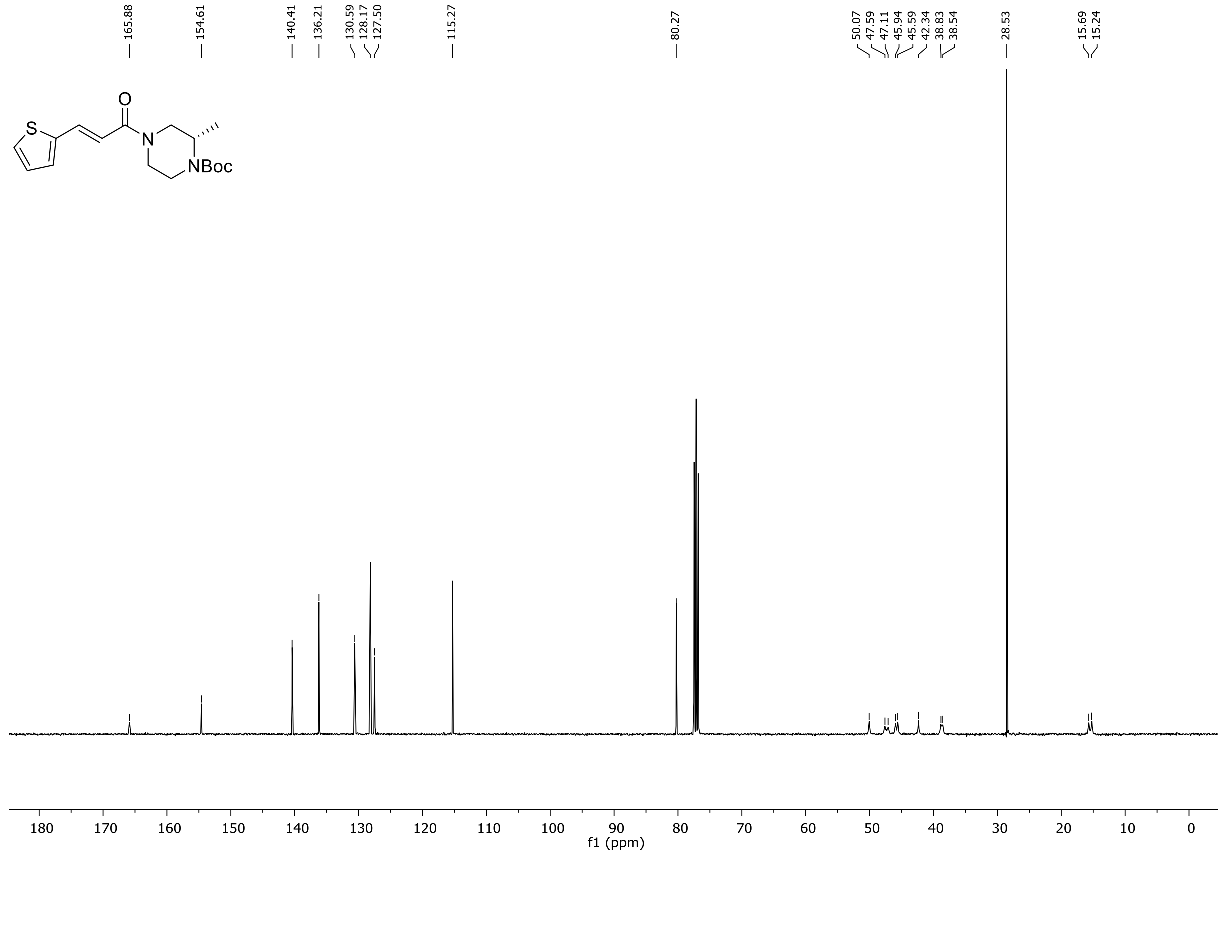

^{1}H-NMR of **I-20** (400 MHz, $CDCl_3$)

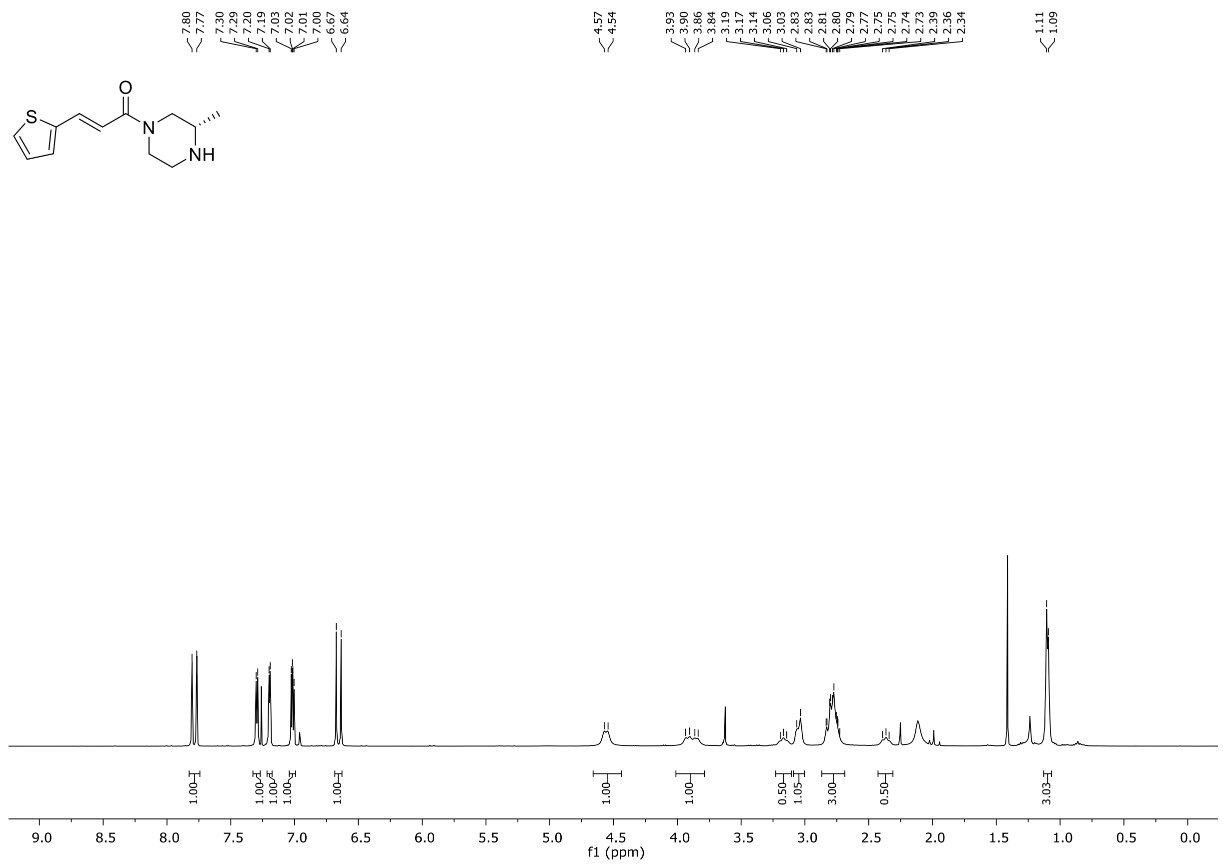

^{13}C-NMR of **I-20** (101 MHz, $CDCl_3$)

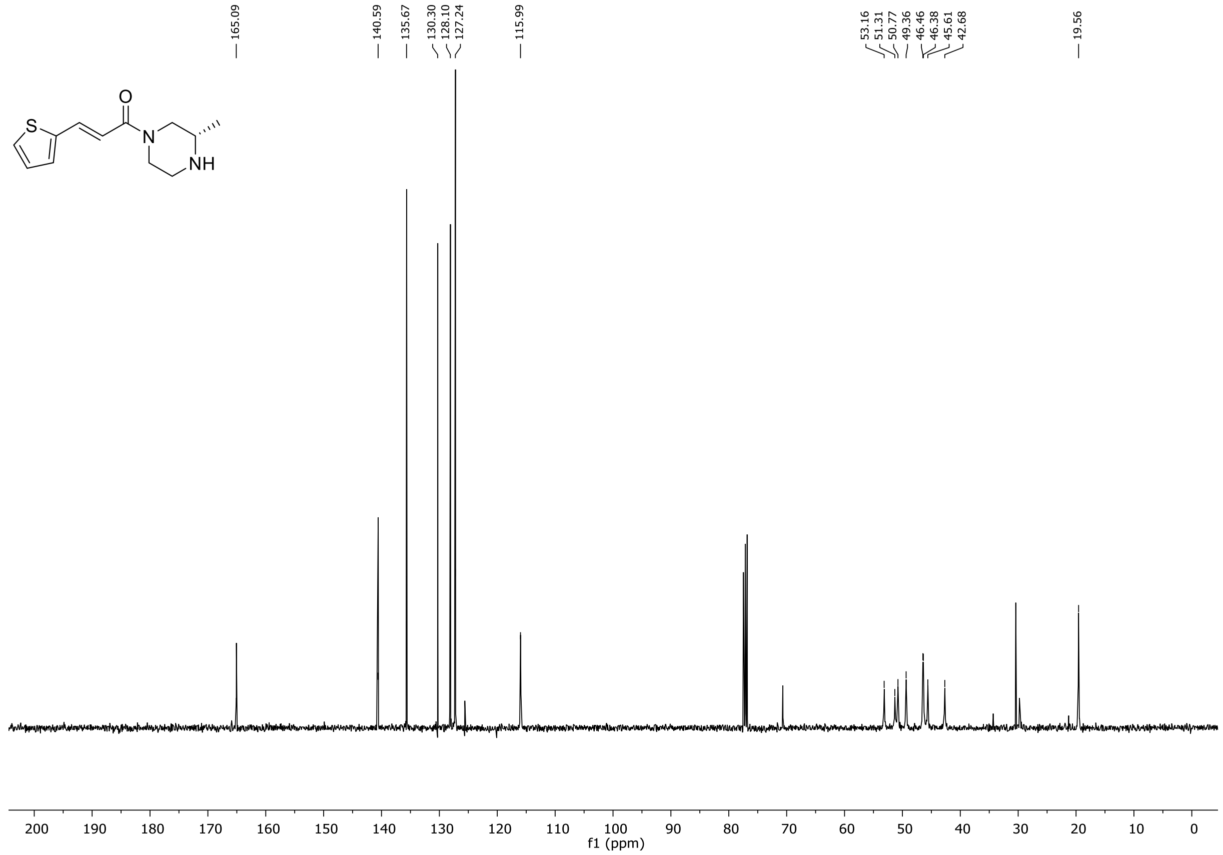

^{1}H-NMR of **APA-27** (400 MHz, $CDCl_3$)

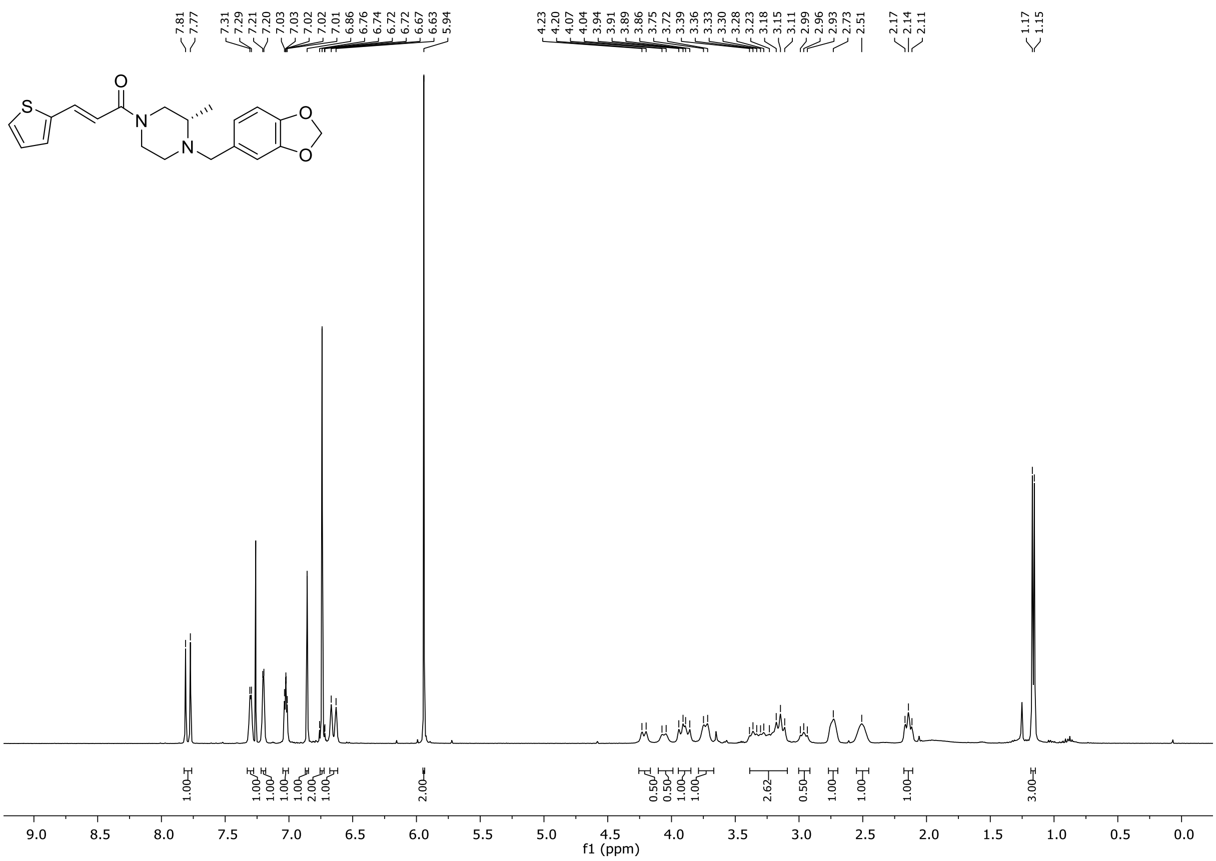

^{13}C-NMR of **APA-27** (101 MHz, $CDCl_3$)

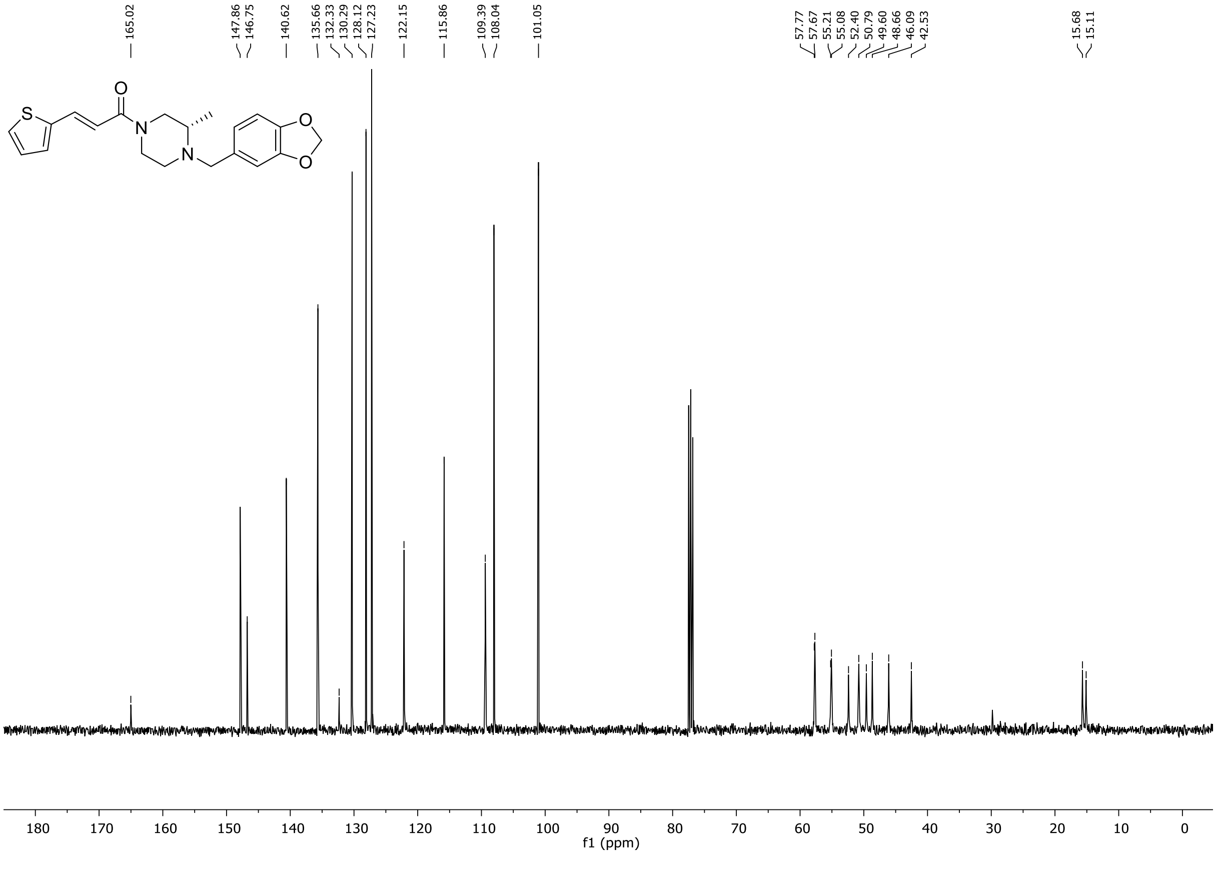

653

AQ-1

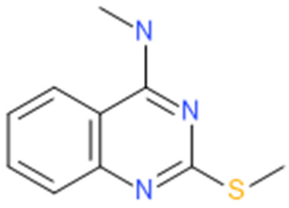

CAS 338776-94-6; From Key Organics, 5N-544S

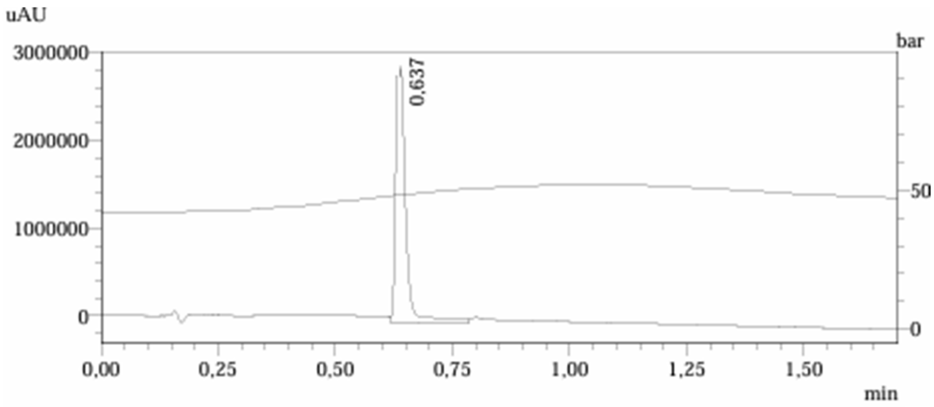

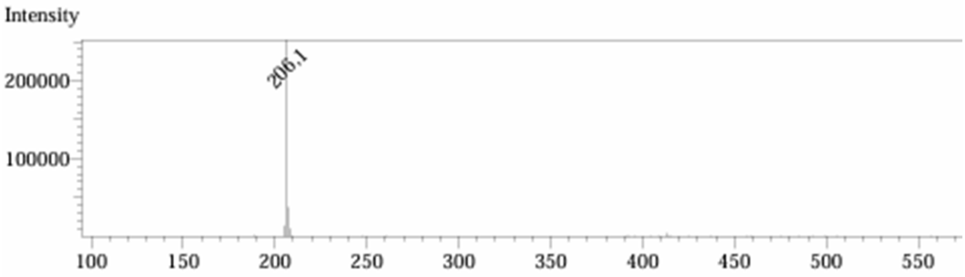

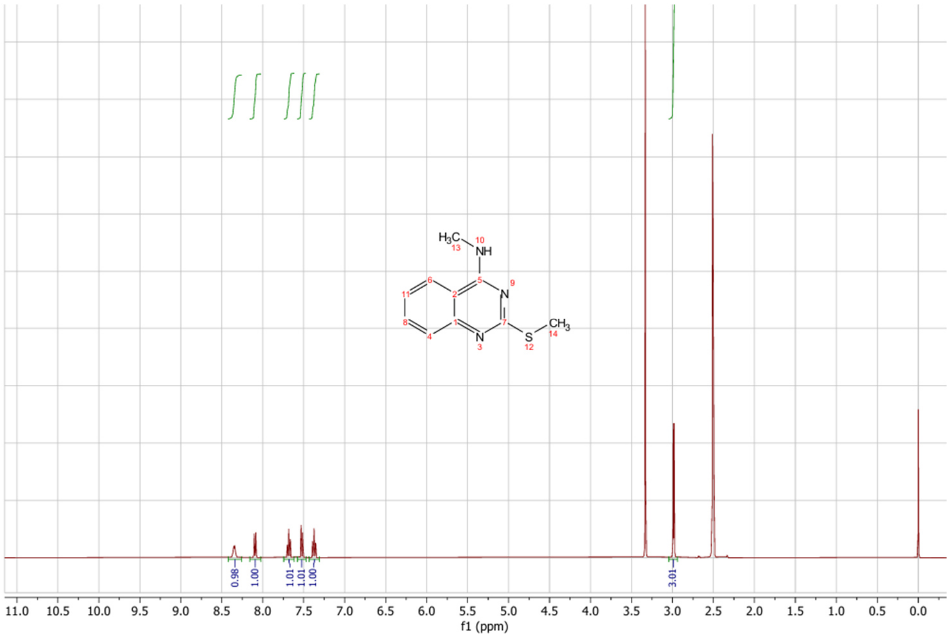

AQ-2

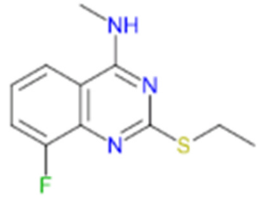

CAS 3010279-62-3

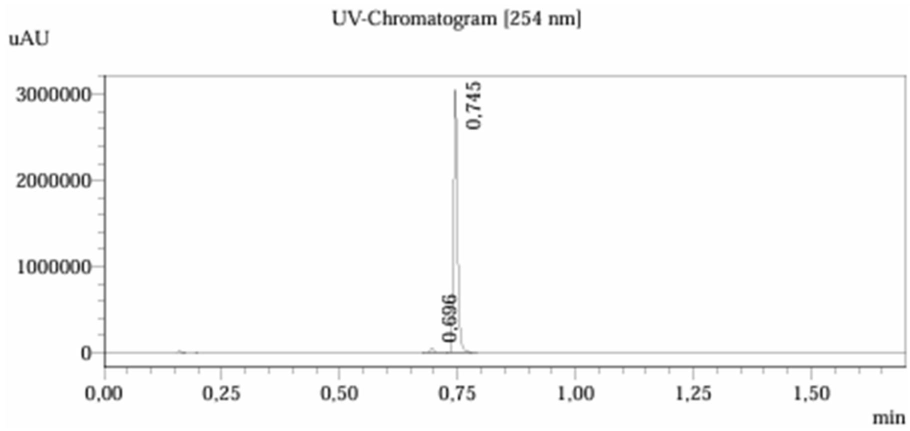

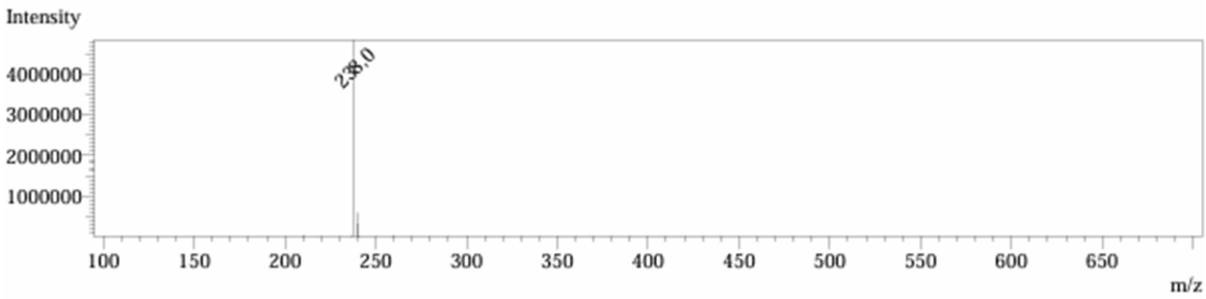

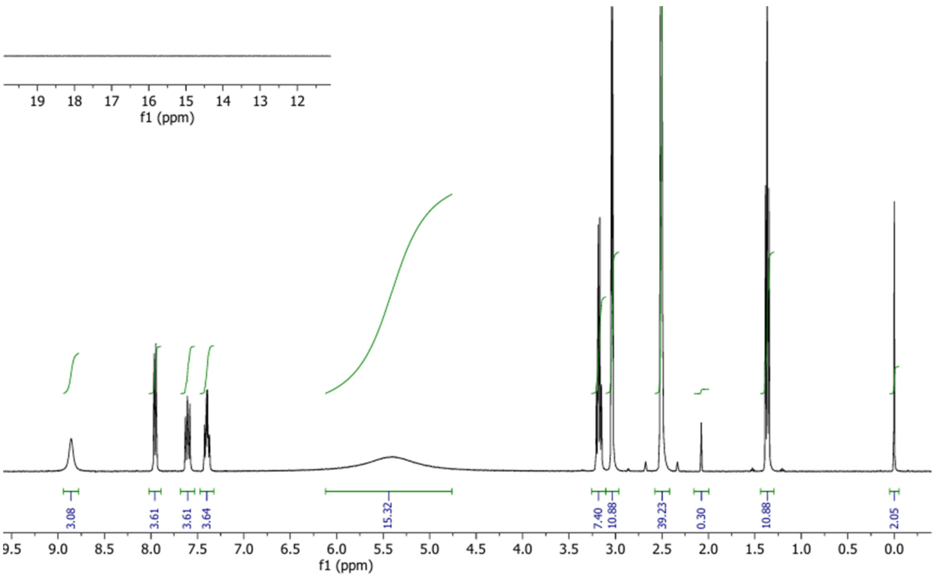

AQ-5

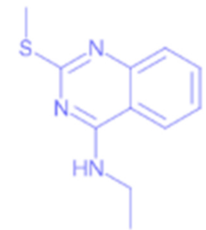

CAS 81683-38-7; From Enamine, Z821298510

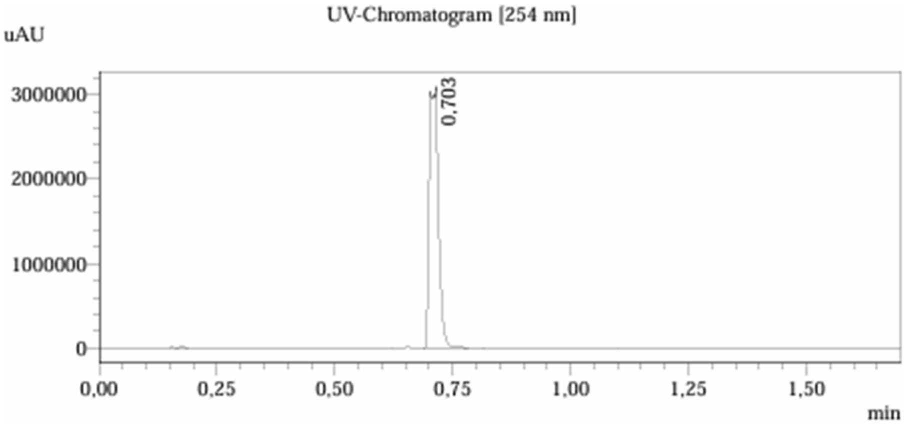

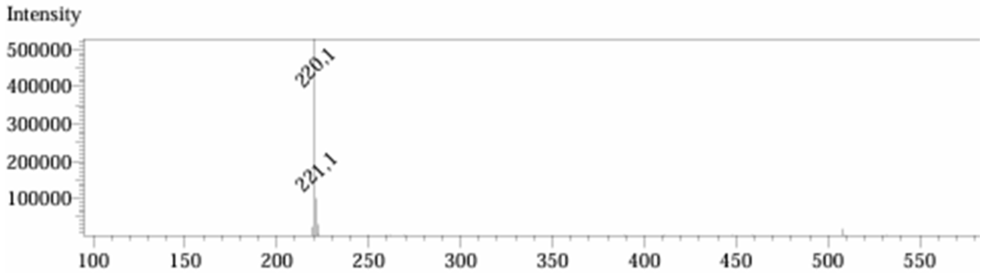

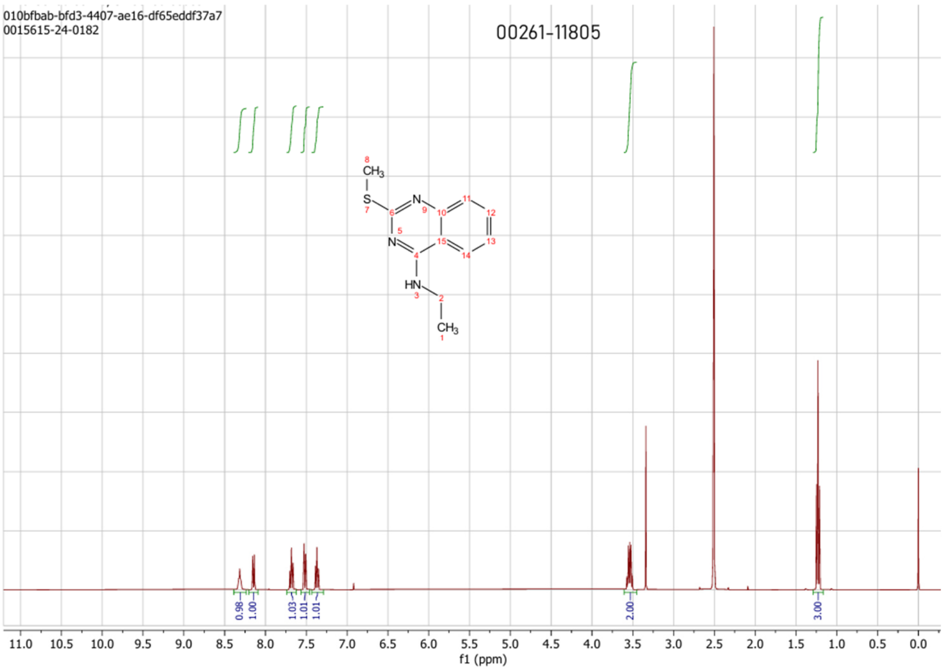

AQ-6

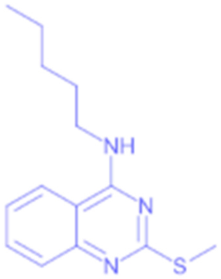
NH
N
N
S

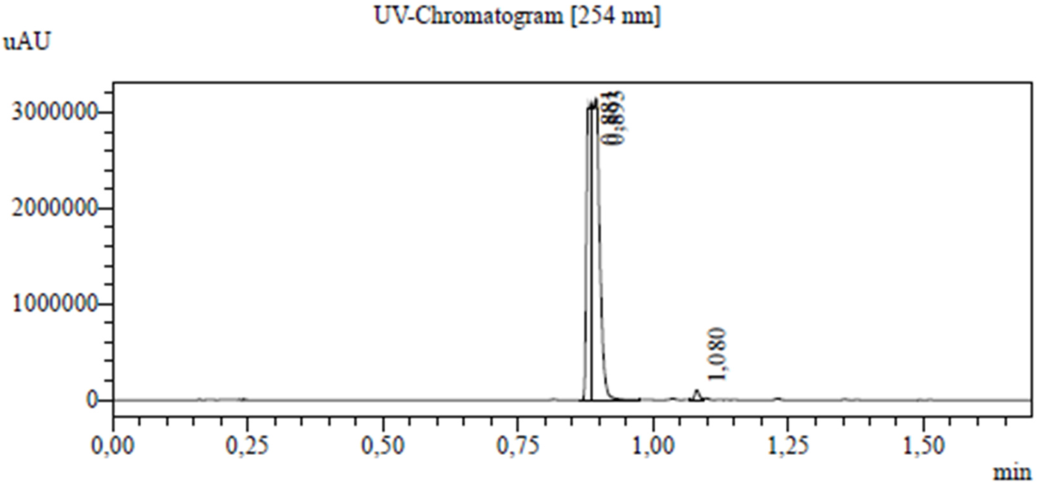
UV-Chromatogram [254 nm]
uAU
3000000
2000000
1000000
0
0,883
1,080
0,00
0,25
0,50
0,75
1,00
1,25
1,50
min

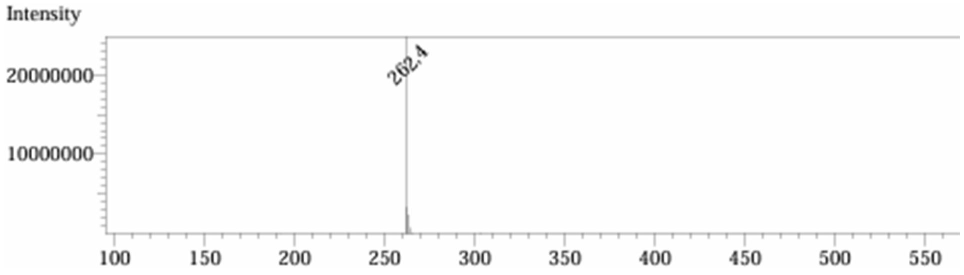
Intensity
20000000
10000000
262,4
100
150
200
250
300
350
400
450
500
550

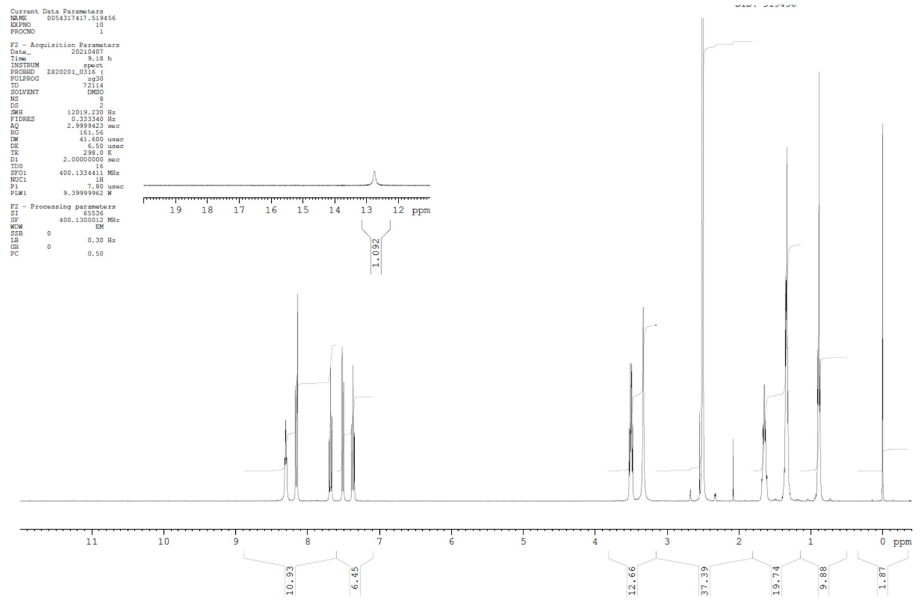
1.092
10.93
6.45
12.66
37.39
19.74
9.88
1.87
ppm

AQ-7

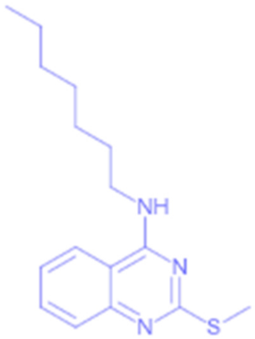

NH
N
N
S

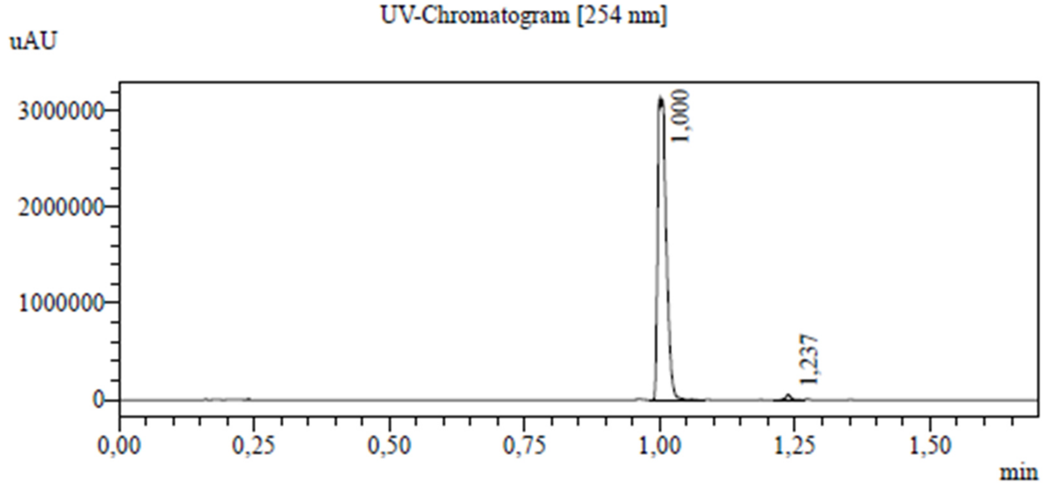

UV-Chromatogram [254 nm]
uAU
3000000
2000000
1000000
0
1,000
1,237
0,00
0,25
0,50
0,75
1,00
1,25
1,50
min

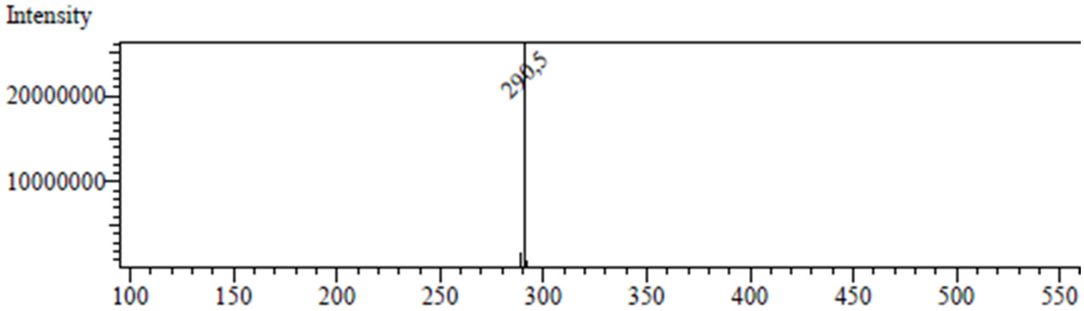

Intensity
20000000
10000000
290,5
100
150
200
250
300
350
400
450
500
550

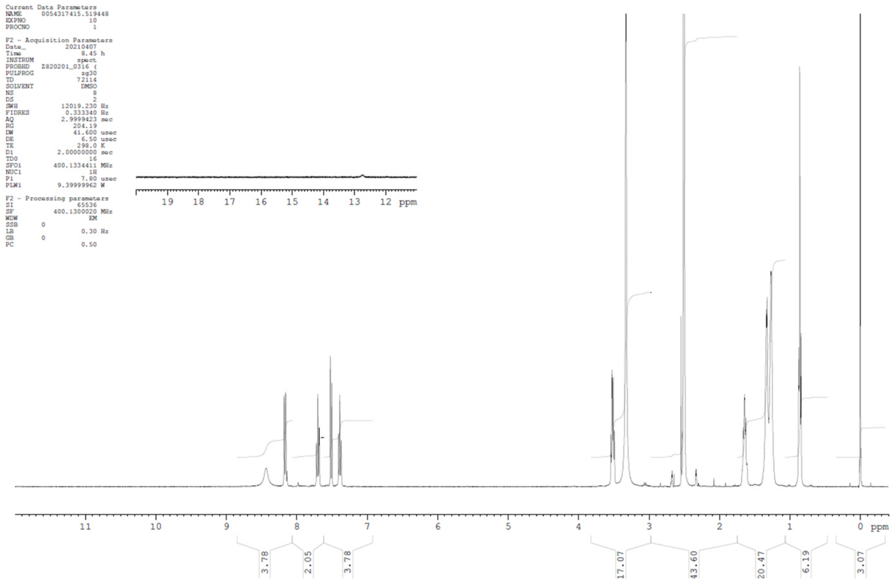

19
18
17
16
15
14
13
12
ppm
11
10
9
8
7
6
5
4
3
2
1
0
ppm
3.78
2.05
3.78
17.07
43.60
20.47
6.19
3.07

AQ-8

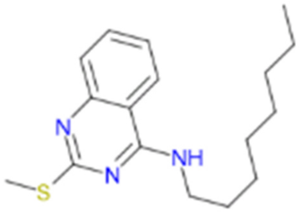

N
N
NH
S

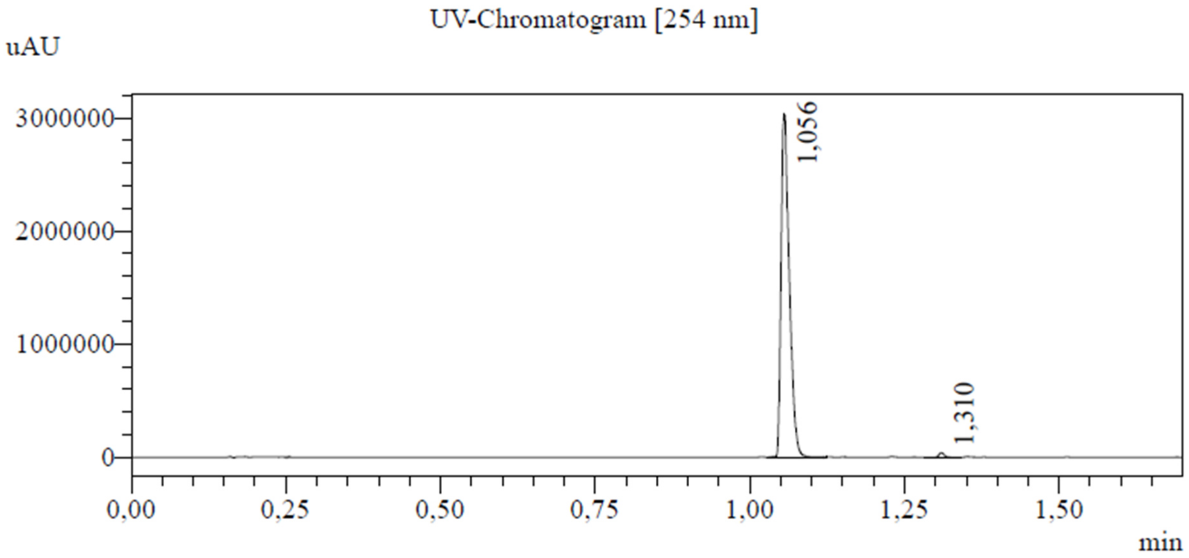

UV-Chromatogram [254 nm]
uAU
3000000
2000000
1000000
0
1,056
1,310
0,00
0,25
0,50
0,75
1,00
1,25
1,50
min

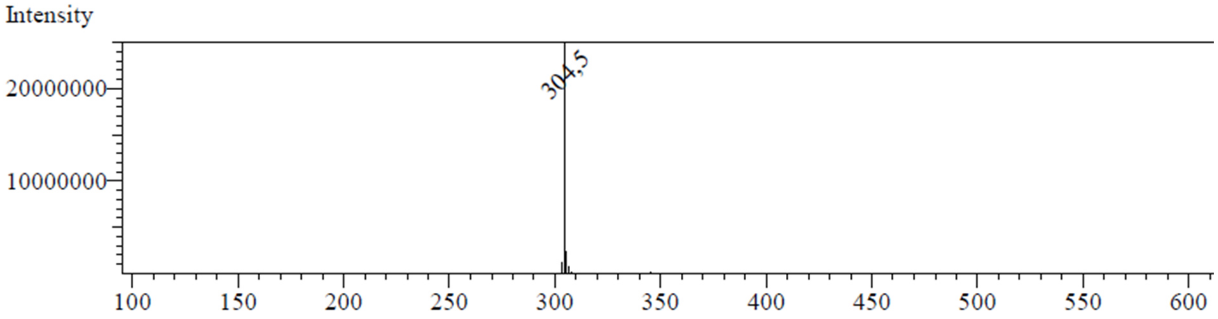

Intensity
20000000
10000000
304,5
100
150
200
250
300
350
400
450
500
550
600

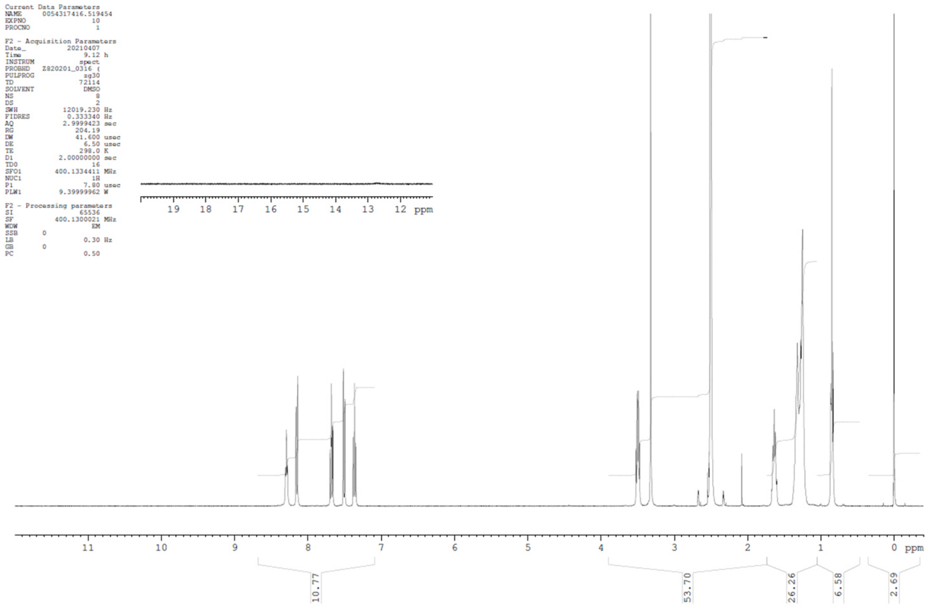

Current Data Parameters
NAME 0054317416.519454
EXPNO 10
PROCNO 1
F2 - Acquisition Parameters
Date_ 20210407
Time 9.12 h
INSTRUM spect
PROBHD Z820201_0316 (
PULPROG zg30
TD 72114
SOLVENT DMSO
NS 8
DS 2
SWH 12019.230 Hz
FIDRES 0.333340 Hz
AQ 2.9999423 sec
RG 204.19
DW 41.600 usec
DE 6.50 usec
TE 298.0 K
D1 2.00000000 sec
TD0 16
SFO1 400.1334411 MHz
NUC1 1H
P1 7.80 usec
PLW1 9.39999962 W
F2 - Processing parameters
SI 65536
SF 400.1300021 MHz
WDW EM
SSB 0
LB 0.30 Hz
GB 0
PC 0.50
19 18 17 16 15 14 13 12 ppm
11 10 9 8 7 6 5 4 3 2 1 0 ppm
10.77
53.70
26.26
6.58
2.69

AQ-9

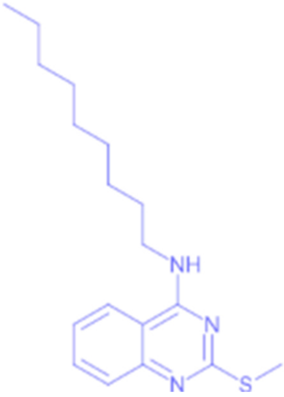
NH
N
N
S

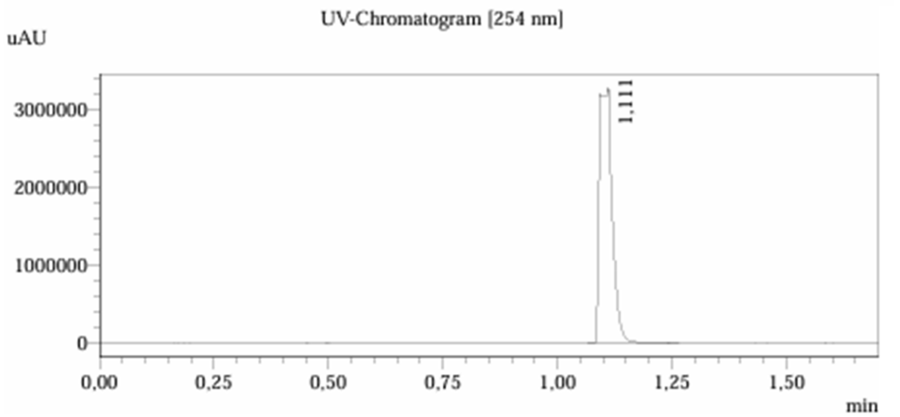
UV-Chromatogram [254 nm]
uAU
3000000
2000000
1000000
0
1,111
0,00
0,25
0,50
0,75
1,00
1,25
1,50
min

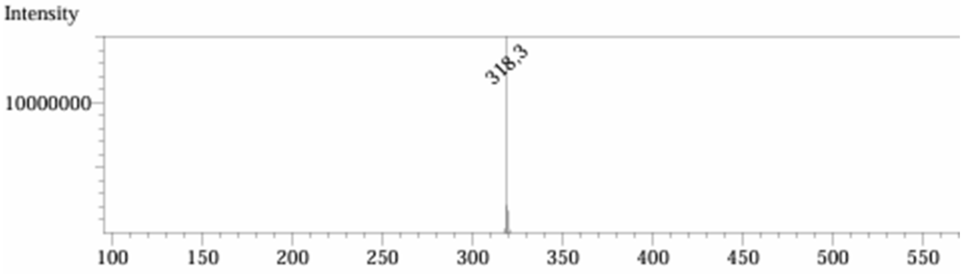
Intensity
10000000
318,3
100
150
200
250
300
350
400
450
500
550

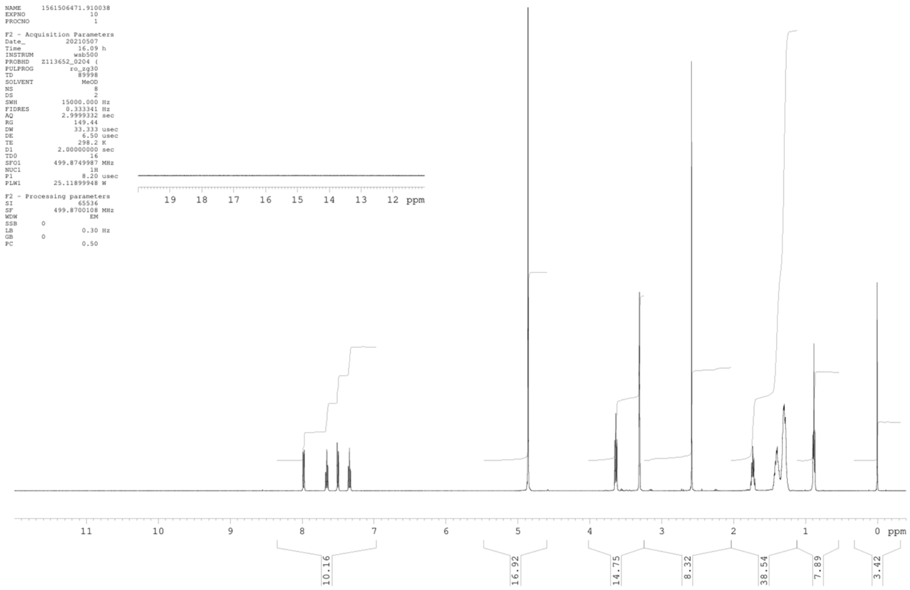
NAME 1561506471.910038
EXPNO 10
PROCNO 1
F2 - Acquisition Parameters
Date_ 20210507
Time 16.09 h
INSTRUM wsb500
PROBHD Z113652_0204 (
PULPROG ro_zg30
TD 89998
SOLVENT MeOD
NS 8
DS 2
SWH 15000.000 Hz
FIDRES 0.333341 Hz
AQ 2.9999332 sec
RG 149.44
DW 33.333 usec
DE 6.50 usec
TE 298.2 K
D1 2.00000000 sec
TD0 16
SFO1 499.8749987 MHz
NUC1 1H
P1 8.20 usec
PLW1 25.11899948 W
F2 - Processing parameters
SI 65536
SF 499.8700108 MHz
WDW EM
SSB 0
LB 0.30 Hz
GB 0
PC 0.50
19 18 17 16 15 14 13 12 ppm
11 10 9 8 7 6 5 4 3 2 1 0 ppm
10.16
16.92
14.75
8.32
38.54
7.89
3.42

AQ-10

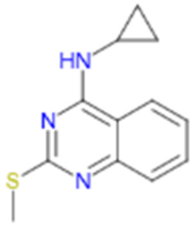

CAS 1879568-22-5; From UkrOrgSynthesis, BBV-47100720

AQ-11

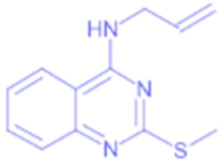

CAS 139047-56-6; From Key Organics, 5N-546S

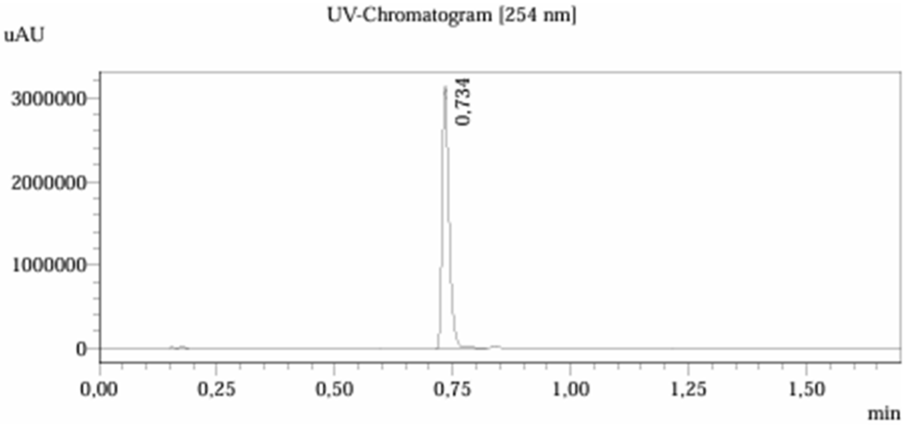

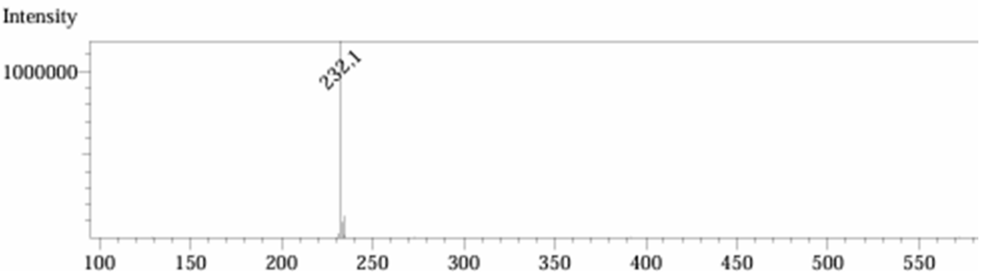

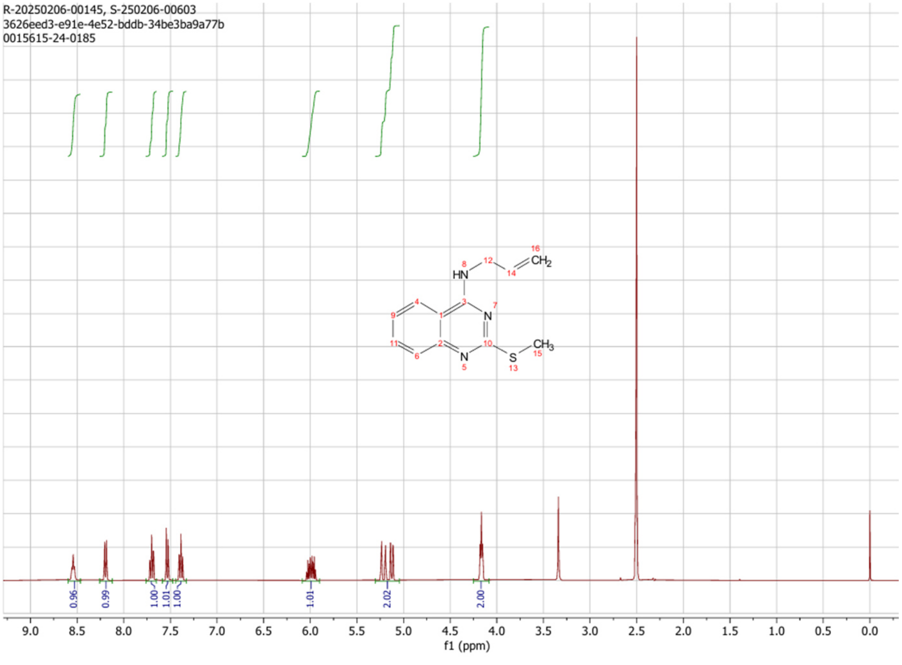

AQ-14

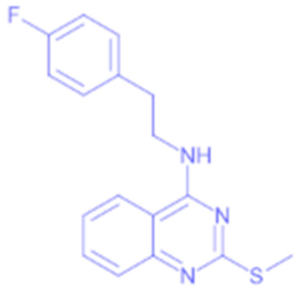
F
NH
N
N
S

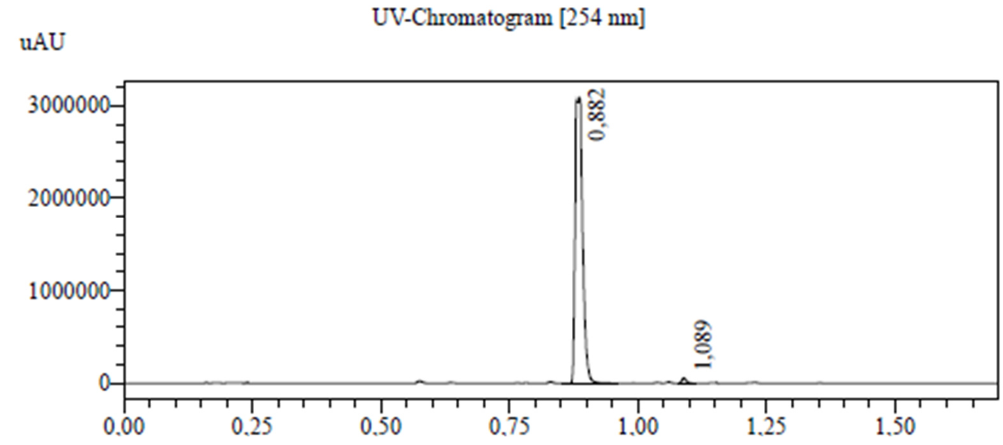
UV-Chromatogram [254 nm]
uAU
3000000
2000000
1000000
0
0,882
1,089
0,00
0,25
0,50
0,75
1,00
1,25
1,50

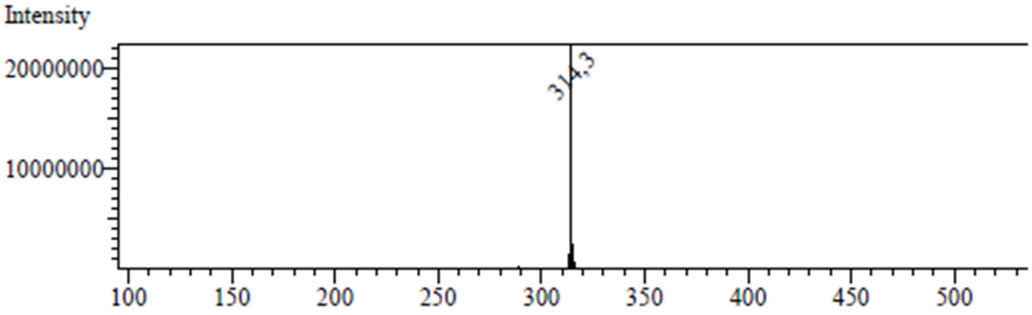
Intensity
20000000
10000000
314,3
100
150
200
250
300
350
400
450
500

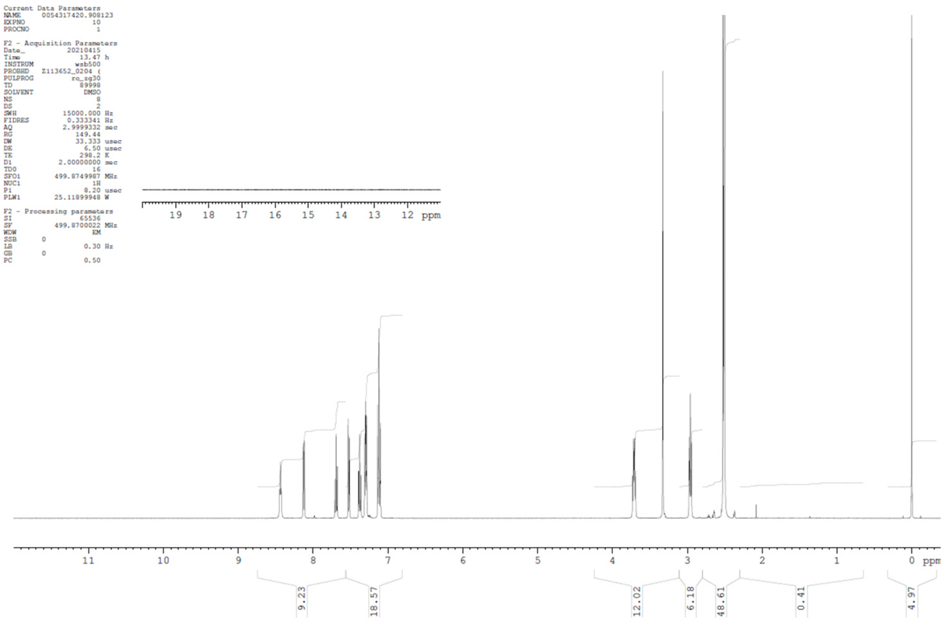
19 18 17 16 15 14 13 12 ppm
11 10 9 8 7 6 5 4 3 2 1 0 ppm
9.23
18.57
12.02
6.18
48.61
0.41
4.97

AQ-25

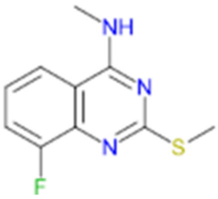

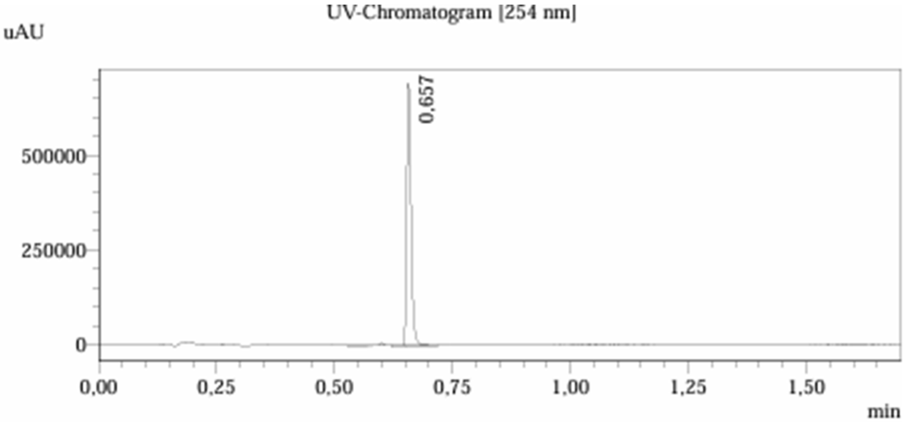
UV-Chromatogram [254 nm]
uAU
500000
250000
0
0,657
0,00
0,25
0,50
0,75
1,00
1,25
1,50
min

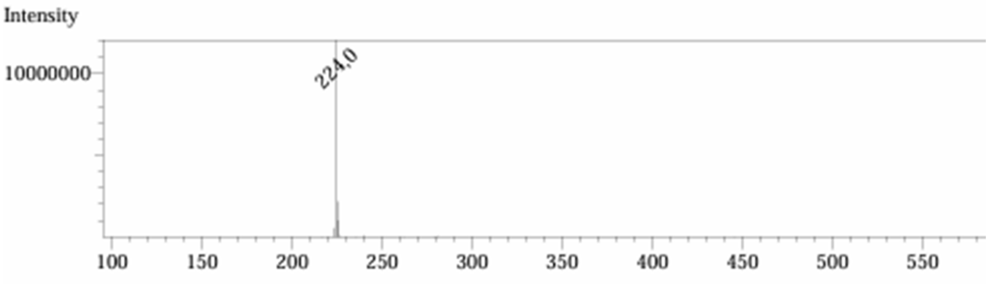
Intensity
10000000
224,0
100
150
200
250
300
350
400
450
500
550

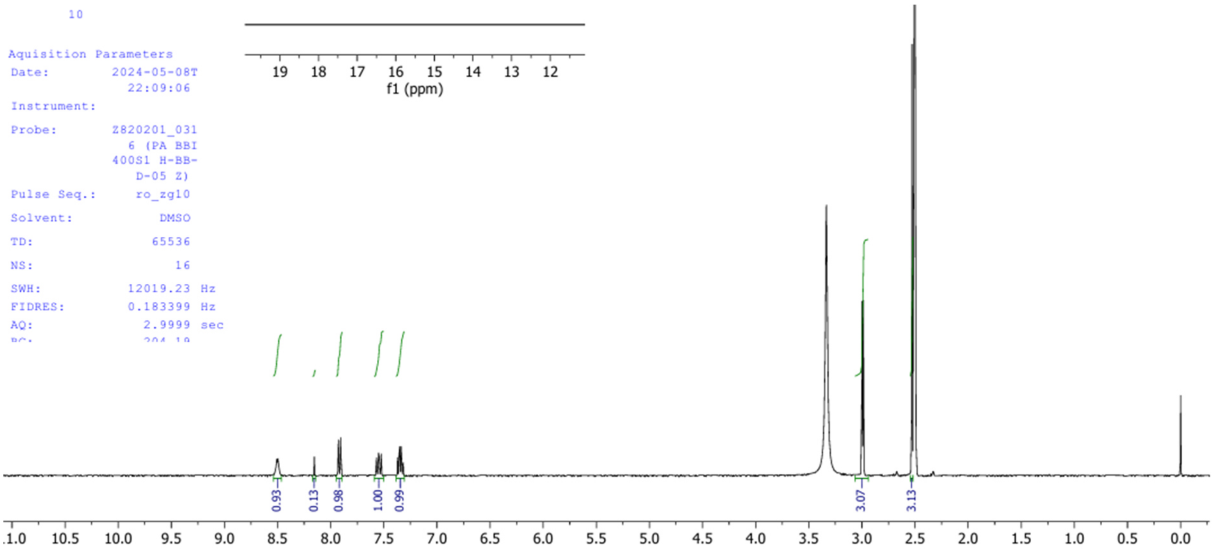
Aquisition Parameters
Date: 2024-05-08T 22:09:06
Instrument:
Probe: Z820201_0316 (PA BBI 400S1 H-BB-D-05 Z)
Pulse Seq.: ro_zg10
Solvent: DMSO
TD: 65536
NS: 16
SWH: 12019.23 Hz
FIDRES: 0.183399 Hz
AQ: 2.9999 sec
0.93
0.13
0.98
1.00
0.99
3.07
3.13
f1 (ppm)

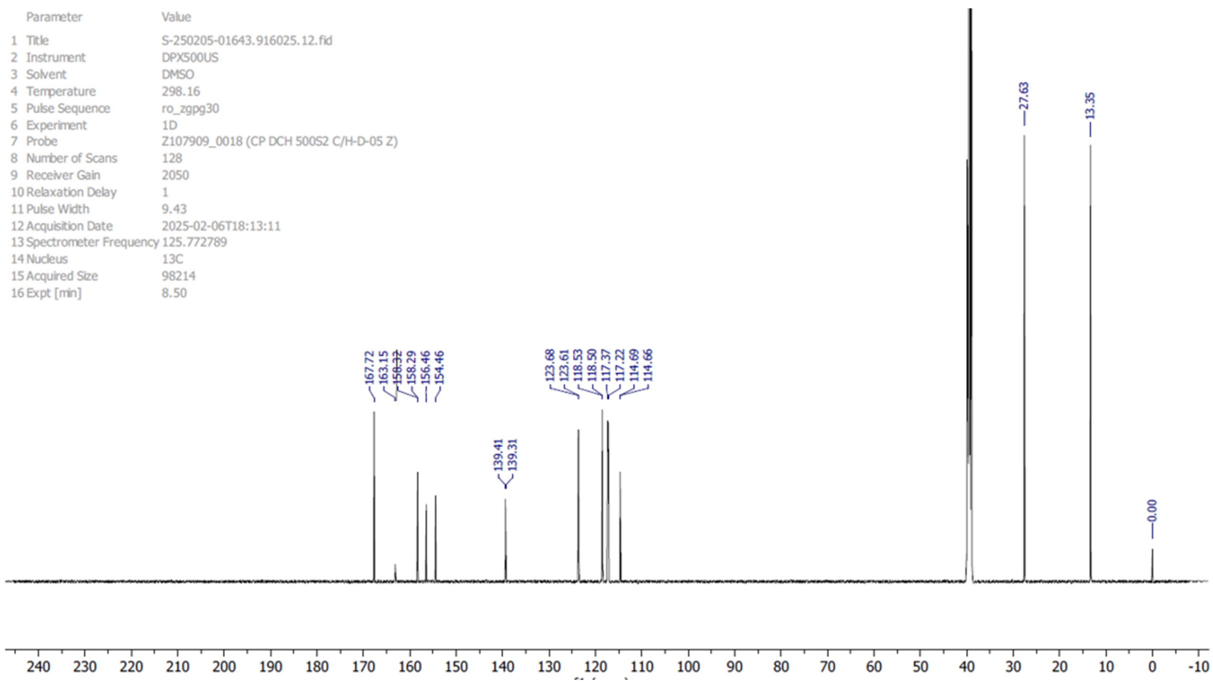
Parameter Value
1 Title S-250205-01643.916025.12.fid
2 Instrument DPX500US
3 Solvent DMSO
4 Temperature 298.16
5 Pulse Sequence ro_zgpg30
6 Experiment 1D
7 Probe Z107909_0018 (CP DCH 500S2 C/H-D-05 Z)
8 Number of Scans 128
9 Receiver Gain 2050
10 Relaxation Delay 1
11 Pulse Width 9.43
12 Acquisition Date 2025-02-06T18:13:11
13 Spectrometer Frequency 125.772789
14 Nucleus 13C
15 Acquired Size 98214
16 Expt [min] 8.50
167.72
163.15
158.32
158.29
156.46
154.46
139.41
139.31
123.68
123.61
118.53
118.50
117.37
117.22
114.69
114.66
27.63
13.35
0.00

AQ-26

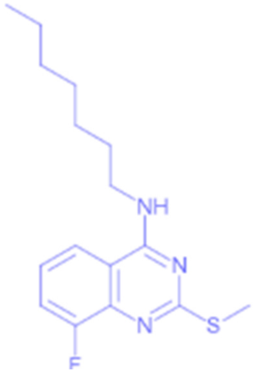
NH
N
N
S
F

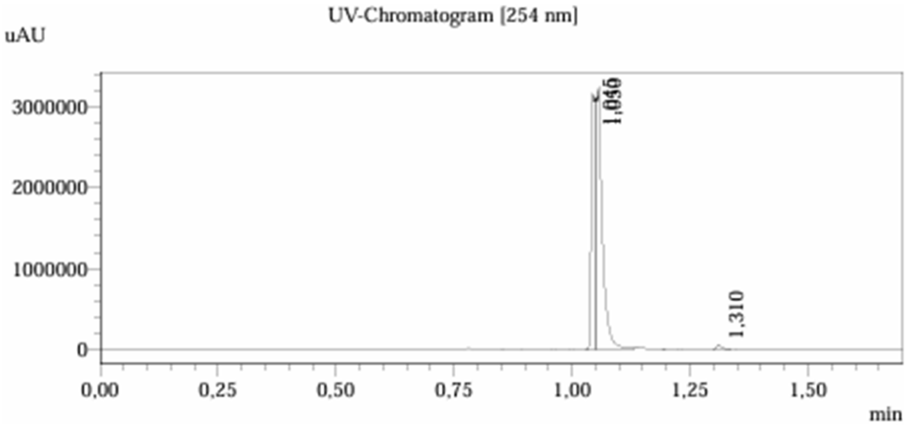
UV-Chromatogram [254 nm]
uAU
3000000
2000000
1000000
0
1,046
1,310
0,00
0,25
0,50
0,75
1,00
1,25
1,50
min

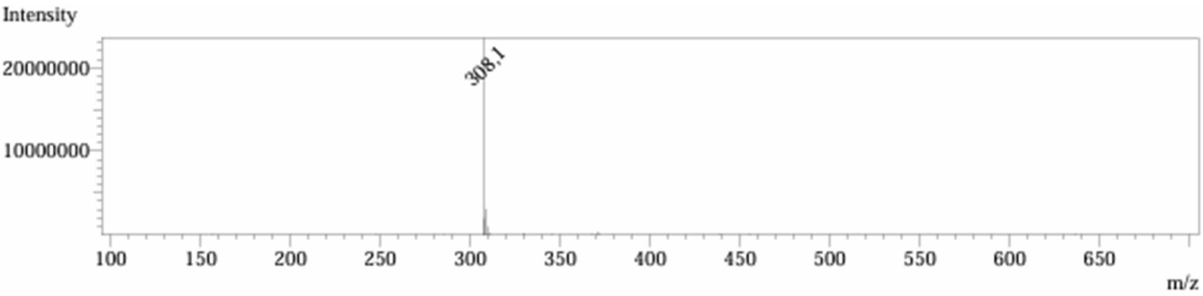
Intensity
20000000
10000000
308,1
100
150
200
250
300
350
400
450
500
550
600
650
m/z

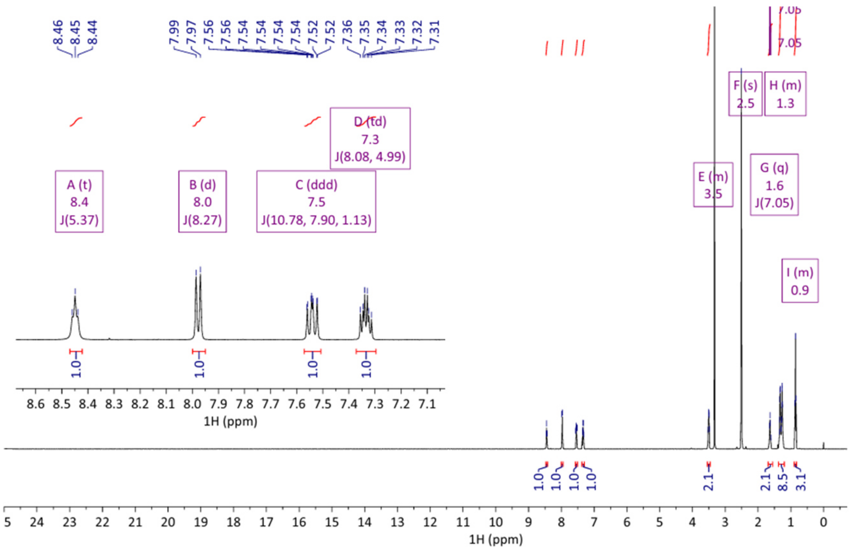
A (t)
8.4
J(5.37)
B (d)
8.0
J(8.27)
C (ddd)
7.5
J(10.78, 7.90, 1.13)
D (td)
7.3
J(8.08, 4.99)
E (m)
3.5
F (s)
2.5
G (q)
1.6
J(7.05)
H (m)
1.3
I (m)
0.9
1H (ppm)

AQ-27

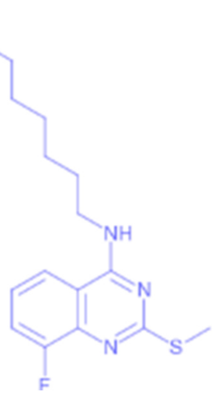

NH
N
N
S
F

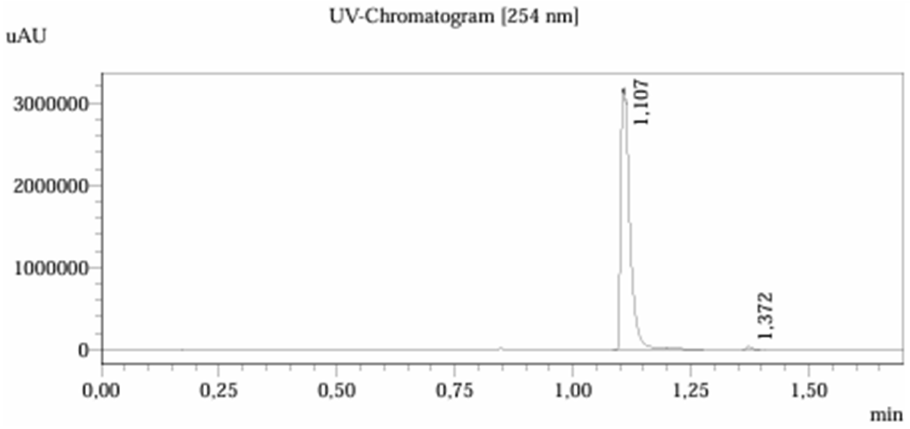

UV-Chromatogram [254 nm]
uAU
3000000
2000000
1000000
0
1,107
1,372
0,00
0,25
0,50
0,75
1,00
1,25
1,50
min

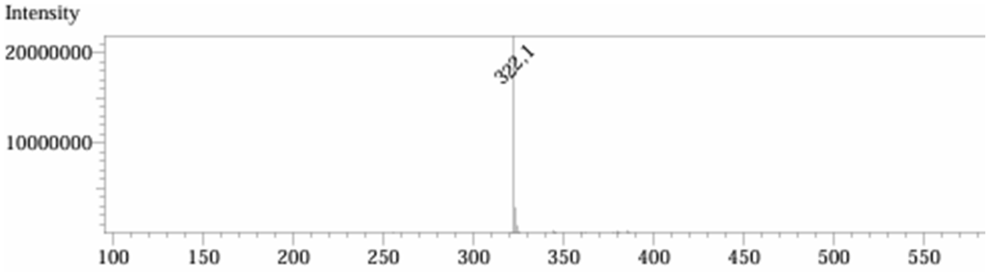

Intensity
20000000
10000000
322,1
100
150
200
250
300
350
400
450
500
550

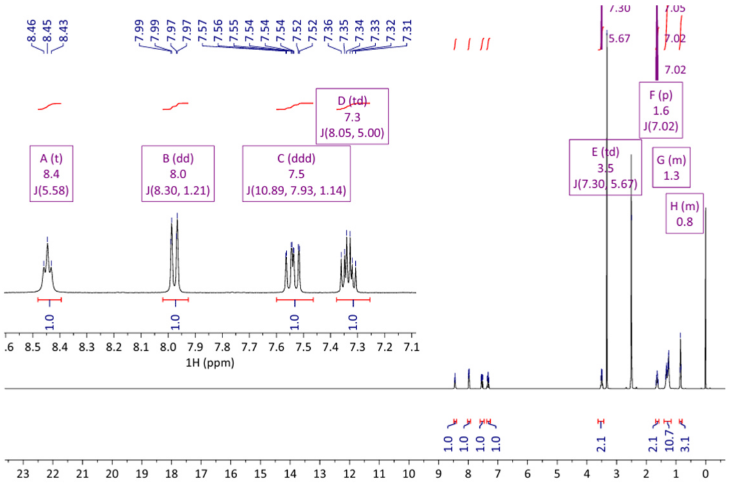

A (t)
8.4
J(5.58)
B (dd)
8.0
J(8.30, 1.21)
C (ddd)
7.5
J(10.89, 7.93, 1.14)
D (td)
7.3
J(8.05, 5.00)
E (td)
3.5
J(7.30, 5.67)
F (p)
1.6
J(7.02)
G (m)
1.3
H (m)
0.8
1H (ppm)

AQ-28

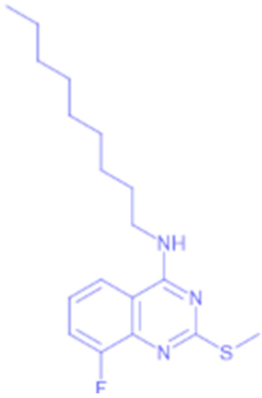

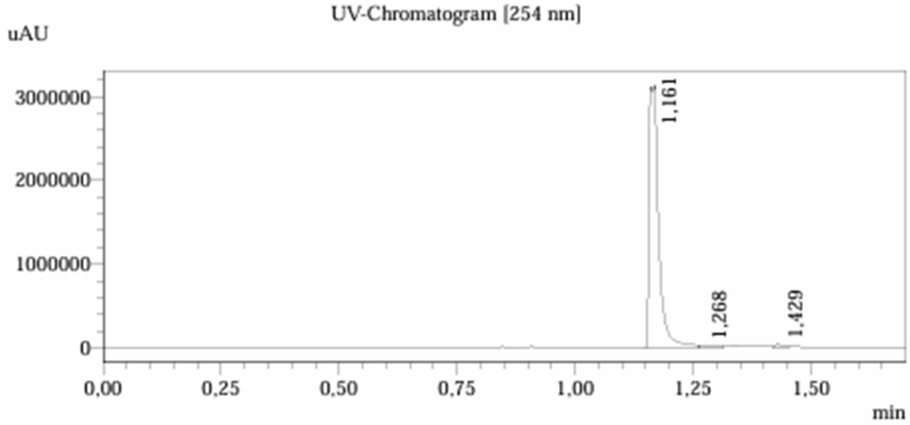
UV-Chromatogram [254 nm]
uAU
3000000
2000000
1000000
0
1,161
1,268
1,429
0,00
0,25
0,50
0,75
1,00
1,25
1,50
min

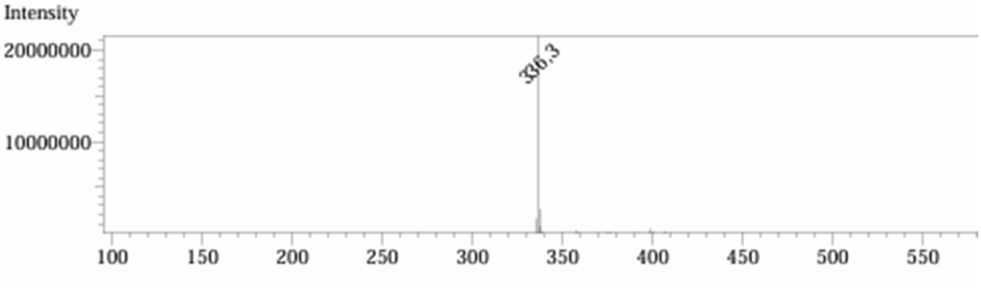
Intensity
20000000
10000000
336,3
100
150
200
250
300
350
400
450
500
550

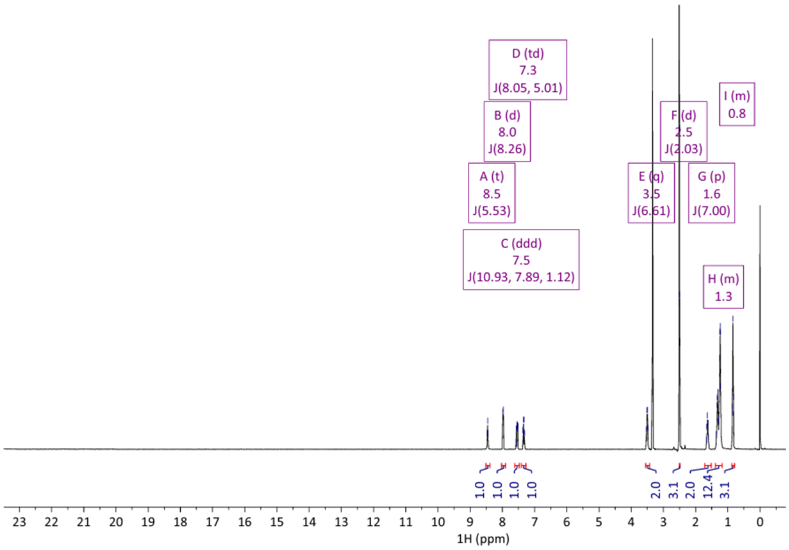
D (td)
7.3
J(8.05, 5.01)
B (d)
8.0
J(8.26)
A (t)
8.5
J(5.53)
C (ddd)
7.5
J(10.93, 7.89, 1.12)
E (q)
3.5
J(6.61)
F (d)
2.5
J(2.03)
I (m)
0.8
G (p)
1.6
J(7.00)
H (m)
1.3
1.0
1.0
1.0
1.0
2.0
3.1
2.0
12.4
3.1
1H (ppm)

AQ-29

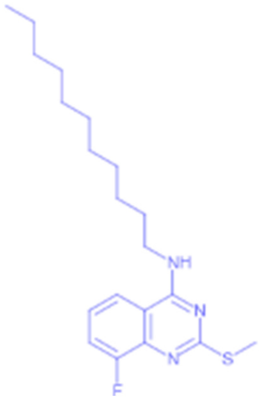
NH
N
N
S
F

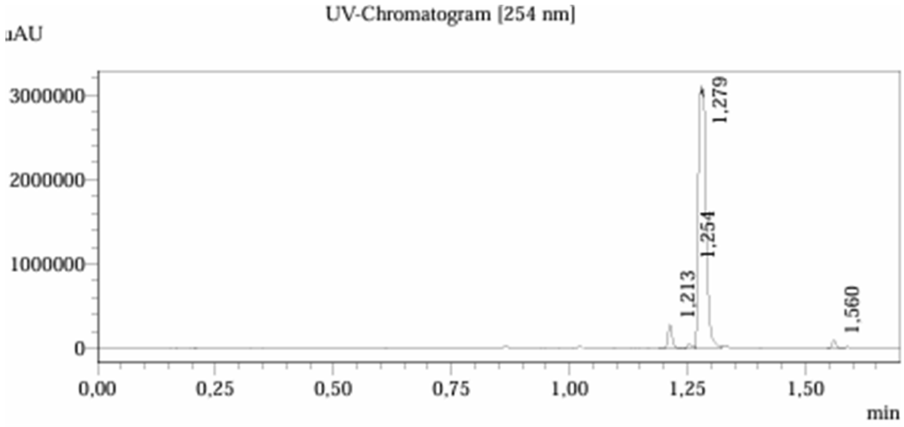
UV-Chromatogram [254 nm]
3000000
2000000
1000000
0
1,279
1,254
1,213
1,560
0,00
0,25
0,50
0,75
1,00
1,25
1,50
min

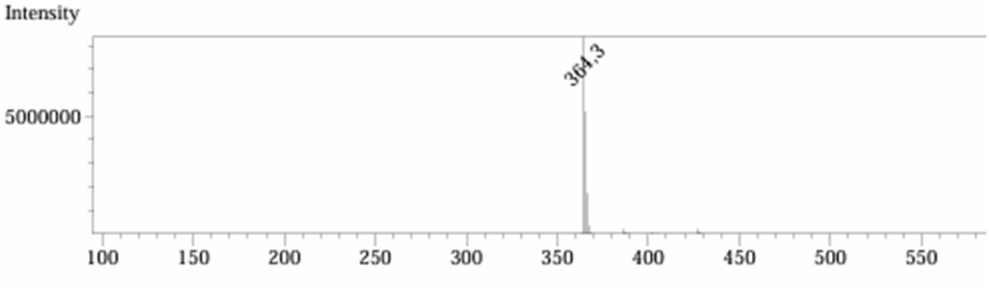
Intensity
5000000
364,3
100
150
200
250
300
350
400
450
500
550

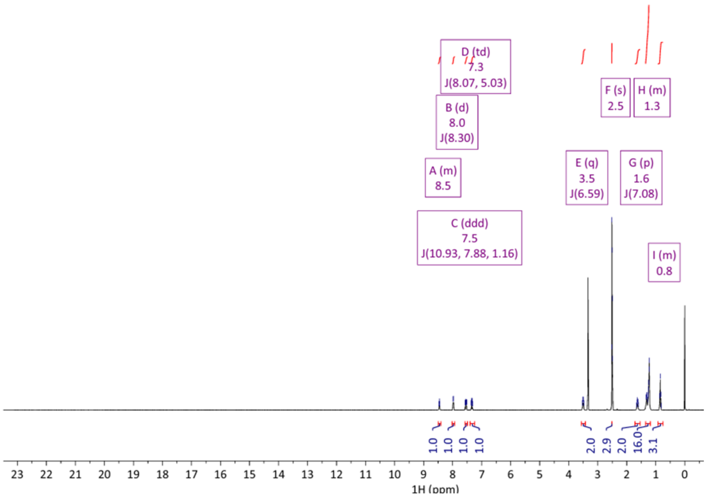
D (td)
7.3
J(8.07, 5.03)
B (d)
8.0
J(8.30)
A (m)
8.5
C (ddd)
7.5
J(10.93, 7.88, 1.16)
F (s)
2.5
H (m)
1.3
E (q)
3.5
J(6.59)
G (p)
1.6
J(7.08)
I (m)
0.8
1.0
1.0
1.0
1.0
2.0
2.9
2.0
16.0
3.1
1H (ppm)

AQ-30

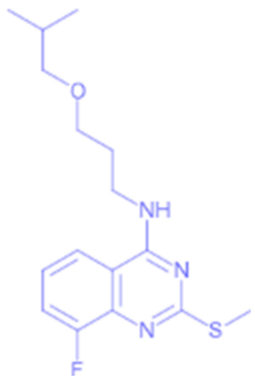

NH
N
N
S
O
F

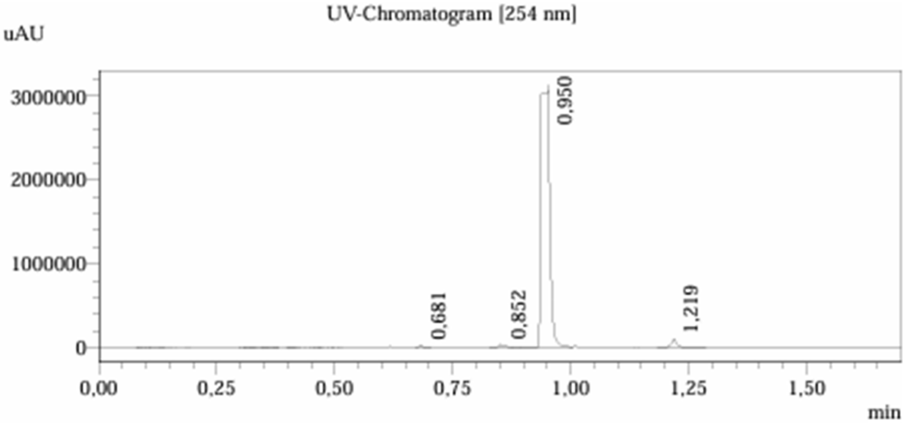

UV-Chromatogram [254 nm]
uAU
3000000
2000000
1000000
0
0,950
0,681
0,852
1,219
0,00
0,25
0,50
0,75
1,00
1,25
1,50
min

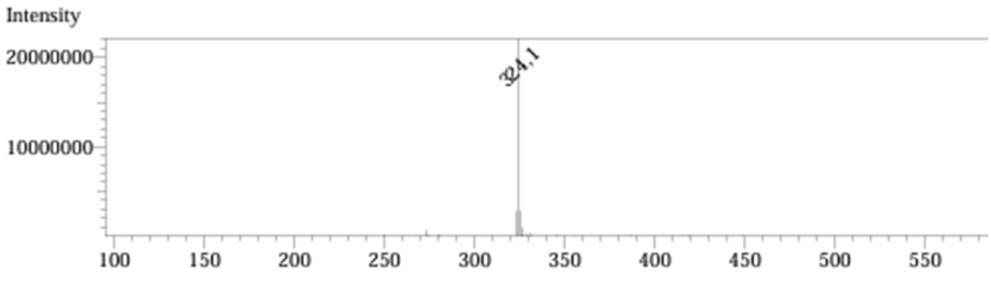

Intensity
20000000
10000000
324,1
100
150
200
250
300
350
400
450
500
550

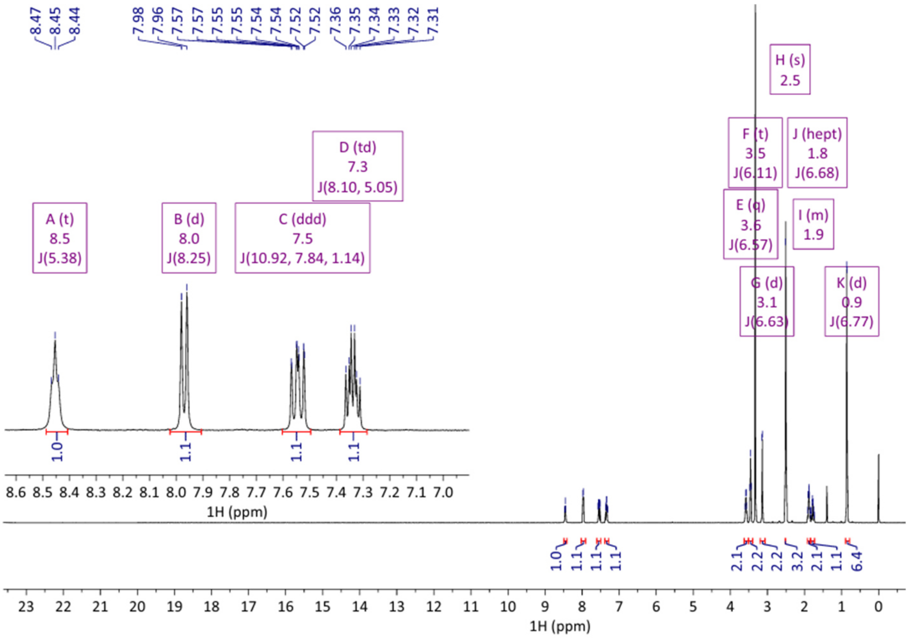

A (t)
8.5
J(5.38)
B (d)
8.0
J(8.25)
C (ddd)
7.5
J(10.92, 7.84, 1.14)
D (td)
7.3
J(8.10, 5.05)
E (q)
3.6
J(6.57)
F (t)
3.5
J(6.11)
G (d)
3.1
J(6.63)
H (s)
2.5
J (hept)
1.8
J(6.68)
I (m)
1.9
K (d)
0.9
J(6.77)
1H (ppm)

AQ-31

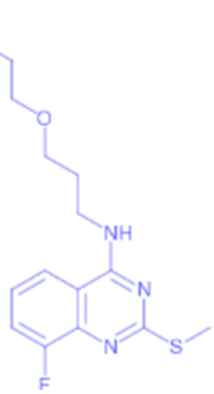

O
NH
N
N
S
F

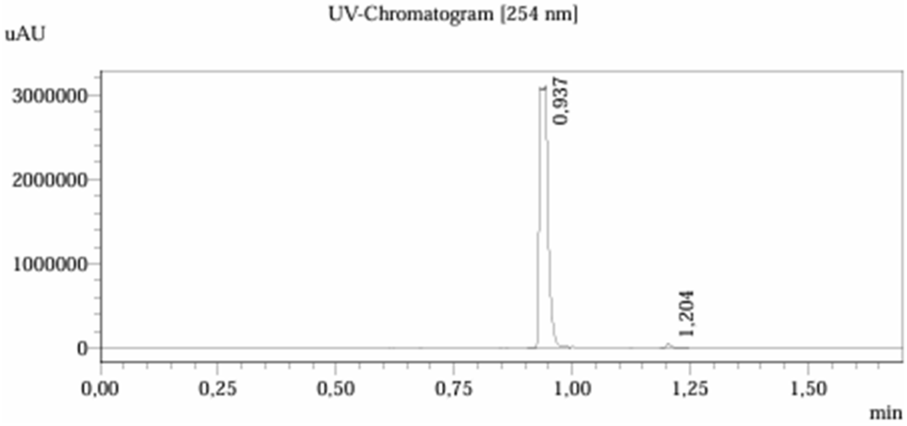

UV-Chromatogram [254 nm]
uAU
3000000
2000000
1000000
0
0,937
1,204
0,00
0,25
0,50
0,75
1,00
1,25
1,50
min

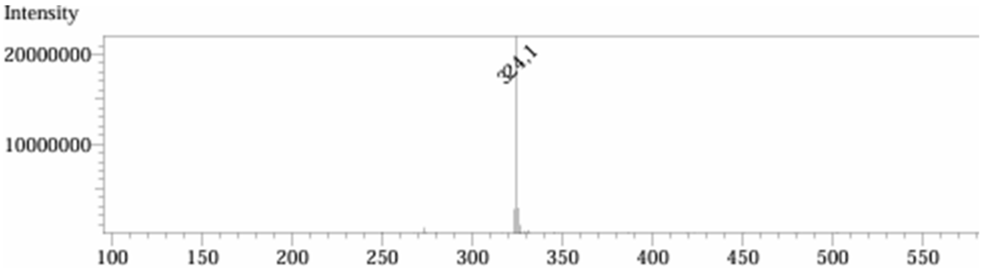

Intensity
20000000
10000000
324,1
100
150
200
250
300
350
400
450
500
550

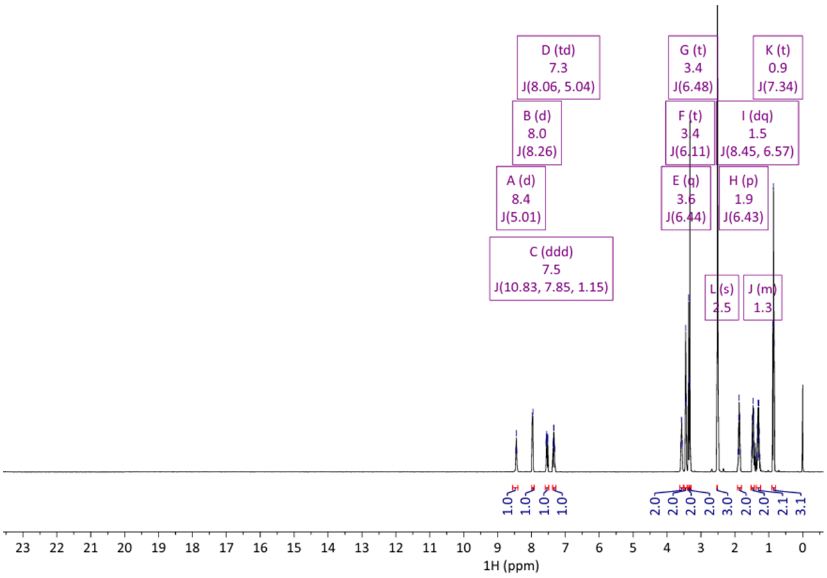

D (td) 7.3 J(8.06, 5.04)
B (d) 8.0 J(8.26)
A (d) 8.4 J(5.01)
C (ddd) 7.5 J(10.83, 7.85, 1.15)
G (t) 3.4 J(6.48)
F (t) 3.4 J(6.11)
E (q) 3.6 J(6.44)
K (t) 0.9 J(7.34)
I (dq) 1.5 J(8.45, 6.57)
H (p) 1.9 J(6.43)
L (s) 2.5
J (m) 1.3
1.0
1.0
1.0
1.0
2.0
2.0
2.0
2.0
3.0
2.0
2.0
2.1
3.1
1H (ppm)

	Parameter	Value
1	Title	S-250205-01655.916033.12.fid
2	Instrument	DPX500US
3	Solvent	DMSO
4	Temperature	298.16
5	Pulse Sequence	ro_zgpg30
6	Experiment	1D
7	Probe	Z107909_0018 (CP DCH 500S2 C/H-D-05 Z)
8	Number of Scans	128
9	Receiver Gain	2050
10	Relaxation Delay	1
11	Pulse Width	9.43
12	Acquisition Date	2025-02-06T19:13:40
13	Spectrometer Frequency	125.772789
14	Nucleus	13C
15	Acquired Size	98214
16	Expt [min]	8.50

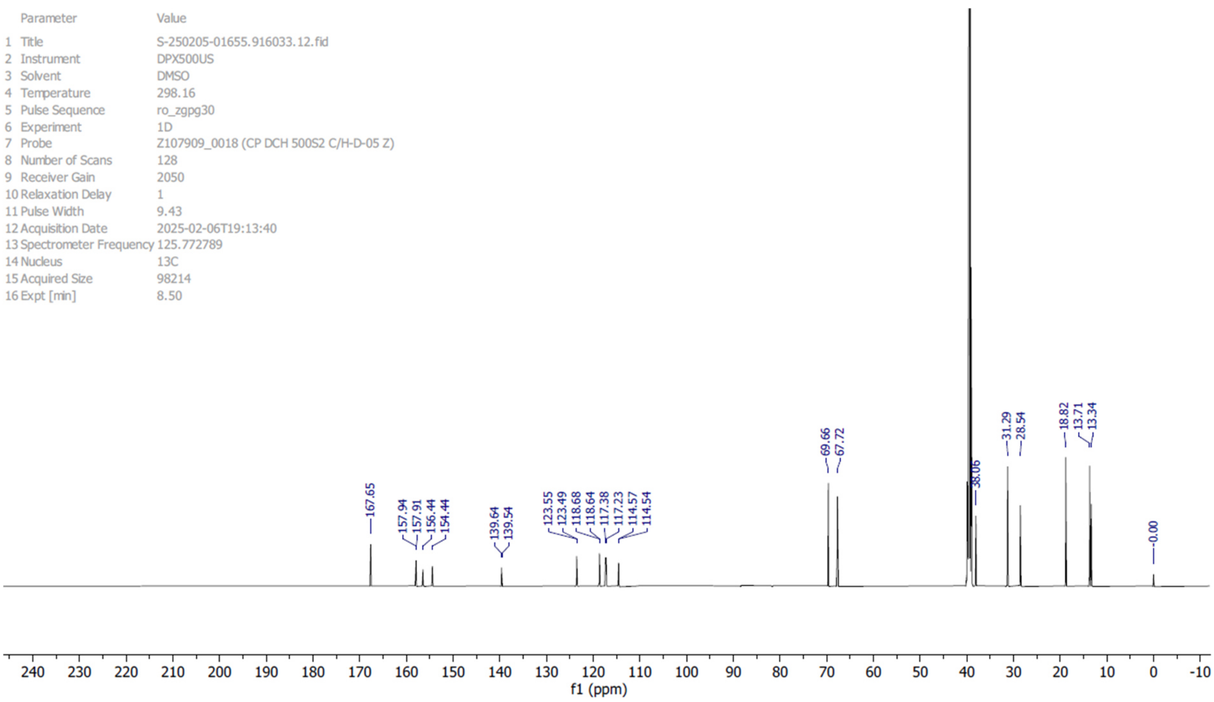

AQ-32

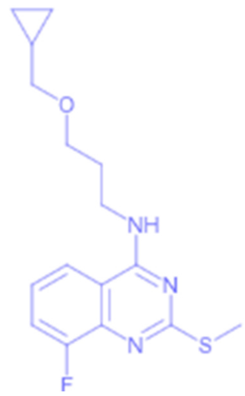
NH
N
N
S
O
F

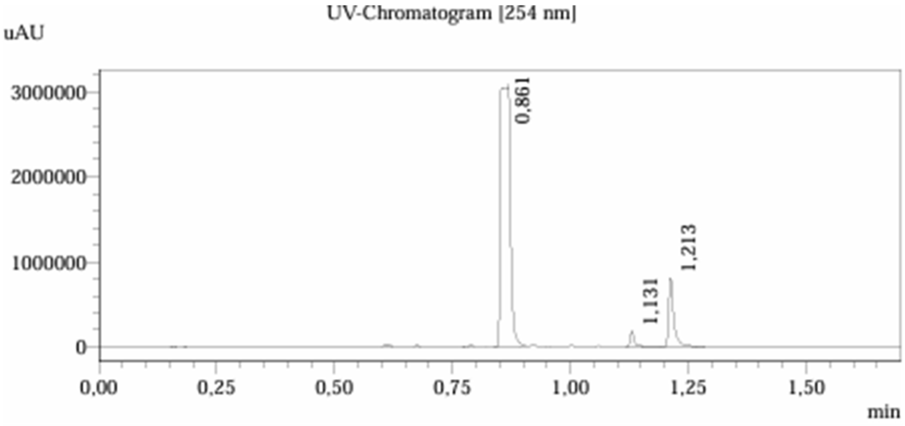
UV-Chromatogram [254 nm]
uAU
0,861
1,131
1,213
min

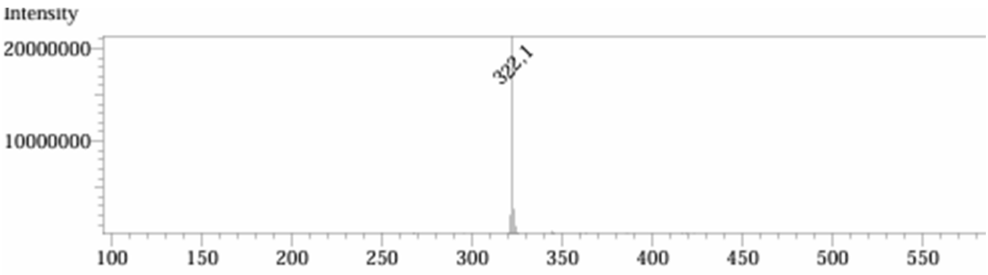
Intensity
322,1

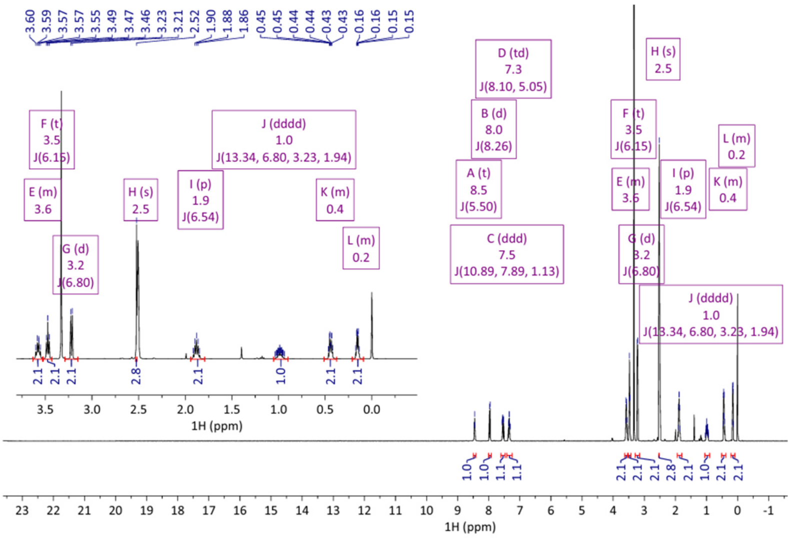
A (t) 8.5 J(5.50)
B (d) 8.0 J(8.26)
C (ddd) 7.5 J(10.89, 7.89, 1.13)
D (td) 7.3 J(8.10, 5.05)
E (m) 3.6
F (t) 3.5 J(6.15)
G (d) 3.2 J(6.80)
H (s) 2.5
I (p) 1.9 J(6.54)
J (dddd) 1.0 J(13.34, 6.80, 3.23, 1.94)
K (m) 0.4
L (m) 0.2
1H (ppm)

AQ-33

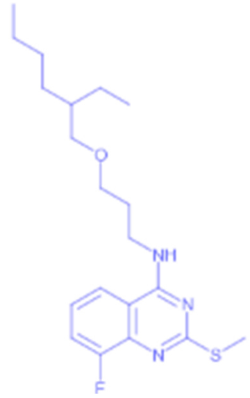

NH
N
N
S
F

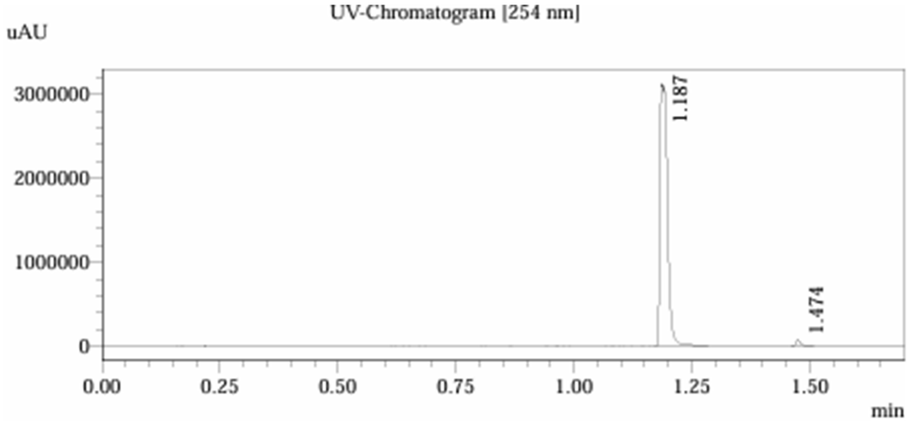

UV-Chromatogram [254 nm]
uAU
3000000
2000000
1000000
0
1.187
1.474
0.00
0.25
0.50
0.75
1.00
1.25
1.50
min

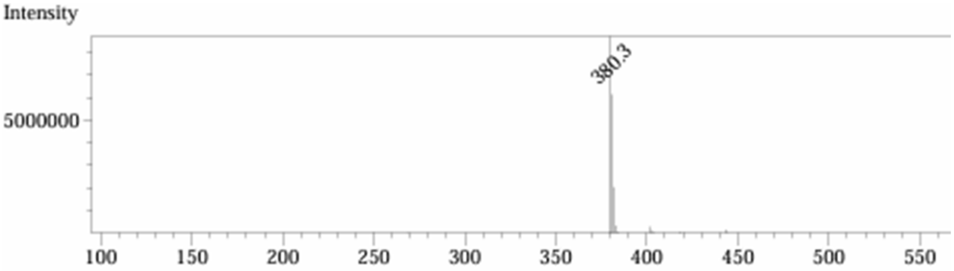

Intensity
5000000
380.3
100
150
200
250
300
350
400
450
500
550

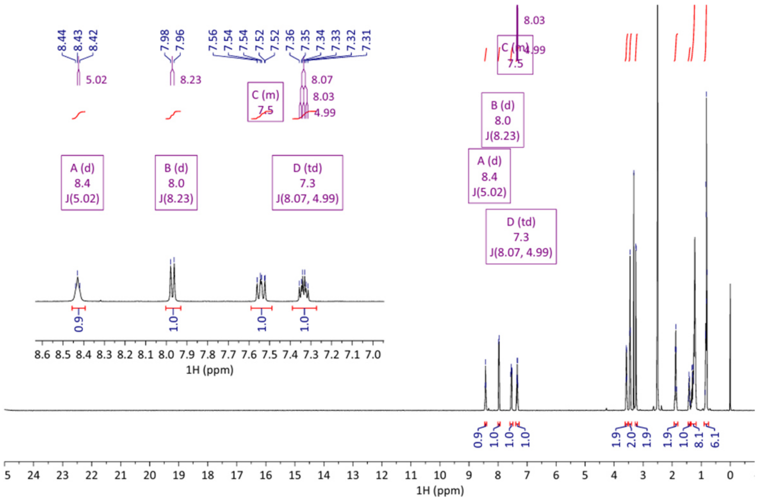

A (d)
8.4
J(5.02)
B (d)
8.0
J(8.23)
C (m)
7.5
D (td)
7.3
J(8.07, 4.99)
1H (ppm)

AQ-34

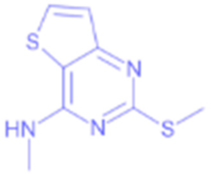

CAS 343374-14-1

From Key Organics, 6N-424S

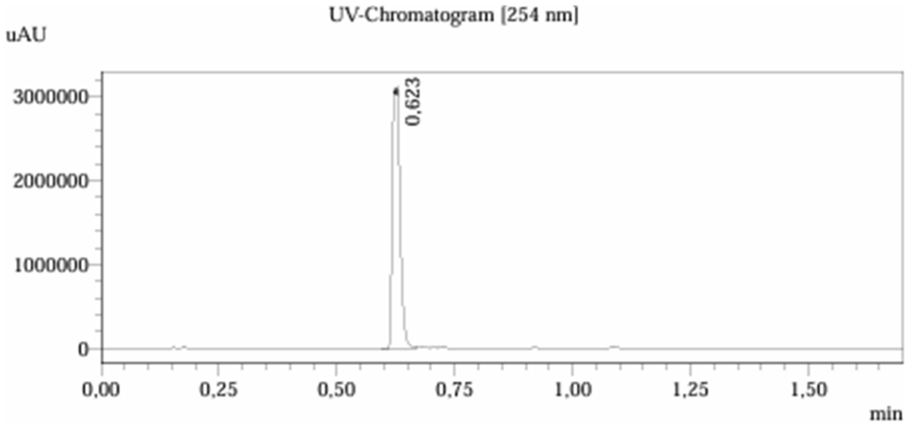

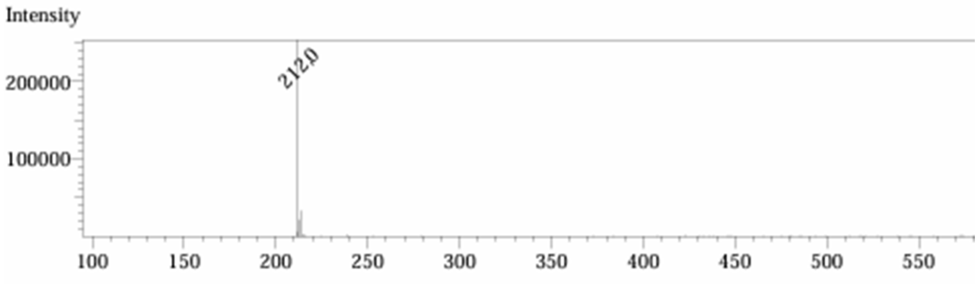

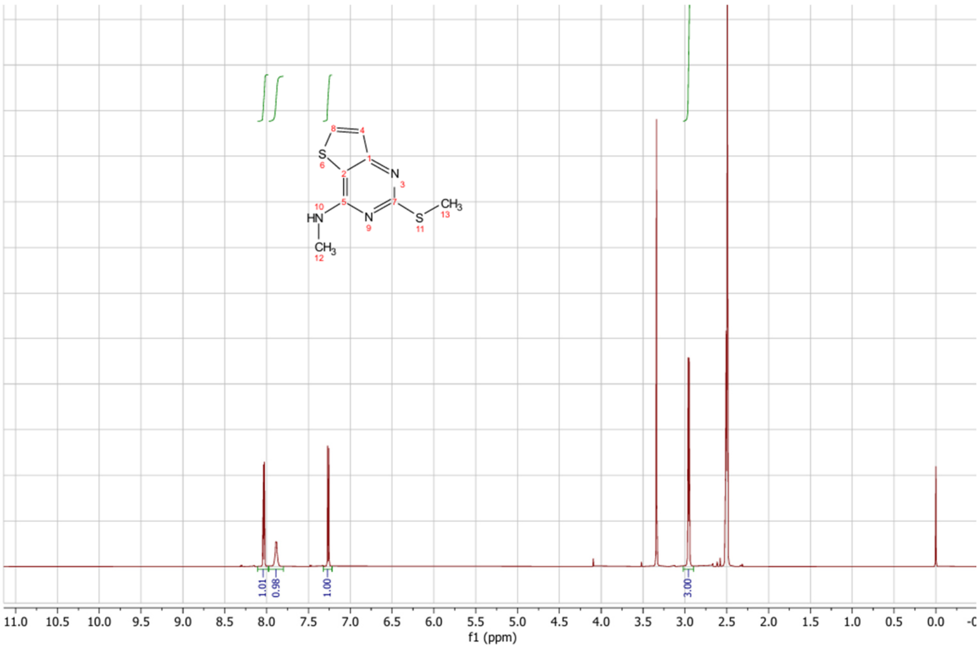

AQ-36

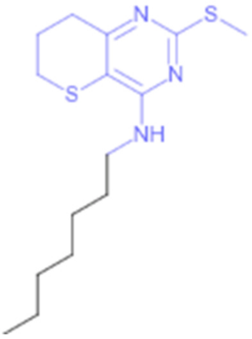
N
S
N
S
NH

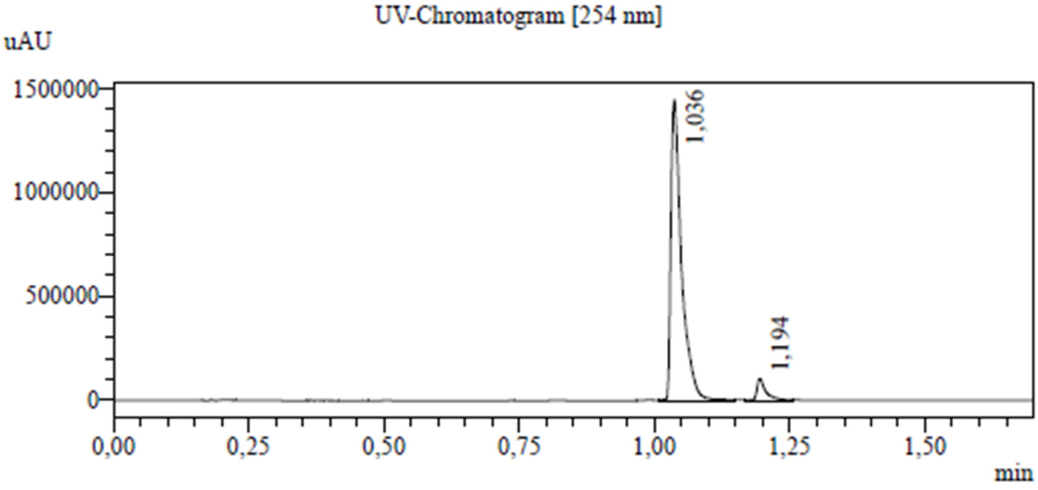
UV-Chromatogram [254 nm]
uAU
1500000
1000000
500000
0
1,036
1,194
0,00
0,25
0,50
0,75
1,00
1,25
1,50
min

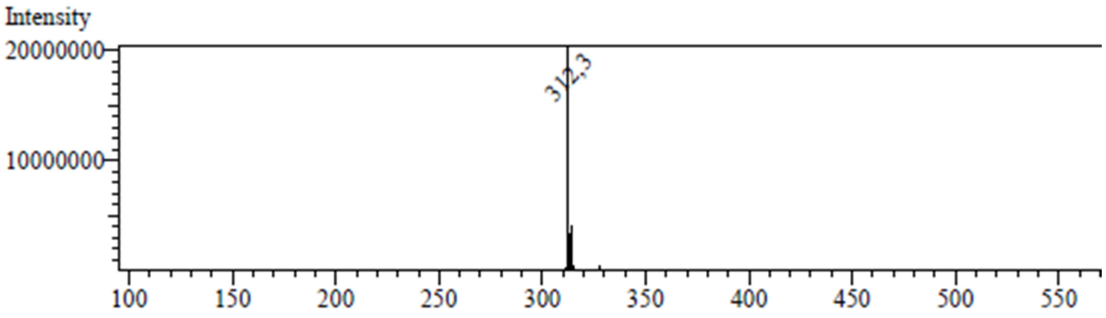
Intensity
20000000
10000000
312,3
100
150
200
250
300
350
400
450
500
550

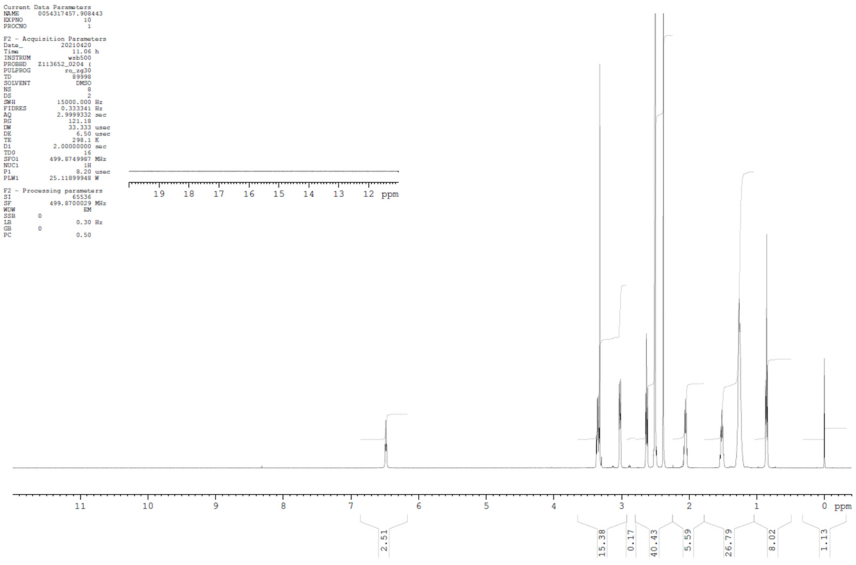
19 18 17 16 15 14 13 12 ppm
11 10 9 8 7 6 5 4 3 2 1 0 ppm
2.51
15.38
0.17
40.43
5.59
26.79
8.02
1.13

AQ-37

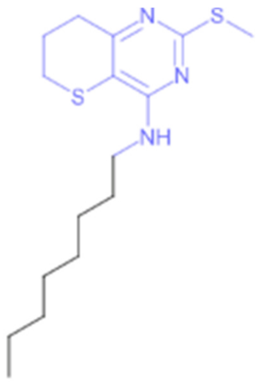

N
S
N
S
NH

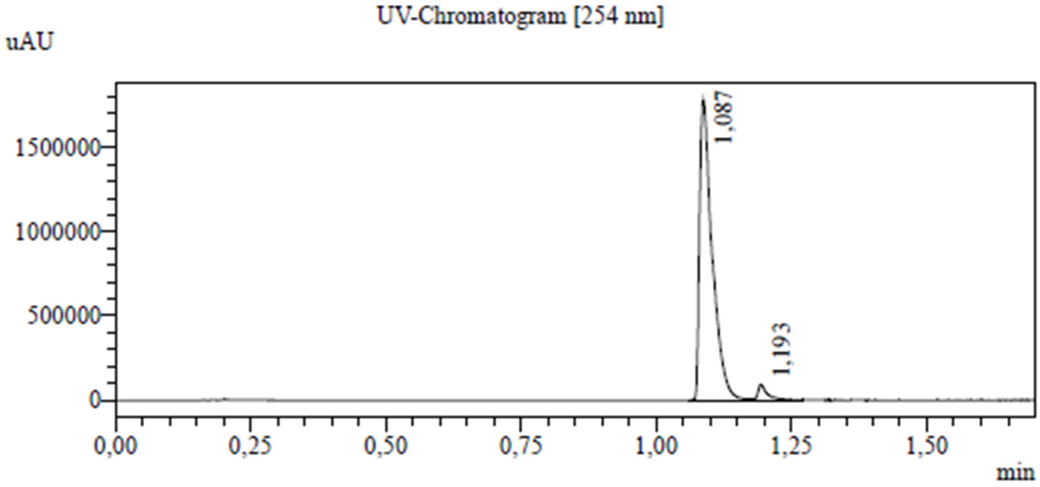

UV-Chromatogram [254 nm]
uAU
1500000
1000000
500000
0
1,087
1,193
0,00
0,25
0,50
0,75
1,00
1,25
1,50
min

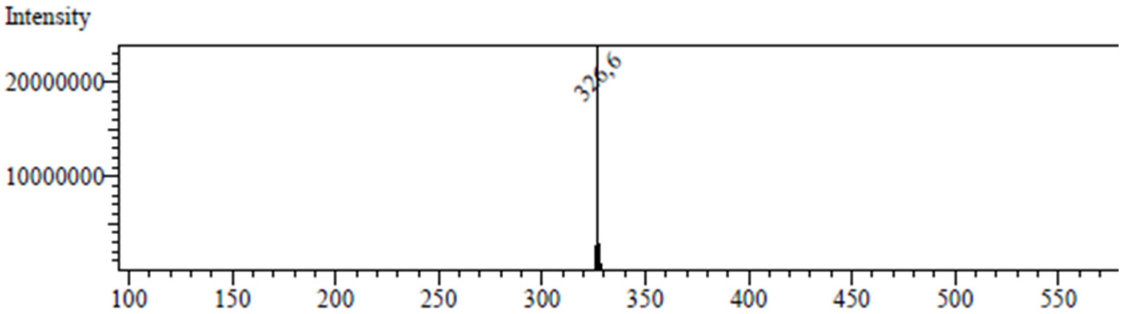

Intensity
20000000
10000000
326,6
100
150
200
250
300
350
400
450
500
550

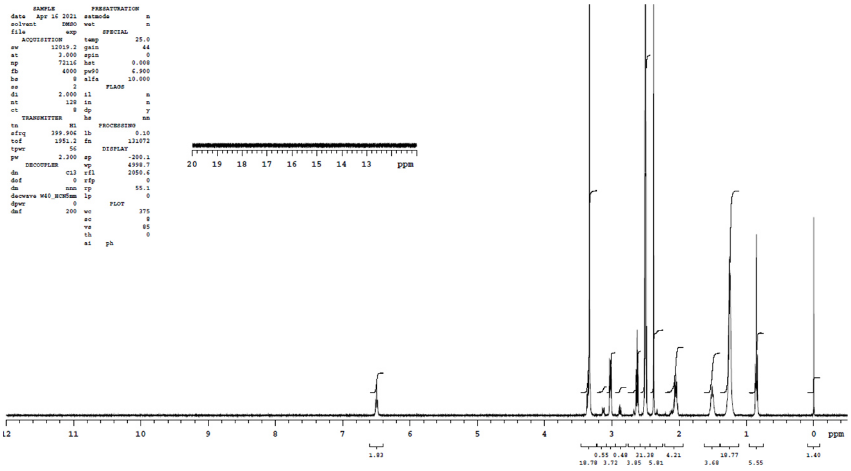

AQ-38

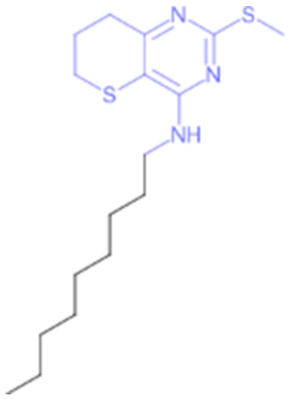
N
S
N
S
NH

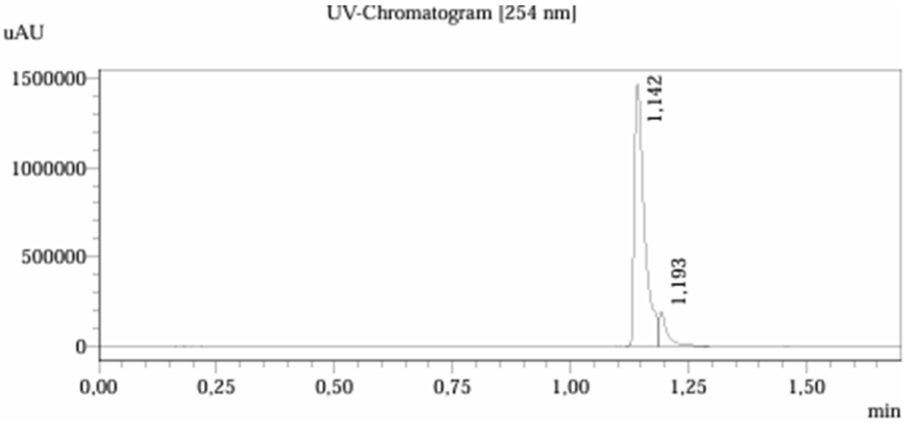
UV-Chromatogram [254 nm]
uAU
1500000
1000000
500000
0
1,142
1,193
0,00
0,25
0,50
0,75
1,00
1,25
1,50
min

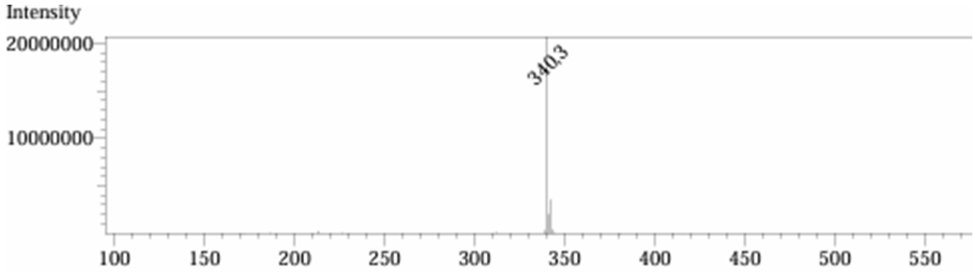
Intensity
20000000
10000000
340,3
100
150
200
250
300
350
400
450
500
550

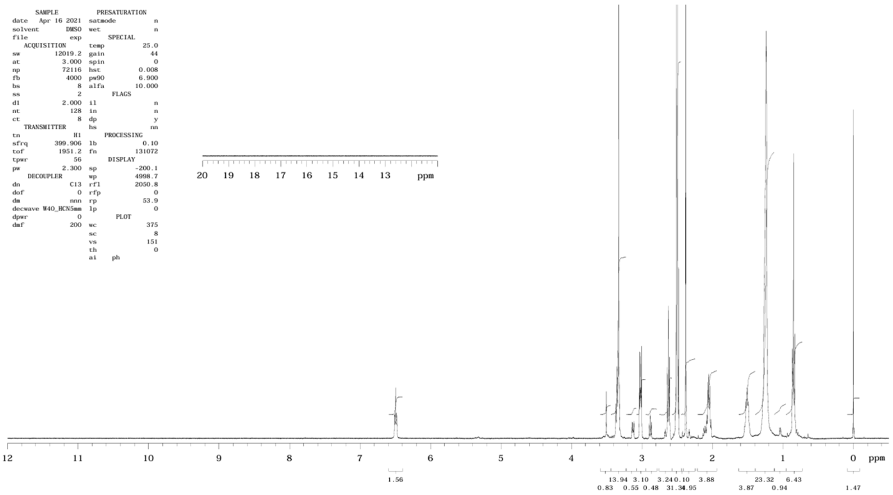
12
11
10
9
8
7
6
5
4
3
2
1
0
ppm
1.56
1.47
